# Conceptualizations of well-being in adults with visual impairment: A scoping review

**DOI:** 10.3389/fpsyg.2022.964537

**Published:** 2022-09-26

**Authors:** Nikki Heinze, Ffion Davies, Lee Jones, Claire L. Castle, Renata S. M. Gomes

**Affiliations:** ^1^BRAVO VICTOR, Research, London, United Kingdom; ^2^Blind Veterans UK, London, United Kingdom; ^3^Northern Hub for Veterans and Military Families Research, Department of Nursing, Midwifery and Health, Faculty of Health and Life Sciences, Northumbria University, Newcastle upon Tyne, United Kingdom

**Keywords:** well-being, visual impairment, sight loss, conceptualization, scoping review

## Abstract

**Background:**

Despite its ubiquity, it is often not clear what organizations and services mean by well-being. Visual impairment (VI) has been associated with poorer well-being and well-being has become a key outcome for support and services for adults living with VI. A shared understanding of what well-being means is therefore essential to enable assessment of well-being and cross-service provision of well-being support.

**Objectives:**

To provide an overview of the ways in which well-being has been conceptualized in research relating to adults living with VI.

**Eligibility criteria:**

Articles were included in the review if the article discussed well-being in the context of adults living with VI, was available in English and as a full text.

**Data sources:**

A systematic search using search terms relating to VI and well-being was conducted in EBSCOHost (Medline, CINHL) and Ovid (Embase Classic, Embase, Emcare 1995, Health + Psychosocial, HMIC Health Management Info, APA, PsycArticles, PsycInfo, PsycTests).

**Charting:**

A team of three reviewers screened titles, abstracts and full-texts articles and extracted data. Ambiguous articles were referred to the research group and discussed.

**Results:**

Of 10,662 articles identified in the search, 249 were included in the review. These referred to 38 types of well-being. The most common types were general well-being (*n* = 101; 40.6%) emotional well-being (*n* = 86, 34.5%) and psychological well-being (*n* = 66, 26.5%). Most articles (*n* = 150; 60.2%) referred to one type only, with a maximum of 9 listed in one article. A large number of articles did not clearly define well-being. A wide range of indicators of well-being related to the domains of hedonia, mood, positive and negative affect, quality of life, mental health, eudaimonia, self/identity, health, psychological reactions to disability and health problems, functioning, social functioning and environment, were extracted, many of which were used just once.

**Conclusions:**

There remains a lack of consensus on how well-being is conceptualized and assessed in the context of adult VI. A standardized multi-domain approach derived with input from adults with VI and practitioners working with them is required to enable comparison of findings and cross-organizational provision of support.

## Introduction

In recent years, well-being has become ubiquitous. It is a key concern across a wide range of fields including the media, politics, organizations, academia and public health. Despite, or perhaps because of, this focus on well-being across different fields, it is often unclear what is meant by well-being. This lack of clarity is reflected in the academic literature. Dodge et al. (2012) point to the challenges of defining well-being, a term described as “commonly used but inconsistently defined” in research (Pollard and Lee, 2003, p. 62). Several models of well-being have been proposed. Hedonic models, for instance, focus on how people are feeling. A widely recognized hedonic model is that of subjective well-being (SWB) (Diener, 1984; Diener and Suh, 1997) consisting of pleasant/unpleasant affect and the cognitive component of life satisfaction. In contrast, eudaimonic models focus on how people are functioning and encompass a combination of aspects related to personal growth and fulfillment. For instance, the model of psychological well-being (PWB) (Ryff, 1989) was developed around six dimensions: autonomy, environmental mastery, positive relationships with others, purpose in life, realization of potential and self-acceptance, while self-determination theory (SDT) comprises autonomy, competence, and relatedness (Ryan and Deci, 2001). Hybrid models, which involve aspects of hedonia and eudaimonia, have been developed to provide a more complete picture of human well-being. Keyes (2002) theorized that flourishing, for instance, referred to a state of positive mental health consisting of emotional (hedonic), psychological (PWB) and social well-being (social functioning). Similarly, Seligman (2012) proposed a hybrid model of well-being which consisted of positive emotions, engagement, relationships, meaning, and accomplishment (PERMA). In addition, related concepts such as quality of life (QoL) (Lent, 2004; Cooke et al., 2016; Linton et al., 2016), happiness (Linton et al., 2016), health (Linton et al., 2016) and wellness (Cooke et al., 2016; Linton et al., 2016) are used interchangeably with well-being. A more recent attempt to define well-being distinguished between descriptions of well-being, which focus on indicators, and definitions, conceptualizing it as the balance point between the psychological, physical and social challenges faced by an individual and the resources available to them (Dodge et al., 2012).

The breadth of definitions is reflected in the way well-being is assessed. A review of 42 well-being measures found considerable differences in the way they conceptualized well-being, and the type and number of indicators used to assess well-being (Cooke et al., 2016). Similarly, Linton et al. (2016) identified 196 distinct dimensions across 99 well-being measures clustering around six domains (mental well-being, social well-being, activities and functioning, physical well-being, spiritual well-being, personal circumstances), with no clear distinction being made between indicators of well-being, factors which factors which impact well-being, and outcomes. While generally accepted to be a multi-dimensional construct (Pollard and Lee, 2003; Linton et al., 2016), a systematic review of child well-being found that 80% of studies assessed only one of five proposed dimensions (cognitive, economic, physical, psychological and social) using a wide range of measures (Pollard and Lee, 2003), pointing to a lack of clarity around what is being measured. In addition, as assessments of health outcomes have shifted away from a focus on symptoms to a more holistic approach which includes affective responses and life satisfaction (McDowell, 2010), adequate measures are required to assess the impact of health conditions and interventions. McDowell (2010) reviewed nine tools commonly used to assess psychological well-being. The scales assessed a range of aspects of well-being including life satisfaction, affect, morale and elements related to eudaimonia (self-acceptance, positive relations with others, autonomy, environmental mastery, purpose in life, personal growth). He concluded that while these scales were adequate for survey research, their validity as health outcomes measures remains largely unclear.

The lack of conceptual clarity is reflected in assessments of well-being in the context of visual impairment (VI). VI refers to a reduction in visual sensitivity which cannot be corrected by standard eyeglasses or medical treatment. In the United Kingdom (UK) alone, it is estimated that, as a result of an aging population, the number of people living with a form of VI is going to increase from around 2 to 4 million by 2050 (Pezzullo et al., 2018). VI has been associated with poorer mental health, social functioning, quality of life and well-being (Pinquart and Pfeiffer, 2011; Fenwick et al., 2012b; Kempen et al., 2012; Zhang et al., 2013; van der Aa et al., 2015; Garcia et al., 2017; Jones et al., 2017; Schliermann et al., 2017; Frank et al., 2019). As a result, well-being has become a key health outcome for medical and service providers supporting people with VI. Previous attempts to define well-being in the context of VI have retraced the evolution of the well-being debate outside of the context of VI (Marques-Brocksopp, 2012), but they have not considered the views of those living with VI and, indeed, if general conceptualizations of well-being are appropriate in this context. Evidence indicates that psychological well-being, for instance, may be poorer when assessed with vision-specific than general measures because participants may report feeling worried about future vision loss (vision-specific measures), but they may not feel worried about the future in general (general measures) (Pinquart and Pfeiffer, 2011). This suggests that a lack of consensus around what well-being means to adults living with VI and how it should be assessed, can impact on the identification of specific support needs and more widely the type of support that is designed and provided to those who need it.

As part of a wider research programme to understand well-being in the context of adult VI, the objective of this scoping review was to gain, in the first instance, an understanding of (1) how, if at all, well-being has been conceptualized in the literature relating to adults living with VI, (2) which tools have been used to assess well-being, and (3) which factors have been found to positively and/or negatively impact well-being in this population. This article primarily presents findings relating to the first question and, specifically, it aims to understand which domains and indicators have been used to address well-being in adults with VI. Presentation of findings relating to factors (research question 3) was not within the scope of this article and will be explored elsewhere.

## Methods

A scoping review was conducted following the guidelines set out in the Preferred Reporting Items for Systematic Reviews and Meta-Analyses extension for Scoping Reviews (PRISMA-ScR) checklist (Tricco et al., 2018).

### Search strategy

Literature searches were performed on 2 June 2021 in EBSCOHost (Medline, CINHL) and Ovid (Embase Classic, Embase, Emcare 1995, Health + Psychosocial, HMIC Health Management Info, APA, PsycArticles, PsycInfo, PsycTests) databases, without date restrictions. In order to make the review manageable, proximity operators were used to restrict the search to articles which include search terms relating to well-being within three words of a version of defin^*^ (e.g., “well-being has been defined,” “wellbeing is defined,” “our definition of well being”) using the following search string:

EBSCOHost: (well-being OR wellbeing OR well+being) w3 defin^*^ AND (vision OR low vision OR vision loss OR reduced vision OR subnormal vision OR diminished vision OR vision impairment OR vision impaired OR vis^*^ impair^*^ OR sight loss OR sight impaired OR blind^*^ OR partially sighted OR purblind OR unsighted).

OVID: (well-being OR wellbeing OR well+being) adj3 defin^*^ AND (vision OR low vision OR vision loss OR reduced vision OR subnormal vision OR diminished vision OR vision impairment OR vision impaired OR vis^*^ impair^*^ OR sight loss OR sight impaired OR blind^*^ OR partially sighted OR purblind OR unsighted).

### Eligibility criteria

As part of a wider project on well-being in adults with visual impairment, the articles were assessed against three research questions: (1) did they define well-being, (2) did they refer to any tools used to assess well-being, and/or (3) did they list any factors which impact well-being in adults aged 18+ living with some form of VI. Definitions of VI varied between articles with some using visual acuity and others self-reported VI. The definition of VI was therefore kept relatively broad to include articles which discussed any form of reduction in visual sensitivity which cannot be corrected by standard eyeglasses or medical treatment.

Articles which did not address any of the three questions were excluded. Articles which answered at least one of these questions were included. As a result, articles may be included which did not provide a definition of well-being. Articles were also excluded if they were not available in English or as a full text. No limitations were set on date of publication. Studies on populations with dual sensory loss or conditions which can result in VI such as diabetes were excluded if findings were not reported for those with VI separately. Studies including samples consisting of young people and adults (e.g., 16–25) were discussed and included if the sample was treated as an adult sample or predominantly consisted of participants aged 18 or over. Abstracts, conference posters, and oral presentations were excluded. A quality appraisal was not conducted as the aim of this article was to identify conceptualizations of well-being rather than assess the impact of interventions or identify the prevalence of certain conditions.

### Review and data charting

Four researchers (CC, FD, LJ, NH) contributed to the abstract and full-text reviews. At both stages, each article was reviewed by one researcher only. Abstract screening was conducted in Covidence systematic review software (Veritas Health Innovation Ltd, Melbourne, Australia; available at www.covidence.org). Ambiguous articles were included in the full text review. The full text of articles was screened against the inclusion criteria. Ambiguous articles were highlighted and reviewed by a second reviewer. Disagreements were resolved by a third researcher (CC). The outcome for each article was marked in an Excel data sheet and on Covidence. Data from included articles were entered into a data extraction table during the full text review and included all type/s of well-being mentioned in the article, indicators of well-being, tools used to assess well-being, and quotes from the text to support the findings. General well-being and well-being, without an adjective, were both coded as “general well-being.” Instances which referred to the well-being of specific groups such as veterans (veteran well-being) or patients (patient well-being) were not coded and treated as general well-being of a group. Indicators were identified by searching the full text for explicit (e.g., “We define well-being as…”) or implicit definitions (e.g., through the tools used to assess well-being). Some articles listed indicators which were subsequently described, e.g., by giving sample items from scales used to assess well-being. The research team discussed how to treat these and decided to code all to capture the breadth of meaning assigned to well-being [e.g., “The index comprises five statements addressing three aspects of the participant's feelings over the previous two weeks: Mood (‘I felt cheerful and in good spirits')...” (Rafaely et al., 2018, p.1231) was coded as mood, feeling cheerful, in good spirits]. Some articles used well-being synonymously with QoL, these were coded as *QoL*, while others defined it as an indicator of QoL, these were coded as *component of QoL* and any additional indicators used to define well-being were extracted. Articles which did not attempt to define well-being were coded as *Not identified*. Cases were coded as *Not clear* if the conceptualization provided was unclear, for instance, where a subscale was used to assess well-being, but it was unclear which one, or where contradictory conceptualizations were provided in the same article [e.g., “Measures of quality of life, specifically social support, well-being, depressive symptoms, and satisfaction with life, have been topics of prior research in the field of visual impairments … The relationship of perceived social support and well-being variables (depression, life satisfaction, and the five sense of well-being factors identified by Rubin et al., 2003)…”, Guerette and Smedema, 2011]. The latter were reviewed by a second researcher to control for researcher bias and a third researcher where there was no consensus.

### Data synthesis and coding

Coding proceeded through several stages. As part of an initial process of familiarization with the data, the indicators extracted for each type of well-being were reviewed against the extracted quotes by two researchers (FD, NH). The results were compared, and discrepancies were discussed by reviewing the original article as a team. Where no consensus was achieved, a third researcher (CC) reviewed the coding. In a further round, the indicators were reviewed and standardized to enable comparison (e.g., “satisfaction with life” was changed to *life satisfaction*, “positive relations with others” was coded as *social relationships*). Separate tables were created for each type of well-being. The list of indicators for each type of well-being was reviewed to identify overarching themes (e.g., *sadness* was coded as Negative affect, and *self-esteem* was coded as Self/identity) resulting in 14 domains (Table 1). Domains may include indicators of the same name. For instance, the domain Mental health includes indicators such as *depression, anxiety* but also *mental health*, which was used in several articles as an indicator to assess or define well-being. Ambiguous indicators and those which would fit into several categories were discussed and allocated to one domain. For instance, *depression* may be coded as Negative affect or Mental health. However, there is overlap between some domains. In order to incorporate theoretical conceptualizations of well-being, Hedonia and Eudaimonia were included as domains. Articles were coded against Hedonia if they used *life satisfaction* and/or an indicator of Mood, and Mood itself contained the domains of Positive affect and Negative affect. Eudaimonia included indicators relating to *self-acceptance, environmental mastery, purpose in life, autonomy, personal growth*, and *social relationships*. The latter was also included in the domain Social functioning. Prevalence was calculated for each indicator and for each domain, based on the number of articles with at least one indicator within the domain out of all relevant articles.

**Table 1:** Overview of domains and indicators included in each domain.

**Domain**	**Example indicators**
Not identified	*Not identified* or *Not clear*
QoL	*QoL* or *Component of QoL*
Hedonia	*Life satisfaction*, Mood (domain)
Mood	*Emotions, affect*, Negative affect (domain), Positive affect (domain)
Positive affect	e.g., *positive emotions, positive affect, happiness, contentment, joy, hope*
Negative affect	e.g., *negative affect or emotions, sadness, anger, frustration, fear, worry*
Eudaimonia	Variations of *autonomy, environmental mastery, personal growth, social relationships, purpose in life*
Mental health	e.g., *depression, anxiety, distress*
Self/identity	Attributes and characteristics relating to the self and identity, e.g., *confidence, self-esteem*
Psychological reaction to disability	e.g., *adaptation, acceptance, attitude to vision loss, coping*
Health	e.g., *health satisfaction, health status, mortality burden, morbidity burden*
Functioning	e.g., *falls, capacity, personal safety, activities of daily living (ADLs), fulfilling responsibilities*
Social functioning	Variations of *social participation, social activity, social relationships*
Environment	Broader life circumstances, e.g., *income, housing conditions*

## Results

**Figure 1:**
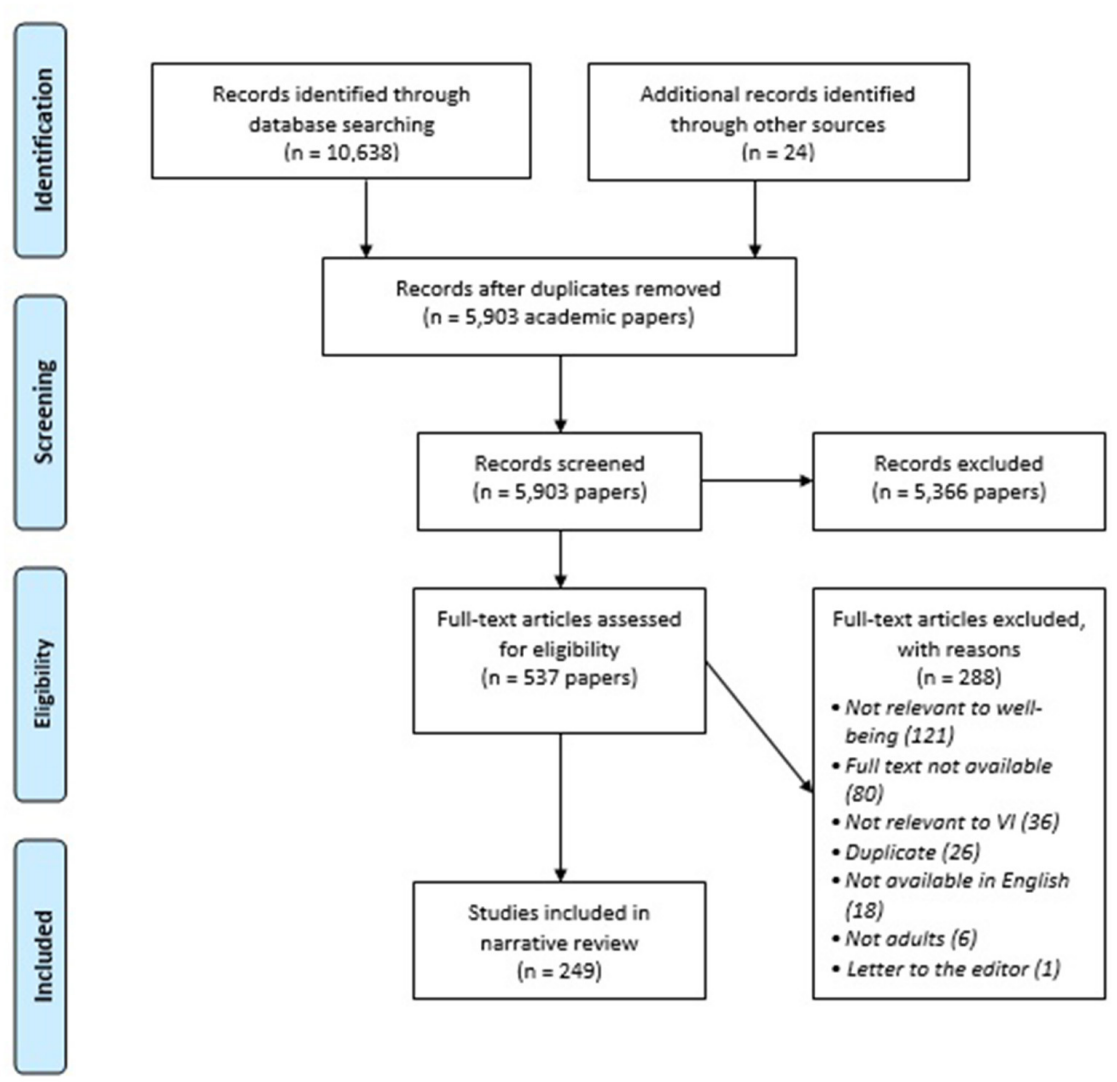
PRISMA 2009 flow diagram.

A total of 10,638 articles were identified in the search and an additional 24 were identified during the full text review. After removing 4,759 duplicates, the title and abstract of 5,903 studies were screened. After excluding 5,366 of these, the full text of 537 articles were reviewed and 288 were excluded (Figure 1).

Data was extracted from a total of 249 unique articles, which referred to 38 different, albeit in some cases related, types of well-being (Table 2). The majority of articles (*n* = 150, 60.2%) referred to one type of well-being only, with a maximum of nine types of well-being mentioned in one article (Marques-Brocksopp, 2014). One article listed eight (Guerette and Smedema, 2011) and four articles listed seven different types of well-being (Smedema and McKenzie, 2010; Mirandola et al., 2019; Godier-McBard et al., 2020; Castle et al., 2021). The most common type of well-being was general well-being (*n* = 101, 40.6%), followed by emotional well-being (*n* = 86, 34.5%) and psychological well-being (*n* = 66, 26.5%).

**Table 2:** Frequency and prevalence of types of well-being.

**Type of well-being**	***n***	**%**
General well-being	101	40.6
Emotional well-being	86	34.5
Psychological well-being	66	26.5
Subjective well-being	38	15.3
Social well-being	29	11.6
Mental well-being	28	11.2
Physical well-being	24	9.6
Psychosocial well-being	18	7.2
Affective well-being	6	2.4
Financial well-being	5	2.0
Personal well-being	4	1.6
Positive well-being	4	1.6
Economic well-being	6	2.4
Holistic well-being	3	1.2
Cognitive well-being	4	1.6
Socio-emotional well-being	3	1.2
Negative well-being	3	1.2
Functional well-being	3	1.2
Spiritual well-being	3	1.2
Physiological well-being	2	0.8
Environmental well-being	2	0.8
Eudaimonic well-being	3	1.2
Medical well-being	2	0.8
Vocational well-being	1	0.4
Health-related well-being	1	0.4
Capability well-being	1	0.4
Visual well-being	1	0.4
Global well-being	1	0.4
Vision-specific well-being	1	0.4
Interpersonal well-being	1	0.4
Individual well-being	1	0.4
Optimal well-being	1	0.4
Socio-ecological well-being	1	0.4
Psychophysical well-being	1	0.4
Vision-related psychological well-being	1	0.4
Existential well-being	1	0.4
Religious well-being	1	0.4
Clinical well-being	1	0.4

**Figure 2:**
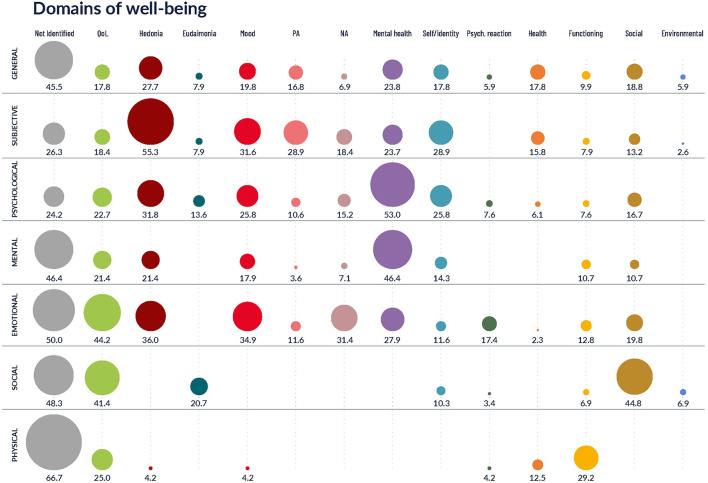
Overview of the prevalence of domains (in %) for the main types of well-being (listed in >20 articles).

The following section provides findings relating to the broader domains and individual indicators used to conceptualize each type of well-being, with related types of well-being presented together. Supplementary Table 1 provides an overview of articles referring to each type of well-being and the indicators and measures extracted for each. Figure 2 provides an overview of the prevalence of domains for the most prevalent types of well-being (*n* > 10).

### General well-being

A total of 101 articles referred to well-being or general well-being. Almost half of these (*n* = 46, 45.5%) did not provide a clear definition of well-being (Supplementary Table 2). The 55 articles which provided a definition used indicators from an average of two domains (*M* = 2.49*, SD* = 2.00) and a maximum of 9 (Deverell et al., 2019). Around a quarter of the articles, respectively, used at least one indicator related to Hedonia (*n* = 28, 27.7%) and Mental health (*n* = 24, 23.8%). Indeed, *life satisfaction* (Hedonia) (*n* = 17, 16.8%) and *depression* (Mental health) (*n* = 18, 17.8%) were the most common indicators. Just under a fifth of articles, respectively, used indicators of Social functioning (*n* = 19, 18.8%) (e.g., *loneliness, social relationships*) and Mood (*n* = 20, 19.8%), which includes Positive affect and Negative affect. Indicators of Positive affect (*n* = 17, 16.8%) (e.g., *happiness, positive affect, enjoyment*) were used more widely than Negative affect (*n* = 7, 6.9%), which included a similarly wide range of negative emotions (e.g., *fear, upset, worry*). In comparison, only eight articles (7.9%) used indicators of Eudaimonia (e.g., *social relationships, self-acceptance, self-realization, autonomy*). Eighteen articles (17.8%), respectively, used indicators relating to Self/identity (e.g., *vitality/energy, morale*) and Health (e.g., *health status, DALYs, mortality burden*). Ten articles (9.9%), respectively, used indicators of Functioning (e.g., *ADLs, falls, feeling safe/assured*). Nine articles, respectively (8.9%) defined well-being as a *component of QoL* or used it synonymously with *QoL*.

In addition to qualitative interviews, clinical/administrative records, self-developed questions, income, mortality and morbidity burden, assessments of general well-being involved 65 distinct assessment tools, most commonly all or some subscales and components of the SF-36 (*n* = 9),versions of the Center for Epidemiologic Studies Depression scale (CES-D) and the Satisfaction with Life scale (SWLS) (*n* = 8, respectively) (Supplementary Table 1). *Life satisfaction* was also assessed using versions of the Cantril's Ladder (*n* = 2), the Life Satisfaction in the Elderly Scale (LSES), Life Satisfaction Index-A (LSI-A) and the Life Satisfaction Index-Well-being (LSI-W) (*n* = 1, respectively). The most common VI-specific scale was the NEI-VFQ-25 (*n* = 8). Twenty-four articles did not identify ways to assess general well-being.

Three articles referred to negative well-being and used indicators of Mental health (*anxiety* and *depression*); Negative affect (*feeling tearful, downhearted/blue, afraid for no reason, upset, panicky*); and a combination of Mental health (*depression*), Health (*health status* and *disability*), and Functioning (*functional ability* and *self-sufficiency*), respectively, to conceptualize it (Supplementary Table 2). Two of four articles which referred to positive well-being did not define it. The remaining two conceptualized it in terms of positive aspects relating to Self/identity (*vitality/energy* and *enthusiasm for life*), and as a combination of indicators of Self/identity (*feeling eager to tackle daily tasks or make new decisions, feeling able to deal with problems or major change in life*) and Hedonia (*happiness, satisfaction with current* and *past life*) (Supplementary Table 2). In addition, three articles referred to holistic well-being. Two of these did not provide a clear definition of holistic well-being and the third defined it as *connection with social, economic, technical, physical and symbolic environments (language, art, literature, and other cultural symbols)*. One article, respectively, referred to global well-being (conceptualized as *life satisfaction* and *depression*) and optimal well-being (not clearly defined).

### Subjective well-being

Subjective well-being was mentioned in 38 articles. The proportion of articles which did not provide a clear definition for subjective well-being was relatively low compared to other types of well-being (*n* = 10, 26.3%) (Supplementary Table 3). The 28 articles which provided a definition used indicators from an average of three domains (*M* = 2.89*, SD* = 2.12) and a maximum of 10 (McManus and Lord, 2012). Similar to general well-being, conceptualizations of subjective well-being used a wide range of indicators, most commonly *life satisfaction* (*n* = 17, 44.7%), *negative* (*n* = 6, 15.8%) and *positive affect/emotions* (*n* = 5, 13.2%), *happiness* (*n* = 5, 13.2%), and *depression* (*n* = 5, 13.2%). Just over half of the articles (*n* = 21, 55.3%) used at least one indicator of Hedonia: in addition to *life satisfaction*, at least one indicator of Mood was present in just over a quarter of articles (*n* = 12, 31.6%). Positive affect (e.g., *positive affect, feeling cheerful, content, full of life, happy, in good spirits, n* = 11, 28.9%) was slightly more common and included a wider range of emotions than Negative affect (*n* = 7, 18.4%) (e.g., *negative affect, feeling bored, sad, upset*). As was the case for general well-being, only 7.9% (*n* = 3) articles used indicators of Eudaimonia (*achievements, environmental mastery, flourishing, goals, purpose in life, self-acceptance, self-realization, and social relationships*) to conceptualize subjective well-being. Over a quarter of articles (*n* = 11, 28.9%) used indicators relating to Self/identity, but there was relatively little consistency, with a wide range of indicators included in this domain (e.g., *concerns, expectations, values, priorities, feelings about the self and future, interest in everyday matters, morale, optimism and pessimism, outlook on life, role difficulties, self-assurance, self-confidence, self-esteem and self-worth, trait anxiety, vitality/energy*). Nine articles (23.7%) used indicators of Mental health (e.g., *depression, distress, stress*), six (15.8%) of Health (e.g., *discomfort, health satisfaction, medical care, sleep*) and five (13.2%) of Social functioning (e.g., *the ability to participate in society, family and social relationships, loneliness*). Conceptualizations which used indicators of Functioning (*the ability to perform expected tasks, dependency* and *leisure time satisfaction*) (*n* = 3, 7.9%) and Environment (*income* and *job satisfaction*) (*n* = 1, 2.6%) were rare.

The review identified 33 measures used to assess subjective well-being (Supplementary Table 1). The most common tool was the Satisfaction with Life scale (SWLS) (*n* = 8), however, life *satisfaction* was also assessed using versions of the Life Satisfaction Index-A (LSI-A) (*n* = 4, respectively), the Life Satisfaction in the Elderly Scale (LSES), Life Satisfaction Index-Well-being (LSI-W) and a single item about overall life satisfaction from the WHO Disability Assessment Schedule (*n* = 1, respectively). Three articles, respectively, listed the Philadelphia Geriatric Center (PGC) Morale Scale and the full or short versions of the Warwick Edinburgh Mental Well-being Scale (WEMWBS). The most common VI-specific scale was again the NEI-VFQ-25 (*n* = 2). Tools were not clearly identified in seven articles.

Four articles referred to personal well-being, two of which did not define it (Supplementary Table 3). The remaining two defined personal well-being as *mood/affect* and *personal safety, personal care* and *leisure activities*. One article listed individual well-being but did not define it.

### Psychological well-being

Supplementary Table 4 provides an overview of the domains and indicators identified for psychological, mental and psychosocial well-being.

A total of 66 articles referred to psychological well-being. Just under a quarter of these (*n* = 16, 24.2%) did not provide a clear definition. The 50 articles which provided a definition used indicators from an average of three domains (*M* = 2.73*, SD* = 2.20) and a maximum of 11 (Pinquart and Pfeiffer, 2011). Over half of the articles used at least one indicator of Mental health (*n* = 35, 53.0%) to conceptualize psychological well-being, most commonly *depression* (*n* = 28, 42.4%), *anxiety* (*n* = 19, 28.8%) and *mental health* itself (*n* = 8, 12.1%). At least one indicator of Hedonia was present in just under a third of articles (*n* = 21, 31.8%). Around a quarter of articles, respectively, used indicators of Mood (*n* = 17, 25.8%), including Positive affect (e.g., *positive affect, relaxation, alertness*) (*n* = 7, 10.6%) and/or Negative affect (e.g., *fear, negative affect, frustration*) (*n* = 10, 15.2%), and Self/identity (*n* = 17, 25.8%) (e.g., *self-esteem, confidence, control, self-efficacy*). Around a fifth of the articles associated psychological well-being with QoL either as a synonym or as a *component of QoL* (*n* = 8, 12.1%, respectively). As observed so far, a much smaller proportion of articles used indicators of Eudaimonia (*n* = 9, 13.6%), however, indicators of Eudaimonia were more prevalent than for all other types of well-being except for social well-being. In addition, individual indicators were used repeatedly including *self-acceptance* and *social relationships* (*n* = 5, 7.6%), *autonomy* (*n* = 4, 6.1%), and *environmental mastery, personal growth* and *purpose in life* (*n* = 3, 4.5%). Eleven articles (16.7%) used indicators of Social functioning (e.g., *social relationships, social isolation, social participation*). Five articles (7.6%), respectively, used indicators of Functioning (e.g., *functioning, reliance on others, perceived security in performing daily occupations*) and Psychological reaction to health problems and disability (e.g., *acceptance of* and *adaptation to disability, coping*) and four (6.1%) of Health (e.g., *problems/symptoms, physical* and *psychological health*). Several articles (*n* = 8, 12.1%) conceptualized psychological well-being as other types of well-being including *emotional, positive, subjective*, and *mental well-being*.

In addition to qualitative interviews, the review identified 59 tools used to assess psychological well-being, the most common being versions of the Center for Epidemiologic Studies Depression Scale (CES-D, *n* = 7) the Geriatric Depression Scale (GDS) and the National Eye Institute Visual Function Questionnaire (NEI-VFQ, *n* = 5, respectively) (Supplementary Table 1).

A total of 28 articles referred to mental well-being and just under half of these (*n* = 13, 46.4%) did not define it. As was the case for psychological well-being, mental well-being was most commonly conceptualized using indicators of Mental health (*n* = 13, 46.4%), most commonly *mental health* itself *(n* = 9, 32.1%), *depression* (*n* = 8, 28.6%), and *anxiety* (*n* = 6, 21.4%). Around a fifth, respectively, viewed mental well-being as either synonymous with *QoL* or a *component of QoL* (*n* = 6, 21.4%) and contained at least one indicator of Hedonia (*n* = 6, 21.4%), mostly Mood (*n* = 5, 17.9%), which included Positive affect (*feeling calm/peaceful*) (*n* = 1, 3.6%) and Negative affect (e.g., *negative affect, anger, shock*) (*n* = 2, 7.1%). None of the articles contained indicators of Eudaimonia. Four articles (14.3%) used indicators of Self/identity (e.g., *self-esteem, identity, able to make up own mind*) and three (10.7%), respectively, used indicators of Functioning (e.g., *functioning, reliance on others, dealing with problems well*) and Social functioning (e.g., *communication, social/leisure activities, social withdrawal*) to conceptualize mental well-being.

A further 18 articles referred to psychosocial well-being. Seven of these (38.9%), respectively, did not define it clearly, defining it as either synonymous with *QoL* (*n* = 4, 22.2%) or a *component of QoL* (*n* = 3, 16.7%). Half of the articles used indicators relating to Mental health (*n* = 9, 50.0%), most commonly *depression* (*n* = 8, 44.4%), *anxiety* and *mental health* itself (*n* = 5, 27.8%, respectively). Indicators of Social functioning (e.g., *social functioning, loneliness, social support*) and Self/identity (e.g., *role disruption, self-esteem, self-worth*) were found in eight articles (44.4%), respectively. Indicators of Hedonia were present in a third of articles (*n* = 6, 33.3%), predominantly Mood (*n* = 5, 27.8%) which includes Positive affect (*happiness*) (*n* = 1, 5.6%) and Negative affect (e.g., *fear, frustration, worry*) (*n* = 5, 27.8%). Two articles (11.1%) included indicators of Eudaimonia (*autonomy* and *interpersonal relationships*). Indicators of Functioning (e.g., *independence, reliance on others*) (*n* = 4, 22.2%), Psychological reaction to health problems (e.g., *adjustment*) (*n* = 2, 11.1%), and Health (*complex visual hallucinations*) (*n* = 1, 5.6%) were also used less frequently to define psychosocial well-being.

An additional article referred to vision-related psychological well-being and conceptualized it using indicators of Psychological reaction to disability and health problems (*acceptance of disability, attitude toward rehabilitation and recognition of limitations* and *remaining capabilities*) and *attitudes toward close relationships*.

### Emotional well-being

Half of the 86 articles which referred to emotional well-being (*n* = 43, 50.0%) did not clearly define it (Supplementary Table 5). The 43 articles which provided a definition used indicators from an average of three domains (*M* = 3.00*, SD* = 2.31) and a maximum of 8 (Misajon et al., 2005; Nyman et al., 2010a,b; Paudel et al., 2015; Assi et al., 2021). Thirty-five (40.7%) conceptualized emotional well-being as a *component of QoL*. Indicators of Hedonia, particularly Mood (*n* = 30, 34.9%), were present in over a third of articles (*n* = 31, 36.0%), while none used indicators of Eudaimonia. Contrary to other types of well-being, indicators of Negative affect (*n* = 27, 31.4%) were not only more prevalent than Positive affect (*n* = 10, 11.6%) (*happiness, hopefulness, positive affect, enjoyment, peacefulness*) but also encompassed a wider range of emotions (e.g., *feeling angry, annoyed, awkward, concerned, despair, embarrassed, fear, like a burden, low, powerless, frustrated, grief, guilty, helpless, hostile, irritated, panicky, regret, upset, vulnerable, worthless*). *Frustration* (*n* = 21, 24.4%) and *sadness* (*n* = 14, 16.3%), alongside *depression* (*n* = 17, 19.8%), were the most commonly used indicators of emotional well-being. This reflects the prevalence of the emotional well-being subscale of the Impact of Impairment (IVI) scale, versions of which were listed in 33 articles including a brief version and versions designed for those with very low vision and those in residential care, suggesting a greater consensus in how emotional well-being should be assessed. Indeed, only 28 measures to assess emotional well-being were identified. In addition to the IVI, the CES-D and NEI-VFQ-25 were used in four articles, respectively (Supplementary Table 1).

Mental health constituted the second most common domain (*n* = 24, 27.9%). Despite the focus on emotions, definitions of emotional well-being also drew on indicators of Social functioning (*n* = 17, 19.8%), (e.g., *loneliness, social isolation*), Psychological reaction to disability (*n* = 15, 17.4%), (e.g., *coping*), Functioning (*n* = 11, 12.8%), (e.g., *health including eyesight interfering with life*), and Self/identity (*n* = 10, 11.6%) (e.g., *confidence*). In addition, indicators of Health (*fatigue* and *symptoms such as headaches, nausea, dizziness, insomnia and appetite loss*) were used in two articles (2.3%).

In addition, six articles referred to affective well-being. Five conceptualized it as *positive* and *negative affect* and one as *depression* and *happiness* (Supplementary Table 5).

### Social well-being

Twenty-nine articles referred to social well-being. Just under half of these (*n* = 14, 48.3%) did not provide a clear definition. The 15 articles which provided a definition used indicators from an average of two domains (*M* = 1.79*, SD* = 1.15) and a maximum of 6 (Fenwick et al., 2012a). Just over a third viewed social well-being as a *component of QoL* (*n* = 10, 34.5%, seven of these did not define it) and two used it synonymously with *QoL* (6.9%). The most common indicators, *social interaction* (*n* = 9, 31.0%), *social activity* (*n* = 4, 13.8%) and *social isolation* (*n* = 4, 13.8%), related to different aspects of Social functioning (*n* = 13, 44.8%). Supplementary Table 6 includes an overview of the subdomains of Social functioning. Indicators of Social participation (e.g., social interaction, social activity, social participation) were present in 41.4% of the articles (*n* = 12). In contrast, indicators of Social relationships (e.g., *interpersonal relationships, the ability to make new* and *maintain existing friendships*) (*n* = 5, 17.2%), Social isolation (*n* = 4, 13.8%) (*social isolation, loneliness, exclusion* and *disengagement from the community*), Intimate relationships (e.g., *relationship status, functioning in* and *satisfaction with the relationship*) (*n* = 2, 6.9%) and Family functioning (e.g., *parental status, functioning, satisfaction with parenting role*) (*n* = 2, 6.9%) were present in less than a fifth of articles, with many indicators used just once. Indicators of Eudaimonia were identified in around a fifth of articles (*n* = 6, 20.7%), predominantly those relating to Social relationships. This is higher than for any other type of well-being. In contrast, none of the articles included indicators of Hedonia. Beyond Social functioning, three articles (10.3%) used indicators of Self/identity (e.g., *altruism, confidence, role disruption*), while two (6.9%) respectively used indicators of Functioning (*dependence* and *fulfilling responsibilities*) and Environment (e.g., *the ability to maintain work, financial strain/loss of income, unequal treatment by others*), and one (3.4%) of Psychological reactions to disability (*concern about treatment by others*). In terms of assessments of social well-being, there was relatively little consistency. The review identified 12 measures but only the Sense of Well-Being Inventory (SWBI) was listed in more than one article and qualitative interviews/focus groups were listed in three (Supplementary Table 1).

In addition, one article referred to interpersonal well-being (not defined) and three to socio-emotional well-being, one of which did not provide a clear definition. One of the remaining two articles conceptualized socio-emotional well-being as Mental health (*socio-emotional distress*) and the other as Negative affect (e.g., *anger, frustration, missing doing the things you used to do*), Self/identity (*confidence, role disruption*), Social functioning (*reduction in social life*) and Functioning (*ability to maintain responsibilities*) (Supplementary Table 6).

### Physical well-being

Two thirds (*n* = 16, 66.7%) of the 24 articles which referred to physical well-being did not provide a clear definition (Supplementary Table 7). The eight articles which provided a definition used indicators from an average of one domain (*M* = 1.42*, SD* = 0.65) and a maximum of 3 (Misajon et al., 2005; Mirandola et al., 2019). A quarter (*n* = 6, 25.0%) conceptualized it as a *component of QoL*, but only two of these provided a further definition of physical well-being. Physical well-being was most commonly conceptualized in terms of Functioning (*n* = 7, 29.2%). Indicators of the subdomain Physical functioning (e.g., *physical functioning, balance, falls, mobility*) were more prevalent (*n* = 7, 29.2%) than for Activity functioning (e.g., *ADLs, physical activity, walking*) (*n* = 3, 12.5%). Three articles (12.5%) also conceptualized well-being in terms of Health (*comorbid health problems* and *physical disabilities, health status, hearing*) and one (4.2%), respectively, included indicators of Self/identity (*coping*) and Mood (*mood*) in their definition of physical well-being. Of the 12 measures identified in this review, only the Activity-Specific Balance Confidence (ABC) was listed in more than one article (*n* = 2). More commonly, assessments of physical well-being involved qualitative interviews (*n* = 3) and the Timed Up and Go test (TUG) (*n* = 2) (Supplementary Table 1).

Two articles referred to physiological well-being. Both conceptualized it as Physical functioning (*balance, falls, grip strength, vibration sense*) and one also as Health (*hearing, lung function, vision*) (Supplementary Table 7). Psychophysical well-being, conceptualized as *QoL*, psychological well-being (*autonomy, environmental mastery, personal growth, positive relations with others, purpose in life*, and *self-acceptance*) and physical well-being (*functioning* and *health status*), was mentioned in one article.

### Other types of well-being

Indicators for the other types of well-being identified in this review are reported in Supplementary Table 8. For instance, three articles referred to spiritual well-being, one of which did not provide a clear definition. The other two articles defined it in terms of inter- and intrapersonal connection, interpersonal meaning and intrapersonal wellness (*n* = 1, 33.3%, respectively). Both articles also defined it as a relationship to a higher power or transpersonal connection. Finally, one article referred to religious and existential well-being, which were defined as relationship and closeness with a higher power, and intrapersonal wellness, purpose, sense of meaning, and fulfillment, respectively.

## Discussion

This scoping review set out to provide an overview of how well-being has been conceptualized in research relating to adults living with VI. This research forms part of a wider project aimed at developing a working definition of well-being which can be applied to future research and practice within the sight loss sector. The wealth of articles identified in the search supports the relative importance of well-being in the context of VI. And yet, as reported elsewhere (Pollard and Lee, 2003; Linton et al., 2016), the findings suggest there remains a lack of consensus on how well-being should be conceptualized and assessed. Firstly, while the appearance of multiple types of well-being in one article is not necessarily surprising considering that multi-dimensional conceptualizations of well-being have comprised specific types such as social or physical well-being (Linton et al., 2016), this review identified 38 different types of well-being listed in the literature. In some cases, these were conceptually related and/or used synonymously, such as physical and physiological health, and 15 appeared in only one article (39.5%) and 30 in fewer than 10 (78.9%). Marques-Brocksopp (2014) referred to a maximum of nine types of well-being (general, emotional, social, holistic, physical, spiritual, environmental, socio-ecological, and eudaimonic well-being).

Secondly, a large proportion of articles did not define well-being. A third of the articles which referred to physical well-being (66.7%) and around half of the articles which referred to general, mental, emotional or social well-being (45.5–50.0%) did not provide a clear definition. This supports previous evidence that clear and complete definitions of well-being are often missing, even from literature reporting on the development of well-being scales (Linton et al., 2016). Missing definitions may reflect the absence of a theoretical framework of well-being, uncertainty about the meaning of well-being or a presumption of a shared understanding of what is meant by well-being. The risk of leaving a notoriously fuzzy concept, such as well-being, undefined is that readers will apply their own understanding of it, which may vary from that of the authors. As reported elsewhere (Pollard and Lee, 2003; Cooke et al., 2016; Linton et al., 2016), this review found considerable variation in the way well-being has been conceptualized. For instance, 137 unique indicators were extracted for general well-being; 86 of these (62.8%) were used only once and the two most frequent indicators, life satisfaction and depression, were used in just a third of articles which provided a definition (31.5%). Similarly, 25 indicators referred to characteristics or attributes related to self and identity, however, among the 18 articles which used indicators of Self/identity to conceptualize general well-being, the two most common indicators, vitality/energy and morale, were used in only 5 and 3 articles, respectively. Although, some types of well-being may be more consistent: of the 86 indicators identified for emotional well-being, just over half (*n* = 45, 52.9%) were used only once and the most common indicator frustration was used in just under half (48.8%) of the 43 articles which provided a definition. This may be due to the prevalence of studies using the Impact of Vision Impairment (IVI) tool which includes an emotional well-being subscale. This shows the myriad ways in which well-being may be understood and highlights the importance of providing a definition to ensure a shared understanding.

A further issue revolves around whether what is being measured is always well-being, particularly where only one indicator is used. For instance, Allen et al. (1999) used self-reported health status as the only indicator of general well-being (“It [EuroQoL] also includes a visual analog scale (VAS) that allows respondents to report their valuation of their overall health status… A self-assessed VAS rating of current health status (general well-being) was recorded on a vertical, ruler-type line on which the best and worst imaginable health scores ranged from 100 to 0, respectively”, p. 1136), while Brenner et al. (1993) and Bergeron and Wanet-Defalque (2013) used life satisfaction. In isolation, both health status and life satisfaction arguably provide an insight into aspects of well-being, but they do not in themselves provide an assessment of well-being as a whole.

The debate around well-being is not new and models of well-being such as those relating to hedonia (SWB, Diener, 1984) and eudaimonia (PWB, Ryff, 1989) are relatively well established, both outside of and within the context of VI (McDowell, 2010; Marques-Brocksopp, 2012). While individual indicators of hedonia were relatively common, conceptualizations which included all indicators of hedonia were not. Greater consistency might be expected for subjective well-being, considering the existence of a well-established model of subjective well-being. Indeed, three quarters (*n* = 21, 75.0%) of the 28 articles which provided a definition for subjective well-being used at least one of the indicators relating to hedonia. However, only eight of the 21 (38.1%) used both, life satisfaction and Mood. Comparatively few articles used at least one of the indicators relating to eudaimonia (between 20.7% for social well-being and 7.9% for general and subjective well-being) and even fewer used all to define well-being. Only a third (*n* = 3, 33.3%) of the nine articles which used indicators of Eudaimonia to define psychological well-being used all indicators included in Ryff's (1989) model of PWB.

The lack of consensus around how well-being is conceptualized has direct implications for how it is assessed and, therefore, how comparable findings are across different studies. It has further practical implications with a lack of shared understanding potentially impacting on the support provided to adults living with VI, particularly across different service providers. Although this may not be applicable to all settings, a standardized approach to conceptualizing and assessing well-being in adults living with VI would enable comparison across studies, replicability, longitudinal assessments of well-being and collaboration between different organizations involved in supporting adults living with VI. Previous work has been reluctant to recommend specific measures, particularly a single measure, to assess well-being (Linton et al., 2016; Lambert et al., 2019). However, there is general agreement that any assessment should take a multi-dimensional approach. Although applied to the context of national well-being, Lambert et al. (2019) recommend the use of multiple measures assessing aspects of hedonia, eudaimonia, culture and religiosity, mental health, physical activity, contact with nature/green spaces, and experience of the immediate environment and respondents' wider life, to derive a complete picture of well-being. A standardized multi-domain approach to conceptualizing and assessing well-being may be usefully applied to the context of adult VI, particularly in health and social care settings. Such an approach may identify areas of vulnerability and strength at one timepoint, providing useful insights for support services, and monitor changes across time.

However, there are several considerations when developing a standardized approach. First, there is a need to establish what is being measured when selecting domains, indicators and tools. For instance, Lambert et al. (2019) recommend including a measure of physical activity and contact with nature. However, this does not distinguish between indicators (“states”) of well-being and factors (“determinants”) which may impact it (Linton et al., 2016). High levels of physical activity or time spent in green spaces in themselves do not equate to well-being but rather are factors which may impact well-being. While factors may be usefully included in assessments of well-being to guide possible interventions, a careful distinction must be made between what is an indicator of well-being and what is a factor to avoid further blurring the understanding of well-being.

Second, Lambert et al. (2019) recommend deriving domains from existing theoretical frameworks. However, this may fail to represent the specific experiences of adults with VI in a conceptualization of well-being. Considering the importance and value of patient involvement (Dean et al., 2017), any standardized approach to conceptualizing well-being would benefit from the input of adults with VI, and those providing support to them, to ensure it is relevant and appropriate. Involving adults with VI in the process of agreeing on a standardized approach to conceptualizing and, ultimately, assessing well-being would further mitigate issues of respondent burden arising from a multi-domain approach which uses multiple measures to assess well-being. The target population of adults with VI are best placed to take decisions on appropriate length and questions for a well-being assessment tool, to avoid instances of missing data and drop-out, particularly where assessments are longitudinal. Several articles used interviews and focus groups with adults with VI to develop new QoL measures (Misajon et al., 2005; Khadka et al., 2015; Paudel et al., 2015). While these tend to incorporate specific types of well-being, e.g., emotional well-being subscales, it is unclear if participants referred to and described what they meant by emotional well-being, or if authors coded participants' experiences as such.

Comparison between the domains identified in this review and the work of Linton et al. (2016) shows considerable agreement, although domain labels may vary. The domain of mental well-being arguably corresponds to the domains of Mental health and Hedonia (Mood and *life satisfaction*), social well-being to Social functioning, physical well-being to Health, activities and functioning to Functioning, and personal circumstances to Environment. Dimensions in the domain spiritual well-being, however, tend to be coded as indicators of Self/identity in this review. Future research will need to work with adults with VI to review the domains, indicators and measures identified in the current and previous reviews and agree on a set which they feel are appropriate to assess their well-being. Finally, there may be cross-cultural and contextual differences which impact on the appropriateness of a standardized multi-method approach. Once indicators have been identified, the model will need to be tested to ensure its applicability in different cultures, populations, and contexts.

### Limitations

There are a number of limitations to consider when reviewing the findings. The scoping review aimed to address three distinct research questions and all articles were assessed against these in the abstract and full text review stages. Articles were included if they addressed at least one of the research questions, however, articles which did not define well-being (research question 1) nor provided any tools to assess it (research question 2) nor factors which impact on it (research question 3), were excluded. As a result, the proportion of articles which did not identify well-being is likely to be higher.

There is an inherently subjective element involved in coding. As a result, the indicators and domains reported in this article may have been interpreted differently by other researchers and may not reflect the authors' intended conceptualization of well-being. For instance, McManus and Lord (2012) conceptualized mental well-being as mental health and provided the items from the Warwick-Edinburgh Mental Well-being Scale (WEMWBS) used to assess it. However, in our coding frame, items such as *Able to think clearly* or *Able to make up own mind* were coded as self/identity. In order to control for researcher bias, articles which provided a definition for an indicator itself, as in the example of McManus and Lord (2012), or were ambiguous, particularly those coded as Not clear, were reviewed by a second reviewer and/or third reviewer if there was no consensus. To enable comparison, some indicators were not coded verbatim (e.g., positive relations with others was coded as social relationships). However, the authors' own terminology was retained as much as possible. This may have inflated the number of indicators found because indicators which represented opposites of each other could have arguably been combined into one code, e.g., *Independence* and *Reliance on others* are arguably opposites of the same concept. In addition, all indicators were coded including any sample items for scales used to assess an indicator. This also highlights that indicators are missing where only some scale items are provided or authors list example indicators but not a full definition of their understanding of well-being [e.g., For “*Although adjustment to vision loss is a component of general well-being, it is not synonymous with adaptation to aging*” (Horowitz and Reinhardt, 1998, p.33). General well-being is coded as *adjustment to vision loss*, however this is listed as just one indicator, not a complete definition of general well being].

Finally, this study did not differentiate between people with congenital and acquired VI, nor between different levels of severity, due to the lack of differentiation and a standard approach to categorizing VI in the literature. However, these differences may be important for our understanding of well-being and should form part of future research.

## Conclusions

This scoping review identified a wealth of research which refers to well-being in adults with VI. However, there remains a lack of consensus on how well-being is conceptualized and assessed, if it is indeed defined at all. A standardized approach which addresses well-being holistically is required to ensure findings are comparable across studies and time, and provide practical insights for practitioners working with adults with VI. This approach should be developed in collaboration with adults living with VI, and practitioners supporting them, to ensure it is relevant and appropriate.

## Data availability statement

The original contributions presented in the study are included in the article/Supplementary material, further inquiries can be directed to the corresponding author/s.

## Author contributions

NH designed the research, performed the review and analysis, and wrote the paper. FD performed the review and analysis and wrote the paper. LJ and CC performed the review and edited the paper. RG designed the research and edited the paper. All authors contributed to the article and approved the submitted version.

## Funding

This work was supported by the Thomas Pocklington Trust, grant number TP-211.

## Conflict of interest

The authors declare that the research was conducted in the absence of any commercial or financial relationships that could be construed as a potential conflict of interest.

## Publisher's note

All claims expressed in this article are solely those of the authors and do not necessarily represent those of their affiliated organizations, or those of the publisher, the editors and the reviewers. Any product that may be evaluated in this article, or claim that may be made by its manufacturer, is not guaranteed or endorsed by the publisher.

## Abbreviations

PWB: Psychological well-being
SWB: Subjective well-being
VI: Visual impairment.

## References

[B1] Allen E. D. Wood C. M. Currie S. Jayamanne D. G. R. (1999). Correlation between early, measurable improvement in quality of life and speed of visual rehabilitation after phacoemulsification. J. Cataract Refract. Surg. 25, 1135–1139. 10.1016/S0886-3350(99)00138-8 10445201

[B2] Assi L. Chamseddine F. Ibrahim P. Sabbagh H. Rosman L. Congdon N. . (2021). A global assessment of eye health and quality of life: a systematic review of systematic reviews. JAMA Ophthalmol. 139, 526–541. 10.1001/jamaophthalmol.2021.0146 33576772 PMC7881366

[B3] Bergeron C. M. Wanet-Defalque M.-C. (2013). Psychological adaptation to visual impairment: the traditional grief process revised. Br. J. Vis. Impair. 31, 20–31. 10.1177/0264619612469371

[B4] Brenner M. H. Curbow B. Javitt J. C. Legro M. W. Sommer A. (1993). Vision change and quality of life in the elderly: response to cataract surgery and treatment of other chronic ocular conditions. Arch. Ophthalmol. 111, 680–685. 8489453 10.1001/archopht.1993.01090050114040

[B5] Castle C. L. Engward H. Kersey T. (2021). Arts activity and well-being for visually impaired military veterans: a narrative discussion of current knowledge. Public Health 194, 232–237. 10.1016/j.puhe.2021.03.010 33962101

[B6] Cooke P. J. Melchert T. P. Connor K. (2016). Measuring well-being: a review of instruments. Couns. Psychol. 44, 730–757. 10.1177/0011000016633507

[B7] Dean S. Mathers J. M. Calvert M. Kyte D. G. Conroy D. Folkard A. . (2017). “The patient is speaking”: discovering the patient voice in ophthalmology. Br. J. Ophthalmol. 101, 700–708. 10.1136/bjophthalmol-2016-309955 28455280 PMC5583687

[B8] Deverell L. Bradley J. Foote P. Bowden M. Meyer D. (2019). Measuring the benefits of guide dog mobility with the Orientation and Mobility Outcomes (OMO) tool. Anthrozoos 32, 741–755. 10.1080/08927936.2019.1673036

[B9] Diener E. (1984). Subjective well-being. Psychol. Bull. 95, 542–575. 10.1037/0033-2909.95.3.542 6399758

[B10] Diener E. Suh E. (1997). Measuring quality of life: economic, social, and subjective indicators. Soc. Indic. Res. 40, 189–216. 10.1023/A:1006859511756

[B11] Dodge R. Daly A. P. Huyton J. Sanders L. D. (2012). The challenge of defining wellbeing. Int. J. Wellbeing 2, 222–235. 10.5502/ijw.v2i3.4

[B12] Fenwick E. Rees G. Pesudovs K. Dirani M. Kawasaki R. Wong T. Y. . (2012a). Social and emotional impact of diabetic retinopathy: a review. Clin. Exp. Ophthalmol. 40, 27–38. 10.1111/j.1442-9071.2011.02599.x 21575125

[B13] Fenwick E. K. Pesudovs K. Khadka J. Dirani M. Rees G. Wong T. Y. . (2012b). The impact of diabetic retinopathy on quality of life: qualitative findings from an item bank development project. Qual. Life Res. 21, 1771–1782. 10.1007/s11136-012-0110-1 22290480

[B14] Frank C. R. Xiang X. Stagg B. C. Ehrlich J. R. (2019). Longitudinal associations of self-reported vision impairment with symptoms of anxiety and depression among older adults in the United States. JAMA Ophthalmol. 137, 793–800. 10.1001/jamaophthalmol.2019.1085 31095253 PMC6537761

[B15] Garcia G. A. Khoshnevis M. Gale J. Frousiakis S. E. Hwang T. J. Poincenot L. . (2017). Profound vision loss impairs psychological well-being in young and middle-aged individuals. Clin. Ophthalmol. 11, 417. 10.2147/OPTH.S113414 28260855 PMC5328297

[B16] Godier-McBard L. R. Castle C. L. Heinze N. Hussain S. F. Borowski S. Vogt D. S. . (2020). A preliminary investigation of the well-being of visually impaired ex-service personnel in the United Kingdom. Br. J. Vis. Impair. 40, 274–288. 10.1177/0264619620973683

[B17] Guerette A. R. Smedema S. M. (2011). The relationship of perceived social support with well-being in adults with visual impairments. J. Vis. Impair. Blind. 105, 425–439. 10.1177/0145482X1110500705

[B18] Horowitz A. Reinhardt J.P. (1998). Development of the adaptation to age-related vision loss scale. J. Visual. Impair Blindness. 92, 30–41.30608511

[B19] Jones L. Bryan S. R. Crabb D. P. (2017). Gradually then suddenly? Decline in vision-related quality of life as glaucoma worsens. J. Ophthalmol. 2017:1621640. 10.1155/2017/1621640 28469940 PMC5392404

[B20] Kempen G. I. J. M. Ballemans J. Ranchor A. V. van Rens G. H. M. B. Zijlstra G. A. R. (2012). The impact of low vision on activities of daily living, symptoms of depression, feelings of anxiety and social support in community-living older adults seeking vision rehabilitation services. Qual. Life Res. 21, 1405–1411. 10.1007/s11136-011-0061-y 22090173 PMC3438403

[B21] Keyes C. L. (2002). The mental health continuum: from languishing to flourishing in life. J. Health Soc. Behav. 43, 207–222. 10.2307/3090197 12096700

[B22] Khadka J. McAlinden C. Craig J. E. Fenwick E. K. Lamoureux E. L. Pesudovs K. (2015). Identifying content for the glaucoma-specific item bank to measure quality-of-life parameters. J. Glaucoma 24, 12–19. 10.1097/IJG.0b013e318287ac11 23552836

[B23] Lambert L. Hotchkiss L. Passmore H.-A. (2019). “Measuring wellbeing: how and why?,” in Positive Psychology in the Middle East/North Africa, eds L. Lambert, and N. Pasha-Zaidi (Cham: Springer), 207–234.

[B24] Lent R. W. (2004). Toward a unifying theoretical and practical perspective on well-being and psychosocial adjustment. J. Couns. Psychol. 51, 482. 10.1037/0022-0167.51.4.482

[B25] Linton M.-J. Dieppe P. Medina-Lara A. (2016). Review of 99 self-report measures for assessing well-being in adults: exploring dimensions of well-being and developments over time. BMJ Open 6, e010641. 10.1136/bmjopen-2015-010641 27388349 PMC4947747

[B26] Marques-Brocksopp L. (2012). The broad reach of the wellbeing debate: emotional wellbeing and vision loss. Br. J. Vis. Impair. 30, 50–55. 10.1177/0264619611428244

[B27] Marques-Brocksopp L. (2014). Mindfulness, spiritual well-being, and visual impairment: an exploratory study. Br. J. Vis. Impair. 32, 108–123. 10.1177/0264619614528343

[B28] McDowell I. (2010). Measures of self-perceived well-being. J. Psychosom. Res. 69, 69–79. 10.1016/j.jpsychores.2009.07.002 20630265

[B29] McManus S. Lord C. (2012). Circumstances of People With Sight Loss: Secondary Analysis of Understanding Society and the Life Opportunities Survey. Natcen Report and RNIB.

[B30] Mirandola D. Monaci M. Vannuzzi A. Manetti M. Marini M. Miccinesi G. . (2019). Psychological well-being and quality of life in visually impaired baseball players: an Italian national survey. PLoS ONE 14, e0218124. 10.1371/journal.pone.0218124 31170226 PMC6553783

[B31] Misajon R. Hawthorne G. Richardson J. Barton J. Peacock S. Iezzi A. . (2005). Vision and quality of life: the development of a utility measure. Investig. Ophthalmol. Vis. Sci. 46, 4007–4015. 10.1167/iovs.04-1389 16249474

[B32] Nyman S. R. Gosney M. A. Victor C. R. (2010a). Emotional well-being in people with sight loss: lessons from the grey literature. Br. J. Vis. Impair. 28, 175–203. 10.1177/0264619610374171

[B33] Nyman S. R. Gosney M. A. Victor C. R. (2010b). Psychosocial impact of visual impairment in working-age adults. Br. J. Ophthalmol. 94, 1427–1431. 10.1136/bjo.2009.164814 19850584

[B34] Paudel P. Burnett A. Naduvilath T. Fricke T. R. Khadka J. Hani Y. (2015). Papua New Guinea vision-specific quality of life questionnaire: a new patient-reported outcome instrument to assess the impact of impaired vision. Clin. Exp. Ophthalmol. 43, 202–213. 10.1111/ceo.12413 25132289

[B35] Pezzullo L. Streatfeild J. Simkiss P. Shickle D. (2018). The economic impact of sight loss and blindness in the UK adult population. BMC Health Serv. Res. 18, 1–13. 10.1186/s12913-018-2836-0 29382329 PMC5791217

[B36] Pinquart M. Pfeiffer J. P. (2011). Psychological well-being in visually impaired and unimpaired individuals: a meta-analysis. Br. J. Vis. Impair. 29, 27–45. 10.1177/0264619610389572

[B37] Pollard E. L. Lee P. D. (2003). Child well-being: a systematic review of the literature. Soc. Indic. Res. 61, 59–78. 10.1023/A:1021284215801

[B38] Rafaely L. Carmel S. Bachner Y.G. (2018). Subjective well-being of visually impaired older adults living in the community. Aging Ment Health. 22, 1223–1231. 10.1080/13607863.2017.1341469 28636409

[B39] Rubin S. E. Chan F. Bishop M. Miller S. M. (2003). Psychometric validation of The Sense of Well-Being Inventory for programme evaluation in rehabilitation. Prof. Rehabil. 11, 54–59.

[B40] Ryan R. M. Deci E. L. (2001). On happiness and human potentials: a review of research on hedonic and eudaimonic well-being. Annu. Rev. Psychol. 52, 141–166. 10.1146/annurev.psych.52.1.141 11148302

[B41] Ryff C. D. (1989). Happiness is everything, or is it? Explorations on the meaning of psychological well-being. J. Pers. Soc. Psychol. 57, 1069. 10.1037/0022-3514.57.6.1069

[B42] Schliermann R. Heydenreich P. Bungter T. Anneken V. (2017). Health-related quality of life in working-age adults with visual impairments in Germany. Disabil. Rehabil. 39, 428–437. 10.3109/09638288.2016.1146353 26937707

[B43] Seligman M. E. (2012). Flourish: A Visionary New Understanding of Happiness and Well-Being. New York, NY: Simon and Schuster.

[B44] Smedema S. M. McKenzie A. R. (2010). The relationship among frequency and type of internet use, perceived social support, and sense of well-being in individuals with visual impairments. Disabil. Rehabil. 32, 317–325. 10.3109/09638280903095908 20055570

[B45] Tricco A. C. Lillie E. Zarin W. O'Brien K. K. Colquhoun H. Levac D. . (2018). PRISMA extension for scoping reviews (PRISMA-ScR): checklist and explanation. Ann. Intern. Med. 169, 467–473. 10.7326/M18-0850 30178033

[B46] van der Aa H. P. Comijs H. C. Penninx B. W. van Rens G. H. van Nispen R. M. (2015). Major depressive and anxiety disorders in visually impaired older adults. Invest. Ophthalmol. Vis. Sci. 56, 849–854. 10.1167/iovs.14-15848 25604690

[B47] Zhang X. Bullard K. M. Cotch M. F. Wilson M. R. Rovner B. W. McGwin G. . (2013). Association between depression and functional vision loss in persons 20 years of age or older in the United States, NHANES 2005-2008. JAMA Ophthalmol. 131, 573–581. 10.1001/jamaophthalmol.2013.2597 23471505 PMC3772677

