# Supplementary material for: Conceptualizations of well-being in adults with visual impairment: A scoping review
Source: Front Psychol. 2022 Sep 26;13:964537. doi: 10.3389/fpsyg.2022.964537 (PMC9549791; doi:10.3389/fpsyg.2022.964537)
Supplement: Supplementary file 1 [file Table_1.doc]

Supplementary Table - Overview of indicators and measures for each type of well-being

| General well-being | | | |
| --- | --- | --- | --- |
| Study | **Indicator** | **Measure** | **Quote** |
| Richards et al. (2010) | Not identified | Not identified | *Similarly the United Nations 2006 Treaty on Rights for the Disabled draws specific attention to the role of cultural activities in promoting citizenship, well-being and life quality… Whilst several individuals spoke of their enjoyment of tourism—encountering new experiences, cultures and people—their experiences also confirm for them that the tourism industry (like others) tends to collectively problematize and marginalize them, at times subjecting them to standardized responses which undermine their confidence and well-being and strip them of dignity and respect.* |
| Patty et al. (2018) | Attachment  Security  Role  Enjoyment  Control | ICECAP-O | *The ICECAP-O consists of five attributes (attachment, security, role, enjoyment and control), one question per dimension and four answering categories per question. The ICECAP-O measures ‘years of full capability’, based on attributes of well-being that have been found to be important for the elderly... Therefore, the explicit decision was made to use the ICECAP-O, as it is specifically aimed at measuring the well-being among elderly* |
| Assi et al. (2021) | Not identified | Qualitative interviews | *Qualitative studies provided insight into the mechanisms of the associations, specifically on well-being and functioning… The review process highlighted the need for a unified definition for quality of life to study and understand the association with vision impairment and ophthalmic interventions on well-being and vision function from the patient perspective… Many systematic reviews were excluded because they considered patient satisfaction or patient-reported symptoms and discomfort as quality-of-life measures. While these measures fall under the umbrella of patient-reported outcomes and capture valuable information, they do not describe general well-being or vision function in day-to-day life* |
| Luu et al. (2020) | Component of the psychological dimension of QoL  Not identified | VROOM  OMO | *However, recently Deverell et al.44 validated two new measures of functional performance in orientation and mobility settings, namely the vision-related outcomes in orientation and mobility (VROOM) and orientation and mobility outcomes (OMO) tools… The scores are calculated via a combination of observed behaviours (out of 30) and self-reported wellbeing (out of 20) with an aggregate score of 50 for vision and 50 for mobility… Aaronson39,55 defined four conceptual dimensions of quality of life, namely: physical (disease symptoms and their treatment), function (self-care, mobility, activity level and ADL), social (social contact and interpersonal relationships) and psychological (cognitive function, emotional status, wellbeing, satisfaction and happiness).* |
| Salminen et al. (2019) | Not identified | Not identified | *However, little is known about interventions that focus on promoting their participation that contributes to health and well-being and is considered the most relevant outcome in rehabilitation… Participation contributes to health and well-being, and in rehabilitation, participation is considered as the most relevant outcome… Difficulties in activities and participation, as well as restricted environmental factors of young people with visual impairment should be recognized in all health care, social care and educational settings to promote full participation and wellbeing of the young.* |
| Godier-McBard et al. (2020) | Life satisfaction  Positive affect  Positive relations  Broader life circumstances | Not identified | *A review of literature found 42 instruments designed to measure some aspect of well-being (…), with huge variation in their length, their conceptualisation of well-being, and the constructs measured. The majority of the instruments measured constructs such as life satisfaction, positive affect, and positive relations, but did not consider broader life circumstances that are reflective of, and impact, well-being (…). In response to the lack of a standardised measure of holistic well-being that can be used with military veterans, Vogt et al. (2019) developed the Well-Being Inventory (WBI), which has been rigorously validated in six phases with multiple cohorts of US military veterans (…). This set of measures assesses individuals’ status, functioning, and satisfaction with regard to key aspects of their life (referred to as ‘domains’), including their health, vocation, finances, and social relationships... Through its consideration of status, functioning, and satisfaction across these key life domains, the WBI provides a comprehensive assessment of factors that set the stage for well-being, which facilitates the ability to pinpoint areas of both strength and vulnerability within the overall concept of well-being* |
| Bell and Foley (2021) | Not clear | Not identified | *It cautions against the growing drive to identify and prescribe a generalised ‘healthy dose’ of nature of specific duration, frequency and type (…); a drive that implicitly frames wellbeing as an almost predictable ‘entity that can be acquired, accumulated and retained’ (…), placing responsibility for attaining it on in the apparently self-sufficient, autonomous and independent individual. Instead, our participant narratives highlight wellbeing as an emergent quality of shifting relationships – with human/nonhuman others as well memories, imaginaries and societal norms and discourses* |
| Wittich et al. (2014) | Sensory impairment (visual and hearing functioning)  Psychological well-being (Depression, social isolation)  Physical well-being (balance, falls)  Cognitive status | ABC  TUG  VF-14  Hearing Handicap Inventory for the Elderly  GDS  Friendship Scale  Montreal Cognitive Assessment (MoCA)  LSQ | *Between September 2011 and October 2012, 30 newly referred clients (age = 71–98 years, M = 85, visual acuity [VA] 20/50 to no-light-perception [NLP], M = 20/126) were evaluated at intake, and after 6 and 12 months, including the Visual Function Questionnaire-14, Hearing Handicap Inventory for the Elderly, Geriatric Depression Scale, Friendship Scale, Timed Up and Go Test, and Montreal Cognitive Assessment (MoCA)… Participants reported statistically unchanged scores on all the measures, except for improved MoCA scores… Considering the vulnerability of this population, the data indicate that the Day Centre contributes to prevent decline in its clients’ general well-being.* |
| Vahabi et al. (2014) | QoL | SF-36 (Persian version) | *Quality of life evaluates the general well-being of individuals and it can be considered as one of the important aspects in programming and giving service to disabled people… In this cross-sectional study, data from 71 blind war (Iran-Iraq) veterans in 2010 were collected using the Short Form Health Survey instrument (SF36)… QoL evaluates the general well-being of individuals and societies.* |
| Castle et al. (2021) | Health  Happiness | Not identified | *‘Well-being’ refers to a holistic concept of health and happiness that is impacted by both physical abilities (e.g. one's ability to attend and perform well at work) and subjective appraisal of one's physical, cognitive and/or affective state.3,4 As Vogt et al. (2018) propose in their work with US veterans, well-being is multidimensional, influenced by both life functioning and satisfaction. This holistic approach considers multiple factors as contributing to a person's health and well-being, just as health and well-being may impact on objective life outcomes* |
| Breheny et al. (2020) | Not identified | Preference-based measures | *Furthermore, PBMs measure aspects of health and wellbeing unrelated to the condition. They are therefore not intended to be strongly associated with condition specific measures or clinical outcomes… Developing measures that are sufficiently broad to measure health-related wellbeing in all common conditions, without the need for bolt-ons should be prioritised* |
| Liljas et al. (2020) | QoL:  Control  Autonomy  Self-realization  Pleasure in life | CASP-19 | *OBJECTIVE To examine associations between concurrent multisensory impairments and aspects of well-being and mental health, namely quality of life and depressive symptoms… Quality of life refers to multiple aspects of functioning, including perceived sense of control, autonomy, self-realization, and pleasure in life.27Research has shown that maintaining mental health and well-being is important to remain independent in later life… Quality of life was assessed in the self-completion questionnaire using the validated 19-item CASP-19 scale (Control, Autonomy, Self-realization and Pleasure), which conceptualizes self-perceived quality of life as the extent to which needs are satisfied, eg, being able to do things that you want to do.3* |
| Wettstein et al. (2021) | Life satisfaction  Positive affect  Negative affect  Depression  Loneliness | SWLS  PANAS  15-item CES-D (German adaptation)  De Jong Gierveld Loneliness Scale (modified version) | *We analyzed how self-reported vision problems as a time-varying predictor are related to long-term changes in health and cognitive ability (functional health, number of chronic diseases, self-rated health, information processing speed), well-being (life satisfaction, positive and negative affect, depressive symptoms, loneliness), and subjective age views (subjective age; aging-related cognitions: social loss, physical decline, continuous growth)… Well-being. Given the multidimensionality of well-being (…), multiple indicators were included. Life satisfaction was measured based on the Satisfaction with Life Scale (SWLS; …) comprising five items... Positive and negative affect were assessed by the Positive and Negative Affect Schedule (PANAS;... Depressive symptoms were assessed based on a 15-item German adaptation (…) of the Center for Epidemiological Studies Depression Scale... Finally, loneliness was assessed by a modified version of the De Jong Gierveld Loneliness Scale* |
| Brunes et al. (2018) | Life satisfaction  Self-efficacy | General Self-Efficacy Scale (GSE, Norwegian version)  Cantril’s Ladder (Norwegian version) | *Self-efficacy: The participants’ general perception of self-efficacy was measured by the General Self Efficacy Scale (GSE scale)… Life satisfaction: Cantril’s Ladder of Life Satisfaction (CLLS) was used to measure current life satisfaction… To our knowledge, this is the first study with a nation-wide probability sampling addressing the prevalence and associated factors of bullying among individuals with VI, as well as the relation of bullying with well-being… First, models on stress and coping suggest that prolonged exposure to a given stressor, such as bullying, leads to a sustained cognitive activation[38]. Problems handling this unbearable state of mind may reduce one’s belief in coping with challenging situations, and the persistent activation could be subsequently manifested as reductions in well-being and unwanted behavioural reactions like social withdrawal[7]. Second, the theory of learned helplessness may also explain the associations between bullying and the above indicators of well-being* |
| McManus and Lord (2012) | Depression  Self-confidence  Self-worth | GHQ-12 | From report page 16: *3 Subjective well-being. 3.1 General well-being and happiness. Table 3.1 Felt unhappy or depressed recently (GHQ 12 item - a_scghqi), Table 3.2 Been feeling reasonably happy recently (GHQ 12 item - a_scghql), Table 3.3 Been losing confidence in self recently (GHQ 12 item - a_scghqj), Table 3.4 Been thinking of self as a worthless person recently (GHQ 12 item - a_scghqk)* |
| Wettstein et al. (2015) | Life satisfaction  Positive affect  Negative affect  Loneliness | SWLS  PANAS  UCLA Loneliness scale | *In this study, we investigate the relationship between the domains of cognitive abilities and well-being in a sample of adults in advanced old age … Well-being contains aspects of cognitive and affective components (such as life satisfaction and positive-negative affect) as well as the assessment of one’s overall social situation (such as loneliness)… Cognitive well-being was assessed with the Satisfaction with Life Scale (SWLS;… The components of affective well-being, positive affect and negative affect, were assessed with the Positive and Negative Affect Schedule (PANAS… Loneliness was assessed with the University of California Loneliness scale* |
| Heine and Browning (2002) | Not identified | Not identified | *Sensory loss, decreased communication performance and psychosocial functioning impacts on one’s quality of life and feelings of well-being… Many older adults with sensory loss successfully adjust, adapt and compensate to their change in lifestyle whilst others have associated health, emotional and social difficulties. Therefore, both sensory loss and the lack of sufficient social communication threaten older adult’s independence and well-being often resulting in devastating social and emotional consequences… A hierarchical needs approach in accordance with the level of realistic independence achievable by clients should be considered to optimize quality of life and well-being… Adaptation to sensory loss, improving physical and mental health, independence, mood and social skills can improve well-being and quality of life* |
| Allen et al. (1999) | Component of HRQoL  Current health status | EQ-5D visual analog scale | *Health-related quality of life (HR-QoL) is a more specific and suitable for design for clinical trial research; it refers to patients’ assessment of their current level of functioning and well-being compared with what they perceive to be ideal… One such instrument is the EuroQoL,3 which was designed as a self-completed questionnaire for use in large-scale surveys of the community. It covers 5 dimensions of health: mobility, self-care, usual activities, pain/discomfort, and anxiety/depression... It also includes a visual analog scale (VAS) that allows respondents to report their valuation of their overall health status… A self-assessed VAS rating of current health status (general well-being) was recorded on a vertical, ruler-type line on which the best and worst imaginable health scores ranged from 100 to 0, respectively* |
| Thurston et al. (2013) | Not identified | 34-item CORE-OM  10-item CORE-OM | *At the pre-counselling assessment, Silvia completed four quantitative measures and one qualitative measure: 1. Clinical Outcomes Routine Evaluation–Outcome Measure 34 (CORE-OM 34): This is a self-completed 34-item scale, yielding four sub-scales (well-being, psychological symptoms, functioning, and risk) and total score… Before each session, Silvia completed two outcome measures: 1. CORE-OM 10: This is a self-completed 10-item scale, yielding four sub-scales (well-being, psychological symptoms, functioning, and risk) and total score… Silvia’s mean well-being scores remained unchanged at 2.75… Judge 1 commented on the CORE scores saying that she would expect functioning and problem scores to change first. She felt the risk score was not relevant because it was low anyway and that the well-being score was always the last to change.* |
| Horowitz and Reinhardt (1998) | Adjustment to vision loss | Adjustment to Blindness | *Fitting (1954) stressed the importance of both functional skill and psychological well-being in adjustment. His measure, Adjustment to Blindness, includes six conceptual and empirical domains: attitudes toward sighted persons, outlook on blindness, family relationships, attitudes toward rehabilitative training, occupational outlook, and general well-being… Although adjustment to vision loss is a component of general well-being, it is not synonymous with adaptation to aging. However, it can be expected that the two constructs are related; that is, more successful adaptation to a chronic visual impairment would be associated with higher levels of general well-being.* |
| Roberts et al. (2010) | Disability-adjusted life years (DALYs):  Mortality burden (years of life lost as a result of premature death)  Morbidity burden (years of healthy life lost as a result of disability) | Morbidity burden: cases * disability weights for mild, moderate, and severe visual impairment  Mortality burden: Age of death from comorbid conditions attributable to visual impairment - life expectancy for people of that age and sex | *Loss of well-being from visual impairment was measured in disability-adjusted life years (DALYs). Disability-adjusted life years have 2 components: the years of life lost as a result of premature death (the mortality burden) and the years of healthy life lost as a result of disability (the morbidity burden). Years of healthy life lost as a result of disability from visual impairment were calculated by multiplying prevalent cases by disability weights for mild, moderate, and severe visual impairment (0.02, 0.17, and 0.43, respectively) based on the global burden of disease study.24 These disability weights represent losing 2%, 17%, and 43%, respectively, of a year of healthy life. Years of life lost were calculated from the age when a person dies from comorbid conditions attributable to visual impairment and the life expectancy for people of that age and sex.* |
| Freitas et al. (1995) | Health status  Personal well-being  QoL | Cantril Self-Anchoring Striving Scale | *The measurements include the following variables: clinical end points (spherical equivalent refraction); visual function (night driving, day driving, near vision, far vision, glare disability); functional status (physical, social, and role functioning, mental status); general well-being (health perceptions, personal well-being, overall quality of life); and satisfaction with surgery (expectations, satisfaction with medical staff, postoperative pain, satisfaction with treatment outcomes)… General well-being: the Cantril Self-Anchoring Striving Scale was used as a quality of life assessment method that is capable of being applied in highly diverse areas of medicine… It was used to elicit subjective assessments of general health status, personal well-being and quality of life* |
| Burmedi et al. (2002) | Used synonymously with subjective well-being  Life satisfaction  Happiness  Contentment  Self-esteem  Morale  Mood  Psychological distress  Trait anxiety  Negative affect  Outlook on life | AVL  ABS  LSI-A  LSI-W  LSES  Mental Health Inventory (MHI) Psychiatric Epidemiology Research Interview (PERI)  PGMS  POMS  Revised Feelings of Inadequacy Scale (RFIS) STAI  Quality of Well-Being (QWB) | *Well-being. In the following, we define well-being in its broadest terms. Hence, research linking vision loss in later life to life satisfaction, happiness, self-esteem, mood, and morale were integrated into this literature review. Table 3 presents an overview of the literature on vision loss and well-being… A vision-related measure of well-being, the Adaptation to Vision Loss scale,76 also showed a moderate correlation with the degree of visual impairment.8 In a few isolated cases, this pattern of results was not observed. Upton et al.,26 for example, did not find any evidence that psychological distress among the visually impaired was higher than those living in the community. Quite unexpectedly, Sinzato et al.61 found the trait anxiety to be significantly lower among diabetics with retinopathy than among diabetics without... vision loss appears to be accompanied by negative affect,62,63 unhappiness, and discontentment… Finally, research on visual loss and general well-being uniformly indicates that visual impairment leads to a decline on a variety of dimensions, including morale, life satisfaction, and mood… Table 3. Well-being among elders with visual impairment. Multi-item measures of well-being: PGMS, LSI-W, RFIS, Factors from MHI & PERI, STAI, LSES, LSI-A, ABS, PMS, QWB, One-item measures of well-being: Morale, Negative feelings, Happiness, Feelings, Outlook on life, Mood, ABS, Affect Balance Scale;65 LSI-A, Life Satisfaction Index – A;66 LSI-W, Life Satisfaction Index – Well-being;67 LSES, Life Satisfaction in the Elderly Scale;68 MHI, Mental Health Inventory;69 PERI, Psychiatric Epidemiology Research Interview;70 PGMS, Philadelphia Geriatric Morale Scale;71 PMS, Profile of Mood States;72 RFIS, Revised Feelings of Inadequacy Scale;73 STAI, State-Trait Anxiety Inventory;74 QWB, Quality of Well-Being* |
| Marques et al. (2020) | Disability-adjusted life years (DALYs)  Quality-adjusted life years (QALYs)  Years of sight loss | Not identified | *We will describe the main reported loss of well-being measures and its general assumptions. Loss of well-being measures will be summarised in their natural units (eg, QALYS and DALYS) rather than reported in their monetised value since there is no consensus on assigning a monetary value to health outcomes21 26 39 and because there is no common acceptable value across countries.* |
| Kutzbach et al. (2009) | Not identified | Not identified | *Additionally, clinical measures of physiologic function or response to an intervention may actually correlate poorly with functional capacity and overall well-being.* |
| Hasan et al. (2018) | Not identified | Qualitative interviews | *Our analyses of these narratives indicates that access to ICTs training and resources played a pivotal role in promoting well being of participants by enhancing their social, economic, political, and educational opportunities; and by providing protective security for them.* |
| Rooney et al. (2018) | Not identified | Qualitative interviews | *Now, with visual impairment set to increase globally and acknowledging the recognised link between quality of dwelling and wellbeing, this article aims to examine the experiences of visually impaired people living in lifetime homes… Home is many things; it is a container of wellbeing, a place of security, a space where social life, leisure and recreation take place… Furthermore, location is fundamental to wellbeing in supporting independence and preventing isolation* |
| Naylor and Labbe (2017) | Not identified | ORS | *The ORS (…) is a four-item ultra-brief measure given to participants to monitor their individual progress from session to session… They were then asked to rank each of the four areas of their life (i.e. individual, personal well-being, interpersonal well-being, social well-being, and overall, general well-being) on a scale of 0–10, 0 being the worst and 10 being the best.* |
| Bambara et al. (2009) | Adjustment to vision loss  Depression  Distress (psychological/emotional) | Not identified | *The impact of emotional support provided by family members on the well-being of persons with vision loss has also been examined (…). For example, Jacobs (1984) showed that older adults with low vision who reported positive adjustment to vision loss also reported good communication with family members, had well-established networks of social support, and stressed the importance of family members’ positive attitudes…. Perceived and received affective support was also shown to be related to fewer depressive symptoms in persons with low vision… Mental health practitioners, broadly speaking, are uniquely trained in the application of evidence-based psychological assessments and psychotherapeutic interventions to understand, prevent, and relieve psychological distress and psychopathology and to promote well-being… This perspective espouses that certain thoughts or ways of interpreting the world can cause emotional distress or result in problems in daily living. Likewise, certain behaviors, such as avoidance of situations, also maintain distress..* |
| Geruschat et al. (2015) | Not identified | FLORA | *During discussions with the US Food and Drug Administration (FDA), it became clear that an additional assessment of real-world functional vision and well-being was necessary to complete the picture of the effect of restoration of vision on subjects’ lives… The self-report takes into consideration the patient’s point of view on the impact of the Argus II on their well-being… The case report documents evaluators’ findings from two points of view: functional visual effects they observed and the subject’s self-reported general observations of well being and satisfaction (or displeasure)… The Functional Low-vision Observer Rated Assessment was developed to evaluate functional vision and well-being in a population of subjects whose ultra-low vision had been restored by the Argus II Retinal Prosthesis System.* |
| Wilson et al. (1998) | Not clear | SF-36 | *Purpose: This study aimed to determine whether patients with glaucoma have different functional status and well-being than patients without glaucoma… The Medical Outcomes Short Form-36 (SF-36) has been studied extensively and has been shown to be a reliable, valid, and statistically proven measure of functional status, well-being, and general health perception* |
| Reinhardt et al. (2006) | Depression  Adaptation to vision loss (acceptance of vision loss, positive attitude toward rehabilitation, positive outlook toward social relationships) | 20-item CES-D  24-item AVL | *Perceived affective support may be an especially important resource with an outcome variable that assesses one’s general sense of well-being, including depressive symptoms… Well-being. Outcomes included the widely used, 20-item measure of depressive symptomatology, the Center for Epidemiological Studies Depression scale… The second outcome was a measure of domain-specific adaptation, the Adaptation to Vision loss scale (…). This 24-item scale assesses the extent to which the person accepts vision loss in a realistic manner, has a positive attitude toward rehabilitation, and a positive outlook toward maintaining relationships with family and friends* |
| Kelly et al. (2021) | Component of VRQoL and HRQoL  Not clear | SF-36v2  NEI-VFQ  IND-VFQ | *Conclusions: Among patients treated with methotrexate or mycophenolate mofetil for uveitis, VRQoL and HRQoL improved significantly over the course of 1 year and did not differ by treatment allocation. These findings suggest that antimetabolites could improve overall patient well-being and daily functioning… This analysis used validated scales (SF-36v2, NEI-VFQ, and IND-VFQ) to measure QoL and to provide a comprehensive measure of health and well-being instead of relying solely on ocular outcomes.* |
| Wilson and Lee (2000) | Component of HRQoL  Not identified | SF-36 | *The patient’s perspective of his or her own health status as it relates to functioning and well-being is referred to as health -related quality of life… The SF-36, unlike the SIP, aims to operationalize health on the more subjective dimensions of well-being and health perceptions in addition to the more objective dimension of behavioral functioning… It evaluates both functional behavior and subjective states of well-being and self-perceptions of health… It [VF-14] emphasizes symptoms and difficulties with tasks, but it neglects other known dimensions of quality of life, such as subjective states of well-being and self-perception of vision* |
| Burton et al. (2016) | Social isolation  Social interaction  Enjoyment | Qualitative interviews | *Maintaining wellbeing. Those participants who lived alone described times when they felt completely isolated from others and some felt that giving up favourite pastimes was just a part of growing old… The majority of participants relied on existing friendship groups and family to get out and about; most did not feel comfortable seeking support elsewhere. One participant imagined that support groups ‘overdo it a bit’ which was a threat to him as ‘an independent-minded individual’ (Brian). But those who did attend groups were hugely grateful for what they had given them; for most that was a revived sense of social interaction and enjoyment… Support groups also acted as a way to maintain wellbeing in terms of an opportunity to be socially engaged and provided an opportunity to interact with other older people coming to terms with sight loss.* |
| Chia et al. (2004) | Component of HRQoL  Not clear | SF-36 | *…the use of the SF-36 enabled comparison of the impact of visual impairment on health-related quality of life (HRQOL) with the impact from a range of other medical conditions… The SF-36 contains 36 items measuring eight dimensions of health and well-being: “physical functioning,” “role limitations due to physical problems,” “bodily pain,” “general health perceptions,” “vitality,” “social functioning,” “role limitations due to emotional problems,” and “mental health.”… Physical and mental component scores (PCS and MCS, respectively) were summary measures calculated using factor analysis and Australian normalized scores* |
| Menon et al. (2020) | Not clear | Case notes | *It is well documented that sight loss can significantly reduce quality of life by limiting activities of daily living,4 affecting employment and financial status5 and potentially leading to social isolation. Furthermore, there is an increased risk of depression, suicide,6 cognitive decline7 and falls8 in those with visual impairment. Patients with sight impairment therefore require access to support services for help with all aspects of their well-being* |
| Mojon-Azzi et al. (2010) | Not identified | Not identified | *Analysis of this dataset from 12 countries demonstrates the strong impact of self-reported visual impairment on individual employment, and therefore on job satisfaction, productivity, and well-being… Our results additionally demonstrate a strong impact of self-reported visual impairment on individual employment, and therefore on worker productivity and well-being. This is an important aspect of job satisfaction, productivity, and well-being that needs further research.* |
| Rajak et al. (2016) | Not identified | WHO/PBD-VF20  WHOQOL-BREF | *No studies have measured the long-term overall effect that trichiasis surgery has on the different QoL domains and overall wellbeing… VRQoL was measured using the WHO/PBD-VF20 tool [14]. This contains 20 questions sub-divided into three subscales: visual symptom (3 questions), general functioning (12 questions) and psychosocial (4 questions)… HRQoL was measured using the WHOQOL-BREF [15]. This contains 26 questions, subdivided into four domains: physical health, psychological, social relationships and environment in the past four weeks [15,19,20]. The first two questions assess general QoL and health… Overall this study demonstrated that trichiasis surgery substantially improves both vision and health related QoL regardless of the visual acuity improvement, suggesting that the effect of trichiasis surgery goes beyond preventing the risk of blindness and improves the overall wellbeing and health perception of affected individuals.* |
| Holmes et al. (2018) | Not clear | Qualitative interviews | *A comprehensive question guide about health and well-being was developed based on review of the literature. This included questions about the impact of VI and barriers to accessing eye care services… Our findings show that these elders view loss of vision as having a major impact on health and well-being… Figure 1. Summary of findings on the impacts of vision impairment for elders. Poor vision reduces elders’ ability to: -Carry out personal tasks, Carry out domestic tasks, Care for and teach grandchildren, Attend religious activities, Undertake paid work, Participate socially, Access information, including about medicines and health, Travel, including for health care, Prevent falls. Resulting in: -Poor health, Restricted life, Reduced ability to contribute to family, Reduced ability to contribute to community, Loneliness, depression, fear, Dependency, Increased risk of falls and injury… Our findings show that these elders view loss of vision as having a major impact on health and well-being.* |
| Dubey et al. (2020) | VRQoL  Social well-being (fulfilling responsibilities, social interaction, interaction with the world)  Personal well-being (personal safety, personal care, leisure activities)  Overall trouble with vision  Satisfaction with present visual health | Glaucoma Quality of Life 15 (GQL-15)  Self-developed social function scale (SFS) | *The Glaucoma Quality of Life 15 (GQL 15) and a self developed social function scale (SFS) were utilized to assess patients’ wellbeing… Six-item SFS was developed based on literature review, clinical expertise, and data from an initial pilot study with 26 glaucoma patients. This questionnaire assessed disability in the following areas: personal care, personal safety, and ability to fulfill responsibility, navigate oneself outdoors, interact with society, and perform leisure activities. Two global questions were asked to capture an impression of patient general wellbeing by inquiring about overall trouble with vision and satisfaction with present visual health. Individual items on the social function questionnaire were grouped under two broad categories to reflect on the primary aspects of functional performance: 1. Personal wellbeing (personal safety, personal care, leisure activities) and 2. Social wellbeing (fulfill responsibilities; interact with the world, social interaction).* |
| Delyfer et al. (2020) | Not Identified | FLORA | *Figure 1. FLORA Final Rating Results. Trained observers rated the impact of the Argus II on patients’ well-being and functional vision at 12 and 24 months post-implantation. The scale included five ratings: positive (the System has improved the subject’s well-being and functional vision), mild positive (the System has improved the subject’s well-being or functional vision, but not both), neutral (the System has had no impact on the subject’s well-being or functional vision), mild negative (the System has worsened the subject’s well-being or functional vision, but not both), or negative (the System has worsened the subject’s well-being and functional vision)… On the FLORA, at Year 1, 70% of patients in our series showed improvements in either well-being or functional vision, with 41% showing improvements in both categories* |
| Engel et al. (2000) | Physical health (overall health status, days in bed, nights in hospital, nights in nursing home, times talked to doctor, days of in-home services, number of falls)  Activities of daily living (difficulty and confidence with preparing meals, walking inside/outside the home, using public transportation, taking medications, difficulty with using the phone, paying bills)  Social activities (sees relatives, friends, visits on phone, attends club-related/senior center activities, engages in hobbies, physical activity, feels isolated, satisfaction with activity)  Mental health/morale (depression, control) | Self-developed questions  CES-D | *We asked participants to assess four areas: physical health, activities of daily living, social activities, and mental health… Mental health included an assessment of depressive symptomology using the short form of the Center for Epidemiologic Studies Depression Scale (…) and their questions about sense of control… Changes in Well-Being. For the details of the findings on the impact of rehabilitation services on physical health, activities of daily living, social activities, and mental health, see Table 2…Table 2. Changes in Outcome Areas of Well-Being. Area: Health Status: overall health status, days in bed, nights in hospital, nights in nursing home, times talked to doctor, days of in-home services, number of falls. Activities of daily living: Difficulty with using the phone, preparing meals, paying bills, walking inside the home, walking outside the home, using public transportation, taking medications. Feels safe or confident in preparing meals, walking inside home, walking outside home, using public transportation, taking medications. Social activities: Sees relatives, sees friends, visits on phone, attends club-related activities, attends senior center activities, engages in hobbies, moderates physical activity, feels isolated, satisfaction with activity. Morale: Total locus of control, total depressive symptoms.* |
| McCormack (2021) | Managing better  Feeling safe  Relaxed state | Qualitative interviews | *Furthermore, representatives of OSPVIs noted the differences in wellbeing between their clients who had received a relatively high amount of training in independence skills versus those individuals who received minimal training...Those that had received independent living skills prior to the storm were able to manage better and were in a more relaxed state in their homes because they already had that training of where things were located in their home, how to access things, their own personal wellbeing, and that their homes were very well organized so that they were able to really maneuver within their home environment in a way that felt safe... This organization’s clients who “manage[ed] better, were in a “relaxed state,” and “felt safe” during the recovery process were linked with having received training in skills in independence prior to Hurricane Maria* |
| McIlvane and Reinhardt (2001) | Not clear | Not identified | *Relationships with family and friends influence well-being across the life span and low levels of support have been consistently linked with lower levels of well-being (…). Older adults may be especially vulnerable to the detrimental effects of stress on their well-being when they do not have adequate levels of social support… In the present study, we assessed three commonly used conceptualizations of support in terms of their effect on multiple outcomes including psychological well-being (i.e., depressive symptomatology and life satisfaction) and adaptation to vision loss in a sample of older men and women… We assessed both positive and negative aspects of life quality, as earlier work has stressed the importance of including both of these aspects in studies of well-being (…). Further, we examined the impact of these support variables on both the general outcome of well-being and the domain-specific outcome of adaptation to vision loss for comparison. Thus, in this study, overall well-being is not considered a proxy for adjustment to vision loss… The purpose of the present study is to extend this earlier finding by examining the interactive effects of the support variables (i.e., family and friend support) and gender on psychological well-being (i.e., depressive symptoms and life satisfaction) and adaptation to vision loss in a sample of elders dealing with chronic vision impairment… We tested depressive symptoms and life satisfaction as a system as these two variables are highly correlated (r = −.72, p < .001) and conceptually similar (i.e., negative and positive global measures of well-being;… we assessed depressive symptoms and life satisfaction together as psychological well-being… Thus, whereas having higher family support is an important part of one's general well-being, having supportive relationships and interacting with peers may be what encourages older adults to continue their daily functioning when faced with chronic impairment.* |
| De Bel et al. (2016) | Stress  Self-acceptance | Qualitative interviews | *Implications for Rehabilitation: Different aspects of career outcomes (e.g. age of retirement) and wellbeing outcomes (e.g. self-acceptance and stress) associate with identity concealment patterns of individuals throughout their careers… The second aim of this study is to describe the career and well-being outcomes associated with different work trajectories, and with various concealment and passing pathways… Our results suggest that there is no clear answer to the question of whether disclosure is advantageous or disadvantageous for an individual’s career outcomes and general well-being. While revealing the identity may reduce the stress associated with trying to pass and can enhance self-acceptance [16], being open may also have negative effects on the person’s career outcomes* |
| Cumberland et al. (2015) | Health status  Health satisfaction  Happiness | Self-report | *This provides a unique opportunity to investigate the frequency of refractive laser surgery in the UK and to compare the socio-demographic profile of those electing and not electing to have this treatment, as well as self-reported outcomes related to their social activities and well-being… Table 5: Distribution by social participation activities and general well-being factors comparing those reporting laser refractive surgery (total, unilateral and bilateral) and those with refractive errors (i.e. eligible) but no reporting surgery. Social activities, health rating, health satisfaction, visit friends or family, happiness* |
| Burton et al. (2015) | Dwelling (feeling at home with what has been given)  Mobility (feeling able to explore new existential possibilities)  Spatiality  Temporality  Intersubjectivity  Mood  Identity  Embodiment | Qualitative interviews | *Galvin and Todres (2011) challenge the dominant deficit model of ageing and argue that well-being is a complex phenomenon involving the interplay of many aspects of a patient’s life, not just the absence of illness. They propose a conceptual framework to illustrate the multiplicity of kinds of well-being drawing on Heidegger’s (1962/1927) ontological writings on homelessness and homecoming. The framework is structured around two factors which make up the experience of well-being; dwelling (feeling at home with what has been given) and mobility (the feeling of possibility and a sense of adventure; …) and the six phenomenological–philosophical lifeworld constituents that make up human experience (spatiality, temporality, intersubjectivity, mood, identity and embodiment)… Therefore, the deepest possibility of well-being involves a paradoxical unity of dwelling and mobility (Dwelling–Mobility) in which an individual feels both at home with what has been given and experiences the opportunity of exploring new existential possibilities. This is supported by Koren’s (2011) work illustrating that well-being is related to the simultaneous occurrence of change and continuity as we age* |
| Glen and Crabb (2015) | Not identified | Qualitative interviews | *Others imposed self-restrictions or gave up activities, thus compromising well-being and independence.* |
| McKean-Cowdin et al. (2010) | Component of VRQoL  Not identified | NEI-VFQ | *Clinically important, longitudinal changes in visual acuity (2 line changes or greater) were associated with significant changes in self-reported visual function and well-being… The National Institute Visual Function Questionnaire (NEI-VFQ) was designed to measure areas of vision-targeted functioning and well-being that were identified as important by persons with eye disease… Higher score represents better visual functioning and well-being… The data suggest that improvements in vision following cataract surgery, glasses or other clinical interventions are associated with gains in perceived well-being and function* |
| McMullan and Butler (2019) | Joy  Depression | Qualitative interviews | *Four themes were found to describe experiences: autonomy and well-being, accessibility, community interactions and self-regulation… Theme one: autonomy and well-being. … The contribution to participants’ well-being was apparent. One spoke of the joy given, and another spoke of how it is protective factors, “I was basically housebound before I got one and that was quite a depressing time”* |
| Araki et al. (2004) | Morale (future-oriented optimism/pessimism):  Agitation  Attitude toward own aging  Lonely dissatisfaction | PGCM (Japanese version) | *General well-being in elderly diabetic patients was evaluated using the PGC morale scale (Japanese version).10 The morale refers to a future-oriented optimism or pessimism regarding the problems associated with living and aging. It consists of three components: agitation, attitude toward own aging and lonely dissatisfaction. Low well-being was defined as a morale score of seven points and under. The low score of morale scale suggests that one has a low well-being.* |
| Brown et al. (2009) | HRQoL (activities, participation, environmental factors, personal factors – social relationships, goals, emotional well-being) | e.g. 25-item NEI-VFQ  IVI | *Health-related quality of life (HR-QoL) is recognized as an important indicator of wellbeing… Quality of life incorporates all components of the WHO International Classification of Functioning, Disability and Health – activities and participation and environmental and personal factors… Scales that measure vision-specific functioning, such as the 14-Item Visual Functioning Index (VF14) [26], have been excluded from this review as they focus primarily on the International Classification of Functioning, Disability and Health components, body structures and functions or activity performance, and exclude participation, environmental and personal factors (such as social relationships, goals and emotional wellbeing)* |
| Deverell et al. (2019) | Physical health (keep active, motivation to exercise)  Mental health (mood, optimistic energy, anxiety, depression, despair, loneliness, happiness, joy, laughter, feeling good, relaxed, at ease, sense of security, feeling accepted, receiving assurance, confidence, capacity)  Activities (social engagement)  Connections (social interaction)  Agency | OMO  VROOM | *The tools were feasible to implement and reduced qualitative information about Orientation and Mobility Outcomes (OMO) and Vision-Related Outcomes in Orientation and Mobility (VROOM) each to a number out of 50, facilitating comparisons. Benefit 3: Wellbeing: The dog had a beneficial impact on the physical and mental health of many participants, but it also served a therapeutic function during illness... Physically, walking helped clients to keep fit, combat obesity, and get moving again after illness. A woman with acquired brain injury described her balance as “terrible,” and although she never felt graceful or fluid, she always felt better after walking. A bonus was that motivation to exercise did not all have to come from the person—the dog could be quite insistent about the need to go out. The dog had an equally important impact on mental health: recognizing mood and making physical contact, shaking hands, or sitting alongside on the couch. Multiple clients described how the dog helped to get them moving when immobilized by anxiety: headbutting their knee reassuringly or taking them away from stressful, milling crowds. Depression and despair seemed to weigh especially heavily with middle-aged participants who’d had to end their previous employment because of vision loss but had not yet found a new direction. The need to care for the dog gave a reason to get up every day. Multiple clients enjoyed the way the dog shadowed them around the house, alert to their needs. This meant that bouts of ill health were not so lonely... Wellbeing was captured in the Part B–Activities, Connections, and Agency subscales. These scales gave a loose indication of mental health and the nourishing quality of the relationship with the dog that underpinned their successful mobility together… Figures 1. Mind map showing what clients (n = 51) gained from choosing guide dog mobility. Well-being in sickness and health – promotes physical health, supports mental health (offers acceptance, feels good, relaxed, at ease, provides sense of security, assurance, inspires confidence, builds capacity, brings joy, happiness), provides therapy (responds to mood, brings optimistic energy, initiates care, motivates recovery)* |
| McGwin and Owsley (2007) | Psychological health  Physical health  ADLs  Social integration  Economic viability  QoL | Questionnaires:  SF-36  NEI-VFQ  VF-14  Activities of Daily Vision Scale  Visual Activities Questionnaire  10-item Vision Core Measure (VCM1)  Driving Habits Questionnaire  WHO Falls Questionnaire  Low Luminance Questionnaire  Reading items from the National Health Interview Survey  Glaucoma Symptom Scale  CES-D  GDS  GHQ  POMS  Behaviours  Administrative records | *Methods: Three methods are described, which are largely complementary of each other. (1) Questionnaires are patient-centered in that they provide the person’s own self-reported perspective on difficulty in engaging in everyday activities, psychological well-being, and/or health status. Examples are generic and vision-targeted health-related quality of life instruments, domain-specific questionnaires, and instruments that screen for psychological comorbidities. (2) Direct assessment of actual behavior provides information about the functional competences the patient has. Examples are mobility performance and reading speed. (3) Administrative records maintained by government, healthcare, and other agencies provide information relevant to the existence of adverse or detrimental circumstances that engender decreased health and well-being. Examples are healthcare utilization, employment history, and motor vehicle collision involvement… There are several ways to measure the personal burden of eye disease and vision impairment that provide information about the person’s own perspective on his/her health and well-being and functional competences… The term “personal burden” can be used to refer to the impact or implications of the eye disease for the person’s overall well-being, including but not limited to psychological and physical health, ability to engage in common daily tasks and activities, social integration, and economic viability… The defining feature of questionnaire measures is that they are patient-centered in that they provide the person’s own self-reported perspective on his/her quality of life, addressing domains such as difficulty in engaging in everyday activities, psychological well-being, and/or health status… One of the most common generic quality of life instruments used in research over the past two decades is the SF-369 that provides a profile of functional health and well-being, as well as physical and mental health subscale scores* |
| Marques-Brocksopp (2014) | Eudemonic dimension:  Self-esteem  Self-control  Hedonic dimension:  Hope  Life satisfaction | Not identified | *…this study sought to consider the role mindfulness may play in the well-being of individuals with a visual impairment, and thus building upon the wider literature on spiritual well-being and adaptation to long-term health condition… The interview schedule was devised to talk first about the participants’ visual impairment and then move on to their mindfulness practice and the impact it was perceived to have on their well-being… These findings therefore suggest that mindfulness may positively influence both the eudemonic and hedonic dimensions of well-being by providing meaning and direction and thus increasing self-esteem and self-control, as well as provoking feelings of hope and life satisfaction* |
| Layat et al. (2017) | Component of QoL  Not identified | Patient interview | *The reasons for the interview were disclosed to the patient in these terms: ‘‘To evaluate the clinical care pathway of the patient, so as to improve his or her management and rehabilitation’’, inviting him or her to respond to some questions allowing us to retrace with the patient the history of his or her disease, the care pathway and to specify the effects of the disease on quality of life… It was discovered that 77% of the patients stated that their well-being was affected and that they were concerned about the progression of their disease….The quality of life in relation to vision, with the effect of visual deficiency on the person’s well-being, the repercussions on daily life, its effect on social and professional life would need to be questioned to a greater extent… Figure 4: Panel A: Impact of visual involvement on quality of life [although this seems to be mislabelled and should be panel B]: Autonomy, mobility, social life, well-being, professional activities, professional concerns regarding disease* |
| Weber and Wong (2010) | Not clear | Qualitative interviews | *The following themes indicated relationships between the subject’s well-being and the ability to cope with vision loss… HOPE AND ACCEPTANCE. Hope and acceptance indicated an increasingly optimistic outlook on living with visual impairment. This theme suggests that participants have come to terms with vision loss... SELECTIVITY AND CONFIDENCE. Other successes involved believing in oneself and having the confidence to selectively learn new approaches to living with vision loss… OPTIMISM AND COMPENSATION. Participants that expressed optimism also tended to score higher on the AVL scale and reflected more positive well-being… SORROW AND REMORSE. Regrets were more commonly expressed than successes. Sorrow included bitterness, a sense of grief, frustration, and fear… BLAME, DEPENDENCE, AND DENIAL. Many participants alluded to pessimistic personal thoughts that comprised of pessimistic thoughts toward vision loss, others, and themselves. Blame took the form of holding others responsible, particularly the ophthalmologist. Dependence of any kind was viewed as unconstructive. Asking for help and being a burden on others was also expressed disapprovingly. The necessity to selectively adjust future plans and lifestyle were considered to be negative aspects of a forced and unwanted new life… FRUSTRATION AND FEAR. Frustration was the most common negative feeling expressed regardless of the length of time a participant had experienced sight loss… Ultimately, the capability to cope with vision loss can lead to improved well-being and function with advice seeking and sharing* |
| Heine et al. (2020) | Health status  Mental health (depression)  Social health (perceived social activity level) | Self-rated health status  Psychogeriatric Assessment Scales (PAS)  Self-rated social activity level | *DSL also inhibits independence, limits one’s activities, restricts participation in society and influences well-being… Although the majority of older women take measures to prepare for their future care needs, many miss key steps recommended for their future potential well-being… The MELSHA survey was designed as a longitudinal survey to follow up the changing health, health service use and well-being among older Australians over 16 years (9 waves in total from 1994 to 2010) when their age increased… Self-rated general health was measured according to respondent’s answers when they were asked to rate their own health compared to people of the same age… Mental health (as measured by the presence or absence of depression) was measured using a cut-off score of 5 or more on the Psychogeriatric Assessment Scales (PAS) [45] (score ranges were 0–12). Perceived current social activity level was used, where 1 = not enough and 0 = about right or too much to identify social health, as it represents both need and practice. Use of community services was categorised as ‘yes’ if a respondent answered ‘yes’ to either of the two questions: “Do you use any organized community services such as the home and community care program?”; “Who helps you with household duties or personal care which you cannot do on your own? Organized community services (e.g., home and community care program)?”… The impacts of declining physical and social health and DSL are numerous, affecting mobility, social connectedness and one’s sense of well-being.* |
| Boerner and Cimarolli (2005) | Depression  Life satisfaction | 10-item CES-D  SWLS | *The relationships between importance of life goals, goal interference and well-being in this population were also examined… Depression was measured with the 10-item Center for Epidemiological Studies Depression Scale (CES-D)... Life satisfaction was assessed with the five-item Satisfaction With Life Scale… Goal interference was significantly related to both well-being indicators. As expected, the directions of these links were positive with depression and negative with life satisfaction, suggesting that those who reported higher levels of interference in these life domains due to their vision loss were also more likely to report more depressive symptoms and lower levels of life satisfaction.* |
| Kaur et al. (2012) | VRQoL | Glaucoma-specific and vision-specific QoL instruments | *While there is no gold standard QOL assessment scale, glaucoma-specific and vision-specific instruments are better than generic tools to assess the impact of the disease per se on the patients’ overall well-being* |
| Bray et al. (2017) | Capability | ICECAP-A | *Purpose: To determine the incremental cost-effectiveness of portable electronic vision enhancement system (p-EVES) devices compared with optical low vision aids (LVAs), for improving near vision visual function, quality of life and well-being of people with a visual impairment… The ICECAP-A is a validated capability measure focussing on well-being beyond health and is scored from 0 (no capability) to 1 (full capability)… Additionally, the ICECAP-A was used to calculate an estimated cost per year of full capability (YFC); the YFC approach is an alternative to the QALY framework which focuses on a broader measurement of well-being beyond health and physical functioning* |
| Bergeron and Wanet-Defalque (2013) | Used synonymously with subjective well-being  Life satisfaction | SWLS | *The aim of this study was to explore the pattern of adaptation to visual impairment in terms of denial, acceptance, well-being, and depression, among patients with varying lengths of time since diagnosis… All participants answered questions in the same order: general demographics, coping strategies (BC;…), depressive symptoms (the CES-D–Short Form;…), and well-being (Satisfaction with Life Scale;… The Satisfaction with Life Scale (…) is a 5-tem questionnaire measuring subjective well-being, which is described as ‘the personal ability to enjoy oneself and one’s own life’* |
| Abas et al. (2009) | Mental health  Self-realisation  Coping  Contributing to communities (social participation)  Social relationships  Enjoyment  Ability to let go of worries (worry)  Life satisfaction  Autonomy  Self-acceptance | Not identified | *Positive mental health "which allows individuals to realise their abilities, cope, and contribute to their communities" [1] and the capacity to sustain social relationships are key dimensions of wellbeing [2]. Wellbeing can be measured in terms of positive psychological symptoms (such as being able to enjoy things and to let go of worries) or life satisfaction, but increasingly multidimensional scales are used which include concepts such as autonomy, self-acceptance and relations with others* |
| Mitchell and Bradley (2001) | Used synonymously with psychological well-being  Anxiety  Depression  Energy (feeling energetic, active or vigorous, feeling dull or sluggish, feeling tired, worn out, used up or exhausted, waking up feeling fresh and rested)  Positive well-being (feeling happy, satisfied or pleased with my personal life, have lived the kind of life I wanted to, feeling eager to tackle my daily tasks or make new decisions, feeling I could easily handle or cope with any serious problem or major change in my life)  Negative well-being (Feeling tearful, downhearted and blue, feeling afraid for no reason at all, getting upset easily or feeling panicky) | W-BQ12  W-BQ22 | *This paper reports the evaluation of a generic measure of psychological well-being, the 12-item Well-Being Questionnaire (W-BQ12), for use with people who have the chronic eye condition, macular disease (MD)… Unforced factor analysis elicted the expected three factors representing constructs of positive well-being, energy and negative well-being. A forced single-factor solution supported use of the whole scale to measure total general well-being… This paper reports the psychometric evaluation of a generic measure of well-being, the 12-item well-being questionnaire (W-BQ12) for use with people with MD… The original scale developed contained 22 items (W-BQ22) and had a four-factor structure with subscales measuring depression, anxiety, energy and positive well-being… The W-BQ22 focuses on the cognitive symptoms of mood states… Work on a Japanese translation of the W-BQ22 led to development of a 12-item version with three subscales: negative well-being (NWB), energy and positive well-being (PWB)… The W-BQ12 asks people how often they have experienced the feelings mentioned in each statement over the past few weeks… All items intended for the 4-item NWB subscale were negatively worded and all items intended for the 4-item PWB subscale were positively worded. Two items intended for the 4-item energy subscale were positively worded and two were negatively worded… Table 1. W-BQ12 items and factor analysis rotated component matrix. Neg 1 I have crying spells or feel like it, Neg 2 I feel downhearted and blue, Neg 3 I feel afraid for no reason at all, Neg 4 I get upset easily or feel panicky, Energy 1 I feel energetic, active or vigorous, Energy 2 I feel dull or sluggish, Energy 3 I feel tired, worn out, used up or exhausted, Energy 4 I have been waking up feeling fresh and rested, Pos 1 I have been happy, satisfied or pleased with my personal life, Pos 2 I have lived the kind of life I wanted to, Pos 3 I have felt eager to tackle my daily tasks or make new decisions, Pos 4 I have felt I could easily handle or cope with any serious problem or major change in my life… Psychological well-being is one psychological outcome of relevance for QoL, and measures of well-being may be expected to correlated positively with measures of QoL.* |
| Dev et al. (2014) | Component of HRQoL  Mental health (role limitations due to emotional problems, energy/vitality, mental health, social functioning) | SF-36 mental component subscales:  Role limitations due to emotional problems (3 items)  Energy/vitality (4 items)  Mental health (5 items)  Social functioning (2 items) | *The SF-36 instrument yields practical, reliable, and valid information on functional health and well-being from the patient’s point of view. This instrument consists of eight subscales [36,37]: physical functioning (10 items), role limitations due to physical health (4 items), bodily pain (2 items), general health (5 items), role limitations due to emotional problems (3 items), energy/vitality (4 items), mental health (5 items), social functioning (2 items). SF-36 has both the physical and mental components. It yields psychometrically-based physical and mental health summary measures. The first four subscales belong to the physical component (PC) and the latter four subscales belong to the mental component (MC) [22,23,36-38].… These could be the reasons for the three domains of the PC-physical functioning, role limitations due to physical health and general health being more affected by VI than the other subscales. The decrease in function and well-being associated with VI is integrated into a person’s HRQoL and is not easily isolated from other medical conditions* |
| Kahaly et al. (2002) | Not clear | SF-36 | *Having blurred vision and/or diplopia has a detectable and significant impact on functional status and well-being, especially in role limitations caused by physical health problems… Marked and significant differences from the control group were especially observed for the following items: vitality, social functioning, mental health, health perceptions, and body pain. MOS-36 did not correlate with the duration or severity of the ophthalmopathy. These results demonstrate the impact of a common visual symptom on health status and well-being, as measured by the MOS-36… In conclusion, we have shown that TAO has a large influence on the quality of life of these patients. The negative impact on well-being seems not to be related to the usual clinical assessment.* |
| Legro (1991) | Component of QoL  Perceptions of health  Energy/fatigue  Pain  Life satisfaction  Feelings about ability to cope  Satisfaction with outcome  Crying episodes on seeing effects of progressive dermatological disease  Income level | Not identified | *In order to formulate wise public policy in this area it is important to describe the effects of this treatment on patient’s quality of life… General Well-Being. Tarlov et al33 suggested that items such as health perceptions, energy/fatigue, pain and life satisfaction be included in this category. To date, there are no published findings which report life satisfaction or general well-being following cataract surgery… Table 2. Results of Outcome Studies. Groups by Topics. General Well-Being: satisfaction with life activity, feelings about ability to cope, satisfaction with outcome, crying episodes on seeing effects of progressive dermatological disease, income level* |
| Mitchell and Bradley (2006) | Not clear | PGWB | *4. Perceived quality of health care, satisfaction with the diagnostic consultation and their relationship to patient well-being… For example, the difference in well-being between the satisfied and dissatisfied groups was greater in more recently diagnosed people (< 2 years) than in those who had had MD for longer… The majority of MDSQ respondents (1247 (90%)) had been told that 'nothing can be done to help with your MD'. Of those 757 (61%) said they felt depressed or anxious on hearing this news and 54 (4.3%) said it led them to feel suicidal… The control group reported improvements in general health, self-confidence and loneliness (not from a published scale), but not to well-being (Psychological and General Well-being Index [123]) at 6 months after light adaptation. The intervention group showed significant improvements in the same items but also reported improved physical condition, appetite, social contacts, self-confidence, temper, depressed mood, vitality and well-being (not psychometrically evaluated measures)* |
| Adigun et al. (2014) | Not clear | Not identified | *The term “quality of life” is popularly used to describe an individual’s overall sense of well-being, and includes aspects such as happiness and satisfaction with life as a whole… The theory of quality of life explains it as consisting of a range of subjective and objective aspects. The subjective aspects of quality of life deal with well-being, satisfaction, happiness, and meaning of life, while the objective aspects can be assessed using factors such as conformity to cultural norms, fulfillment of needs, realization of life’s potential, and maintaining biological order, ie, the ability to function within a societal norm* |
| Brunnström et al. (2004) | Positive psychological state  Behavioural-emotional control  Feeling of belonging  Anxiety  Depression  Loneliness  Mood (faces life in a good spirits)  Interest in everyday matters  Life satisfaction  Energy/Vitality | PGWB | *Well-being includes a positive psychological state, behavioural-emotional control, feeling of belonging and a lack of anxiety, depression and loneliness (…). This study has concentrated on evaluating the effect of basic light adjustments on ADL, quality of life and well-being before and after adaptation in the home. It has also analysed how an additional intervention to provide task lighting in the living room, affected the quality of life and well-being of the visually impaired… Lighting measurements were made and interviews were conducted to determine ADL and quality of life/well-being before light adjustment…Questions in the interview about the perceived quality of life were based upon the result of a previously performed study into the effects of lighting on quality of life for the elderly (…). The factors dealt with were loneliness, health, humour, self-esteem, physical fitness, appetite, contact with relatives and others... An evaluation of well-being was made using three dimensions in the psychological and general well-being (PGWB) scale. The dimensions analysed were: • Well-being: faces life in a good spirit, satisfied with life, interested in daily goings-on • Vitality: full of energy, wakes up refreshed and rested, peps up others, active, not tired and worn out • Depression: often feels depressed, dejected, worried and experiences despair… Figure 2 shows that the comparison group has the same quality of life and well-being both before and after the adaptation. No significant change can be seen… Figure 2. Quality of life in comparison group before and 6 months after intervention. General health, Physical condition, Appetite, Contact relatives, Other contacts, Self-confidence, Temper, Loneliness, Depressed mood, Vitality, Well-being* |
| Kowalski et al. (2012) | Not clear | Visual Function Questionnaire - Utility Index (VFQ-UI)  25-item NEI-VFQ | *The NEI VFQ-25 was designed to measure vision-related functioning and well-being in persons with ocular diseases [29, 32]. It contains 25 items organized into the 12 subscales of the original 51-item questionnaire with a reduced number of items within each… Scores range from 0 to 100, with higher scores indicating better patient-reported vision-related functioning and well-being… Table 7 The VFQ-UI health-state classification. This classification system was formed based on the following items from the NEI VFQ-25: Near vision, item Q6 [doing work or hobbies that require seeing well up close, such as cooking, sewing, fixing things around the house or using hand tools]; Social vision, item Q11 [seeing how people react to things I say]; Distance vision, item Q14 [going out to see movies, plays or sports events]; Role difficulty, item Q18 [limited in how long I can work or do other activities]; Vision dependency, item Q20 [stay at home]; Mental health, item Q25 [worry about doing things that will embarrass me or others]… We developed a health-state classification based on the NEI VFQ-25 that will allow for the valuation of health preferences for vision-related function and well-being.* |
| Pilling et al. (2005) | Component of social function  Not identified | Not identified | *The impact of nystagmus on visual function (VF; ability to perform activities of daily life) and social function (SF; self perception of wellbeing, interaction with and contribution to society) is unknown… Children’s lifestyles affect their current wellbeing, development, and adult health… The majority of ocular quality of life questionnaires concentrate on tasks and symptoms rather than wellbeing and SF.* |
| Rafaely et al. (2018) | Used synonymously with subjective well-being  Tension  Pressure  Distress  Depression  Mood (feeling cheerful and in good spirits)  Vitality (feeling active and vigorous)  Interest in everyday matters | WHO-5 | *High levels of social support were also found to moderate well-being measures such as tension, pressure, distress, and depression among the elderly population… Subjective well-being World Health Organization (WHO) Well-Being Index This index, developed by the WHO and validated for the older adults population by Bonsignore, Barkow, Jessen, and Heun (2001), evaluates participants’ over-all sense of well-being. The index comprises five statements addressing three aspects of the participant’s feelings over the previous two weeks: Mood (‘I felt cheerful and in good spirits’), vitality (‘I felt active and vigorous’), and interest in everyday matters (‘My daily life was filled with things that interest me’)… For example, we cannot determine whether study participants who reported high SWB felt that way because of their high positive self-rated health or because ranking their health high improves their general sense of well-being.* |
| Pinniger et al. (2013) | Not clear | 25-item NEI-VFQ  SWLS | *To our knowledge, this is the first time tango dance has been employed as a strategy to improve well-being in people with ARMD using a randomised controlled trial design…. In line with the prior relevant literature, we expect that (1) tango dance will be a feasible and acceptable activity for people with ARMD, (2) tango dance participants will show greater reduction in depression levels relative to wait-list controls, and (3) tango dance participants will show greater improvements in self-esteem and SWL relative to wait-list controls.… VFQ-25. This scale has been widely used in ARMD studies (…). It was designed to measure vision-related function and well-being in individuals with ocular disease… SWL scale. This 5-item scale (…) is designed to assess global perceptions of well-being and contentment with life… Self-esteem was assessed using the SES 10 items (…), which has been extensively used to assess self-reported positive and negative attitudes about oneself… GDS–short version. This 15-item scale (…) assesses depressive state in the elderly, with high scores indicating worse depression and scores ranging from 0 to 15* |
| Shah et al. (2020) | Not identified | Not identified | *This is notable since participation in valued social activities can have important health benefits, including increased well-being, decreased anxiety and depression, and even lower rates of dementia and mortality… Understanding the relationship between poor vision and SP is an important step toward promoting health and well-being in visually impaired adults… In addition, the consistent association between poor vision and restricted SP points to an unmet opportunity to develop, evaluate, and implement models of vision rehabilitation that promote SP, which could ultimately improve health, well-being and vision-related quality of life.* |
| Marques-Brocksopp (2012) | Hedonic dimension:  Life satisfaction  Happiness  Eudemonic dimension:  Flourishing  Personal development | Not identified | *One way in which the multiple ‘voices’ of the term may be understood is by framing the concept within a clear two-dimensional framework. The majority of research into wellbeing has concentrated on the ‘life satisfaction’ or ‘hedonic’ dimension – hence claims about people who report frequent happy feelings being less likely to suffer the effects of particular chronic diseases. Life satisfaction and subjective experience is indeed important for understanding the psychological impact of vision loss (e.g. the risk of depression). However, there is a growing number of academics who suggest that looking at this dimension of wellbeing in isolation may not tell the whole story. As noted at a King’s College seminar in early 2011 (Measuring Well-being: A Need or Political Agenda?) and in the wellbeing literature (…), academics are now focusing on a two-dimensional framework of wellbeing which includes both life satisfaction and experiences (‘hedonic’) and a personal development (‘eudemonic’) dimension. A two-dimensional framework not only considers how vision loss affects subjective experiences, but also what conditions are required to develop in a fulfilling way as a functioning individual… Signs of this shift from a one- to a two-dimensional framework of wellbeing are evident in the discourse on distinguishing ‘flourishing’ from ‘happiness’, and the need to consider wellbeing from a holistic standpoint, thus taking into account the needs and personal development of visually impaired individuals physically, socially, emotionally and spiritually.* |
| Zimdars et al. (2012) | Not clear | Not clear | *We are interested in exploring the circumstances of elderly people with regards to their physical and cognitive function, economic position, social networks and well-being… We use the following binary ELSA measures to capture physical functions – mobility difficulties, falls, activities of daily living, instrumental activities of daily living, self-reported health and hearing. The study includes a range of measures of cognitive function, however some of these depended in part on visual acuity. Here we use those measures that did not involve reading or writing skills – animal names recall, word recall and numeracy. For economic circumstances we use measures of income and wealth as well as housing tenure, housing conditions and labour market involvement. Social engagement is estimated by organizational membership, interactions with children, friends and family and feelings of belonging. We operationalize life satisfaction using the eight item CES-D depression scale… When investigating whether there was an independent effect of vision on overall well-being, we found the initial negative effect of poor vision on well-being reduced by the collinear association between poor vision and poor health and economic status. Importantly, the analysis found that reported poor vision appears not to be singled out as a crucial factor triggering depression and low well-being, rather other variables (many of which are associated with visual impairment themselves) were found to be significantly associated.* |
| Heine and Browning (2004) | Not identified | Qualitative interviews | *As well as these personal reactions, broader societal factors impact on well-being, for people with sensory loss experience ‘ stigma’ (…). For example, some participants felt that using a white cane brought them too much attention, and many reported the ridicule of ‘ sighted people’… As a society, we need to provide supportive environments that enhance the independence and well-being of older adults with sensory loss.* |
| Matthews et al. (2017) | Depression  Life satisfaction  QoL  Social engagement  Income  Physical functioning  Health status  Mental health  Risk of mortality | CES-D  SWLS  CASP-19  Binary variable describing participation in organizations, clubs or societies (e.g. political parties, environmental groups, neighbourhood watch groups, religious groups, charitable associations, educational groups or classes, social clubs and exercise classes or gyms  Equivalised weekly income | *Studies have also shown poorer vision to be associated with lower levels of various types of well-being, including physical functioning,5e8 self-reported health,5,9 mental health,5,10,11 an increased risk of mortality12 and reduced social engagement… Depressive symptoms are measured using an eight-point version of the Center for Epidemiologic Studies Depression (CES-D) scale score… Satisfaction with life is measured using the Satisfaction With Life Scale20 and asks the respondent to rate aspects of life satisfaction, such as having achieved important goals and excellence of life conditions, from the response options ranging from ‘strongly agree’ to ‘strongly disagree’ on a seven point Likert scale... Quality of life is measured using the Control, Autonomy, Self-realisation, and Pleasure (CASP) scale... Social engagement is measured using a binary variable describing whether or not the respondent belongs to any organizations, clubs or societies, including political parties, environmental groups, neighbourhood watch groups, religious groups, charitable associations, educational groups or classes, social clubs and exercise classes or gyms. Finally, equivalized weekly income is treated as a continuous variable and is comprised of an individual's total income from employment, pensions, benefits, assets and other sources, adjusted to account for household size… The research presented in this paper demonstrates the importance of understanding the impact of changes in self-reported vision on the psychological, social and financial well-being of older people. Changes in all aspects of well-being are affected to a greater magnitude by deterioration in self-reported vision, and comparison of standardized results suggests that depression and quality of life are most affected… Improvement in self-reported vision is also associated with improvement in some areas of well-being, including satisfaction with life, quality of life and social engagement. Where well-being is adversely impacted by deterioration in self-reported vision, the largest changes are observed among individuals who report a shift from optimal to suboptimal vision. For these people, detrimental effects on depression, satisfaction with life, quality of life and organizational engagement remain significant after controlling for a comprehensive range of socio-economic and health factors* |
| Baker et al. (2020) | Health status  Mobility  Self-care  Usual activities  Discomfort  Anxiety | EQ-5D | *There is an impact of glaucoma on patients general well-being as determined by the EQ-5D and more tellingly on visual function with particular impact on role limitations as determined by the VF25… The first questionnaire was the EQ5D-3L [4]. This is a validated method of measuring general patient utility and overall health status covering the domains of mobility, self-care, usual activities, discomfort and anxiety* |
| Gordon et al. (2011) | Disability-adjusted life years (DALYs):  Disability  Premature death | Total years lost due to disability (YLD, cases * disability weights for mild, moderate, and severe visual impairment) + years of life lost due to premature death (YLL) | *A major cost of VL is the loss of well-being and the quality of life that it entails. Loss of well-being and premature mortality—called the “burden of disease and injury”—were measured in terms of Disability Adjusted Life Years or DALYs according to the method of Murray and Lopez… To calculate the DALYs, disability weights for VL were based on the Dutch weights from the global burden of disease study,4 weighting these numbers by the overall prevalence of mild, moderate and severe VL in Canada... This number was, in turn applied against the prevalence of VL in male and female Canadians separately to get the total years of life lost due to disability (YLD). To this number was added the years of life lost due to premature death (YLL). This number was based on an Australian report by Begg et al.32 on premature death due to VL that was applied against the number of people with VL in Canada. The overall loss of well-being due to VL (total DALYs) was a sum of YLD and YLL.* |
| Cruess et al. (2011) | Disability-adjusted life years (DALYs):  Disability  Premature death | Total years lost due to disability (YLD, cases * disability weights for mild, moderate, and severe visual impairment) + years of life lost due to premature death (YLL) | *The main cost of VL is the loss of well-being and quality of life it entails… This loss of well-being was calculated according to the methodology outlined in The Cost of Vision Loss in Canada: Methodology1 and was found to be 77306 DALYs… Additionally, the value of the lost well-being (disability and premature death, not a financial cost) was a further $11.7 billion.* |
| Taylor et al. (2006) | Disability-adjusted life years (DALYs):  Mortality burden (premature death)  Morbidity burden (disability) | Years of life lost (YLL)  Years of healthy life lost (YLD) | *Loss of wellbeing. People’s suffering and premature death from the disabling and distressing symptoms of disease goes well beyond the financial costs. The overall impact of disease and premature death can be measured as ‘‘burden of disease’’ or ‘‘loss of wellbeing.’’ This estimates disability adjusted life years (DALY). DALY has two components; the years of life lost (YLL) as a result of premature death—the mortality burden; and the years of healthy life lost as a result of disability (YLD)—the morbidity burden.* |
| Lang and Brooks (2015) | Not identified | Qualitative interviews | *This Interpretive Phenomenological Analysis (IPA) study employed semi-structured interviews to explore how four older-age women with sight loss in the United Kingdom experienced and made sense of participating in audio book groups … IPA’s phenomenological, hermeneutic and idiopathic approach offers a methodology congruent with the aim of occupational science to understand how engagement in everyday living can improve health and well-being… The social environment, informed by the cultural, political and economic context in which people live, has significant implications for the way in which occupational identity is maintained or eroded, and the implications for health, well-being and quality of life* |
| Boerner et al. (2006) | Depression  Life satisfaction | 10-item CES-D  SWLS | *Links of occurrence and type of change with well-being were found for the self- and worldview domains… Depression was measured with the 10-item Center for Epidemiological Studies Depression Scale... Life satisfaction was assessed with the 5-item Satisfaction with Life Scale… Regarding which group (i.e., positive, negative, in between, or no change) was doing better in terms of well-being, we found that relationships to well-being outcomes differed by domain and well-being indicator (life satisfaction or depressive symptoms). More significant differences appeared for life satisfaction than depressive symptoms, perhaps because life satisfaction is a more overarching indicator of well-being than depression, which is more affectively based and clinical in nature.* |
| Estcourt et al. (2008) | Not identified | Qualitative interviews | *A qualitative approach allows detailed exploration of patients’ perceptions of wellbeing and can capture their lived experiences… Several participants expressed frustration at the loss of control over issues of personal safety and wellbeing… This first qualitative study in TED provides insight into patients’ perceptions of wellbeing and has identified the phenomenon of an altered identity in patients with TED* |
| Hernandez Trillo and Dickinson (2012) | Depression | WHO-5 | *Personality (BFI-10), religious beliefs (SBI-15), social support (MOS), the mental and physical components of general health (the MCS and PCS of SF-12), well-being (WHO-5), use of magnifiers (MLVQ), understanding of their eye condition and satisfaction with the eye clinic (MLVQ), level of education, and financial status were all considered as predictive of QoL… The factors of age, sex, personality,19 religious beliefs,20 coping strategies,21 social/family support,22 level of education, financial status,23 home circumstances (living alone),24 the patients’ understanding of their condition,25 and depression/ well-being26 were identified as potential contributors to QoL… TABLE 1. Summary of Outcome Measures and Independent Variables (‘‘Contributing Factors’’) Investigated. Contributing Factors (Questionnaires): WHO-529 (well-being/depression)* |
| Glick et al. (2019) | Mental health/Emotional well-being (depression)  Activities of Daily Living (ADLs)  Social participation  Food security  Assets  Per capita household consumption | CES-D  Household Food Insecurity Index (HFIAS, adapted) | *Appendix 1 - Details on the Construction of Selected Indicators of Well-Being. Mental Health: Emotional well-being is measured using an adaptation of the 20 question Center for Epidemiologic Studies Depression Scale (CES-D)… Activities of Daily Living (ADLs). The survey administered standardized questions on the functioning in terms of an individual’s ability to perform unaided a range of Activities of Daily Living10 including personal hygiene and grooming, dressing and undressing, going to the toilet, and feeding… Social Participation: As noted in the text, respondents were asked whether and how often they participated in activities including attending church/mosque, community meetings, visiting friends, going to market, and attending weddings and funerals… Food Security: We measure food security at both the household and individual (blind person) level employing adaptations of the Household Food Insecurity Index11. The HFIAS questionnaire consists of a list of questions about different aspects of food availability for the household or individual during the previous 30 days… Assets: The asset index is a measure of household wealth based on assets owned by the household... Per capita household consumption: This measure is derived from the detailed consumption module of the survey, adapted from the Ethiopia Rural Household Survey (…). The module gathered information on household consumption (though purchases, own production, or gifts) of 32 food items in the last week as well as non-food purchases in the last month. For each item, unit prices were derived from the survey information on units, quantity purchased, and expenditures.* |
| Nastasi (2014) | Not clear | Not clear | *Instruments to collect data included a demographic questionnaire, visual assessments, the Self-report Assessment of Functional Visual Performance, the MOS Social Support Survey, semi-structured interviews, and observations. … An Occupational Life Rich with Well-being. AOTA (2014) describes well-being as “a general term encompassing the total universe of human life domains, including physical, mental, and social aspects” (p. S4). For the participants, their occupational lives embody a life rich with well-being. Support from family and friends enhanced participation and well-being. All of the participants rated their overall quality of life as good and their happiness as an eight or a nine on a scale from one to ten… The support of family and friends allowed the individuals with visual impairment to remain active in the community and to participate in multiple clubs or organizations in the community, which supported their well-being. The participants all reported their overall health and quality of life as fair to good and their emotional well-being between 8-9 on a scale of one to ten. Support from family and friends facilitated their independence in ADL, IADL, leisure, and social participation, which enabled their well-being…* |
| Wahl (2013) | May be used synonymously with subjective well-being  Life satisfaction  Self-acceptance  Positive relations with others  Tone of future-time perspective | Not identified | *4.4. Subjective Well-Being-Related Outcomes, Depression, and Adaptational Processes. Subjective well-being (SWB) is frequently defined via its cognitive component as degree of satisfaction with one’s current life… In general, widening the picture of adaptation to visual impairment with the consideration of everyday competence, robust evidence underscores that differences in well-being-related indicators—including constructs such as self-acceptance, positive relations with others, and tone of future-time perspective between visually impaired and visually unimpaired older adults—are clearly less pronounced than differences in impairment in ADL-IADL status and leisure activity level… The critical role of maintaining everyday competence (and not objective vision impairment) for well-being and adaptation to vision loss in visually impaired older adults has also been confirmed in other research [83], including the transition from assimilative to accommodative coping [19, 20]…In addition to change in self-regulation as a significant adaptational process, social resources have been found to play a critical role in adaptation to vision loss and other well-being-related outcomes—cross-sectionally as well as longitudinally—and family and friends provide distinct contributions to the maintenance of well-being* |
| La Grow et al. (2015) | Not clear | Not clear | *Such measures simply define QOL as the impact of disease, while measures of PQOL provide a much broader assessment of general well-being and social satisfaction that is foundational for understanding successful aging in visually impaired and sighted older adults… Significant differences found on the remaining variables suggest that the visually impaired group is more likely to be single, in poorer health, less economically well off, less satisfied with their ability to perform activities of daily living, less mobile, less satisfied with their lives, and experiencing worse PQOL than the sighted group. The visually impaired group was also found to be significantly lonelier (particularly severely lonely) than the comparison group. Although the effect size for the differences found between the groups was small for loneliness, the effect size for all other significant differences found met or exceeded the “medium” threshold, indicating that visual impairment is significantly and detrimentally associated with older adults’ general well-being… STAGE 2: ASSESSING OLDER VISUALLY IMPAIRED ADULTS’ WELL-BEING BY LEVELS OF LONELINESS In order to assess the relationship between loneliness and well-being in older visually impaired adults, those in this group (n = 315) were divided into three subgroups according to reported loneliness level: not lonely = 147, moderately lonely = 122, and severely lonely = 46… The results in Table 2 indicate that significant differences were found across the groups on all variables except for age, gender, marital status, and living arrangement. Follow-up analysis indicates that ordinal increases in loneliness were directly associated with significant decreases in economic well-being, mental health, satisfaction with activities of daily living, satisfaction with life, and PQOL… Table 2 Comparison of well-being predictor variables among older visually impaired adults stratified by levels of loneliness. Variables: Age, gender, marital status, living arrangement, economic well-being, number of health conditions, physical health, mental health, satisfaction with ADL, ability to get around, life satisfaction, perceived QoL…* |
| Lee et al. (1995) | Not identified | SF-36 | *The association between Snellen visual acuity, Amsler grid distortion and presence of diabetic retinopathy with self-reported functioning and well-being (SF-36) were examined In a sample of 327 diabetics from the Medical Outcomes Study (MOS)… Functioning and well-being was assessed using the SF-36.8'9 The SF-36 measures eight distinct concepts: physical functioning, role limitations due to physical problems, social functioning, bodily pain, emotional well-being, role limitations due to emotional problems, energy and fatigue and general health.* |
| Guerette and Smedema (2011) | Not clear | SWBI | *Subjective measures of quality of life include a sense of well-being, depressive symptoms, and satisfaction with life… Measures of quality of life, specifically social support, well-being, depressive symptoms, and satisfaction with life, have been topics of prior research in the field of visual impairments… In relation to well-being outcomes, greater significant differences were found with life satisfaction than with depressive symptoms in adults with visual impairments… The relationship of perceived social support and well-being variables (depression, life satisfaction, and the five sense of well-being factors identified by Rubin et al., 2003), controlling for demographic characteristics (age and employment) and disability variables, was calculated using hierarchical regression analysis* |
| Schryer et al. (2019) | Life satisfaction  Depression | SWLS | *We also explore the role of public transportation use as a potential moderator of the effects of cessation on the well-being of older adult drivers and their social partners… Life satisfaction. Social contacts and drivers were asked to rate their life satisfaction on the five item Satisfaction With Life Scale (SWLS… Researchers using the SWLS in clinical settings have found that the measure is sufficiently sensitive to identify changes in wellbeing over the course of treatment… One possibility is that life satisfaction is a relatively temporally stable indicator of well-being. Mood indicators such as depression may be more sensitive to fluctuations in emotions associated with life events such as driving cessation… Measures of life satisfaction tell us only about individuals’ cognitive appraisal of their own well-being* |
| Friedman et al. (2003) | Not identified | Self-report | *Results. All subjects reported subjective improvement in well-being, including enhanced effort tolerance following an increase in hematocrit… There were five azotemic diabetic subjects treated for anemia with erythropoietin. All five subjects remarked on substantially improved well-being and greater tolerance for effort as their red cell mass (hematocrit) increased.* |
| Brenner et al. (1993) | Life satisfaction | Cantril Ladder | *The interviews were directed to the impact of vision change on the patient’s quality of life and tapped the following domains: vision-specific information, social functioning, psychological status, and general well-being… General Well-being.—Life satisfaction is often used to represent the general wellbeing of individuals and groups. The Cantril Ladder*' was presented to the patient with the following explanation and question: "This ladder has 10 rungs. Imagine that the top rung is the best possible life that you could be having, given your own situation. Imagine that the bottom rang is the worst possible life you could be having. Where would you put your life right now?"* |
| Wu et al. (2009) | Not clear | 25-item NEI-VFQ | *The NEI-VFQ-25 was added to the study protocol in BISED II to assess vision functioning and well-being, focusing on the high risk groups with open-angle glaucoma, age-related cataract and visual impairment… The NEI-VFQ-25, developed by RAND Corporation and funded by the NEI, is a validated instrument designed to measure vision-targeted health-related QOL (Mangione et al., 2001). The questionnaire contains one question rating general health and generates subscales for the following dimensions: overall vision, difficulties with near and distance vision activities, limitations in social functioning due to vision, role limitations due to vision, dependency on others due to vision, mental health symptoms due to vision, driving difficulties, limitations with peripheral and color vision, and ocular pain… As seen in Figure 1, persons with correctable visual impairment (VA < 20/40) perceived their QOL and well-being as slightly worse than those without visual impairment for all domains, except for general vision.* |
| Ghazi-Nouri et al. (2004) | HRQoL | Vision-related health status questionnaires  Clinical examinations | *METHODS: The National Eye Institute 25-Item Visual Function Questionnaire (VFQ-25) and the 36-Item Short Form Health Survey (SF-36) were self-administered by 30 patients before and 4 months after macular hole surgery. Preoperative, intraoperative, and postoperative clinical data were collected including visual acuity, contrast sensitivity, and metamorphopsia… The use of vision-targeted health status questionnaires in conjunction with detailed clinical examination provides a more comprehensive overview of individuals’ daily well-being after surgical intervention… Traditional clinical measures of vision, such as Snellen visual acuity, may fail to provide important information regarding aspects of visual function that may be vital for patients’ daily performance and well-being.* |
| Das et al. (2018) | Not clear | 25-item NEI-VFQ  Nystagmus-specific quality-of-life questionnaire (NYS-29) | *Our study examines visual functioning of adults with IINS using the National Eye Institute Visual Function Questionairre-25 (VFQ-25)… Participants specifically demonstrated lowest scores for the impact of IINS on mental health, role limitations and dependency... IINS can have a greater than expected impact on an individual’s quality of life, without necessarily causing markedly reduced visual acuity. Our study showed lowest scores in the domains of mental health and wellbeing… Despite reasonable social functioning scores, the most substantial effect of IINS in this study was on mental health, role difficulties, and well-being, to a greater extent than would be expected from the documented level of visual acuity, particularly the mental health subscale… As a result, a nystagmus-specific quality-of-life questionnaire (NYS-29) was developed18, with a different personal and social subscale. This could make it a better tool for assessing the additional domains of wellbeing than other vision-related QoL tools.* |
| Siira et al. (2020) | Not identified | Not identified | *Older adults with VI have indeed been found more dependent in both personal and instrumental daily life activities – such as cutting their toenails, cooking, cleaning, reading, going out in the neighborhood (…) – than healthy controls. Moreover, they have been shown to participate less in society, which is essential for active aging, well-being, and quality of life* |
| Global well-being | | | | |
| Study | **Indicator** | **Measure** | **Quote from article** | |
| Horowitz and Reinhardt (1998) | Used synonymously with subjective well-being  Life satisfaction  Depression | 18-item LSI-A  CES-D | *In lieu of a specific measure of adaptation to late-life vision loss, other researchers have relied on global measures of well-being (such as life satisfaction) or on one or more indicators of psychological status (such as depression, anxiety, and self-esteem)… An assessment of subjective well-being was also included in the interview schedule, measured by the 18-item version (…) of the Life Satisfaction Inventory (LSI-A; …) and depressive symptomatology… Even the highest correlation in this set of results (r = .74 for AVL with CES-D in Study 1) indicates that the two measures share only 55 percent of the total variance. This finding supports the hypothesis that adaptation to a visual impairment is a component of, but not equivalent to, global well-being* | |
| Holistic well-being | | | | |
| Author | **Indicator** | **Measure** | **Quote from article** | |
| Castle et al. (2021) | Not identified | Not identified | *While there is growing evidence of the benefits of arts engagement among both general and military populations, the role of the visual arts in the everyday lives of broader veteran samples, and the impact of these activities on holistic well-being, remains underexplored. The current article highlights the need for art as activity to be differentiated from art as therapy and argues that the former might offer a tool to positively impact the holistic well-being of visually impaired veterans… It remains that the role of visual art activities and their impact on holistic well-being for UK veterans remains largely overlooked… Art as activity may offer benefits to the wider UK veteran population relating to holistic well-being needs, the promotion of good mental health, and the maintenance of active social and leisure lives, but this has not been explored.* | |
| Godier-McBard et al. (2020) | Not identified | WBI | *The aim of this study was to provide an assessment of the holistic well-being of UK blind veterans, utilising a sample of BVUK members, which consisted of primarily older adults… In response to the lack of a standardised measure of holistic well-being that can be used with military veterans, Vogt et al. (2019) developed the Well-Being Inventory (WBI), which has been rigorously validated in six phases with multiple cohorts of US military veterans (…). This set of measures assesses individuals’ status, functioning, and satisfaction with regard to key aspects of their life (referred to as ‘domains’), including their health, vocation, finances, and social relationships… Through its consideration of status, functioning, and satisfaction across these key life domains, the WBI provides a comprehensive assessment of factors that set the stage for well-being, which facilitates the ability to pinpoint areas of both strength and vulnerability within the overall concept of well-being…* | |
| Marques-Brocksopp (2014) | Connection with social, economic, technical, physical and symbolic (language, art, literature, and other cultural ‘symbols’) environments | Not identified | *In their anthropological eco-systemic approach to understanding holistic well-being, they point to connecting not only with the social, ‘human-made’ environment, as suggested by the WHO, or only a social, economic, and technical environment, as suggested by Capra (1982), but also with a physical environment, including other living creatures and a symbolic dimension representing language, art, literature, and other cultural ‘symbols* | |
| Optimal well-being | | | | |
| Study | **Indicator** | **Measure** | **Quote from article** | |
| Bambara et al. (2009) | Not identified | Not identified | *Thus, a greater understanding of family members’ experiences is needed to promote the optimal well-being and successful adjustment of both individuals with low vision and their family members… In a related study by Cimarolli and Boerner (2005), persons with low vision who perceived that they were receiving only overprotective support reported less optimal well-being than did those who perceived that they were receiving more positive types of support.* | |
| Positive well-being | | | | |
| Study | **Indicator** | **Measure** | **Quote from article** | |
| Gleeson et al. (2017) | Not identified | Not identified | *Physical activity in old age can reduce the onset of disabilities and illness, as well as maintain independence and foster positive well-being… Gait speed is an objective measure of mobility and faster gait speeds were associated with fewer depressive symptoms. Mobility is a key factor in maintaining positive well-being into old age, as it provides a means of freedom, independence and quality of life.* | |
| Weber and Wong (2010) | Not identified | Qualitative interviews | *OPTIMISM AND COMPENSATION. Participants that expressed optimism also tended to score higher on the AVL scale and reflected more positive well-being* | |
| Mitchell and Bradley (2001) | Happiness  Satisfaction with personal life  Have lived the kind of life I wanted to  Feeling eager to tackle my daily tasks or make new decisions  Feeling able to deal with problem or major change in life | W-BQ12  W-BQ22 | *This paper reports the evaluation of a generic measure of psychological well-being, the 12-item Well-Being Questionnaire (W-BQ12), for use with people who have the chronic eye condition, macular disease (MD)… … The original scale developed contained 22 items (W-BQ22) and had a four-factor structure with subscales measuring depression, anxiety, energy and positive well-being… Work on a Japanese translation of the W-BQ22 led to development of a 12-item version with three subscales: negative well-being (NWB), energy and positive well-being (PWB)… All items intended for the 4-item NWB subscale were negatively worded and all items intended for the 4-item PWB subscale were positively worded… Table 1. W-BQ12 items and factor analysis rotated component matrix. Pos 1 I have been happy, satisfied or pleased with my personal life, Pos 2 I have lived the kind of life I wanted to, Pos 3 I have felt eager to tackle my daily tasks or make new decisions, Pos 4 I have felt I could easily handle or cope with any serious problem or major change in my life* | |
| Mitchell and Bradley (2006) | Component of psychological well-being  Vitality/energy  Enthusiasm for life | SF-36 Vitality subscale  W-BQ12 | *Measures which also investigate positive well-being (e.g. the well-being scales within the SF-36 measure vitality [14]) and particularly those which measure positive well-being with items concerned with enthusiasm for life (e.g. the Well-being Questionnaire (W-BQ12) which measures energy and positive well-being as well as anxiety and depression [15]) are more likely to detect improvement in psychological well-being* | |
| Negative well-being | | | | |
| Study | **Indicator** | **Measure** | **Quote from article** | |
| Harada et al. (2008) | Depression  Health status  Functional ability  Disability  Self-sufficiency | 5-item GDS  Self-rated health status  Tokyo Metropolitan Institute of Gerontology Index of Competence (TMIG-IC) | *Recent evidence that vision and hearing impairments are related to negative well-being, including depression, disability, reduced self-sufficiency, and diminished physical function, highlights the importance of studying age-related declines in sensory functions among elderly people… We evaluated three negative well-being measures through the survey and the medical assessments: depression; self-perceived poor health; and reduced functional activity… Depressive symptoms were evaluated with a five-item version of the Geriatric Depression Scale (GDS5)… Participants who ranked their health status as “poor” or “very poor” (rather than “very good”, “good”, or “fair”) were considered to be in poor health. Functional activity was evaluated according to the Tokyo Metropolitan Institute of Gerontology Index of Competence (TMIG-IC)* | |
| Mitchell and Bradley (2006) | Anxiety  Depression | HADS  W-BQ12 | *Some well-being scales, such as the Hospital Anxiety and Depression Scale (HADS) [13] measure only negative well-being (anxiety and depression).* | |
| Mitchell and Bradley (2001) | Tearfulness  Feeling downhearted and blue  Fear (feeling afraid for no reason at all)  Upset  Feeling panicky | W-BQ12  W-BQ22 | *This paper reports the evaluation of a generic measure of psychological well-being, the 12-item Well-Being Questionnaire (W-BQ12), for use with people who have the chronic eye condition, macular disease (MD)… The original scale developed contained 22 items (W-BQ22) and had a four-factor structure with subscales measuring depression, anxiety, energy and positive well-being… Work on a Japanese translation of the W-BQ22 led to development of a 12-item version with three subscales: negative well-being (NWB), energy and positive well-being (PWB)… Table 1. W-BQ12 items and factor analysis rotated component matrix. Neg 1 I have crying spells or feel like it, Neg 2 I feel downhearted and blue, Neg 3 I feel afraid for no reason at all, Neg 4 I get upset easily or feel panicky* | |
| Subjective well-being | | | | |
| Study | **Indicator** | **Measure** | **Quote** | |
| Waisbourd et al. (2015) | QoL | 25-item NEI-VFQ  Modified Glaucoma Symptom Scale (MGSS)H | *There are three distinct approaches to measuring the impact of glaucoma on individuals’ lives: 1) clinical measures (for example, visual acuity (VA), contrast sensitivity (CS), and visual field (VF)), 2) self-reported measurements of subjective well-being (QoL), and 3) performance-based assessments of the ability to carry out daily activities… The current study aims to investigate the complex relationships between clinical measures (e.g., VA, IOP, VF, OCT and CS tests), performance-based measures (assessed by the CAARV), and subjective self-reported measures (NEI-VFQ-25 and MGSS).* | |
| McManus and Lord (2012) | Happiness  General well-being (depression, self-confidence, self-worth)  Life satisfaction  Health satisfaction  Income satisfaction  Leisure time satisfaction  Job satisfaction  Mental well-being (mental health: feeling optimistic about the future, feeling useful, feeling relaxed, dealing with problems well, thinking clearly, feeling close to others, able to make up own mind) | GHQ-12  SWEMWBS | From report page 16: *3 Subjective well-being. 3.1 General well-being and happiness. Table 3.1 Felt unhappy or depressed recently (GHQ 12 item - a_scghqi), Table 3.2 Been feeling reasonably happy recently (GHQ 12 item - a_scghql), Table 3.3 Been losing confidence in self recently (GHQ 12 item - a_scghqj), Table 3.4 Been thinking of self as a worthless person recently (GHQ 12 item - a_scghqk), 3.2 Satisfaction with domains of life. Table 3.5 Satisfaction with health, Table 3.6 Satisfaction with income, Table 3.7 Satisfaction with leisure time, Table 3.8 Satisfaction with job among employed people scored 1 to 7, Table 3.9 Satisfaction with life overall, 3.3 The Short Warwick-Edinburgh Mental Wellbeing Scale (SWEMWBS). The Warwick Edinburgh Mental Wellbeing Scale2 (WEMWBS) was developed in recent years for assessing positive mental health (mental well-being)… It covers most aspects of positive mental health (positive thoughts and feelings) currently in the literature, including both hedonic and eudaimonic perspectives… Table 3.10 Feeling optimistic about the future, Table 3.11 Feeling useful, Table 3.12 Feeling relaxed, Table 3.13 Dealing with problems well, Table 3.14 Thinking clearly, Table 3.15 Feeling close to others, Table 3.16 Able to make up own mind* | |
| Wettstein et al. (2015) | Life satisfaction (cognitive well-being)  Positive affect (affective well-being)  Negative affect (affective well-being) | SWLS  PANAS | *In this study, we investigate the relationship between the domains of cognitive abilities and well-being in a sample of adults in advanced old age. By assessing well-being, we followed the established multidimensional conception of subjective well-being (…), which is composed of cognitive (i.e., life satisfaction) and affective (i.e., positive and negative affect) aspects… Cognitive well-being was assessed with the Satisfaction with Life Scale (SWLS;… The components of affective well-being, positive affect and negative affect, were assessed with the Positive and Negative Affect Schedule (PANAS…* | |
| Renaud and Bedard (2013) | QoL  Life satisfaction  Well-being in relation to goals, expectations, concerns, values, and priorities  Achievements | LSI-A  Quality of Life Index | *QOL as subjective well-being, which will be referred to as subjective QOL in this paper, represents mostly the sum of the cognitive and emotional reactions describing the person’s satisfaction with life and well-being in relation to his/her goals, expectations, concerns, values, and priorities. Subjective QOL evaluation depends mainly on the congruence or discrepancy between the person’s achievements and expectations. It tends to reflect a more global aspect of QOL. The instruments used to assess subjective QOL aim to understand the way individuals perceive their condition according to their values and expectations… Only two papers in this review investigated the relationship between depressive symptoms and subjective QOL.14,50 One used the Life Satisfaction Index-A59 and the other used the Quality of Life Index* | |
| Erickson et al. (2004) | Component of HRQoL  Happiness  Contentment  Self-assurance  Self-confidence  Sleep  Optimism  Pessimism | Multidimensional Quality-of-Life Scale for Myopia | *Five dimensions of health-related quality of life in myopia were hypothesized to affect satisfaction with visual correction modality. Items on these dimensions reflected the frequency of visual compromise and ocular symptoms; individual tolerance of these compromises and symptoms; cosmesis; psychological constructs (including situation-dependent characteristics such as adaptability, self-efficacy, and subjective well-being); and personality traits such as extraversion and introversion.… TABLE 2. Factor analysis of the final iteration of the multidimensional vision quality-of-life scale. Psychological states (health proneness): Happy and content (subjective well-being), Self-assured and self-confident (subjective well-being), Sleep well at night (Subjective well-being)… For example, subjective well-being encompasses the sense of feeling positive or negative or optimistic or pessimistic about a situation being experienced.* | |
| Acton et al. (2016a) | Not identified | WEMWBS | *Secondary outcome measures were: the Patient Health Questionnaire (PHQ-9), an assessment of depression symptom severity20; the Warwick-Edinburgh Mental Well-being Scale (WEMWBS), a population measure of subjective wellbeing* | |
| Acton et al. (2016b) | Positive thoughts  Positive feelings | WEMWBS | *Secondary outcome measures will include: the Patient Health Questionnaire (PHQ-9), an assessment of depression symptom severity on a nine-item scale, based on criteria for depressive episodes including concentration problems and suicide [22]; the Warwick-Edinburgh Mental Well-being Scale (WEMWBS), a population measure of subjective well-being, involving 14 positively worded questions about aspects of positive thoughts and feelings [23];* | |
| Kekecs et al. (2014) | Happiness  Discomfort | Pain Affect Faces Scale | *The groups did not differ in sleep quality before the day of the operation, heart rate during the procedure, and subjective well-being… Subjective well-being was measured using the Pain Affect Faces Scale [35] at the measurement points: First meeting, Before surgery, After surgery and Postoperative visit. The Faces Scale is a visual scale where a series of 9 schematic line drawn faces are presented to the patient. These faces show different levels of happiness or discomfort from which the patient chooses the one that best represents his current mood. The responses were coded on a 9 point scale, 1 meaning the worst, 9 the best Well-being.* | |
| Burmedi et al. (2002) | Used synonymously with general well-being  Life satisfaction  Happiness  Contentment  Self-esteem  Morale  Mood  Psychological distress  Trait anxiety  Negative affect  Outlook on life | AVL  ABS  LSI-A  LSI-W  LSES  Mental Health Inventory (MHI) Psychiatric Epidemiology Research Interview (PERI)  PGCM  POMS  Revised Feelings of Inadequacy Scale (RFIS) State-Trait Anxiety Inventory (STAI)  Quality of Well-Being (QWB) | *With the concept of emotional adjustment, we target work concerned with depression and other mental disturbance on the one hand and subjective well-being-oriented research on the other… Our second hypothesis is that this link will be less clear and consistent when it comes to well-being, a second major expression of emotional adjustment. This is based on the fact that, in contrast to depression, well-being-related constructs and measures are quite diverse, covering concepts such as life satisfaction, morale, and mood, and involving either full-length scales or only one-item assessments… Well-being. In the following, we define well-being in its broadest terms. Hence, research linking vision loss in later life to life satisfaction, happiness, self-esteem, mood, and morale were integrated into this literature review… A vision-related measure of well-being, the Adaptation to Vision Loss scale,76 also showed a moderate correlation with the degree of visual impairment.8 In a few isolated cases, this pattern of results was not observed. Upton et al.,26 for example, did not find any evidence that psychological distress among the visually impaired was higher than those living in the community. Quite unexpectedly, Sinzato et al.61 found the trait anxiety to be significantly lower among diabetics with retinopathy than among diabetics without... vision loss appears to be accompanied by negative affect,62,63 unhappiness, and discontentment… Table 3. Well-being among elders with visual impairment. Multi-item measures of well-being: PGMS, LSI-W, RFIS, Factors from MHI & PERI, STAI, LSES, LSI-A, ABS, PMS, QWB, One-item measures of well-being: Morale, Negative feelings, Happiness, Feelings, Outlook on life, Mood, ABS, Affect Balance Scale;65 LSI-A, Life Satisfaction Index – A;66 LSI-W, Life Satisfaction Index – Well-being;67 LSES, Life Satisfaction in the Elderly Scale;68 MHI, Mental Health Inventory;69 PERI, Psychiatric Epidemiology Research Interview;70 PGMS, Philadelphia Geriatric Morale Scale;71 PMS, Profile of Mood States;72 RFIS, Revised Feelings of Inadequacy Scale;73 STAI, State-Trait Anxiety Inventory;74 QWB, Quality of Well-Being* | |
| Barr et al. (2012) | Feelings about oneself  Feelings about the future | CORE-OM | *The CORE-OM was designed to be suitable for use across a wide variety of client groups and service types. It taps into a ‘core’ of clients’ distress, including subjective wellbeing, commonly experienced problems or symptoms, and life/social functioning… The specific domains of psychological functioning found to present the greatest concern for this sample of people with visual impairment were wellbeing (feelings about oneself and the future) and problems (depression, anxiety, physical health, and trauma).* | |
| Heyl and Wahl (2014) | Not identified | Not identified | *Subjective Well Being–related Outcomes and Depression: Visually impaired older adults have shown evidence of diminished well-being as compared with sensory unimpaired older adults [53], although effect sizes were rather small in a respective meta-analysis [54]…. At the same time, it is critical to acknowledge that visually impaired older adults represent an at-risk population, in which the positive impact of human adaptation and the drawback of reaching the limits of psychological resilience go hand in hand. Affect balance (ratio of positive and negative affect) has been found to be more toward the negative pole in visually impaired older adults [60] and depression has consistently been found to be significantly increased in visually impaired older adults* | |
| Haibach-Beach et al. (2020) | Life satisfaction | SWLS | *Thus, the aim of this study was to examine the effectiveness of a home-based balance intervention upon gait, balance, and well-being with older adults with visual impairments... The Satisfaction with Life Scale (…) is a brief assessment of an individual’s sense of their entire life that has been used in hundreds of studies to examine subjective well-being.* | |
| Horowitz and Reinhardt (1998) | Life satisfaction  Depression  Morale | 18-item LSI-A  CES-D | *In gerontological research, general measures of subjective well-being, a term that is often used interchangeably with morale and life satisfaction, focus on older people's current emotional state and on the congruence between desired and achieved lifetime goals… An assessment of subjective well-being was also included in the interview schedule, measured by the 18-item version (…) of the Life Satisfaction Inventory (LSI-A; …) and depressive symptomatology, measured by the Center for Epidemiological Studies Depression Scale (CES-D; …)* | |
| Madsen et al. (2021) | Not identified | WHO-5 | *In the 12 persons who completed the 6 weeks of treatment, the post-treatment depression score was reduced (p < 0.001), and subjective wellbeing (p = 0.01) and sleep quality were improved (p = 0.03)… Table 3. Self-reported mood, wellbeing and sleep at inclusion, mid-treatment and end of treatment. Self-reported outcomes from the Hamilton Depression Rating Scale, 6-item self-report version (Ham-D6), the WHO-5 wellbeing Index (WHO-5), sleep quality rating from 0 to 10 (worst to best).* | |
| Glen and Crabb (2015) | Not identified | Not identified | *Cultural influences are also likely to impact subjective wellbeing and how people adapt to a condition [38]* | |
| Schilling and Wahl (2006) | Life satisfaction  Positive affect  Negative affect | SWLS  PANAS | *Adaptation of subjective well-being (SWB) in later life has become an important topic in gerontological research in the past decade, prompted by the absence of age-related decline in SWB as reported by numerous empirical studies and literature reviews… However, these findings concern life satisfaction, which in well-being research is seen as the cognitive component of SWB, meaning the evaluation of one’s own life or living circumstances in terms of subjective judgments, which must be distinguished from the person’s emotional experiences (…). With respect to the affective component of SWB, typically operationalized as positive affect (PA) and negative affect (NA), reviews of the literature on age-related changes in PA and NA have revealed mixed findings* | |
| Alma et al. (2011) | Not identified | Not identified | *This is an important finding since participation in society can be considered as an indicator of successful aging [54] and has a positive influence on physical and mental health [55], quality of life [56] and subjective well-being [54]* | |
| De La Jara et al. (2010) | Component of QoL  Optimism (when facing a particular situation)  Pessimism (when facing a particular situation) | Health proneness questionnaire (HPQ) | *Psychological state refers to how a patient adjusts to different situations and includes subjective well-being, self-efficacy and adaptability. Subjective well-being describes how optimistic or pessimistic a person feels when facing a particular situation… The IER QOL Scale assesses physical status, psychological state, personality traits and cosmesis, which are dimensions associated with a patient's QOL... This multidimensional scale comprises 26 vision-oriented items (13 items related to frequency of disturbing visual and ocular symptoms, 13 items corresponding to tolerance of visual and ocular symptoms), 13 psychologically and personality trait-oriented items (health proneness questionnaire (HPQ) which includes 10 items related to adaptability, self-efficacy and subjective well-being and three personality trait items related to extraversion/introversion) and three items measuring cosmesis.* | |
| Bergeron and Wanet-Defalque (2013) | Used synonymously with general well-being and affective well-being  Life satisfaction  Negative affect  Positive affect | SWLS  PANAS | *The aim of this study was to explore the pattern of adaptation to visual impairment in terms of denial, acceptance, well-being, and depression, among patients with varying lengths of time since diagnosis… In one case, psychological adaptation to age-related macular degeneration (AMD) was studied in terms of subjective well-being (i.e. positive and negative affects; …). In Schilling and Wahl’s study, 90 seniors with AMD were followed for a 1-year period, during which five measurement points of affective well-being (measured by the Positive and Negative Affect Schedules [PANAS]) were conducted at 3-month intervals… All participants answered questions in the same order: general demographics, coping strategies (BC; …), depressive symptoms (the CES-D–Short Form; …), and well-being (Satisfaction with Life Scale; …). … The Satisfaction with Life Scale (…) is a 5-item questionnaire measuring subjective well-being, which is described as ‘the personal ability to enjoy oneself and one’s own life’* | |
| Mirandola et al. (2019) | Component of QoL  Not identified | SF-12 (Italian version) | *The Italian version of the SF-12 questionnaire consists of 2 components: a physical component score and a mental component score. Higher scores on these subscales indicate greater levels of functioning and a more favorable health status, thus being indicative of a better QoL [27]... Our data on QoL, which measures the perceived physical and mental health levels as well as the overall subjective well-being, seem also to support this hypothesis.* | |
| Pinquart and Pfeiffer (2011) | Not clear | Not identified | *Studies were identified from the literature through electronic databases [PSYCINFO; MEDLINE; CINAHL; EMBASE – search terms: (visual impairment or blindness or low vision or glaucoma or cataract or diabetic retinopathy or retinitis or AMD) and (PWB or subjective well-being or psychological health or mental health or quality of life or depression or anxiety or loneliness or positive affect or life satisfaction or self-concept or self-esteem)], and cross-referencing.* | |
| Wahl et al. (1999) | Nonagitation  Satisfaction with the aging process  Life satisfaction  Future orientation | PGCM | *As a measure of emotional adaptation, we used the well-known Philadelphia Geriatric Center Morale Scale (…) in its slightly revised version as used in the Berlin Aging Study (…). In particular, we omitted two items from the original questionnaire and used a 5-point rating scale (from "strongly agree" to "completely disagree") as a response format. According to Lawton (1975), a total score as a proxy for overall subjective well-being as well as three subscores (nonagitation, satisfaction with the aging process, general life satisfaction) can be calculated.* | |
| Kahaly et al. (2002) | Not identified | Not identified | *Subjective well-being as well as the physical and mental ability to function in everyday life became the focus of attention, not the least of which with regard to the use of medical intervention as well.* | |
| Kaleemunnisha et al. (2014) | Component of VRQoL/HRQoL General health  Psychological well-being (social functioning, mental health, role difficulties, dependency) | 25-item NEI-VFQ | *Subjective well-being of uveitis patients. Schiffman et al. described vision and health related quality of life in 76 patients with various types of uveitis and concluded that uveitis has a wide effect on vision and health related quality of life (QOL) of the individual… Vision related quality of life (VR-QOL) was evaluated using the National Eye Institute Visual Functioning Questionnaire (NEI VFQ-25)… The psychological well-being and general health subscales showed significant improvement in the mean score following immunosuppressive therapy… The four components of psychological well being-social functioning, mental health, role difficulties, dependency showed moderate to large improvement in the sub-scale scores similar to other studies.* | |
| Yawson et al. (2014) | Life satisfaction | Single item Satisfaction with life overall (WHO Disability Assessment Schedule) | *Subjective wellbeing (SWB). Well-being or life satisfaction was assessed through a multi-dimensional scale, the WHO Disability Assessment Schedule 2.0, including a question about satisfaction with life overall [17, 18]. SWB as a single item measure was based on the overall life satisfaction question.* | |
| Xiang et al. (2020) | Positive and negative emotions (feeling cheerful, bored, full of life, upset)  Self-realization (purpose in life, self-acceptance, environmental mastery)  Symptomology (e.g. depressive symptoms)  Hedonic characteristics (e.g. happiness)  Eudemonic characteristics (e.g. flourishing) | NHATS items | *Conceptualization of SWB has also evolved from a relatively limited focus on symptomology (eg, depressive symptoms) to recognition of the importance of both hedonic (eg, happiness) and eudaimonic (eg, flourishing) characteristics... Subjective well-being: NHATS includes four items reflecting positive and negative emotions (frequency of feeling cheerful, bored, full of life, or upset in the last month on a five-point Likert scale) and three items reflecting self-realization (extent of disagreement with statements about purpose in life, self-acceptance, and environmental mastery on a 3-point Likert scale)* | |
| Bazargan et al. (2001) | Used synonymously with psychological well-being  Component of QoL  Not identified | 17-item PGCM | *Subjective well-being is important as a psychological summary of the quality of an individual’s life in society. Several social psychological concepts tap aspects of quality of life indirectly, such as self-esteem, depression, anxiety, and alienation, but only subjective well-being and happiness have a “bottom-line” finality in terms of consequences for individuals... Subjective well-being. The dependent variable, subjective well-being, was measured by the 17-item revised version of The Philadelphia Geriatric Center (PGC) Morale Scale... Higher scores in this index represent a higher level of psychological well-being.* | |
| Elliott et al. (2005) | Life satisfaction | SWLS | *Life satisfaction. The Satisfaction With Life Scale (SWLS;…) was used to evaluate subjective well-being and overall life satisfaction. The SWLS is a 5-item instrument with items rated on a Likert-type response format ranging from 1 (strongly disagree) to 7 (strongly agree). Higher scores reflect greater subjective well-being.* | |
| Silverman and Cohen (2014) | Life satisfaction  Stress | SWLS  Stress Scale | *Subjective well-being. Participants completed two validated scales of well-being: a Life Satisfaction Scale (…) and a Stress Scale (…).* | |
| Rafaely et al. (2018) | Mood (feeling cheerful and in good spirits)  Vitality (feeling active and vigorous)  Interest in everyday matters (daily life is filled with things that interest me)  Life satisfaction  Affect balance  Contentment  Sadness | WHO-5 | *The likelihood of vision impairment rises with age (…), tends to threaten everyday functional competence (…), tends to be associated with depression symptoms and lower level of life satisfaction (…) and consequently affects people’s subjective well-being (SWB) (…). This concept reflects one’s personal evaluation and feelings concerning life, manifested at the cognitive-intellectual level – at which people judge their lives in general and at the emotional level, wherein they report of the relative prominence of positive affects over negative ones in the present or the recent past (…). SWB is characterized by over-all contentment, as contrasted with feelings of sadness and despair… Subjective well-being. World Health Organization (WHO) Well-Being Index. This index, developed by the WHO and validated for the older adults population by Bonsignore, Barkow, Jessen, and Heun (2001), evaluates participants’ over-all sense of well-being. The index comprises five statements addressing three aspects of the participant’s feelings over the previous two weeks: Mood (‘I felt cheerful and in good spirits’), vitality (‘I felt active and vigorous’), and interest in everyday matters (‘My daily life was filled with things that interest me’).* | |
| Silverman (2015) | Life satisfaction  Stress | SWLS  Stress Scale | *Subjective well-being. Participants completed two validated scales of well-being: a Life Satisfaction Scale (…) and a Stress Scale (…).* | |
| Wahl (2013) | May be used synonymously with general well-being  Life satisfaction  Self-acceptance  Positive relations with others  Tone of future-time perspective | Not identified | *4.4. Subjective Well-Being-Related Outcomes, Depression, and Adaptational Processes. Subjective well-being (SWB) is frequently defined via its cognitive component as degree of satisfaction with one’s current life… In general, widening the picture of adaptation to visual impairment with the consideration of everyday competence, robust evidence underscores that differences in well-being-related indicators—including constructs such as self-acceptance, positive relations with others, and tone of future-time perspective between visually impaired and visually unimpaired older adults—are clearly less pronounced than differences in impairment in ADL-IADL status and leisure activity level [36, 77]… The critical role of maintaining everyday competence (and not objective vision impairment) for well-being and adaptation to vision loss in visually impaired older adults has also been confirmed in other research [83], including the transition from assimilative to accommodative coping [19, 20]…In addition to change in self-regulation as a significant adaptational process, social resources have been found to play a critical role in adaptation to vision loss and other well-being-related outcomes—cross-sectionally as well as longitudinally—and family and friends provide distinct contributions to the maintenance of well-being [62, 85]. Perceived overprotection may also lead to negative consequences in terms of heightened depression and anxiety over time [73].* | |
| Smedema and McKenzie (2010) | Physical well-being and associated feelings about self  Psychological well-being  Family and social well-being  Financial well-being  Medical care | SWBI | *Purpose. To determine the relationship among amount and type of internet use and perceived social support and subjective well-being in persons with visual impairments… The participants completed a survey containing a demographic questionnaire, an internet use questionnaire, the personal resources questionnaire – 2000 (PRQ-2000), and the sense of well-being inventory (SWBI)… The SWBI is a subjective well-being measure developed specifically for people with disabilities… The SWBI consists of five subscales: physical well-being and associated feelings about self, psychological well-being, family and social well-being, financial well-being, and medical care.* | |
| Toyoshima et al. (2018) | Depression  Loneliness | GDS Short Form  10-item UCLA Loneliness Scale | *The primary purpose of these studies was to identify characteristics related to subjective well-being (SWB) and understand how oldest-old adults maintain their SWB even following decreases in physical, cognitive, and sensory functioning with aging… Subjective well-being. SWB was examined in terms of depression and loneliness. Depression was assessed using the Geriatric Depression Scale Short Form (GDS; …), and loneliness was measured using 10 items of the UCLA Loneliness Scale* | |
| Guerette and Smedema (2011) | Depression  Life satisfaction  Physical well-being and associated feelings about self  Psychological well-being  Financial well-being  Family and social well-being  Medical care/well-being | CES-D-10  SWLS  SWBI | *Subjective well-being was measured via the outcome variables from Cimarolli and Boerner’s study (depressive symptoms and satisfaction with life), along with five additional sense of well-being factors: physical well-being, psychological well-being, financial well-being, family or social well-being, and medical well-being… The CES-D-10 (…) is a measure of depression in which participants indicate how often in the previous week they experienced each of 10 symptoms (such as “I felt depressed”)... The SWLS (…) is a measure of global life satisfaction in which individuals use a 7-point Likert scale (from 1 = strongly disagree to 7 = strongly agree) to indicate the extent to which they agree with five items (for example, “In most ways my life is close to ideal”)… Subjective well-being was measured by the SWBI... The five subscales of the SWBI are physical well-being and associated feelings about self, psychological well-being, financial well-being, family and social well-being, and medical care* | |
| Van Boemel and Rozee (1992) | Family and social relationships  Ability to perform expected tasks  Spiritual life  Ability to participate in society  Adjustment to living in foreign country | Well-being inventory | *The exit interview consisted of portions of the entrance questionnaire, including the psychosomatic checklist, the sections relating to adjustment in the United States, and the well-being inventory… Subjective Well-Being. In our attempt to better understand the level to which these women's lives could be subjectively improved, we addressed the issue of subjective well-being for this sample. Subjective well-being was a combined score from a multi-item scale, which was operationalized as how the respondent felt about her family and social relationships, her ability to perform her expected tasks, her spiritual life, her ability to participate in American society, and her adjustment to living in the United States.* | |
| Liu et al. (2016) | Life satisfaction  Positive affect  Negative affect  Affect Balance | LSI-A  ABS | *Our objective was to examine the associations of visual and hearing impairment with subjective well-being (SWB), an important psychological concept defined by life satisfaction [LS], positive affect [PA], negative affect [NA], and affect balance [AB] among long-lived individuals (LLIs) over 95 years of age… SWB was assessed using two scales, the Life Satisfaction Index A (LSIA) (…) and the Bradburn’s Affect Balance Scale (ABS) (…) as described elsewhere* | |
| Siira et al. (2020) | Not identified | Not identified | *The subjective well-being of elderly people warrants attention because it may play a protective role and help maintain health and quality of life while aging* | |
| Personal well-being | | | | |
| Study | **Indicator** | **Measure** | **Quote** | |
| Freitas et al. (1995) | Mood  Affect | Cantril Self-Anchoring Striving Scale | *The measurements include the following variables: clinical end points (spherical equivalent refraction); visual function (night driving, day driving, near vision, far vision, glare disability); functional status (physical, social, and role functioning, mental status); general well-being (health perceptions, personal well-being, overall quality of life)… Personal well-being: The Cantril Self-Anchoring Striving Scale was presented to the patient with the following explanation and question: “This ladder has 10 rungs. Imagine that the top rung is the best possible general mood or affect that you could have, and that the bottom rung is the worst. Where would you put your personal well-being right now?”* | |
| Dubey et al. (2020) | Personal safety  Personal care  Leisure activities | Social function scale (SFS) | *Individual items on the social function questionnaire were grouped under two broad categories to reflect on the primary aspects of functional performance: 1. Personal wellbeing (personal safety, personal care, leisure activities) and 2. Social wellbeing (fulfill responsibilities; interact with the world, social interaction).* | |
| McCormack (2021) | Not identified | Qualitative interviews | *For example, the representative of an organization serving an older blind population shared such an observation as it related to Hurricane Maria recovery: Those that had received independent living skills prior to the storm were able to manage better and were in a more relaxed state in their homes because they already had that training of where things were located in their home, how to access things, their own personal wellbeing, and that their homes were very well organized so that they were able to really maneuver within their home environment in a way that felt safe.* | |
| Béchetoille et al. (2008) | Component of QoL  Not identified | Not identified | *Spilker (1990) suggested a hierarchical QoL model with three levels, the first of which corresponds to the overall assessment of QoL as proposed by the World Health Organization Quality of Life (WHOQoL) group, defined as ‘an individual’s overall satisfaction with life, and one’s general sense of personal wellbeing’.* | |
| Individual well-being | | | | |
| Study | **Indicator** | **Measure** | **Quote** | |
| Naylor and Labbe (2017) | Not identified | ORS | *The ORS (…) is a four-item ultra-brief measure given to participants to monitor their individual progress from session to session... Traditionally, participants are to mark on a line with one end being 0 and the other being 10, indicating how the participants’ feel that area of their life has been for them over the course of the week (i.e. individual well-being, interpersonal well-being, social well-being, and general well-being)* | |
| Psychological well-being | | | | |
| Study | **Indicator** | **Measure** | **Quote** | |
| Riazi et al. (2014) | Used synonymously with mental well-being  Mental health | WEMWBS | *Our primary outcome measure is psychological well-being, measured by the Warwick-Edinburgh Mental Well-being Scale (WEMWBS). The WEMWBS is a scale for assessing positive mental health (mental well-being)... It covers most aspects of positive mental health (positive thoughts and feelings) currently in the literature, including both hedonic and eudaimonic perspectives.* | |
| Salminen et al. (2019) | Not identified | Not identified | *Group meetings in an institutional setting were arranged in four modules. The first module (five days) dealt with themes of interpersonal skills and life management. At this phase, a great deal of effort was put into building group cohesion among participants. The themes of the second period (two days) were social and psychological well-being, sexuality and self-determination, leisure time, and hobbies.* | |
| Godier-McBard et al. (2020) | Not clear | Not identified | *Rather, it has highlighted poor mental health and psychological well-being in working-age visually impaired veterans… Subsequently, three papers have been published investigating psychological well-being in blind veterans (…). One of these papers reported relatively high rates of mental health problems and hazardous drinking in blind veterans (…). An adverse impact on a number of daily life domains was also reported in relation to sight loss, resulting in financial hardship and strain on family relationships, loss of confidence, and a negative impact on identity (…). Blind veterans have reported using a number of strategies to cope with sight loss, including both adaptive (e.g., using low visions aids or downwards social comparison, whereby individuals compare themselves to others who are worse off than themselves) and maladaptive behaviours (e.g., substance misuse, social withdrawal)* | |
| Lindo and Nordholm (1999) | Mood (pleasantness, activity, relaxation, extroversion, social orientation, confidence) | MACL | *Psychological well-being was assessed by the MACL, which consists of 71 adjectives describing various moods, such as insecure, content, elated, nervous, irritable, indifferent, and happy. The respondent indicates how he or she feels at the time of the assessment ... The adjectives of the MACL are organized into six bipolar dimensions: pleasantness-unpleasantness (hedonistic tone), activation-deactivation (activity), calmness-tension (relaxation), extroversion-introversion (extroversion), positive-negative social orientation (social orientation), and confidence-lack of confidence (confidence).* | |
| Scuderi et al. (2011) | Hopelessness  Depression  Suicidal history  Emotional well-being | Beck Hopelessness scale  Gotland Male Depression scale  Suicidal History Self-Rating Screening Scale  Perceived Disability Questionnaire  Emotional Well-being Scale | *Thus, the aims of the study were to study: (i) affective temperaments in OAG patients with some degree of functional visual impairment; (ii) psychological well-being and perceived disability, and their associations with affective temperaments; and (iii) associations between visual impairment, affective temperaments and psychological well-being. As measures of well-being, we administered rating scales measuring hopelessness, suicidality and depression.* *Table 2* | |
| Wittich et al. (2014) | Depression  Social isolation | 15-item GDS  Friendship Scale  LSQ | *Psychological well-being. Screening for the presence of depressive symptoms was conducted using the 15-item Geriatric Depression Scale… The Friendship Scale and the Life Space Questionnaire (LSQ) were used to measure social isolation* | |
| Castle et al. (2021) | Anxiety  Changes in clinical symptomology (mental health) | Not identified | *Sight loss in older adults has been associated with lower psychological well-being, poorer quality of life, functional impairment in daily life and higher rates of depression and anxiety... Instruments have varied from established therapeutic instruments such as the Core Outcome Measure (CORE OM)64 to a focus on one aspect of psychological well-being such as anxiety,82 or changes in clinical symptomology of psychological conditions* | |
| Yuzawa et al. (2013) | QoL | e.g. 25-item NEI-VFQ | *The National Eye Institute Visual Functioning Questionnaire (NEI VFQ-25) is the most widely used eye disease-specific QoL instrument in AMD. It has been shown to correlate significantly*  *with visual acuity (VA). QoL reflects aspects of AMD including psychological well-being, functional capacity, and the ability to perform patients’ valued activities, which are not captured*  *by a single, numerical VA score* | |
| Rees et al. (2016) | Anxiety  Depression | HADS | *This study is needed to clarify inconsistent findings regarding the association between diabetes-related eye complications and psychological well-being. Assessment of Psychological Well-being. Anxiety and depression were assessed using the Hospital Anxiety and Depression Scale (HADS)* | |
| Cruess et al. (2007) | Anxiety  Depression | HADS | *The utilization of the HADS instrument is a novel means by which to assess the psychological well-being of subjects with neovascular AMD.* | |
| Chou and Chi (2004) | Depression | 15-item GDS | *The impact of visual impairment on psychological well-being among elderly Chinese is more robust than hearing loss...* *Depression. The 15-item Geriatric Depression Scale (GDS) was used to measure depressive symptoms… It seems that auditory acuity deteriorate, the self-perceived health status suffers, which in turn leads to increasing the negative consequences in psychological well-being such as depressive symptoms.* | |
| Kirkcaldy and Barr (2011) | Used synonymously with psychosocial well-being  Component of QoL  Motivation to try new things  Happiness  Confidence  Limitations on going out of the home  Frustration  Worry  Control  Reliance on others | Birmingham Assessment of Low Vision Focus-QoL Questionnaire | *The Birmingham Assessment of Low Vision Focus-QoL Questionnaire (Fylan et al., 2005; Morrison-Fokken, 2005) was used to generate data concerning changes in QoL that could be associated with the three programmes evaluated. It was developed to provide an assessment of visual functioning and psychosocial well-being... A further eight questions assessed participants’ psychological well-being, including motivation to try new things, general happiness, levels of confidence, limitations on going out of the home, frustration, worry, control and reliance on others. Total scores potentially ranged from 0 to 100, with higher scores indicating a better QoL.* | |
| Allen et al. (1999) | Component of HRQoL  Anxiety  Depression | EQ-5D | *Within 1 month of phacoemulsification, change in vision was accompanied by significant changes in HR-QoL functions such as home activities, social activities, self-care, mobility, and psychological well-being…*  *One such instrument is the EuroQoL,3 which was designed as a self-completed questionnaire for use in large-scale surveys of the community. It covers 5 dimensions of health: mobility, self-care, usual activities, pain/discomfort, and anxiety/depression.* | |
| Li et al. (2013) | Depression | CES-D | *Identified individuals were administered the Center for Epidemiological Studies Depression Scale (CES-D) … Over one-third of all individuals with visual disabilities registered with the Disabled Persons’ Federation in Wuhan have clinically significant depressive symptoms. Therefore, services for persons with serious visual disabilities – and for individuals with other types of serious physical or sensory disabilities – should include activities aimed at improving psychological wellbeing, periodic screening for psychological problems and, when needed, professional treatment for mental disorders.* | |
| Horowitz and Reinhardt (1998) | Not clear | Adjustment to Blindness | *Fitting (1954) stressed the importance of both functional skill and psychological well-being in adjustment. His measure, Adjustment to Blindness, includes six conceptual and empirical domains: attitudes toward sighted persons, outlook on blindness, family relationships, attitudes toward rehabilitative training, occupational outlook, and general well-being.* | |
| Mozaffar Jalali et al. (2014) | Anxiety  Depression  Stress  Self-esteem | DASS (Persian version)  Eysenck Self-Esteem Inventory | *The scales of depression, anxiety and stress (DASS) which was constructed by Lovibond and Lovibond in 1995… Eysenck’s Self Esteem Inventory This questionnaire which was constructed by Eysenck in 1976 has been used to measure self esteem… In this study, factors regarding psychological well being such as depression, anxiety, stress and self-esteem were discussed and analysed* | |
| Barr et al. (2012) | Psychological distress:  Subjective well-being (feelings about self and future)  Problems/symptoms (depression, anxiety, physical health, and trauma)  Life functioning (general day-to-day functioning, close relationships and social relationships)  Risk to self and others | CORE-OM | *Comparisons were made between service users’ CORE Outcome Measure (CORE-OM) scores at baseline and post-intervention to assess the impact of the services on psychological wellbeing… The CORE-OM was designed to be suitable for use across a wide variety of client groups and service types. It taps into a ‘core’ of clients’ distress, including subjective wellbeing, commonly experienced problems or symptoms, and life/social functioning. In addition, items on risk to self and to others are included…However, mean item scores for each of the four domains of wellbeing, problems/symptoms, life functioning and risk can also be used separately where it is desirable to produce a finer grained analysis. The CORE-OM has 34 items addressing these four domains, all of which are scored on a five-point Likert scale ranging from 0 (not at all) to 4 (most or all of the time), higher scores reflecting greater psychological distress in the participant… The specific domains of psychological functioning found to present the greatest concern for this sample of people with visual impairment were wellbeing (feelings about oneself and the future) and problems (depression, anxiety, physical health, and trauma). Least concern was evident in the domains of functioning (general day-to-day functioning, close relationships and social relationships) and risk (to self and others).* | |
| Nyman et al. (2012) | Depression  Self-worth  Fear of further sight loss | Not identified | *Psychological well-being was reduced with the onset of depression (n=4), low self-worth (n=8), and fear of further vision loss (n=9), and social well-being was reduced by difficulties in social functioning (n = 5) and social isolation (n=5).* | |
| Peters et al. (2013) | Used synonymously with emotional well-being  Psychological distress:  Anxiety  Depression  Phobic anxiety (fear in public and open places)  Somatisation (perception of bodily dysfunction)  Paranoid ideation (projection of hostility, suspiciousness, fear of loss of autonomy)  Hostility  Obsessive–Compulsive  Interpersonal sensitivity  Psychoticism | Brief Symptom Inventory (BSI) | *The data points show visits where the BSI was administered to assess emotional wellbeing… We chose the BSI as an additional questionnaire to accompany the patients in the Retinal Implant Study, because we wished to obtain a measure of the psychological wellbeing (and hence a symptom inventory). This questionnaire provides patient-reported data on 53 items, used to measure nine primary symptom dimensions: Somatization (SOM); Obsessive–Compulsive (O–C); Interpersonal Sensitivity (I-S); Depression (DEP); Anxiety (ANX); Hostility (HOS); Phobic Anxiety (PHOB); Paranoid Ideation (PAR) and Psychoticism (PSY) [8]. The questionnaire provides three major indices representing the overall psychological distress level for the time of testing (total Global Severity Index, tGSI), the severity of reported symptoms (total Positive Symptom Distress Index, tPSDI) and the number of self-reported symptoms (total Positive Symptom Total, tPST).* | |
| Ahmmed et al. (2021) | Psychological distress | Not identified | *Additionally, significantly higher levels of psychological distress were noted in 149 men when compared to baseline for the Iranian population and those with amputated limbs from war-related injuries; highlighting how detrimental OSCI can be on psychological wellbeing* | |
| Dersh (1997) | Anxiety  Depression  Attitudes towards vision loss  Acceptance of disability  Self-efficacy  Locus of Control  Attributional Style | NAS 2 | *Table 1: Dependent Variable Measures of Functional Skill And Psychological Well-being and Possible Covariates: Psychological Variables. 1. Anxiety 2. Depression 3. Self-esteem 4. Attitudes toward Disability 5. Acceptance of Vision Loss 6. Self-efficacy 7. Locus of Control 8. Attributional Style* (p.32)… *The psychological well-being variables were measured at three points in time… Based on these analyses five functional skills variables and four psychological well-being variables were selected. The dependent variables tested were: Psychological well-being variables. Depression, Attitudes towards vision loss, Anxiety, Acceptance of disability* (p.150) | |
| Pankow et al. (2004) | Psychological reaction to vision loss:  Anxiety  Depression  Self-esteem  Acceptance of disability  Adaptation to disability  Self-efficacy | NAS 2 | *Each participant had pretests of living skills performance, independent movement, and psychological well-being. The Nottingham Adjustment Scale 2 (NAS2) is an assessment of psychological reaction to vision loss. The scale comprises questions drawn from previously published measures with existing validity and reliability data, which address the psychological factors of depression, anxiety, self-esteem, acceptance of disability, adaptation to disability, and self-efficacy* | |
| Hodge et al. (2013) | Subjective well-being  Problems/symptoms  Functioning  Risk to self and others | CORE-OM | *Data collected using a standardised measure of psychological well-being (Clinical Outcomes in Routine Evaluation–Outcome Measure; CORE-OM) show an improvement in the psychological well-being of clients of the service between baseline and follow-up assessment… The CORE-OM is a 34-item validated measure of psychological well-being and social functioning that looks at four domains: subjective well-being, problems/symptoms, functioning, and risk to self and others* | |
| Bambara et al. (2009) | Not identified | Not identified | *McIlvane and Reinhardt (2001) found that women with high levels of support from both friends and relatives experienced better psychological well-being, whereas men with high levels of support from both friends and relatives or only from relatives had better psychological well-being.* | |
| Reinhardt et al. (2006) | Not identified | Not identified | *After a lifetime with sight, the experience of vision loss in later life can be devastating, and is often accompanied by functional disability and poor psychological well-being* | |
| Rees et al. (2013) | Depression  Vision-specific distress | PHQ-9  IVI – emotional well-being subscale | *Research from other disability areas also indicate that a person’s confidence in their ability to cope (i.e., coping efficacy) is likely to be a key factor in determining psychological well-being. Coping efficacy, use of social support, and other coping strategies may therefore be important predictors of vision-specific distress and/or depression* | |
| Engel et al. (2000) | Not identified | Not identified | *Specifically, cross-sectional studies have found that the loss of vision is associated with difficulties in performing basic activities of daily living (…), with health problems and the perceptions of poorer health status (…), with psychological and emotional well-being (…), and with social interactions (…)* | |
| Zheng et al. (2012) | Used synonymously with mental well-being  Psychosocial mental health:  Anxiety  Depression  Feeling calm and peaceful  Feeling downhearted and blue  Energy  Perceived mental health status | From SF-12 (mental component measure):  How much of the time during the past 4 weeks have you felt calm and peaceful?  How much of the time during the past 4 weeks have you felt downhearted and blue?  How much of the time during the past 4 weeks did you have a lot of energy?  From EQ-5D (mental status domain):  Problems with anxiety/depression: no problem, some problem, major problem?  Perceived mental health status: poor, fair, good, very good, excellent? | *Mojon-Azzi and colleagues24 analyzed mental well-being indicators in persons aged 50 years and older using data from the 2004 Survey of Health, Ageing, and Retirement in Europe (SHARE) and found self-reported low vision was associated with lower psychological well-being. Although related to depression, overall psychological well-being is a broader indicator of self-rated or perceived psychosocial mental health… Although there is no established methodology of assessing mental well-being, the five quantitative mental well-being indicators used in this study are consistent with those of Monjon-Azzi and associates24 and measure overall psychological well-being… Mental well-being was analyzed as a latent variable with five indicators. Each indicator was derived from one of the following questions: 1, ‘‘How much of the time during the past 4 weeks have you felt calm and peaceful?’’ 2, ‘‘How much of the time during the past 4 weeks have you felt downhearted and blue?’’ 3, ‘‘How much of the time during the past 4 weeks did you have a lot of energy?’’ 4, ‘‘Problems with anxiety/depression: no problem, some problem, major problem?’’ 5, ‘‘Perceived mental health status: poor, fair, good, very good, excellent?’’ Questions 1 to 3 were from the mental component measure of the SF-12,33 and possible answers were as follows: none of the time, a little of the time, some of the time, good bit of time, most of time, all of the time. Question 4 was from the mental status domain of the EuroQol 5-D.34 Question 5 was a self-rated mental health measure.* | |
| McIlvane and Reinhardt (2001) | Used synonymously with general well-being  QoL  Depression  Life satisfaction | LSI-A  CES-D | *Psychological well-being was measured with both a positive and a negative indicator. The Life Satisfaction Index–A (LSI;…) was used as a positive indicator of well-being...... psychological well-being (i.e., depressive symptomatology and life satisfaction)...The CES-D (…) is a measure of depressive symptomatology that was used as a negative measure of well-being...We assessed both positive and negative aspects of life quality, as earlier work has stressed the importance of including both of these aspects in studies of well-being (…). Further, we examined the impact of these support variables on both the general outcome of well-being and the domain-specific outcome of adaptation to vision loss for comparison.* | |
| Zulfiqar et al. (2018) | Anxiety  Depression  Mental well-being | GAD-7  PHQ-9  WEMWBS | *Ophthalmologists seeing BD patients are aware of the serious, potentially life-threatening, systemic complication of this condition, yet may not appreciate that BD also has a profound effect on quality of life (QoL) and the psychological wellbeing of the patients… The Patient Health Questionnaire-9 (PHQ)-9 is used provisionally to diagnose depression and grade severity of symptoms … The General Anxiety Disorder-7 (GAD-7) form measures severity of anxiety … The Warwickshire-Edinburgh Mental Wellbeing Scale (WEMWBS) measures mental wellbeing using a 14- item scale with 5 response categories… BDAI was weakly correlated with EQ-5D, PHQ-9, GAD-7, and WEMWBS, but patient and clinician perception correlated stronger with all psychological scores. This implies that the number of symptoms alone, as indicated by BDAI, does not impact psychological wellbeing as much as the severity of symptoms, as indicated by clinician perception and that patient perception appears the most important determinant of psychological questionnaire scores* | |
| Peyron et al. (2011) | QoL:  Anxiety  Depressed mood  Positive well-being  Self-control  General health  Vitality | PGWB | *Quality of life was assessed according to the Psychological General Well-Being Index (PGWBI), which is a 22-item, self-reporting system…Patients rated their well-being during the past 2 weeks... A higher score indicates a feeling of well-being. The index is subdivided into 6 categories: anxiety, depressed mood, positive well-being, self-control, general health and vitality.* | |
| Pabon et al. (2017) | Anxiety  Depression  Fear  Frustration  Anger | Not identified | *The loss of vision can cause significant fear, frustration, anger, severe anxiety, and depression. Maximizing independence and engagement in meaningful activities will help the patient’s psychological well-being.* | |
| Béchetoille et al. (2008) | Component of QoL  Not identified | 36-item Glau-QoL  Psychological Wellbeing domain (see Table 3):  - Feeling that it is unfair that you are ill, rather than somebody else,  - Feeling of being misunderstood,  - Feeling of discouragement,  - Feeling of frustration,  - Feeling weak or vulnerable,  - Feeling useless | *Spilker (1990) suggested a hierarchical QoL model with three levels, the first of which corresponds to the overall assessment of QoL as proposed by the World Health Organization Quality of Life (WHOQoL) group, defined as ‘an individual’s overall satisfaction with life, and one’s general sense of personal wellbeing’. The second level comprises three main domains concerning the functional, psychological and social functioning that are necessary to achieve the satisfaction and sense of wellbeing described in the definition of the previous level. The third level corresponds to specific aspects of each of these domains (for example, measures of anxiety or depression specific to the psychological functioning domain)… Each domain of the questionnaire reliably represented a specific aspect of patients’ HRQoL, with the exception of the Psychological Wellbeing and Self-image domains, which should be interpreted jointly* | |
| McGwin and Owsley (2007) | Component of QoL  Not identified | QoL measures | *The defining feature of questionnaire measures is that they are patient-centered in that they provide the person’s own self-reported perspective on his/her quality of life, addressing domains such as difficulty in engaging in everyday activities, psychological well-being, and/or health status* | |
| Ben-Zur and Debi (2005) | Life satisfaction  Mental health:  Positive affect (joy, interest, excitement, confidence, and alertness)  Negative affect (subjective distress, dissatisfaction, anger, fear, sadness, guilt, contempt, and disgust) | PANAS | *Psychological well-being is composed of cognitive and affective components and is defined as a subjective global state of satisfaction and positive mental health (…). The affective component, which was tested in this study, is based on two dimensions of emotional experience, termed positive affect (PA) and negative affect (NA) (…). PA reflects the concurrence of positive emotional states, such as joy, interest, excitement, confidence, and alertness, whereas NA describes subjective distress and dissatisfaction and is composed of negative emotional states, such as anger, fear, sadness, guilt, contempt, and disgust.* | |
| Sweeting et al. (2020) | Used synonymously with mental well-being  Anxiety  Depression  Stress  Mood  QoL  Emotional well-being | Not identified | *In addition to physical health, regular physical activity is also known to benefit psychological well-being including a reduction in the risk of depression and anxiety, lowering of stress levels and improving mood… Six (35%) studies examined the impact of the intervention of an aspect of mental well-being such as anxiety, depression or quality of life… Psychological well-being was measured in only six studies and no significant results were observed in these outcomes… Table 2 - Summary of outcome measures: Psychological well-being §Including measures of quality of life, anxiety, depression and emotional well-being* | |
| Onuigbo et al. (2020) | Self-esteem | Rosenberg Self‑Esteem Scale | *A sense of mastery is a component of psychological well-being in the areas of self-evaluative processes such as self-esteem. Falci (2011) asserts that a sense of mastery is an important evaluative dimension of self that is linked to self-esteem.* | |
| Garcia et al. (2017) | Mood (affect)  Quality of interpersonal interactions (social participation, social relationships)  Career goals (career aspirations and motivations) | Have you experienced any of the following in the past 2 weeks:  1) depressed mood,  2) anhedonia (“loss of interest or pleasure in doing things you previously enjoyed”),  3) feelings of guilt or worthlessness,  4) fatigue,  5) changes in appetite,  6) sleep disturbances,  7) psychomotor retardation or agitation,  8) difficulty concentrating,  9) suicidal ideations  Impact rating (IR) of vision loss on the quality of interpersonal interactions and career goals (21-point Likert scale) | *The model of psychological well-being employed in this investigation is outlined in Figure 1. Profound vision loss serves as the primary physiological stressor in this model. Psychological well-being has three components in this model: mood (affect), quality of an individual’s interpersonal interactions, and career goals. Interpersonal interactions comprise social participation and relationships both inside and outside of one’s household. Career goals represent aspirations and motivation for subjective career success within one’s current career or desired future vocation. This is a future-oriented paradigm, rather than an assessment of happiness with current career status, as the latter can vary between individuals regardless of outside influences. Furthermore, this forward-thinking assessment of career goals accounts for individuals who are not employed or do not yet have an established career, which may be particularly relevant among younger individuals. These components of psychological well-being may also influence one another and likely contribute to the overall quality of life. Although other factors can impact psychological well-being, the three elements employed in this model were of particular interest in the analyses because of their central importance in long-term psychological health, and because of their susceptibility to profound change with vision loss.* | |
| Bergeron and Wanet-Defalque (2013) | Adaptation to vision loss | AVL | *Individuals with visual impairment may experience a degradation of psychological well-being, decreased life satisfaction, and poor adaptation to vision loss… More recently, Schilling and colleagues (2011) conducted a 1-year follow-up study among 451 AMD patients to establish the adaptation dynamics of chronic functional impairment. Their results suggested that perceived functional vision losses mediated the effects of vision loss on psychological well-being (measured by the Adaptation to Age-Related Vision Loss Scale [AVL]* | |
| Rees et al. (2010a) | Mental health  Anxiety  Depression  Vision-specific distress  Self-efficacy  Coping  Adjustment to vision loss  Mood  Emotional distress  Activities, beliefs, expectations and satisfaction with visual function  Perceived security in performing daily occupations  Component of QoL  HRQoL  Self-esteem  Goal attainment  Life perspective (outlook on life)  Locus of control | POMS  AMD self-efficacy Questionnaire  Clinical interview (DSM-IV)  15-item GDS-15  HDRS  CES-D  NAS  AVL  NEI-VFQ  PGWB  SF-36 (full and mental health score only)  10-item Vision Core Measure (VCM1)  Age-related vision loss self-efficacy questionnaire  Rosenberg’s self-esteem scale  Goal-attainment scaling  Cantril’s self-anchoring striving scale  Rotter internal-external locus of control scale | *Given the prevalence of distress in people with vision impairment and the interrelationship between depression and disability, it is critical to understand the impact of vision rehabilitation on psychological well-being… Only those studies that included scales or subscales assessing mental health, psycho scales or subscales assessing mental health, psychological symptoms (e.g., anxiety and/or depression), or measures of vision-specific distress or adjustment were included. We also included studies that assessed broader aspects of psychological well-being including constructs such as self-efficacy and coping…Psychological outcomes were examined with 34 different measures. Well-established measures of depression were commonly used, and were reported in eleven studies (31%). These included the Centre for Epidemiological Studies-Depression (CES-D) scale [18] and the Geriatric Depression Scale (GDS) [19]. In addition, mental health scores from well-validated health quality of life measures, such as the Short Form-36 Health Survey (SF-36) [20] were used in five studies. Vision-specific measures of adjustment to vision loss, including the Nottingham Adjustment Scale (NAS) [21] and the Adaptation to Age-Related Vision Loss scale (AVL) [22], were used in seven studies (23%), although the psychometric properties of these measures are not fully established. A number of measures of vision-specific quality of life that include an assessment of psychological well-being were used, although the psychometric properties of many of these scales remain unclear. For example, while the National Eye Institute visual function questionnaire (NEI-VFQ) contains a mental health subscale, it was developed predominantly as a functional measure and has not been validated as a measure of psychological outcomes…Table 1 - Summary of randomized controlled trials.* | |
| Mirandola et al. (2019) | Autonomy  Environmental mastery  Personal growth  Positive relations with others  Purpose in life  Self-acceptance | 18-item PWB (Italian version) | *psychological well-being (PWB)… Finally, the third part included the 18-item Italian versions of the PWB (PWB-18) scale and the Short Form-12 (SF-12) questionnaire to assess the PWB and QoL, respectively [23–30]. In particular, PWB-18 scale, based on Ryff’s multidimensional model of PWB, evaluates the personal perception of well-being relative to 6 dimensions: autonomy, environmental mastery, personal growth, positive relations with others, purpose in life, and self-acceptance…* | |
| Iuliano et al. (2013) | Autonomy  Environmental mastery  Personal growth  Positive relations with others  Purpose in life  Self-acceptance | PWB (Italian version) | *The Psychological Well-Being Scale (PWB Scale) (…). PWB Italian version (…) assesses personal perceptions of well-being relative to 6 dimensions: autonomy (AU), positive relations with others (PR), environmental mastery (EM), personal growth (PG), purpose in life (PL), self-acceptance (SA)* | |
| Pinquart and Pfeiffer (2011) | Mental health  Positive affect/emotions  Negative affect/emotions  Self-acceptance  Life satisfaction  Subjective well-being  Psychological health  QoL  Depression  Anxiety  Loneliness  Self-concept  Self-esteem | 25-item NEI VFQ (mental health subscale)  SF-36  SF-12  CES-D  GDS  HADS (anxiety subscale)  PGCM  Single items on the frequency of happiness  Tennessee Self-Concept Scale  Rosenberg Self Esteem Scale | *Given the effects of vision loss on daily life (…), it is believed that limited vision performance is negatively associated with psychological well-being (PWB). With the term PWB we refer to internal, individual states, such as being free of mental illness, having high levels of positive emotions/low levels of negative emotions, self-acceptance, and being satisfied with life...*  *Studies were identified from the literature through electronic databases [PSYCINFO; MEDLINE; CINAHL; EMBASE – search terms: (visual impairment or blindness or low vision or glaucoma or cataract or diabetic retinopathy or retinitis or AMD) and (PWB or subjective well-being or psychological health or mental health or quality of life or depression or anxiety or loneliness or positive affect or life satisfaction or self-concept or self-esteem)], and cross-referencing.* | |
| Abas et al. (2009) | Harmony  Interdependence with close persons  Respect from others  Acceptance  Enjoyment | 15-item Thai psychological well-being scale | *One aim of this study was to see whether patterns of association between impairment, disability and psychological well-being in Thailand are similar to or different from those described elsewhere. Psychological well-being. We used the 15-item Thai well-being scale [12, 18], developed using extensive qualitative and quantitative methods. It has five dimensions of wellbeing which are harmony, interdependence with close persons, respect (from others), acceptance and enjoyment.* | |
| Cooper et al. (2020) | Anxiety  Depression  Mood | Not identified | *Outcomes: Psychological well-being, such as depression and anxiety… Studies were categorised in the review into psychological well-being if they included measures of mood, depression, and/or anxiety.* | |
| Mitchell and Bradley (2001) | Used synonymously with general well-being  Anxiety  Depression  Energy (feeling energetic, active or vigorous, feeling dull or sluggish, feeling tired, worn out, used up or exhausted, waking up feeling fresh and rested)  Positive well-being (feeling happy, satisfied or pleased with my personal life, have lived the kind of life I wanted to, feeling eager to tackle my daily tasks or make new decisions, feeling I could easily handle or cope with any serious problem or major change in my life)  Negative well-being (Feeling tearful, downhearted and blue, feeling afraid for no reason at all, getting upset easily or feeling panicky) | W-BQ12  W-BQ22 | *This paper reports the evaluation of a generic measure of psychological well-being, the 12-item Well-Being Questionnaire (W-BQ12), for use with people who have the chronic eye condition, macular disease (MD)… Unforced factor analysis elicted the expected three factors representing constructs of positive well-being, energy and negative well-being. A forced single-factor solution supported use of the whole scale to measure total general well-being… The original scale developed contained 22 items (W-BQ22) and had a four-factor structure with subscales measuring depression, anxiety, energy and positive well-being… The W-BQ12 asks people how often they have experienced the feelings mentioned in each statement over the past few weeks… Table 1. W-BQ12 items and factor analysis rotated component matrix. Neg 1 I have crying spells or feel like it, Neg 2 I feel downhearted and blue, Neg 3 I feel afraid for no reason at all, Neg 4 I get upset easily or feel panicky, Energy 1 I feel energetic, active or vigorous, Energy 2 I feel dull or sluggish, Energy 3 I feel tired, worn out, used up or exhausted, Energy 4 I have been waking up feeling fresh and rested, Pos 1 I have been happy, satisfied or pleased with my personal life, Pos 2 I have lived the kind of life I wanted to, Pos 3 I have felt eager to tackle my daily tasks or make new decisions, Pos 4 I have felt I could easily handle or cope with any serious problem or major change in my life* | |
| Dev et al. (2014) | Component of QoL  Not identified | Not identified | *QoL can be assessed with measures of health status, functional status and psychological well-being… Visual function is important for an optimal orientation in functional and social life and has effects on physical, psychological, mental and emotional well-being* | |
| Mitchell and Bradley (2006) | Mood  Anxiety  Depression  Energy/Vitality  Enthusiasm for life  Emotional distress | HADS  SF-36  W-BQ12  HUI-3  EQ-5D  Sickness Impact Profile (SIP)  Sickness Impact for vision (SIPv) Low vision QoL (LVQOL)  POMS  DSM-IV  GHQ  PANAS  Vision-related QoL (VQOL)  GDS  PGWB | *2.1 Measuring Patient Reported Outcomes (PROs) in eye disease 2.1.1 Psychological well-being measures. These measure mood. People who feel depressed and anxious are unlikely to describe their QoL as good. However, even those who are not depressed or anxious may still feel that their QoL is severely damaged by MD. Some well-being scales, such as the Hospital Anxiety and Depression Scale (HADS) [13] measure only negative well-being (anxiety and depression). Where people have no anxiety or depression to begin with, such a measure could show no improvement. Measures which also investigate positive well-being (e.g. the well-being scales within the SF-36 measure vitality [14]) and particularly those which measure positive well-being with items concerned with enthusiasm for life (e.g. the Well-being Questionnaire (WBQ12) which measures energy and positive well-being as well as anxiety and depression [15]) are more likely to detect improvement in psychological well-being… Table 1: Patient reported outcomes referred to, constructs measured and populations for which they are validated… 3.1 Psychological well-being. A number of studies have looked at the impact of MD on psychological well-being. In an American cross-sectional study investigating 86 MD patients with a VA of 20/200 or worse in at least one eye [30] participants reported greater emotional distress (Profile of Mood States [POMS] [65]) than similar aged people without visual impairment* | |
| Kaleemunnisha et al. (2014) | Component of VRQoL  Social functioning  Mental health  Role difficulties  Dependency | 25-item NEI-VFQ | *Vision related quality of life (VR-QOL) was evaluated using the National Eye Institute Visual Functioning Questionnaire (NEI VFQ-25)… The four components of psychological well being-social functioning, mental health, role difficulties, dependency showed moderate to large improvement in the sub-scale scores similar to other studies.* | |
| Thetford et al. (2015) | Not identified | Qualitative interviews | *Vision impairment has multi-faceted impacts upon people's lives; it impacts on functional ability and mobility (both in terms of getting out and about and individual functional mobility), which affects social interaction and psychological wellbeing… Without intervention, Eva was vulnerable because of her heavy dependency on her husband, and was in a ‘spiral of decline’ associated with reduced everyday activities and social interaction which impact on psychological wellbeing* | |
| Shpigelman and Vorobioff (2019) | Self-acceptance  Autonomy | Qualitative interviews | *Self-acceptance is a key component of psychological wellbeing. Autonomy, which involve skills of self-determination, is another important component of psychological wellbeing* | |
| Bazargan et al. (2001) | Used synonymously with subjective well-being  Not identified | PGCM | *Subjective well-being.—The dependent variable, subjective well-being, was measured by the 17-item revised version of The Philadelphia Geriatric Center (PGC) Morale Scale (Lawton, 1975)... Higher scores in this index represent a higher level of psychological well-being.* | |
| Lehane et al. (2017b) | Not clear | WHO-5 | *The aim of the current study was to investigate sexuality among couples living with ADB in Denmark and its association with psychological well-being … The WHO-5 [34] measures current psychological well-being. It contains five items referring to positive well-being such as how often over the past two weeks an individual has ‘‘felt cheerful and in good spirits’’… Higher scores indicate better psychological well-being. The scale cut-off point is 13. Scores lower than 13 indicate poor well-being and the need to assess for depression.* | |
| Cimarolli and Boerner (2005) | Life satisfaction  Depression | 10-item CES-D  SWLS | *In a final step, patterns of combinations of support received—"positive support only," "negative support only," "negative and positive support," and "no support"—were determined, and mean differences on the two indicators of well-being (depressive symptomatology and life satisfaction) were explored among the groups* | |
| Upton et al. (1998) | Positive affect  Negative affect | ABS (positive and negative affect subscales) | *The positive- and negative-affect subscales of the Bradburn Affect Balance Scale (…) were used as subjective measures of general psychological well-being in addition to the more symptom-focused HSCL. These subscales tap subjects' experiences of positive and negative affective states during the previous few weeks.* | |
| Dean (1999) | Psychological wellness | Conversational Symptom Assessment (CSA) | *The effectiveness of TELECARE intervention with 13 graduates/students of blind rehabilitation centers was studied by using the Conversational Symptom Assessment (CSA,…), which assesses the psychological wellness and adjustment in six dimensions, and the Blind Skills Assessment (BSA) which assesses the skill use and mastery level of abilities on six dimensions. Each participant was given these scales as pre and posttest measures of psychological well-being and skill use/mastery.* | |
| Matthews et al. (2017) | Used synonymously with mental well-being  Depression  Life satisfaction  QoL | CES-D  SWLS  CASP | *Using longitudinal data, we investigate whether deterioration and improvement in self-reported vision among people aged 50 years and older in England experience subsequent changes in various aspects of economic, psychological and social well-being… Depressive symptoms are measured using an eight-point version of the Center for Epidemiologic Studies Depression (CES-D) scale score… Satisfaction with life is measured using the Satisfaction With Life Scale... Quality of life is measured using the Control, Autonomy, Self-realisation, and Pleasure (CASP) scale... Increasing the uptake of free eye tests among older people potentially provides an effective means of protecting against vision decline42 and the subsequent decline in mental, social and financial well-being associated with worsening sight* | |
| Hernandez Trillo and Dickinson (2012) | QoL | Not identified | *QoL is used to define the physical, psychological, functional, social, and economic well-being of an individual: the impact of a disease may reduce health-related quality of life8 or in this specific case, vision-related QoL* | |
| Reinhardt (1996) | Depression  Life satisfaction | 20-item CES-D  18-item LSI-A | *Adaptation was operationalized with two global measures of psychological well-being (life satisfaction, depressive symptoms) and a domain-specific measure, adaptation to vision loss…* *The 18-item version (…) of the Life Satisfaction Index-A (LSI-A) was used as a measure of psychological well-being. The 20-item Center for Epidemiological Studies Depression Scale (CES-D) was used as a measure of depressive symptomatology* | |
| Glick et al. (2019) | Not identified | Not identified | *The estimates thus confirm the existence of a range of benefits to cataract surgery in a very low income setting, and are in line with a number of studies in developed and developing countries showing improvements in physical functioning, psychological well-being, and social inclusion among older adults following successful cataract surgery* | |
| Sorensen et al. (2015) | Autonomy  Environmental mastery  Personal growth  Positive relations with others  Purpose in life  Self-acceptance | PWB | *Other data collected included detailed assessments of psychological well-being, depression and anxiety, attitudes toward the future, preparation for future care, valued activities, life satisfaction, and life events… The first was the Psychological Well-Being scale (PWB),48 a 42-item multidimensional measure assessing 6 dimensions of PWB: Autonomy, Environmental Mastery, Personal Growth, Positive Relations With Others, Purpose In Life, and Self-Acceptance* | |
| Orr (1991) | Not identified | Not identified | *Visual impairment can have a significantly greater negative impact on the psychological well being of the older person if personal and professional supports are not in place to understand, encourage, teach and support the individual through the process of adjustment.* | |
| Smedema and McKenzie (2010) | Not identified | SWBI | *The SWBI is a subjective well-being measure developed specifically for people with disabilities [39]... The SWBI consists of five subscales: physical well-being and associated feelings about self, psychological well-being, family and social well-being, financial well-being, and medical care.* | |
| Yu and Liljas (2019) | Not clear | Not identified | *Hearing and vision impairments have also been associated with poor self-rated health,10-12 a strong subjective measure for overall health that encompasses both physical health and psychological well-being, predicting major adverse health outcomes such as chronic illness,13 functional decline14,15 and depression* | |
| Guerette and Smedema (2011) | Component of subjective well-being  Self-esteem | SWBI | *Subjective well-being was measured via the outcome variables from Cimarolli and Boerner’s study (depressive symptoms and satisfaction with life), along with five additional sense of wellbeing factors: physical well-being, psychological well-being, financial well-being, family or social well-being, and medical well-being… Subjective well-being was measured by the SWBI... A significant positive association was found in the area of psychological well-being with both age and social support, suggesting that older individuals and those who report more social support have higher levels of psychological well-being (like self-esteem).* | |
| van der Aa et al. (2016) | Component of VRQoL  Mental health | NEI-VFQ  IVI - emotional well-being subscale  WB-Q - negative well-being subscale  CORE-OM psychosocial well-being subscale | *Psychological well-being was mostly determined with a mental health subscale of vision-related quality of life questionnaires: four studies15,29-31 used the ‘mental 14 health’ subscale of the National Eye Institute Visual Functioning Questionnaire (NEIVFQ), one study36 used the ‘emotional well-being’ subscale of the Impact of Visual Impairment scale (IVI); one study25used the ‘negative well-being’ subscale of the Well-Being Questionnaire (WB-Q), which shows good reliability and validity in people with macular disease,60 and another study39 used the ‘psychosocial well-being’ subscale of the CORE outcome measure,* | |
| Mental well-being | | | | |
| Study | **Indicator** | **Measure** | **Quote** | |
| Riazi et al. (2014) | Used synonymously with psychological well-being  Mental health | WEMWBS | *Our primary outcome measure is psychological well-being, measured by the Warwick-Edinburgh Mental Well-being Scale (WEMWBS). The WEMWBS is a scale for assessing positive mental health (mental well-being). It covers most aspects of positive mental health (positive thoughts and feelings) currently in the literature, including both hedonic and eudaimonic perspectives.* | |
| Yuzawa et al. (2013) | Component of QoL  Not identified | QoL measures | *World Health Organization constitution describes health as a state of physical, mental, and social well-being, not merely the absence of disease or infirmity. Consequently, the measurement of health should encompass an assessment of mental and social well-being, which can be accomplished by using QoL measures.* | |
| Heine and Browning (2002) | Not identified | Not identified | *They are often aware of the consequences of their sensory loss and acknowledge that their sensory loss impacts on their physical and mental well-being, often resulting in decreased social performance.* | |
| Svaerke et al. (2019) | Not identified | SCL-90-R | *The Symptom Checklist-90-Revised (SCL-90-R) is a mental well-being questionnaire…* | |
| Anil and Garip (2018) | Not identified | WEMWBS | *Emotional Health was measured using Depression, Mental Wellbeing (MW), and General Happiness with Life (GHL), which were recorded using standardised questionnaires (detailed in materials).* | |
| Acton et al. (2016b) | Used synonymously with subjective well-being  Positive thoughts and feelings | WEMWBS | *Mental well-being is a prominent issue in governmental health policy, and the utility of the WEMWBS has been demonstrated in general populations… The Warwick-Edinburgh Mental Well-being Scale (WEMWBS) - population measure of subjective well-being, involving 14 positively worded questions about aspects of positive thoughts and feelings* | |
| Rooney et al. (2018) | Not identified | Not identified | *The quality of the home environment influences an occupant’s mental and physical wellbeing, although not always positively* | |
| Hodge et al. (2013) | Psychological distress | Qualitative interviews | *Three individuals report feeling that their mental well-being was so low at the time they went for counselling that they had either had thoughts of ending their lives or just wished they were dead… These quotes illustrate the level of psychological distress experienced by some of the participants in the study* | |
| Dillon et al. (2020) | Mental health  Anxiety  Depression  Nervous breakdowns | Qualitative interviews | *Stakeholders expressed that many of their clients have concerns regarding their mental well-being, indicating the importance of this topic and need for such programs: “I’m finding most of the clients that I…work with, express that they have anxiety, depression, or have had nervous breakdowns, or are currently having mental health issues.”* | |
| Kelly et al. (2021) | Component of VR and HRQoL  Mental health | SF-36  NEI-VFQ  IND-VFQ | *In addition to the NEI-VFQ and SF-36v2, participants enrolled in India also completed the IND-VFQ.… The 25-item NEI-VFQ is a validated VRQoL questionnaire that measures functioning and well-being in physical, mental, and social aspects of a patient’s life... findings from a recent Rasch analysis demonstrated a higher validity of the IND-VFQ when using 4 subscales instead of 3 (vision-specific mobility, activity limitation, psychosocial impact, and visual symptoms)… The SF-36v2 evaluates HRQoL by assessing a patient’s self-perception of their physical and mental health... The questionnaire can be divided into 2 components, physical and mental health, and 8 domains: physical functioning, role limitations caused by physical health, bodily pain, general health perceptions, vitality (energy and fatigue), general mental health (psychological distress), role limitations because of emotional problems, and social functioning limitations because of emotional problems... This study found that the antimetabolites methotrexate and mycophenolate mofetil significantly improved the physical and mental well-being of patients with noninfectious uveitis* | |
| Gleeson et al. (2017) | Not identified | Not identified | *An eight-year prospective longitudinal study on the impact of physical and leisure activity on mental well-being in the elderly found that a higher level of leisure activity, better levels of mobility and a low level of chronic health conditions predicted mental well-being in later life.* | |
| Menon et al. (2020) | Not identified | Not identified | *Objective. Vision loss has a significant impact on physical, mental and social well-being* | |
| Zheng et al. (2012) | Used synonymously with psychological well-being  Psychosocial mental health:  Anxiety  Depression  Feeling calm and peaceful  Feeling downhearted and blue  Energy  Perceived mental health status | From SF-12 (mental component measure):  How much of the time during the past 4 weeks have you felt calm and peaceful?  How much of the time during the past 4 weeks have you felt downhearted and blue?  How much of the time during the past 4 weeks did you have a lot of energy?  From EQ-5D (mental status domain):  Problems with anxiety/depression: no problem, some problem, major problem?  Perceived mental health status: poor, fair, good, very good, excellent? | *Mojon-Azzi and colleagues 24 analyzed mental well-being indicators in persons aged 50 years and older using data from the 2004 Survey of Health, Ageing, and Retirement in Europe (SHARE) and found self-reported low vision was associated with lower psychological well-being. Although related to depression, overall psychological well-being is a broader indicator of self-rated or perceived psychosocial mental health… Mental well-being was analyzed as a latent variable with five indicators. Each indicator was derived from one of the following questions: 1, “How much of the time during the past 4 weeks have you felt calm and peaceful?” 2, “How much of the time during the past 4 weeks have you felt downhearted and blue?” 3, “How much of the time during the past 4 weeks did you have a lot of energy?” 4, “Problems with anxiety/depression: no problem, some problem, major problem?” 5, “Perceived mental health status: poor, fair, good, very good, excellent?” Questions 1 to 3 were from the mental component measure of the SF-12, 33... Question 4 was from the mental status domain of the EuroQol 5-D. 34 Question 5 was a self-rated mental health measure. Data were recoded such that higher values indicated better mental well-being.* | |
| McCormack (2021) | Not identified | Qualitative interviews | *Those individuals with visual impairments who had extensive training in independence skills prior to Hurricane Irma explained that they utilized those skills throughout the hurricane season, and it was an aid to their physical and mental wellbeing… Connecting with others socially was a benefit to the mental wellbeing of hurricane survivors with visual impairments, which contributed to confidence throughout their lives, part of which involves skills in independence* | |
| Zulfiqar et al. (2018) | Feeling  Functioning | WEMWBS | *Wellbeing: The Warwickshire-Edinburgh Mental Wellbeing Scale (WEMWBS) measures mental wellbeing using a 14- item scale with 5 response categories; 0 being “none of the time” and 5 being “all of the time”. It covers both feeling and functioning and can be compared to the general population. Scores range between 0 and 70, with 0–32 indicating a “low” wellbeing, 32–40 indicating “below average”, 40–59 suggesting “average” and 59–70 indicating “above average* | |
| McManus and Lord (2012) | Positive mental health:  Feeling optimistic about the future  Feeling useful  Feeling relaxed  Feeling close to others  Dealing with problems well  Thinking clearly  Able to make up own mind | SWEMWBS | *The Warwick Edinburgh Mental Wellbeing Scale2 (WEMWBS) was developed in recent years for assessing positive mental health (mental well-being). The short version3 (SWEMWBS), used in waves 1 and 2 of the USoc survey, is a scale consisting of seven positively worded items. These each have five response categories. It covers most aspects of positive mental health (positive thoughts and feelings) currently in the literature, including both hedonic and eudaimonic perspectives. The items are reported on separately, and a summary score is also presented in this chapter (Feeling optimistic about the future, useful, relaxed, close to others, Dealing with problems well, Thinking clearly, Able to make up own mind)* | |
| Dillon et al. (2021) | Mental health  Anxiety  Depression  Mood | Qualitative interviews | *Although mental health disorders are treatable, approximately 60% of a nationally representative sample of Australians aged 55 to 64 years meeting the criteria for a mental disorder did not make use of mental health services.13 Similar data are seen in American older adults, where approximately 70% (n = 348, >55 years old) with mood and anxiety disorders did not use mental health services.14 This highlights the need for and access to programmes that focus on mental wellbeing for older people, particularly those with AMD who are at risk of the cumulative impact of vision loss and mental health issues… We aimed to investigate the perspectives of people with AMD on mental wellbeing programmes in a novel qualitative study in order to involve them meaningfully in planning, developing and implementing a psychological programme targeting depression and anxiety.* | |
| Sweeting et al. (2020) | Used synonymously with psychological well-being  Anxiety  Depression  QoL | Not identified | *Six (35%) studies examined the impact of the intervention of an aspect of mental well-being such as anxiety, depression or quality of life… Psychological well-being was measured in only six studies and no significant results were observed in these outcomes…* | |
| Mirandola et al. (2019) | Not clear | Not clear | *On these premises, to determine whether playing baseball can improve the physical and mental well-being of people with visual impairment, here we performed a national survey based on a structured online questionnaire assessing the PWB and QoL of visually impaired baseball players from Italian teams in comparison with visually impaired sedentary individuals… Finally, the third part included the 18-item Italian versions of the PWB (PWB-18) scale and the Short Form-12 (SF-12) questionnaire to assess the PWB and QoL, respectively [23–30]. The Italian version of the SF-12 questionnaire consists of 2 components: a physical component score and a mental component score. Higher scores on these subscales indicate greater levels of functioning and a more favorable health status, thus being indicative of a better QoL [27]… Our data on QoL, which measures the perceived physical and mental health levels as well as the overall subjective well-being, seem also to support this hypothesis* | |
| Kahaly et al. (2002) | Component of QoL  Not identified | Not identified | *The third approach mainly examines the comparability of the quality of life among various individuals. In addition to physical and mental well-being, mainly the ability to function in the workplace and in the private realm is surveyed.* | |
| Stevelink and Fear (2016) | Mental health  Anxiety  Depression  PTSD  Alcohol misuse  Negative affect (irritation, frustration, anger, shock, feeling low, bereavement)  Suicide attempts  Suicidal thoughts  Social withdrawal  Sense of identity  Independence  Reliance on others  Self-worth  Self-esteem | PHQ-9  GAD-7  PTSD CheckList - Civilian version (PCL-C)  Alcohol use disorders identification test (AUDIT) Qualitative interviews | *Impact of visual impairment on mental well-being (phase 2). Becoming visually impaired was accompanied by a wide range of emotions including irritation, frustration, anger, shock, and feeling low… “It’s like a bereavement you know at the time and you had to just sort of readjust.” Female ex-Service personnel reported alcohol misuse, non-fatal suicide attempts and suicidal thoughts, social withdrawal and depressive feelings. This corresponds with the findings on the mental health screening measures. Further, their visual impairment adversely affected their sense of identity, as they had not only lost their vision, but also in some cases, their job, independence, and social activities. The loss of independence and consequently increased reliance on other people was one of the main concerns... They questioned their self-worth, as they felt that they were no longer contributing to society or able to fulfil expected roles… The results indicated that the mental well-being of ex-Service personnel who had sustained a visual impairment was adversely affected. Several participants screened positive for probable mental health disorders including PTSD, depression, anxiety and alcohol misuse…* *Further, mental well-being was hampered and this showed in various ways including feeling low, having an impaired self-esteem, being afraid for further loss of vision and being less active socially* | |
| Dev et al. (2014) | Not identified | Not identified | *Visual function is important for an optimal orientation in functional and social life and has effects on physical, psychological, mental and emotional well-being* | |
| Adigun et al. (2014) | Component of QoL  Mental health | Visual Function and Quality of Life questionnaire | *The study instrument was the standardized Visual Function and Quality of Life questionnaire designed to measure the impact of impaired vision on the patient’s ability to perform activities of daily living… Two hundred and forty-one (64.2%) patients were found to have poor quality of life in the domain of visual function; 17 (4.5%) in the domain of self-care; 166 (42.9%) in the domain of mobility; 191 (50.9%) in the social interaction domain; and 171 (47.2%) in the mental well-being domain… More than half of those in the sample reported good quality of life in terms of mental well-being and mobility... Table 3 Quality of life of the respondents in five domains (visual function, self-care, social interaction, mobility, and mental status). QoL domains: Visual function, Self-care, Mobility, Social interaction, Mental health* | |
| Rafaely et al. (2018) | Not identified | Not identified | *High levels of mental well-being were found among older adults who reported provision and/or receipt of social support and high levels of community and municipal involvement* | |
| Heine and Browning (2004) | Not clear | Qualitative interviews | *The aims were to identify the communication difficulties and conversational strategies used by the subjects, and to explore their perceptions of their social adjustment, quality of life and physical and mental well-being… The problems of adjusting to sensory loss, depression, anxiety, lethargy and social dissatisfaction were cited as factors that affected their physical and mental well-being, while being optimistic, coping with their sensory loss, and maintaining social contact contributed to an improved quality of life… Both the reaction to sensory losses and the communication limitations they impose tend to damage physical and mental well-being and psychosocial functioning, and these in turn tend to reduce social interaction and may bring about social isolation* | |
| Matthews et al. (2017) | Used synonymously with psychological well-being  Depression  Life satisfaction  QoL | CES-D  SWLS  CASP | *Using longitudinal data, we investigate whether deterioration and improvement in self-reported vision among people aged 50 years and older in England experience subsequent changes in various aspects of economic, psychological and social well-being… Depressive symptoms are measured using an eight-point version of the Center for Epidemiologic Studies Depression (CES-D) scale score… Satisfaction with life is measured using the Satisfaction With Life Scale... Quality of life is measured using the Control, Autonomy, Self-realisation, and Pleasure (CASP) scale... Increasing the uptake of free eye tests among older people potentially provides an effective means of protecting against vision decline42 and the subsequent decline in mental, social and financial well-being associated with worsening sight* | |
| Stevelink et al. (2015b) | Mental health  Anxiety  Depression  PTSD  Alcohol misuse | PHQ-9  GAD-7  PTSD CheckList - Civilian version (PCL-C)  Alcohol use disorders identification test (AUDIT) | *This cross-sectional study examined the mental well-being of ex-servicemen (aged 22–55 years) with a visual impairment and determined if the mental health of those with a combat-related visual impairment differed from those whose visual impairment is not combat-related… Male ex-service personnel with a visual impairment completed a telephone interview assessing the presence of depressive symptomatology, probable anxiety disorder, post-traumatic stress disorder (PTSD) symptomatology and alcohol misuse.* | |
| Stevelink et al. (2015a) | Depression  Self-esteem  Social activity  Interpersonal relationships  Communication | Qualitative interviews | *Sustaining a visual impairment may have a substantial impact on various life domains such as work, interpersonal relations, mobility and social and mental well-being… In addition, a negative impact on mental well-being was described such as the onset of depression, impaired self-esteem, being less socially active and experiencing challenges with regards to interpersonal relationships and communication* | |
| Vision-related psychological well-being | | | | |
| Study | **Indicator** | **Measure** | **Quote** | |
| Schilling et al. (2011) | Psychological reactions to vision loss:  Acceptance of vision loss  Recognition of limitations  Recognition of remaining capabilities  Positive attitudes towards rehabilitation  Attitudes towards close relationships | 12-item AVL | *For both samples, the 12-item version of the Adaptation to Age-Related Vision Loss Scale was utilized (…). The AVL Scale was conceptually defined and psychometrically developed to encompass specific psychological reactions to the vision loss, that is, the acceptance of the vision loss, the recognition of limitations and remaining capabilities, positive attitudes towards rehabilitation, and attitudes towards relationships with friends and family, including acceptance of needed assistance without feeling excessively dependent. A substantial body of research supports its validity as a measure of “vision related” psychological well-being and sensitivity to change over time* | |
| Psychosocial well-being | | | | |
| Study | **Indicator** | **Measure** | **Quote** | |
| Kirkcaldy and Barr (2011) | Used synonymously with psychological well-being  Component of QoL  Motivation to try new things  Happiness  Confidence  Limitations on going out of the home  Frustration  Worry  Control  Reliance on others | Birmingham Assessment of Low Vision Focus-QoL Questionnaire | *The Birmingham Assessment of Low Vision Focus-QoL Questionnaire (…) was used to generate data concerning changes in QoL that could be associated with the three programmes evaluated. It was developed to provide an assessment of visual functioning and psychosocial well-being... A further eight questions assessed participants’ psychological well-being, including motivation to try new things, general happiness, levels of confidence, limitations on going out of the home, frustration, worry, control and reliance on others. Total scores potentially ranged from 0 to 100, with higher scores indicating a better QoL.* | |
| Paz et al. (2013) | Component of HRQoL  Frustration/upset  Annoyance/anger  Sadness/depression  Social isolation  Feeling like a burden on others  Worry  Concerned about safety at home  Reliance on others  Visits with family and friends | Vision-targeted HRQoL measure | *We conducted a review of existing vision-targeted HRQOL surveys and identified color vision, low luminance vision, distance vision, general vision, near vision, ocular symptoms, psychosocial well-being, and role performance domains... Response options for the psychosocial well-being items were tailored specifically to what the item was asking, with standard phrasings of “Did not feel … at all,” “Felt a little bit …,” “Felt somewhat …,” and “Felt … a lot.” For example, an item that asked about feeling frustrated or upset had the following response options: (1) Did not feel frustrated or upset at all, (2) Felt a little bit frustrated or upset, (3) Felt somewhat frustrated or upset, and (4) Felt frustrated or upset a lot... Table 3: Factor 5—psychosocial. G.1 Frustrated or upset, G.2 Annoyed or angry, G.3 Felt like burden on others, G.4 Felt sad or depressed, G.5 Felt socially isolated, G.6 Concerned about safety at home, G.7 Worried, G.8 Rely on others, G.9 Visits with friends or family* | |
| Horowitz and Reinhardt (1998) | Not clear | Not identified | *Again, it must be stressed that the AVL Scale is intended to serve as one outcome measure in evaluation studies, to be used in conjunction with both global measures of psychosocial well-being and with measures of the acquisition of skills… With the aging of the population, age-related vision loss will affect increasing numbers of older people, which makes it imperative to gain a greater understanding of the crucial role that vision plays for the functional and psychosocial well-being of older people* | |
| Nyman et al. (2012) | Used synonymously with emotional well-being  Psychological well-being:  Adaptation/Adjustment Mental health Stress Depression Anxiety Negative affect/emotions (worry, anger, fear, frustration) Self-worth  Fear of further vision loss  QoL Social well-being:  Social functioning  Social support Family relations Loneliness Interpersonal relationships | Various | *The inclusion criteria were that papers were in press or published in a peer-reviewed academic journal, reported an empirical study that recruited older people (mean age or at least two thirds of the sample aged 60+) with an irreversible visual impairment, concerning perceived psychosocial well-being or perceived inhibitors/facilitators to psychosocial adjustment to vision loss. The term "psychosocial" referred to emotional well-being that would represent psychological constructs such as depression, anxiety, mental health and quality of life and social constructs such as social functioning, social support and loneliness...Visual impairment had a profound impact on adults' psychosocial well-being. Psychological well-being was reduced with the onset of depression (n=4), low self-worth (n=8), and fear of further vision loss (n=9), and social well-being was reduced by difficulties in social functioning (n = 5) and social isolation (n=5). Table 1. Keywords used for the literature search strategy. Psychosocial well-being: adaptation, psychological; or exp mental disorders; or stress, psychological; or exp depression; or exp emotions; or exp mental health; or exp quality of life; or exp sickness impact profile; or exp social behavior; or exp social environment; or exp family relations; or psychosocial.af; or depress*/anxiety/anxious/worry/anger/fear/frustrat*.af; or quality oflife.af; or emotion*.af; or social*/family/lonel-/ interpersonal/friend*/adjust*.* | |
| Burton et al. (2016) | Not identified | Qualitative interviews | *In addition, higher levels of vision impairment are associated with poorer general health [5] and psychosocial wellbeing.[6]* | |
| Brown et al. (2009) | Not identified | Not identified | *Vision impairment associated with diabetic retinopathy (DR) impacts on an individual’s functioning, psychosocial, physical and financial wellbeing.* | |
| Tallouzi et al. (2020) | Psychosocial adjustment:  Social anxiety  Acceptance of the disease  Changing personal items  Autonomy  Independence  Social reaction  Role disruption | Focus groups interviews | *Various threats to psychosocial well-being were discussed in the focus groups, for example, anxiety related to social reactions towards the person with uveitis, or a feeling of loss of autonomy and independence… Table 4: Components of psychosocial adjustment. Threats to psychosocial well-being – Definition: Things that indicate that individuals are having difficulty with psychosocial adjustment to uveitis or going through a process of adjustment, for example, social anxiety, acceptance of the disease, social reaction, changing personal items, autonomy and independence. Examples: Grief for losses incurred, for example, vision/sight loss. Lack of acceptance of the disease and adjustments to life required. Lack of predictability of the disease and impacts-related uncertainty regarding the future. Anxiety related to perceived or actual social reactions to the person with the disease. Feeling of dependence on others and loss of autonomy. Sense of role disruption, for example, work, family. Need to change things that are components of self-image, for example, unable to wear make-up, unable to wear items of clothing that are tied to self-image* | |
| Dillon et al. (2021) | Not identified | Not identified | *Adjustment and/or acceptance of vision loss has been shown to increase psychosocial wellbeing and use of services* | |
| Kahaly et al. (2002) | QoL  Vitality  Social functioning  Emotional role limitations  Mental health: including anxiety and depressive attacks | SF-36 | *To assess the impact of TAO on psychosocial well-being using a general self-reporting questionnaire, we performed a prospective descriptive study on 102 consecutive patients... General quality of life was assessed with the medical outcome 36-item short form health survey (MOS, SF-36)… 5. Vitality (4 items): extent of energy and/or tiredness and exhaustion. 6. Social functioning (2 items): extent of the limitation in normal social activities because of emotional problems. 7. Emotional role limitations (3 items): extent of the limitation in work and other daily activities due to emotional strain. 8. Mental health (5 items): overall mental health including depressive or anxiety attacks. The first four and the second four scales can be grouped according to the aspects of the physical and psychosocial quality of life.* | |
| Nyman et al. (2010b) | Depression/Mental health  Anxiety  QoL  Social functioning  Social support  Loneliness  Complex visual hallucinations  Life satisfaction Empowerment Independence | Various | *Aim: To review the evidence for the presence of lower levels of psychosocial well-being in working-age adults with visual impairment and for interventions to improve such levels of psychosocial well-being… and provided statistical data on one or more of seven psychosocial outcome measures: depression/mental health, anxiety, quality of life, social functioning, social support, loneliness or the experience of complex visual hallucinations... Table 2. Categorisation and number of outcome measures reviewed. Outcome variables: Depression/mental health, Anxiety, Quality of life, Social functioning, Social support… Three studies investigated psychosocial outcomes of rehabilitation and interventions…Rehabilitation can also focus life goals on relationships, which are associated with greater satisfaction with life (r=0.21) and lower levels of depressive symptoms (r=0.27). A study observed that those with visual impairment in extended-employment programmes reported greater satisfaction (MD=+47.33%) and empowerment/independence (MD=+49.33%)…* | |
| Mitchell and Bradley (2006) | Not identified | Qualitative interviews | *Wong et al [77] said that the most striking feature of in-depth interviews in a qualitative Australian study of people with MD relating to the participants' psychosocial well-being was the importance of 'understanding' the condition.* | |
| Lehane et al. (2017a) | Mental health  Depression  Anxiety  Loneliness  QoL  Self-confidence  Self-esteem Social support | Rand Mental Health Index  Beck Depression Inventory (BDS) CES-D  DSM-IV  GDS  State-Trait Anxiety Inventory (STAI)  8-item ABS  Revised UCLA Loneliness Scale  Social Support Questionnaire  Perceived Social Support Scale  Rosenberg Self-Esteem Scale  Focus group and interviews | *Psychosocial wellbeing. Using data from 418 older couples, it was found that those married to an individual with visual impairments hold a risk for lower psychological and social wellbeing, and higher levels of depression. Cross-sectional studies have also suggested an association between one spouse’s vision loss and their partner’s quality of life and depressive symptomology... Conducting interviews with 34 persons diagnosed with age-related macular degeneration, McCloud, Khadka, Gilhotra, and Pesudovs (2014) revealed that maintaining self-confidence was an issue among married participants, reportedly linked to their inability to contribute to shared, home-based daily tasks… Table 2. Articles investigating couples’ experiences of vision loss. Revised UCLA Loneliness Scale; Social Support Questionnaire Married participants reported higher social support and less loneliness… Rand Mental Health Index; Beck Depression Inventory; Rosenberg Self-Esteem Scale; Perceived Social Support Scale; Family Assessment Device Vision loss is reported as a risk for separation, decrease in affection, and communication issues for couples… State-Trait Anxiety Inventory; Beck Depression Inventory Spouses of those with retinal degeneration reported highest depression scores… CES-D; The Quality of Marriage Index Vision loss predicted spouse depression. Poorer marital quality related to depression… 12-item diagnostic criteria for a major depressive episode DSM-IV; 8-item Bradburn Affect Balance Scale; Self-made marital quality and unhappiness items Spouse vision impairment negatively affected partner depression, wellbeing, social participation, and marital quality… Caregiver Burden Scale; Geriatric Depression Scale Burden was significantly related to depression. No differences were found between sensory loss groups and controls on depression scores.* | |
| Lang and Brooks (2015) | Not identified | Qualitative interviews | *The opportunity and ability to continue to enjoy leisure occupations has been identified as a key determinant of psychosocial well-being following the onset of sight loss in later life… This Interpretive Phenomenological Analysis (IPA) study employed semi-structured interviews to explore how four older-age women with sight loss in the United Kingdom experienced and made sense of participating in audio book groups. Four interrelated themes emerged; sharing occupation with others, friendship and belonging, the role of the librarians, and the contrast with experiences beyond the group… Research supporting this belief has started to demonstrate the opportunity and ability to maintain participation in meaningful occupation as a key determinant of psychosocial well-being in older adults with sight loss* | |
| Stevelink et al. (2015b) | Depression  Self-esteem  Social interaction  Fear of further sight loss | PHQ-9 | *Impaired psychosocial well-being was reflected by the onset of depression, decreased self-esteem, fear of further deterioration of sight and difficulties in social interaction.* | |
| Orr (1991) | Not identified | Not identified | *Loss of unspoken communication has a considerable impact on psychosocial well being…* | |
| Smedema and McKenzie (2010) | Social support  Sense of well-being | Personal Resource Questionnaire-2000 (PRQ-2000)  SWBI | *The relationship between Internet use and psychosocial well-being of individuals with visual impairment has yet to be investigated. Therefore, the purpose of this study is to address the following research question: What is the relationship between amount and type of Internet use and perceived social support and sense of well-being in individuals with visual impairments?... The instruments used in this study included a demographic questionnaire, an Internet use questionnaire, the Personal Resources Questionnaire – 2000 (PRQ-2000), and the Sense of Well-Being Inventory (SWBI)… The PRQ-2000 [38] is a measure of perceived social support... The SWBI is a subjective well-being measure developed specifically for people with disabilities* | |
| Khoo et al. (2019) | Component of HRQoL/VRQoL  Depression  Anxiety  Mental health  Distress (vision- and diabetes-specific) | e.g.  SF-12 (mental health composite score)  SF-36 (mental health composite score)  NEI-VFQ  PHQ-9  DSM | *In this paper, we have used the term “psychosocial” as an umbrella term to capture a wide range of potential psychosocial factors including depression, depressive disorder, anxiety, vision-specific distress, diabetes-specific distress and emotional and social well-being… In this systematic review of the relationship between DR and psychosocial functioning, we found that DR/DME and related visual impairment, especially in more severe stages of DR, were significantly cross sectionally and longitudinally associated with poorer psychosocial outcomes, including higher levels of depression, anxiety and worse scores on mental health domains of health- and vision-related QoL questionnaires…. Our findings support the need for interventions to improve psychosocial well-being in patients with DR and also highlight the importance of prevention* | |
| Ong et al. (2017) | Depression  Fear of further vision loss  Self-worth | Not identified | *A recent meta-synthesis found that VI had a profound effect on adults’ psychosocial well-being, including fear of further vision loss, feelings of depression and low self-worth.2* | |
| Emotional well-being | | | | |
| Study | **Indicator** | **Measure** | **Quote** | |
| Richards et al. (2010) | Not identified | Focus group interviews | *This paper presents a critical analysis of the tourism encounters of individuals with vision problems and the positive impacts these can have on their emotional well-being, as well as the challenges they encounter whilst travelling… Themes: Experiencing Tourism with a Visual Impairment; Not Just About Vision—Embodied Tourism Encounters; Inhospitable Tourism Spaces—The Need for Awareness Training for Hospitality Staff (Similarly, hotel restaurant menus are a constant source of frustration and embarrassment.); Navigating Tourism Environments—Moments of Anxiety and Fear* | |
| Assi et al. (2021) | Component of QoL  Thoughts  Emotions  Emotional states  Trauma  Depressive symptoms  Shock  Fear  Panic  Distress  Helplessness  Frustration  Feeling powerless  Despair  Vulnerability  Fatigue  Isolation  Confidence | Qualitative interviews | *The systematic reviews of qualitative studies assessed emotional well-being and daily functioning among older adults with vision impairment7 and age-related macular degeneration (AMD)51 and children and adults with mendelian eye conditions,53 including retinitis pigmentosa specifically in a second review… Table 3. Quality of life outcome or measure: Emotional well-being: Diagnosis of vision impairment was identified as a traumatic event in 8 studies. An array of emotions was reported around the time of diagnosis, including feelings of shock, fear, panic, distress, helplessness, and frustration… Some accepted the diagnosis, while others felt powerless and in despair. Negative thoughts and depression symptoms were not confined to those with the most severe cases… The diagnosis of retinitis pigmentosa was commonly accompanied by shock, negative emotional states, and a loss of confidence. Participants reported fatigue, fear, isolation, and vulnerability as they coped with the disease and dealt with their own judgements and perceived stigma* | |
| Luu et al. (2020) | Anxiety  Depression | CES-D  HADS | *A more patient-centric model of low vision care could include using additional instruments such as the Centre for Epidemiologic Studies Depression Scale (CES-D) or Hospital Anxiety and Depression Scale (HADS) for emotional wellbeing,92...* | |
| Rees et al. (2015) | Anxiety  Depression  Stress | DASS | *Secondary outcomes emotional well-being, self-efficacy and adaptation to vision loss were measured using the depression, anxiety, stress scale (DASS), general self-efficacy scale (GSES), and short form adaptation to age-related vision loss scale (AVL12)* | |
| Bell and Foley (2021) | Not clear | Qualitative interviews | *In foregrounding the complex biographical temporalities of such experiences, this paper cautions against overly simplistic or Romantic tropes of what nonhuman nature can do for happiness and emotional wellbeing and for whom.* | |
| Jackson et al. (2017) | Coping  Frustration | IVI – Emotional well-being subscale | *The IVI is a validated 28-item questionnaire designed to identify vision-specific limitations in activities that affect patients’ quality of life. The 28 questions fall into three domains: mobility and independence (mobility), emotional well-being (well-being), and reading and accessing information (reading)… The IVI well-being subscale includes questions about how much the subject perceives their vision to impact their ability to cope and emotions such as frustration* | |
| Scuderi et al. (2011) | Loneliness  Happiness  Suicidal thoughts | Emotional Well-being Scale (EWS) | *We did not find strong evidence supporting the fact that measures of visual impairment were linked to emotional well-being and depression… The Emotional Well-being Scale (EWS) is a self-rating measure composed of 21 dichotomous items (45); statements are worded in positive and negative form (e.g., ‘Almost every day I felt lonely’, ‘I never felt lonely’). Furthermore, some items are worded to assess whether the individual experiences a current condition of well-being (e.g. ‘Nearly always I felt happy’), and others are worded to assess whether the individual experiences current distress (e.g. ‘I sometimes thought that for me, my family, or for people around me it is better to be dead’).* | |
| Castle et al. (2021) | Not identified | Not identified | *Art as activity may offer benefits to the wider UK veteran population relating to holistic well-being needs, the promotion of good mental health, and the maintenance of active social and leisure lives, but this has not been explored. This is surprising given the cognitive, social, emotional and physical well-being benefits which have been associated with engagement with art activities in general populations* | |
| Ang et al. (2020) | Component of VRQoL  Not identified | GOAT | *Moreover, unlike the LiGHT study which focused on generic health-related QoL and glaucoma-specific symptoms and visual functioning alone, our study measured a range of other glaucoma-specific QoL outcomes, including emotional well-being, concerns, social, costs and treatment inconvenience.* | |
| Gothwal et al. (2013) | Component of VRQoL  Loneliness  Isolation  Feeling like a burden/nuisance  Embarrassment  Sadness  Feeling low  Stopped doing things you want to do  Coping  Frustration  Annoyance  Eyesight interfering with life  Worry (about further vision loss) | IVI - Emotional well-being subscale | *Rasch analysis demonstrated the validity of the IVI to assess VRQoL through two subscales: vision-specific functioning (VF) and emotional well-being (EWB)… The construct of VRQoL encompasses vision functioning (VF), symptoms, EWB, social relationships, concerns, and convenience as they are affected by vision… Figure. Felt lonely or isolated, Felt like a nuisance or burden, Felt embarrassed, Felt sad or low, Stopped you doing things you want to do, Made you concerned or worried about coping, Felt frustrated or annoyed, Interfered with life, Worried about eyesight getting worse* | |
| Fenwick et al. (2016) | Component of VRQoL  Not identified | IVI – Emotional well-being subscale (Chinese version) | *The Chinese IVI initially demonstrated multidimensionality and was split into three scales: ‘Reading and Accessing Information’; ‘Mobility and Independence’; and ‘Emotional Well-being’.* | |
| Jackson et al. (2019) | Depressive symptoms  QoL  Life satisfaction  Loneliness | CES-D  CASP  SWLS  3-item revised UCLA Loneliness Scale | *Emotional Well-being. Our primary outcome for analyses of well-being was depressive symptoms. Secondary outcomes were QOL, life satisfaction, and loneliness. Depressive symptoms were assessed with an 8-item version of the Center for Epidemiologic Studies Depression Scale…Quality of life was assessed with the CASP-19 (control, autonomy, self-realization, and pleasure),46 a scale designed to measure QOL in older people… Life satisfaction was assessed with the Satisfaction With Life Scale…*  *Loneliness was measured using the 3-item Revised UCLA Loneliness Scale* | |
| Gan et al. (2019) | Component of VRQoL  Not identified | IVI – Emotional well-being subscale | *In this study, we investigated the relationship between the severity of DR and three specific VRQoL domains, namely ‘Reading and Accessing Information’, ‘Mobility and Independence’ and ‘Emotional Well-Being’, using the Impact of Vision Impairment (IVI) questionnaire...* | |
| Soubrane et al. (2007) | Anxiety  Depression | HADS | *The HADS includes 14 items, of which 7 assess anxiety and 7 assess depression; subscale scores range from 0 to 21, with higher scores representing more symptoms and poorer emotional well-being.* | |
| Lotery et al. (2007) | Anxiety  Depression | HADS | *The HADS includes 14 items, seven assessing anxiety and seven assessing depression; subscales scores range from 0 to 21, with higher scores representing more symptoms and poorer emotional well-being.* | |
| Abels (2001) | Life adjustment:  Positive affect  Negative affect  Life satisfaction | SWLS  MAACL-R6 | *Although the literature suggests that there are many ways to define the construct of emotional well-being and adjustment, there appears to be a distinct affective and cognitive element in this process (…). The affective aspect simply distinguishes between positive and negative affect. The cognitive component deals with life satisfaction* | |
| Thurston et al. (2013) | Not identified | Not identified | *There is compelling evidence to suggest that acquired sight loss negatively impacts on emotional well-being.* | |
| Hackney et al. (2013) | Not identified | 25-item NEI-VFQ | *The National Eye Institute Visual Function Questionnaire-25 (VFQ-25… The VFQ-25 measured vision-related QOL, the influence of visual disability on emotional well-being, and social functioning.* | |
| Finger et al. (2014b) | Component of VRQoL  Not identified | IVI-VLV | *Measurement of QoL involves a person self-rating the impact of their visual impairment (if any) or ocular condition on various components, such as mobility, activity limitation, reading and accessing information, or emotional well-being, using a set of relevant and validated items (questions)… Based on the PCA, confirmed by a factor analysis, the scale was split into two subscales, the Emotional Well-Being (EWB) and Activities of Daily Living, Mobility, and Safety (ADLMS) subscales… This has been shown to influence coping rates of depression, and emotional well-being.* | |
| Runjic et al. (2018) | Component of VRQoL  Not identified | IVI – Emotional well-being subscale | *The individuals’ quality of life has to be estimated through physical health, material, social and emotional well-being… In order to assess the quality of life, we used the Impact of Vision Impairment questionnaire... Difference analysis was performed for each of the three areas of the questionnaire (reading and accessing information, mobility and independence, and emotional well-being).* | |
| Gan et al. (2020b) | Not identified | IVI – Emotional well-being subscale | *Outcomes included presenting distance and near visual acuity (PNVA); Rasch-transformed Reading, Emotional and Mobility scores from the Impact of Vision Impairment questionnaire;… We found a statistically significant within-group decrement in Emotional well-being in the usual care group at 6months… Between-group analyses revealed a significantly greater improvement in Emotional well-being in ROC participants compared with usual care* | |
| Nyman et al. (2012) | Used synonymously with psychosocial well-being  Anxiety  Depression  Mental health  Quality of life  Social functioning  Social support  Loneliness | Not identified | *The term "psychosocial" referred to emotional well-being that would represent psychological constructs such as depression, anxiety, mental health and quality of life and social constructs such as social functioning, social support and loneliness* | |
| Nyman et al. (2010a) | Anxiety  Depression  Mental health  Suicide risk  Self-harm  QoL  Shock  Fear  Frustration  Grief  Vulnerability  Self-doubt  Confidence  Empowerment  Attitude to life  Social functioning  Social contact  Social activity  Social support  Loneliness  Exclusion  Social isolation  Social participation (leaving the house)  Physical symptoms (headaches, nausea, dizziness, insomnia, and appetite loss) | Not identified | *For emotional well-being we included psychological indicators (depression, mental health, anxiety, and quality of life) and social indicators (social functioning, social support, and loneliness), as well as studies that evaluated rehabilitation and other services or interventions that aimed to improve emotional well-being… The studies indicated that people with sight loss can report reduced emotional well-being in the form of shock, fear, depression, grief, vulnerability, and self-doubt and lack of confidence… A survey of 400 people aged 55+ with sight loss revealed that most participants responded positively in regard to emotional well-being, with 46 percent reporting a positive attitude to life and 38 percent feeling that ‘life was alright’… Social well-being. The studies reviewed suggested that adults with sight loss are not satisfied with their level of social contact and activity, as people with sight loss have reported feelings of loneliness, exclusion, and social isolation (…). A quarter of one sample did not feel sufficiently in touch with people, and over half did not feel engaged with their community and wanted to go out more often… Whether these measures accurately reflect emotional well-being is for future research to discover… Similarly, in 2008 it was reported that 36 out of 38 clients had made a statistically and clinically significant improvement in emotional well-being post-counselling, including a reduction in unexplained physical symptoms (headaches, nausea, dizziness, insomnia, and appetite loss) (…), and a reduction in risk of suicide (…) and self-harm…The intervention demonstrated feelings of empowerment and a decrease in feelings of isolation post-counselling. Participants reported insight into their fears and frustrations associated with sight loss and an appreciation for the opportunity to talk to people other than family members, both of which helped them come to accept their sight loss* | |
| Peters et al. (2013) | Used synonymously with psychological well-being  Psychological distress:  Anxiety  Depression  Phobic anxiety (fear in public and open places)  Somatisation (perception of bodily dysfunction)  Paranoid ideation (projection of hostility, suspiciousness, fear of loss of autonomy)  Hostility  Obsessive–Compulsive  Interpersonal sensitivity  Psychoticism | Brief Symptom Inventory (BSI) | *The aim of this study was to assess the emotional wellbeing of patients undergoing implantation of a subretinal implant… The Brief Symptom Inventory (BSI), a short self-report scale of nine primary symptoms, was used to assess reaction to the psychological distress related to study participation… We chose the BSI as an additional questionnaire to accompany the patients in the Retinal Implant Study, because we wished to obtain a measure of the psychological wellbeing (and hence a symptom inventory). This questionnaire provides patient-reported data on 53 items, used to measure nine primary symptom dimensions: Somatization (SOM); Obsessive–Compulsive (O–C); Interpersonal Sensitivity (I-S); Depression (DEP); Anxiety (ANX); Hostility (HOS); Phobic Anxiety (PHOB); Paranoid Ideation (PAR) and Psychoticism (PSY) [8]… At the end of this study, before the retinal implants were extracted, the average results from nine patients showed an increase in score, for four of the nine symptoms measured in the BSI (see Table 1), indicating a small but not significant worsening of emotional wellbeing: The subjects showed an increase in their perception of bodily dysfunction (Somatization), fear in public and open places (Phobic Anxiety), projection of hostility, suspiciousness, fear of loss of autonomy (Paranoid Ideation) and Hostility in thoughts, feelings and actions.* | |
| Prem Senthil et al. (2017) | Emotional and psychological challenges:  Anxiety  Depression  Anger  Frustration  Shock  Optimism  Hopefulness  Worry about further vision loss  Fear (of repeated eye injections and laser treatment) |  | *Theme 2: Participants with both hereditary retinal diseases and acquired retinal diseases faced emotional and psychological challenges (Emotional well-being):… The commonly expressed emotional comments in the hereditary retinal diseases group were frustration, anxiety, shock, depression, and anger... In contrast to participants with hereditary retinal diseases, participants with acquired retinal diseases were more optimistic about their eye condition. They believed that treatment would make their eye condition better. Participants whose vision had not improved with treatment worried about losing their sight and involvement of the other eye... They feared the repeated eye injections and laser treatments… Table 2: Emotional well-being (Feel frustrated, Feel anxious, Feel hopeful, Feel shocked)* | |
| Fenwick et al. (2020) | Component of VRQoL  Not identified | Various | *Indeed, glaucoma, particularly at the vision-threatening stages, can have a substantial impact on several patient-reported outcomes, including multiple domains of quality of life (QoL) (…), such as emotional well-being (…) and visual functioning (…)… However, while such measures may overcome some of the bias associated with self-reported assessment of ability levels (…), they are limited in scope to measure functioning (e.g. activities of daily living and mobility) and cannot tap into other aspects of QoL such as symptoms, concerns, emotional well-being and loss of social life…* | |
| Bambara et al. (2009) | Not identified | Not identified | *A longitudinal study by Strawbridge, Wallhagen, and Shema (2007) found that the spouses of individuals with vision loss had an increased risk of poorer physical and emotional well-being over five years and that the characteristics of one had an impact on the other.* | |
| Fenwick et al. (2011) | Component of VRQoL  Not identified | e.g. Retinopathy Dependent QoL (RetDQoL) | *We focus on vision-related QoL, a complex concept that encompasses functional ability, symptoms, emotional well-being, social relationships, concerns and convenience as they are affected by vision… A DR-specific QoL tool (the Retinopathy Dependent QoL or RetDQoL)6 has recently been developed and validated.16 It contains 26 items sampling various aspects of QoL including functional ability, social, family and working life, emotional well-being and self-care ability.* | |
| Runjic et al. (2017) | Component of QoL  Feeling sad/low  Frustration/annoyance  Feeling like a burden/nuisance  Embarrassment | IVI – Emotional well-being subscale | *To gather information about the effect of visual impairment on QoL, we used the Impact of Vision Impairment (IVI) questionnaire… This questionnaire measures the effect of visual impairment on QoL in three areas: reading and accessing information, mobility and independence, and emotional well-being… Consequently, the emotional well-being results are expected and confirmed by this study. Constant confrontation with everyday life tasks that are performed under conditions of impaired vision affects the emotional state of women as measured in the variables Have you felt sad or low because of your eyesight?, Have you felt frustrated or annoyed because of your eyesight?, Have you felt like a nuisance or a burden because of your eyesight? and Have you felt embarrassed because of your eyesight?* | |
| Rees et al. (2019) | Component of VRQoL  Feelings:  Frustration  Annoyance | IVI-RC – Emotional well-being subscale | *Specifically, the functional and emotional impact of VI have been found to be key predictors of depressive symptoms in community living adults with VI… VRQoL was measured using the Impact of Vision Impairment for Residential Care (IVI-RC) questionnaire… The last eight items comprise the Emotional Well-being scale. Participants are asked to ‘Think about how your eyesight has made you feel in the past month’ (e.g., ‘Have you felt frustrated or annoyed because of your eyesight?’)...* | |
| Hooper et al. (2007) | Component of VRQoL  Not identified | IVI – Emotional well-being subscale | *To investigate if cataract surgery improves overall and specific areas of quality of life (QoL) in patients with early age-related macular degeneration (AMD) using the impact of vision impairment (IVI) questionnaire…Recently, the dimensionality of the 28-item IVI was assessed and a 3-subscale structure possessing interval level measurement characteristics was confirmed.27 The three subscales were “mobility and independence”(11 items), “emotional well-being”(8 items), and “reading and accessing information” (9 items)* | |
| Mojon-Azzi et al. (2008) | Not clear | Survey of Health, Ageing and Retirement in Europe (SHARE) | *Using binary logistic regressions on data from the 2004 Survey of Health, Ageing and Retirement in Europe (SHARE), we analysed the association between self-reported corrected vision in general, corrected distance vision and corrected reading vision on 11 variables capturing emotional well-being, future hopes and perspectives, and concentration on daily activities… The effect of visual impairment was examined for the following outcome variables: (1) having ever suffered from depression, (2) having felt sad or depressed in the month before the interview, (3) having cried in the month before the interview (tearfulness), (4) lack of enjoyment, reflected by no mention of enjoying an activity recently, (5) hopelessness, based on no mention of hopes for the future, (6) wishing to be dead, measured by mention of suicidal feelings, (7) fatigue, (8) irritability, (9) having less interest than usual in everyday experiences, (10) having difficulty concentrating on reading and (11) having difficulty concentrating on entertainment, for example, a film or television or radio programme.* | |
| Gleeson et al. (2017) | Emotional distress:  Embarrassment  Frustration  Loneliness  Feeling like a burden | IVI – Emotional well-being subscale | *The aim of this study was to explore the impact of lessons in the Alexander technique on emotional well-being and to investigate the prevalence and determinants of depressive symptoms in this study population… The emotional subscale from the Impact of Vision Impairment Profile (IVI)30 uses eight items to capture the degree to which the participant perceives that their visual impairment causes emotional distress... There was a trend for less visual impairment-related emotional distress in the intervention group. This may be attributed to improvements to the participant’s mobility, which indirectly impacted emotional well-being… Collectively, these changes can lead to negative emotional well-being due to embarrassment, frustration, loneliness and feeling like a burden, all of which are correlated with depression* | |
| Menon et al. (2020) | Not clear | Case notes | *Three case vignettes, chosen by the ECLO, highlighted the positive impact of the ECLO with respect to emotional support, practical advice and as a point of contact ensuring continuity of care, also during the COVID-19 pandemic… Ms A, registered sight impaired, was living in unsuitable accommodation, had financial difficulties, struggled to mobilise safely and was socially isolated… Mr B experienced severe visual loss of sudden onset and hence was referred to the ECLO to discuss registration. The ECLO felt that Mr B was very distressed, overwhelmed and unable to internalise the information given… Ms C, a sight-impaired patient, was concerned about a deterioration in her vision, and required an appointment, however, due to pandemic-related staffing issues, was unable to contact the clinical team… This study illustrates the significant impact of an ECLO on patient well-being and highlights the need for this crucial role within ophthalmology services. ECLOs are a core patient service within ophthalmology and have a significant impact on patients’ emotional and physical well-being and ultimately their quality of life.* | |
| Fenwick et al. (2009) | Component of VRQoL  Psychological distress | Nursing Home Vision-Targeted Health-Related Quality-of-Life questionnaire (NHVQoL) | *Vision-related QoL was measured with the 57-item NHVQoL,… The questionnaire was designed to evaluate the impact of visual impairment and eye disease on QoL and mental health in older nursing home residents and to assess the affect of psychosocial and eye care interventions in nursing home settings. It consists of nine subscales focusing on general vision (6 items), reading (3 items), ocular symptoms (9 items), mobility (7 items), psychological distress (10 items), activities of daily living (6 items), social activities and hobbies (8 items), adaptation and coping (2 items), and social interaction (6 items).The QoL aspects most affected by vision loss were related to general vision, reading, hobbies, emotional well-being, and social interaction… With the exception of the ocular symptoms subscale, there was a consistent overall deterioration in the remaining seven subscales: general vision, reading, activities of daily living, mobility, hobbies, psychological, and social interaction with a worsening of both distance and near vision (P < 0.05)* | |
| Man et al. (2018) | Component of VRQoL  Not identified | IVI – Emotional well-being subscale | *The IVI has 28 items comprising an overall score and three subscales of VRQoL, namely reading and accessing information, mobility and independence and emotional well-being… We observed a differential short-term improvement to VRQoL after KPro implantation with a significant impact on emotional well-being, which may not be fully explained by visual improvement alone.* | |
| Holmes et al. (2018) | Not identified | Not identified | *In 2012, Nyman et al. undertook a systematic review of 17 qualitative studies of emotional well-being and adjustment to vision loss in later life from the USA, Australia, and European countries* | |
| Vu et al. (2005) | Not clear | Health habits interview | *The health habits interview included the outcomes of whether the participant did not feel full of life in the past month at all or had health/emotional problems that extremely interfered with normal social activities in the past month… Tables 2-4 items:* *Fell at home, Fell when away, Ever fell, Hip replacement surgery, Nursing home placement, Using supplied meals, Getting help with chores, Dependency, Health/emotional problems, Did not feel full of life, Reading the telephone book, reading newspaper, Watching television, Seeing faces, Doing other activities… Non-correctable unilateral vision loss was associated with issues of safety and independent living while non-correctable bilateral vision loss was associated with nursing home placement, emotional wellbeing, use of community services, and activities of daily living… On the other hand, non-correctable bilateral visual loss was not associated with falling, but it was associated with dependency, nursing home placement, emotional wellbeing, and visual tasks* | |
| Nguyen et al. (2018) | Not identified | IVI-VLV – Emotional well-being subscale | *Before activating their Aira subscription, patients were contacted by phone and were verbally administered the IVI-VLV questionnaire10 (Supplementary Fig. S1). All patients were English-speaking and were asked to complete the questionnaires, with questions divided into four sections: activities of daily living, mobility, safety, and emotional well-being.* | |
| Gleeson et al. (2014) | Not identified | IVI – Emotional well-being subscale | *Secondary outcome measures will be balance, mobility, social participation and emotional well-being at 3 and 12 months… Mood will be assessed with the Geriatric Depression Scale,43 the Positive and Negative Affect Scale,44 and the Emotional Well-Being subscale of the Impact of Vision Impairment Scale45 at baseline, 3 and 12 months* | |
| Engel et al. (2000) | Not identified | Not identified | *Specifically, cross-sectional studies have found that the loss of vision is associated with difficulties in performing basic activities of daily living (…), with health problems and the perceptions of poorer health status (…), with psychological and emotional well-being (…), and with social interactions…* | |
| Khadka et al. (2015) | Component of VRQoL  Not identified | Item bank | *Therefore, the purpose of this study was to identify*  *content for a new, glaucoma-specific, quality-of-life (QOL) item bank… The domains were activity limitation, mobility, visual symptoms, ocular surface symptoms, general symptoms, convenience, health concerns, emotional well-being, social well-being and economic issues… Interestingly, the final item pool bears the majority of the items under “activity limitation”, “symptoms (visual, ocular surface and general)” and “emotional well-being”.* | |
| McKean-Cowdin et al. (2010) | Component of HRQoL  Not identified | 25-item NEI-VFQ | *Vision-targeted HRQOL was assessed by the NEI-VFQ-25.2, 21 The survey measures the influence of visual impairment and symptoms on generic health domains such as emotional well-being and social functioning, in addition to task-oriented domains related to daily visual functioning* | |
| Brown et al. (2009) | Component of QoL  Not identified | e.g.  25-item NEI-VFQ  IVI | *The IVI questionnaire assesses the extent to which vision impairment interferes with an individual’s participation in activities… Confirmatory factor analysis and Rasch analysis transformed the original 32-item questionnaire into 28 items and confirmed three subscales: reading and accessing information, mobility and independence, and emotional wellbeing… The NEI VFQ was designed to assess the impact of vision problems on physical functioning, emotional wellbeing and social functioning… … QoL instruments that demonstrate an appropriate domain structure can provide easily interpretable results that can indicate the effects of the treatment on various aspects of QoL, such as reading or emotional wellbeing, as well as overall QoL.* | |
| Marques-Brocksopp (2014) | Resilience  Empowerment  Self-esteem  Self-control  Hopefulness/Optimism  Coping  Immediate satisfaction  Happiness  Mood | Qualitative interviews | *Emotional well-being... This was divided into the subthemes of (1) resilience and empowerment and (2) immediate satisfaction. Resilience and empowerment. The participants also spoke of a perceived improvement in their self-esteem and self-control…This sense of empowerment through mindfulness practices was also described in relation to an increased sense of hope and optimism. By feeling more optimistic and hopeful, some participants spoke of how they felt able to ‘cope’ or ‘manage’ difficult situations… Immediate satisfaction. Apart from provoking a sense of empowerment and resilience, which may be related to long-term eudemonic well-being, mindfulness was also identified with a more hedonic sense of satisfaction. This was described in terms of happiness or good mood* | |
| Selivanova et al. (2019) | Component of VRQoL  Not identified | IVI – Emotional well-being subscale | *The Impact of Vision Impairment is a vision-related quality-of-life questionnaire that has been previously validated in a low-vision population.11 The questionnaire has a total scale12,13 and three subscales: reading and accessing information (reading), mobility and independence (mobility), and emotional well-being (well-being).* | |
| Tallouzi et al. (2020) | Frustration  Anger | Focus group interviews | *Table 1: Outcome domains. 6) Psychological morbidity and emotional well-being – definition: Psychological and emotional morbidity that may occur in patients with PSIU - Items in domain: Depression; anxiety and stress; emotional well-being… Emotional well-being, including feelings of frustration and anger, for example, when patients were in pain, was also a component of this domain* | |
| Gothwal and Bharani (2015) | Component of VRQoL  Mental health | IVI – Emotional well-being subscale | *Vision-related QoL is a complex trait that encompasses visual disability, symptoms, EWB, social relationships, concerns, and convenience as they are affected by vision.32 We chose the IVI and VA LV VFQ-48… Although many activities were improved, EWB was much less likely to improve. One suggestion is that LVR programs might target mental health issues more explicitly. However, this also implies that specific LVR efforts targeted toward improvement in mental health/EWB of patients are reimbursed and that the persons providing these services have appropriate training.* | |
| Paudel et al. (2015) | Component of VRQoL  Happiness  Worry about seeing an enemy approach (safety)  Embarrassment  Frustration  Loneliness  Sadness  Feeling low  Feeling like a burden  Eyesight interfering with life  Worry (about further vision loss) | PNG-VS QoL | *Finally, the Rasch model demonstrated that the 17-item PNG-VS QoL instrument consisting of the 8-item activity limitation and 9-item emotional well-being subscales was a valid and psychometrically robust instrument for the assessment of impact of impaired vision on QoL… Table 1. Structure, item content and scales of the 41-item pilot vision-specific quality of life questionnaire. Well-being items: 27 I feel life is not worth living because of my eyesight, 28 My eyesight is absolutely fine, 29† I feel unhappy about my poor eyesight, 30† I am worried I may not see an enemy approaching because of my poor eyesight, 31† I feel embarrassed because of my eyesight, 32† I feel frustrated because of my eyesight, 33† I feel lonely because of my eyesight, 34† I feel sad or low because of my eyesight, 35† I feel like a burden because of my eyesight, 36† My poor eyesight interferes with my life, 37 I feel I would be happy if I could see well, 38† I worry about my eyesight getting worse, 39 My eyesight allows me to do the things expected of someone my age, 40 Overall, and including my eyesight, I consider myself to be very healthy for my age, 41 I am completely happy with my eyesight… †Represents the final item numbers retained in the instrument.* | |
| Sweeting et al. (2020) | Not identified | Not identified | *Outcome measures included a range of physical measurements, such as body fat percentage, blood pressure, body mass, waist circumference; physical activity/ fitness measures such as flexibility, daily step count, balance and muscle strength and endurance; and well-being measures including social and emotional well-being and depression… Table 2 Summary of outcome measures. Psychological well-being §Including measures of quality of life, anxiety, depression and emotional well-being* | |
| Bray et al. (2017) | Depression | WHO-5 | *The WHO-5 is a validated emotional well-being index, which is highly sensitive for screening depressive symptoms* | |
| Yildiz et al. (2021) | Mental health | SF-36 – Emotional well-being subscale | *Patients with a psychiatric diagnosis scored lower on physical functioning, role limitations due to emotional problems, energy/fatigue; emotional well-being, social functioning and pain subscales of the Short Form-36 (SF-36)… Table 3 Comparison of SF-36 scores with and without a psychiatric diagnosis according to SCID-1 in patients with keratoconus.* [SF-36 domain Emotional well-being labelled as ‘Mental health’] | |
| Mirandola et al. (2019) | Component of QoL  Not identified | Not identified | *Physical activity and sports also represent a viable strategy for improving the QoL defined as a personal sense of physical and mental health, social functioning and emotional well-being* | |
| Dev et al. (2014) | Not identified | Not identified | *Visual impairment (VI) affects physical, psychological, and emotional well-being, and social life as well… Visual function is important for an optimal orientation in functional and social life and has effects on physical, psychological, mental and emotional well-being* | |
| van der Aa et al. (2016) | Fear  Sadness  Frustration  Hopefulness  Peacefulness | IVI – Emotional well-being subscale  RAND-36 – Emotional well-being subscale | *Of interest: depressive symptoms, anxiety symptoms, and stress (DASS), emotional wellbeing (subscale IVI)… Of interest: emotional well-being/ response (five questions on a 4-point Likert scale on experiencing fear, sadness, frustration, hopefulness and peacefulness)… Of interest: emotional well-being (subscale of the RAND-36)* | |
| Clark et al. (2008) | Not identified | 25-item NEI-VFQ | *The VFQ-25 measures the influence of visual disabilities and visual symptoms on generic health domains such as emotional well-being and social functioning, and task-oriented domains related to visual functioning.* | |
| Adigun et al. (2014) | Not identified | Not identified | *Background: Visual function is important for optimal orientation in functional and social life, and has an effect on physical and emotional well-being… Visual function is important for optimal orientation in functional and social life, and has an effect on physical and emotional well-being* | |
| Rees et al. (2017) | Component of VRQoL  Frustration  Annoyance  Loneliness  Isolation  Worry (about further vision loss)  Coping (with daily life)  Feeling like a burden/nuisance  Eyesight interfering with life | Brief IVI – Emotional well-being subscale | *The Brief 15-item IVI can obtain valid and responsive measurement of VRQoL with half the items in the original and has potential to reduce respondent burden in QoL studies… Supplementary Table S1. The items in the original IVI and Brief IVI and corresponding subscales. 22 Have you felt frustrated or annoyed because of your eyesight (Item 10, EWB), 23 Have you felt lonely or isolated because of your eyesight (Item 11, EWB), 25 How often have you worried about your eyesight getting worse (Item 12, EWB), 26 How often has your eyesight made you concerned or worried about coping with everyday life (Item 13, EWB), 27 Have you felt like a nuisance or burden because of your eyesight (Item 14, EWB), 28 How much has your eyesight interfered with your life in general (Item 15, EWB)* | |
| Dagnelie and Kiser (2008) | Not identified | Developed questionnaire on complementary and alternative medicine (CAM) | *RP patients are using CAM and are experiencing some impact on vision and physical/emotional well-being… One item inquired about the motivations or reasons for using each of the CAM areas and the results are depicted in Figure 2. The respondents were able to select multiple answers and could choose from ‘physical well-being’, ‘emotional well-being’, ‘might help, can’t hurt’, ‘desire to fight RP’ and ‘to help with a disease other than RP’. Across each of the CAM areas, over one-half of respondents using CAM did so for physical and emotional well-being, while 63 per cent who tried acupuncture felt that it ‘might help, can’t hurt’… There is ample evidence linking CAM use with reduction of negative mood states, stress, anxiety, depression and fatigue for other types of chronic, disabling diseases. Our findings indicate that only a minimal proportion of patients did not feel that these areas were impacted by CAM use. This suggests that CAM met the patients’ expectations in terms of the potential to improve physical and emotional well-being…* | |
| Thetford et al. (2015) | Not identified | Qualitative interviews | *However, her over-reliance on the support provided by her husband may in fact have been contributing to her declining emotional wellbeing.* | |
| Fenwick et al. (2012a) | Mental health  Emotional reactions  Anxiety  Depression  Stress  Distress  Psychiatric symptomology  Somatization  Phobic anxiety  Emotional instability  Mood swings  Anger  Fear  Frustration  Guilt  Vulnerability  Worry  Loss of independence  Awkwardness  Irritation  Feelings of inferiority  Feelings of hostility  Feelings of being burdensome  Confidence  Self-esteem  Demoralization  Difficulty coping  Worthlessness  Helplessness  Uncertainty | Various | *By ‘emotional’, we encompass all emotional reactions to actual or threatened vision loss, such as fear, vulnerability, demoralization, worry, guilt, loss of confidence, dependence, uncertainty, anger, helplessness, worthlessness, stress and low self-esteem, as well as mental health disorders such as depression and anxiety… IMPACT OF DR ON EMOTIONAL WELL-BEING. A range of negative emotional reactions associated with DR have been identified in qualitative studies, including fear, depression, anger, mood swings, guilt, loss of independence, awkwardness, irritation, feelings of inferiority and hostility, feelings of being burdensome, loss of confidence and self-esteem, frustration, vulnerability, demoralization, difficulty coping, worry, worthlessness, vulnerability, anxiety and stress… Other studies have reported an association between DR and depressive symptoms,24,32,33,37 distress,24,32 psychiatric symptomology,35,41 anxiety,33,39 stress,24,40 somatization, phobic anxiety and worry,33 emotional instability,24 fear/worry36 and low self-esteem… Table 2. Literature review findings on the impact of DR on emotional well-being parameters.* | |
| Marques-Brocksopp (2012) | Mental health  Happiness  Optimism  Resilience  Self-esteem | Not identified | *‘Emotional wellbeing’ has been documented as being of paramount importance to the future of visually impaired children, for example (…), however what do we actually mean when we use this term?...*  *Although the term appears to be all-encompassing, visual impairment research has tended to look specifically at the concept of emotional wellbeing. Much has been written on the relationship between chronic illness and mental health outcomes, particularly in terms of ‘happiness’… A wealth of evidence exists to support the relationship between vision loss and functional disability, and subsequent negative effect on quality of life and emotional wellbeing (..). As a consequence of such research, there is an increasing awareness of the importance of ‘preventing’ negative mental health outcomes in the visually impaired population and ‘promoting’ emotional wellbeing, especially in children and young people… Fuelled by the current interest in wellbeing, significant links have been made between emotional wellbeing and constructs such as optimism, resilience and self-esteem* | |
| Zimdars et al. (2012) | Depression | CES-D | *Emotional well-being. In this final analysis we estimate what the effects of reported vision are on scoring in the lowest decile of the CES-D depression score controlling for the associations between poor vision and health, economic and social factors* | |
| Rees et al. (2007a) | Component of VRQoL  Not identified | IVI – Emotional well-being subscale | *To evaluate the effectiveness of a multidisciplinary low-vision rehabilitation program on quality of life evaluated by the Impact of Vision Impairment (IVI) instrument… After rehabilitation, significant improvements were recorded for the overall IVI score (P = 0.006) and two subscales: reading and accessing information and emotional well-being (P = 0.007 and 0.009, respectively).* | |
| Finger et al. (2014a) | Component of VRQoL  Not identified | IVI – Emotional well-being subscale | *The VRQoL as measured by the IVI using its 3 subscales: Accessing Information, Mobility, and Emotional Well-being.* | |
| Fenwick et al. (2012b) | Component of QoL  Sadness (Feeling depressed, sad, low, upset, miserable, devastated, raw, suicidal thoughts, loss of motivation, hopelessness, loss of pleasure in things, missing work or hobbies, loss of spontaneity)  Anger (Frustration, feeling annoyed, moody, agitated, cheated, oppressed, being left despondent)  Fear (Feeling frightened, stressed, scared, vulnerable, disconcerted, nervous, extra pressure)  Self-perception (Self-worth, self-image, self-confidence, feeling older, less competent, inadequate, less important, like a burden, non-productive, role disruption, embarrassment) | Focus groups and qualitative interviews | *We isolated nine key domains of QoL, namely visual symptoms, ocular surface symptoms, vision-related activity limitation, mobility, emotional well-being, health concerns, convenience, social, and economic (Table 2)… Table 2 –The nine QoL domains and sub-domains identified. Emotional: Sadness, Anger, Fear, Self-perception… Emotional well-being. We categorised patients‟ emotional responses about their DR below into sadness, anger, fear, and self-perception based on the tree-structure of emotions proposed by Parrot (2001).[41] Sadness (n=78). Several participants reported feeling depressed due to their DR and some even admitted to having suicidal thoughts. Others described feeling sad or low, upset, emotionally raw, miserable and devastated. Other negative emotions included „loss of motivation‟, „hopelessness‟, „loss of pleasure in things‟, ‟missing work or hobbies‟ and „loss of spontaneity‟… Anger (n=97). Almost all participants reported feeling frustrated because of the limitations imposed by their vision loss from DR (n=42). Many participants described feeling angry, annoyed, moody and agitated, especially in cases where their eye condition continued to worsen despite vigilant diabetic control. In these cases, participants often felt „cheated‟ and were left despondently pondering „why me?‟ A few participants also reported feeling oppressed because of a sudden loss of sight or gradually deteriorating vision… Fear (n=38). The time of diagnosis of DR was very frightening for participants. Many felt stressed about their vision and reported that it was ‟always on their mind‟. Others described feeling scared when unexpected events occurred such as sudden loss of vision from a haemorrhage. Several participants described situations in which they felt vulnerable, disconcerted or nervous. Others described feeling extra pressure to maintain their vision so they could continue to care for their family… Self-perception (n=47). Participants often described feeling a loss of self-worth, self-image or self-confidence. Many felt older, less competent, inadequate or less important because of their restricted role in the family or community. In contrast, reliance on family members made other participants feel burdensome and like non-productive adults. Several participants also felt embarrassed when they had to ask a stranger for help or made a social faux pas…* | |
| Fenwick et al. (2017) | Component of VRQoL  Not identified | IVI – Emotional well-being subscale (Chinese version) | *The reading, mobility and emotional well-being subscales of the impact of vision impairment (IVI) scale were validated using Rasch analysis and used as the main outcome measures and collectively referred to as VRQoL… The 32-item IVI questionnaire was used to assess VRQoL. The IVI has undergone extensive psychometric validation in the past,13 and our group has recently validated the Chinese version in the SCES using Rasch analysis.* | |
| Ratanasukon et al. (2016) | Component of VRQoL  Embarrassment  Frustration  Annoyance  Loneliness  Isolation  Sadness  Feeling low  Worry (about further vision loss)  Coping (with daily life)  Feeling like a burden/nuisance | IVI – Emotional well-being subscale (Thai version) | *Statistical analysis demonstrated the Thai-version IVI questionnaire is valid and reliable to evaluate the VRQoL of the Thai patients through three subscales: (i) mobility and independence, (ii) reading and accessing information, and (iii) emotional well-being... S1 Table. The specific items of the IVI questionnaire. Domains: Emotional well-being. 21. Have you felt embarrassed because of your eyesight? 22. Have you felt frustrated or annoyed because of your eyesight? 23. Have you felt lonely or isolated because of your eyesight? 24. Have you felt sad or low because of your eyesight? 25. In the past month, how often have you worried about your eyesight getting worse? 26. In the past month, how often has your eyesight made you concerned or worried about coping with everyday life? 27. Have you felt like a nuisance or a burden because of your eyesight? 28. In the past month, how often has your eyesight interfered with your life in general?* | |
| Marakis et al. (2020) | Component of VRQoL  Embarrassment  Frustration  Annoyance  Loneliness  Isolation  Sadness  Feeling low  Worry (about further vision loss)  Coping (with daily life)  Feeling like a burden/nuisance | IVI – Emotional well-being subscale (Greek version) | *These statistical evidence supported the separation of the Greek IVI into three subscales: Mobility and Independence (items 4, 8, and 16– 24), Reading and Accessing Information (items 2, 3, 6, 7, and 9–15), and Emotional Well-being (items 25–32)… Table 1. Items of the Greek IVI and scoring characteristics. 25 Felt embarrassed, 26 Felt frustrated or annoyed, 27 Have you felt lonely or isolated, 28 Have you felt sad or low, 29 Worried about your eyesight getting worse, 30 Concerned or worried about coping with everyday life, 31 Felt like a nuisance or a burden, 32 Interfered with your life in general* | |
| Holz et al. (2011) | Component of VRQoL  Embarrassment  Frustration  Annoyance  Loneliness  Isolation  Sadness  Feeling low  Worry (about further vision loss)  Coping (with daily life)  Eyesight interfering with life  Feeling like a burden/nuisance | IVI – Emotional well-being subscale (German version) | *The IVI is an instrument to measure the impact of vision impairment on VRQoL... Items form three specific subscales: reading and accessing information, mobility and independence, and emotional well-being (Table 1)… TABLE 1. Structure, Item Content, and Scales of the German IVI Questionnaire. Emotional well-being 21. Felt embarrassed? 22. Felt frustrated or annoyed? 23. Felt lonely or isolated? 24. Felt sad or low? 25. Worried about your eyesight getting worse? 26. Concerned or worried about coping with everyday life? 27. Felt like a nuisance or a burden? 28. Interfered with your life in general?* | |
| Rees et al. (2007b) | Component of QoL  Embarrassment  Frustration  Annoyance  Loneliness  Isolation  Sadness  Feeling low  Worry (about further vision loss)  Coping (with daily life)  Eyesight interfering with life  Feeling like a burden/nuisance | IVI – Emotional well-being subscale | *The Impact of Vision Impairment (IVI) questionnaire was designed to assess participation in daily activities and determine the outcome of low-vision rehabilitation on quality of life in people with low vision... Table 3. The Item Labels and Factor Loadings of the Three-Factor Model of the IVI Questionnaire. Emotional well-being items: 12. Felt embarrassed?, 13. Felt frustrated or annoyed?, 14. Have you felt lonely or isolated?, 15. Have you felt sad or low?, 16. Worried about your eyesight getting worse?, 17. Concerned or worried about coping with everyday life?, 18. Interfered with your life in general?, 19. Felt like a nuisance or a burden?* | |
| Glick et al. (2019) | Mental health  Depression | CES-D | *Appendix 1 - Details on the Construction of Selected Indicators of Well-Being. Mental health: Emotional well-being is measured using an adaptation of the 20 question Center for Epidemiologic Studies Depression Scale (CES-D).* | |
| Sturrock et al. (2015) | Component of VRQoL Embarrassment  Frustration  Annoyance  Isolation  Loneliness  Sadness  Worry (about further vision loss)  Coping (with daily life)  Feeling like a burden/nuisance | IVI – Emotional well-being subscale | *Vision-related quality of life (VRQoL) represents the measurement of the impact of vision loss on emotional well-being, social relationships, and independence in daily functional activities… The Vision-Related Emotional Well-Being scale asks participants to indicate how they have been feeling because of their vision impairment over the past month. Items refer to embarrassment, frustration, annoyance, isolation, loneliness, sadness, and worry about change in vision, coping with daily life, and feeling like a burden.* | |
| Nastasi (2014) | Happiness  Sadness | How would you rate your emotional well-being on a scale of one to ten, where ten is very happy and one is very sad? | *Participants described their overall health and quality of life as fair to good, and rated their emotional well-being between eight and nine on a scale of one to ten… APPENDIX C THE OCCUPATIONAL LIVES OF INDIVIDUALS WITH VISUAL IMPAIRMENT DEMOGRAPHIC QUESTIONNAIRE (OLIVIDQ). The Occupational Lives of Individuals with Visual Impairment Demographic Questionnaire (OLIVIDQ). 19. How would you rate your emotional well-being on a scale of one to ten, where ten is very happy and one is very sad?* | |
| Horowitz (2004) | Depression | Not identified | *This article will review recent evidence regarding the prevalence of vision impairment among older adults, as well as the wide-ranging consequences of vision impairment for the physical functioning, emotional well-being, and health and health service needs of older adults in later life… The consequences of a late-life vision loss are especially pronounced for the emotional well-being of older adults, and in particular for the experience of depression.* | |
| Wahl (2013) | Not identified | Not identified | *On the one hand, a well-filled “tool box” of strategies is at hand to help the old individual affected by severe vision impairment to make the best out of her/his situation in terms of autonomy and well-being, because many older adults have optimized the world in which they live over the years as a highly predictable and socially stable one, they are able to counteract threats to their cognitive-emotional well-being to a large extent.* | |
| Khoo et al. (2019) | Component of psychosocial functioning  Component of VRQoL  Not identified | RetCAT | *To determine the relationship between DR/DME and psychosocial functioning, the latter an umbrella term used to capture the emotional and social aspects of functioning which may include, for example, depression; depressive disorder; anxiety; vision-specific distress; diabetes-specific distress and emotional and social well-being… Current evidence for the bi-directional relationship between poor psychosocial outcomes and DR was limited to depression, with no studies reporting other aspects of psychosocial functioning (e.g. anxiety, emotional well-being)… RetCAT [71, 72] is an item bank and computerised adaptive testing (CAT) system comprising domains of DR-specific QoL including emotional well-being, social well-being and concerns.* | |
| Lee et al. (1995) | Not identified | SF-36 | *The SF-36 measures eight distinct concepts: physical functioning, role limitations due to physical problems, social functioning, bodily pain, emotional well-being, role limitations due to emotional problems, energy and fatigue and general health.* | |
| Finger et al. (2011) | Emotions (e.g. embarrassment) | IVI – Emotional well-being subscale | *The IVI questionnaire is a vision-specific instrument which measures the impact of vision impairment on various QoL parameters and was developed using focus group discussions and input from existing instruments... Items form three specific subscales: 'reading and accessing information', 'mobility and independence' and 'emotional well-being'… The Emotional Scale had adequate discriminant ability and satisfied the requirements for unidimensionality. One item (Item 21) displayed misfit (MNSQ 1.64 logits), however, it was retained as deleting it did not improve fit statistics and it captures important emotional information, i.e. embarrassment caused by eyesight.* | |
| Gan et al. (2020a) | Frustration  Anger  Upset  Sadness  Feeling low  Miss doing things you used to do  Enjoyment  Confidence  Worry (about further vision loss, having eye treatment)  Concerned (about things like losing driver’s license)  Social isolation  Role disruption  Ability to work | Diabetic Retinopathy Utility Index (DRU-I) | *Supplementary Table 2. Dimensions and levels of the DRU-I. Dimension 4. Emotional well-being: Because of your diabetic eye disease and vision loss, you may feel frustrated, angry, upset, or sad or low. You may miss doing the things you used to do or enjoy things less, and feel less confident. You may also feel worried about your vision getting worse and having treatment for your eyes (e.g. laser therapy). You may also be concerned about things like losing your driver’s license, becoming socially isolated, not being able to maintain your roles and responsibilities, and not being able to work. The list is not exhaustive and individuals may have other emotional reactions or concerns similar to these. 1. No difficulty means that you have no emotional reactions or concerns relating to your diabetic eye disease or vision loss. 2. Some difficulty means that you sometimes experience emotional reactions and concerns, but overall they do not last very long. 3. A lot of difficulty means that you often experience emotional distress and concerns about your vision, and this may interfere with your ability to function day to day...* | |
| Pesudovs et al. (2008) | Not clear | IVI – Emotional well-being subscale | *There was evidence of multidimensionality, indicating that the overall IVI score should be discarded; however, the 3 subscales (reading and accessing information, mobility and independence, and emotional well-being) functioned well… The largest disparities were in recognizing people (-2.2), getting about outdoors (-1.6), Lonely (-1.6), and sad or low (-2.4), - items easier for cataract patients - and spilling things (1.4), frustrated (2.5), coping (1.2), and interfering with life (1.4) - items more difficult for cataract patients. This suggests that different issues are important to people with cataract. For instance, cataract patients seem less troubled than low-vision patients by depression type of emotional issues such as sadness or loneliness. On the other hand, cataract patients were more troubled by emotional issues such as difficulty coping, experiencing frustration, and vision interfering with life overall* | |
| Misajon et al. (2005) | Component of VRQoL  Upset  Worry (about further vision loss)  Anxiety  Happiness  Confidence to join in activities  Coping | VisQoL  Does my vision affect my confidence to join in everyday activities?  My vision  - makes me more confident to join in everyday activities.  - has no effect on my confidence to join in everyday activities.  - makes me feel a little less confident.  - makes me feel moderately less confident.  - makes me feel a lot less confident.  - makes me not confident at all. | *Across the three focus groups, both intrinsic and mediating factors were revealed to facilitate participation and perceived QoL (Table 1). Intrinsic factors were defined as issues concerning the self, such as independence, social well-being, emotional well-being, physical well-being, and self-actualization… The items were drawn from the broad areas of social, emotional, and physical well-being; independence; self-actualization; and planning and organization… TABLE 2. VisQoL Item Bank: 33 Items across Six Broad Dimensions. V12 Frequency that I get upset about vision V14 Frequency that I worry about vision getting worse V18 Vision causing anxiety about health V22 How happy I am V28 Vision affects my confidence to join in activities V31 Vision makes me fearful about ability to cope with future problems… Appendix. TABLE 8. Final VisQoL Items. Q6 Does my vision affect my confidence to join in everyday activities? My vision e makes me more confident to join in everyday activities. e has no effect on my confidence to join in everyday activities. e makes me feel a little less confident. e makes me feel moderately less confident. e makes me feel a lot less confident. e makes me not confident at all.* | |
| Lange et al. (2021) | Frustration  Embarrassment  Anxiety  Sadness  Anger  Depression  Helplessness  Regrets  Gratitude/Recognition of remaining capabilities  Self-esteem | Qualitative interviews  IVI – Emotional well-being subscale | *participants completed semi-structured qualitative interviews, the Impact of Vision Impairment (IVI) questionnaire and the RAND 36-Item Health Survey…Emotional well-being. Participants described feelings of frustration related to their limited vision… Embarrassment and anxiety were also common. For many, severe PFL meant that they could be easily startled by people or objects outside of their central vision, and this provoked anxiety… Sadness, anger, and even clinical depression, occurred in response to losing independence. Some expressed feelings of helplessness, wondering why this happened to them, or why their disease had progressed faster than others’… My self-esteem went down. I got depressed. I didn't want to accept it. It's embarrassing. It's like you can't have fun anymore. It's like your eyesight is so important. I would rather have my arm or my leg cut off. I just want my sight to see the beauty of the world. (53 year-old male; glaucoma)… Several older participants regretted not fully taken advantage of better vision earlier in life. Notwithstanding, many expressed gratitude for what they are able to do in spite of severe PFL.* | |
| Tan et al. (2019) | Component of VRQoL  Not identified | IVI – Emotional well-being subscale | *The Impact of Vision Impairment (IVI) Questionnaire is a vision-specific QoL instrument developed to measure social participation, function, and emotional well-being in patients with vision impairment (VI)* | |
| Aroney et al. (2016) | Component of VRQoL  Not identified | IVI – Emotional well-being subscale | *Vision-related quality of life was measured at baseline and 24 months, using the IVI's three component scales, namely reading, mobility, and emotional well-being… Previous large clinical trials have reported improved VRQoL in patients following other anti-VEGF treatment (such as ranibizumab) compared with laser or sham in DME.12,13 However, in these studies improvements were not consistent across all NEIVFQ subscales and were predominantly related to visual functioning. This contrasts with our study, where we found significant improvements across all three VRQoL domains, including emotional well-being.* | |
| Rees et al. (2010b) | Vision-specific distress:  Embarrassment  Frustration  Annoyance  Loneliness  Isolation  Sadness  Depression  Worry (about further vision loss)  Coping  Feel like a burden/nuisance  Eyesight interfering with life | IVI – Emotional well-being subscale | *The third subscale comprises eight items that assess emotional well-being. The Emotional Well-being subscale was used as a measure of vision-specific distress. This subscale asks participants to state how they have been feeling because of their eyesight. Items refer to embarrassment, frustration and annoyance, loneliness and isolation, feeling sad or depressed, worrying about eyesight getting worse, worrying about coping with everyday life, concern about being a nuisance or burden, and interference with life in general* | |
| Affective well-being | | | | |
| Study | **Indicator** | **Measure** | **Quote** | |
| Wahl et al. (2019) | Positive affect  Negative affect | PANAS | *Affective well-being. Positive and negative affect were assessed based on the 20- item Positive and Negative Affect Schedule (PANAS)* | |
| Schilling et al. (2016) | Happiness  Depression | 20-item CES-D  Single item rating overall happiness on an 11-point scale, ranging from (0) not happy at all to (10) very happy | *Affective well-being encompasses “a person’s feelings or emotional states, typically measured with reference to a particular point in time” (…), and is commonly defined in terms of the balance of pleasure and displeasure (…). Thus, affective well-being could be considered as the degree of overall pleasantness that results from the positive and negative emotions and moods the person feels within the reporting period. [ 1 ] We analyze two outcomes that represent crucial facets of affective well-being— general sense of happiness and depressive symptoms… The 20-item Center for Epidemiological Studies Depression Scale (CES-D;…) was used to assess depressive symptoms experienced in the past week… A single item was used as an indicator of happiness. Participants were asked to rate their overall happiness on an 11-point scale, ranging from (0) not happy at all to (10) very happy.* | |
| Wahl et al. (2014) | Positive affect (interested, excited, strong, enthusiastic, proud, alert, inspired, determined, attentive, and active)  Negative affect (distressed, upset, guilty, ashamed, hostile, irritable, nervous, jittery, scared, afraid) | Positive and Negative Affect Schedule (PANAS) | *Affective well-being frequently is characterized by two conditions: the presence of positive affect (PA) and the absence of negative affect (NA;…)... In terms of the affective component of well-being, PA was assessed using the PANAS (Positive Affect and Negative Affect Schedule;..), which consists of 10 PA items (interested, excited, strong, enthusiastic, proud, alert, inspired, determined, attentive, and active) and 10 NA items (distressed, upset, guilty, ashamed, hostile, irritable, nervous, jittery, scared, afraid).* | |
| Schilling and Wahl (2006) | Positive affect  Negative affect | PANAS (German version) | *Age-related macular degeneration (AMD) was used as a case model to longitudinally study adaptation in affective well-being under a prevalent chronic health condition. Measures of positive and negative affect, obtained at 5 subsequent measurement occasions with 3-month intervals in between, were analyzed in 90 older adults diagnosed with AMD… Affect. PA and NA were assessed by the German version of the Positive and Negative Affect Schedules* | |
| Wahl et al. (2013) | Positive affect  Negative affect | PANAS | *affective well-being, positive and negative affects were assessed with the Positive and Negative Affect Schedule (…), which consists of 10 positive and 10 negative affect items rated in reference to the month before assessment.* | |
| Wettstein et al. (2015) | Positive affect  Negative affect | PANAS | *The components of affective well-being, positive affect and negative affect, were assessed with the Positive and Negative Affect Schedule (PANAS; …)* | |
| Social well-being | | | | |
| Study | **Indicator** | **Measure** | **Quote** | |
| Assi et al. (2021) | Component of QoL  Not identified | Not identified | *Quality of life, an important measure of physical, emotional, and social well-being. Existing evidence suggests that vision impairment is associated with lower quality of life(2) defined as physical, emotional, and social well-being.* | |
| Salminen et al. (2019) | Not identified | Not identified | *The themes of the second period (two days) were social and psychological well-being, sexuality and self-determination, leisure time, and hobbies.* | |
| Godier-McBard et al. (2020) | Intimate relationship status  Functioning in intimate relationships  Relationship satisfaction  Being in a parental role  Parental functioning  Satisfaction with the relationship with children  Broader social interaction (with friends/family and wider community)  Social functioning  Satisfaction with social involvement | WBI | *Table 2. Proportional results for WBI domain status, functioning, and satisfaction. WBI variables: Social well-being: In an intimate relationship, Functioning well in an intimate relationship, Satisfied with the intimate relationship, Serves in a parenting role, Functioning well as a parent, Satisfied with the relationship with children, Broader social involvement (with friends/family and wider community), Functioning well in the community, Satisfied with the community* | |
| Castle et al. (2021) | Not identified | Not identified | *Art as activity may offer benefits to the wider UK veteran population relating to holistic well-being needs, the promotion of good mental health, and the maintenance of active social and leisure lives, but this has not been explored. This is surprising given the cognitive, social, emotional and physical well-being benefits which have been associated with engagement with art activities in general populations* | |
| Wettstein et al. (2015) | Loneliness  Social isolation | UCLA Loneliness scale | *Finally, higher loneliness, an important indicator of social well-being (…) reflecting the aversive experience of perceived social isolation (…), was found to be associated with lowered cognitive abilities (…) and higher risk of cognitive impairment… Loneliness was assessed with the University of California Loneliness scale* | |
| Ang et al. (2020) | Component of glaucoma-specific QoL  Not identified | GOAT | *Glaucoma-specific QoL (primary outcome) was measured*  *using the Glaucoma Outcomes Assessment Tool (GOAT;*  *342 items, 12 domains). While we found a significant between-group difference in the GOAT social well-being domain at 2 years in ITT analysis, with patients in the SLT arm showing a significant improvement compared with no changes in the medication arm, no other between-group differences were observed for the remaining QOL domains* | |
| Runjic et al. (2018) | Component of QoL  Not identified | Not identified | *Quality of life is a multi-dimensional concept that comprises different aspects of life. The individuals’ quality of life has to be estimated through physical health, material, social and emotional well-being* | |
| Burmedi et al. (2002) | Not clear | Not clear | *The relationship between objective vision factors and loneliness remains rather unclear, particularly the question whether low-vision elderly are lonelier than sighted older adults. Subjective vision seems to be important for the social well-being of visually impaired elderly* | |
| Nyman et al. (2010a) | Social contact  Social activity  Loneliness  Exclusion  Social isolation  Social participation (leaving the house)  Disengagement with the community | Not identified | *Social well-being. The studies reviewed suggested that adults with sight loss are not satisfied with their level of social contact and activity, as people with sight loss have reported feelings of loneliness, exclusion, and social isolation (…). A quarter of one sample did not feel sufficiently in touch with people, and over half did not feel engaged with their community and wanted to go out more often... Studies suggested that compared to their sighted peers, people with sight loss report a lower level of emotional well-being and can report lower levels of social well-being in the form of reduced social activity, loneliness, and disengagement with the community.* | |
| Khoo et al. (2019) | Component of QoL  Not identified | RetCAT | *In this paper, we have used the term “psychosocial” as an umbrella term to capture a wide range of potential psychosocial factors including depression, depressive disorder, anxiety, vision-specific distress, diabetes-specific distress and emotional and social well-being… RetCAT [71, 72] is an item bank and computerised adaptive testing (CAT) system comprising domains of DR-specific QoL including emotional well-being, social well-being and concerns* | |
| Yuzawa et al. (2013) | Component of QoL  Not identified | QoL measures | *In this context, it is notable that the World Health Organization constitution describes health as a state of physical, mental, and social well-being, not merely the absence of disease or infirmity. Consequently, the measurement of health should encompass an assessment of mental and social well-being, which can be accomplished by using QoL measures* | |
| Mirandola et al. (2019) | Component of QoL  Not identified | Not identified | *Quality of life (QoL) is multidimensional and includes factors such as health, physical functioning, life satisfaction, sense of happiness and social well-being amongst others* | |
| Naylor and Labbe (2017) | Not identified | ORS | *The ORS (…) is a four-item ultra-brief measure given to participants to monitor their individual progress from session to session... Traditionally, participants are to mark on a line with one end being 0 and the other being 10, indicating how the participants’ feel that area of their life has been for them over the course of the week (i.e. individual well-being, interpersonal well-being, social well-being, and general well-being).* | |
| Prem Senthil et al. (2017) | Component of QoL  Social interaction  Interpersonal relationships  Social support  Social activities | Qualitative interviews | *Data were collected through semistructured interviews… We identified nine quality of life themes (domains) relevant to both the groups… Table 2: Social well-being (Interacting socially with people, Strain in personal relationships, Getting help and support from your family and friends, Being part of social activities)* | |
| Gleeson et al. (2017) | Social interaction (socialisation) | Frequency of:  -visits with family or friends  -family and agency assistance | *We sought to measure the impact of lessons in the Alexander technique on vision-related emotional and social well-being, as secondary outcomes to a study on improving physical functioning in this population… Socialisation was measured as the frequency with which participants visited family or friends and was scored as a binary variable of weekly or more versus less than weekly interactions. Family and agency assistance were also measured in this manner* | |
| Menon et al. (2020) | Not identified | Not identified | *Objective. Vision loss has a significant impact on physical, mental and social well-being…* | |
| Dubey et al. (2020) | Fulfilling responsibilities  Social interaction  Interaction with the world | Social functional scale (SFS) | *Social functional scale. Six-item SFS was developed based on literature review, clinical expertise, and data from an initial pilot study with 26 glaucoma patients… Individual items on the social function questionnaire were grouped under two broad categories to reflect on the primary aspects of functional performance: 1. Personal wellbeing (personal safety, personal care, leisure activities) and 2. Social wellbeing (fulfill responsibilities; interact with the world, social interaction)* | |
| Marques-Brocksopp (2014) | Community/social network (social participation, interpersonal connectedness)  Altruistic behaviours (wanting to help others)  Connection with natural physical environment | Qualitative interviews | *Social well-being. The experience of mindfulness practices was also linked to an increased sense of social well-being through a sense of interpersonal connectedness… Improved community/social network. This subtheme relates to social participation and a perceived improved connectedness with other people… Encouraging altruistic behaviours. Participants also spoke of feelings of wanting to help others and share what they had learned… Analysis of the study narratives suggested first that the experience of mindfulness did indeed lead to a sense of spiritual well-being (meaning and purpose in life and growth), which was found to contribute to a state of emotional well-being (empowerment and resilience) and also social well-being (a sense of connectedness to others and the natural physical environment), which in turn was found to lead to positive physical outcomes.* | |
| Garcia et al. (2017) | Component of psychological well-being  Interpersonal interactions  Career goals | Interpersonal impact rating  Career impact rating | *Participants appraised the effects of vision loss on their interpersonal interactions and career goals by providing an impact rating (IR) on a 21-point psychometric scale from −10 to +10. Social well-being index was defined as the average of interpersonal IR and career IR… This value, which was termed social well-being index, is a numerical representation of the effects on the social components of an individual's psychological well-being. aSocial well-being index is calculated as the average of an individual’s interpersonal impact rating and career impact rating.* | |
| Fenwick et al. (2012a) | Component of QoL  Family life (family functioning, problem solving, communication, role maintenance, personal relationships)  Work life (ability to maintain work/unemployment, financial strain/loss of income, premature retirement)  Social life (social interaction, participation in social activities, interpersonal relationships, intimate relationships, social isolation) | Various | *IMPACT OF DR ON SOCIAL WELL-BEING. Social life. In four qualitative studies, patients with DR have reported difficulty maintaining social interaction and disintegration of their social lives… Recently Devenney et al. 27 found that practical difficulties associated with patients participating in social activities led them to withdraw from social situations and consequently become more socially isolated.…Patients have also described anxiety over maintaining friendships or acquaintances, or meeting new people because of difficulty recognizing faces,31 or because of becoming more dependent and feeling burdensome.27 Younger participants with DR described visual impairment as a major deterrent to finding potential partners and forming romantic relationships… Family life. Qualitative evidence also suggests that the lifestyle changes associated with DR may often result in increased stress, tension and irritation between family members; and a worsening of family relationships.26,30,31 Individuals with DR have also reported feeling suffocated from excess family attention and fuss over their condition.30 Others have felt a shift in relationship structure as a result of not being able to perform usual family roles and responsibilities due to vision loss from DR…However, in some cases, DR improved family relationships by bringing members together in a network of caring and kindness... Bernbaum et al. found that family functioning as assessed by the 60-item family assessment device was significantly worse in those with vision loss from diabetes than in a normative population, especially in the areas of problem solving, communication, role maintenance and overall functioning level … Evidence from these studies suggests that DR has a detrimental impact on family functioning, personal relationships, role maintenance within the family unit and may be a contributor to marriage dissolution... Work life… Both Woodcock et al. and Devenney et al. have reported that patients’ eye problems have a major impact on their working life and, in some cases, were the main causes of unemployment and loss of income, usually as a result of no longer being able to drive… Overall, the limited available evidence suggests that people with severe DR such as PDR and clinically significant DME may experience difficulty maintaining employment, unwanted premature retirement and financial burden… The socioemotional impact of DR has more recently been explored indirectly as part of vision-related QoL investigations using various QoL outcome measures* | |
| Misajon et al. (2005) | Component of VRQoL  Physical/emotional intimacy  Unequal treatment by family/friends  Ability to make new friends  Social support  Social contact  Concern about treatment by others  Ability to have friendships | Does my vision affect my ability to have friendships? My vision  -makes having friendships easier.  -has no effect on my friendships.  -makes friendships more difficult.  -makes friendships a lot more difficult.  -makes friendships extremely difficult.  -makes me unable to have friendships.  -Not applicable; I have no friendships. | *Across the three focus groups, both intrinsic and mediating factors were revealed to facilitate participation and perceived QoL (Table 1). Intrinsic factors were defined as issues concerning the self, such as independence, social well-being, emotional well-being, physical well-being, and self-actualization… The items were drawn from the broad areas of social, emotional, and physical well-being; independence; self-actualization; and planning and organization… TABLE 2. VisQoL Item Bank: 33 Items across Six Broad Dimensions. V3 Vision affects physical/emotional intimacy V6 Being treated better/worse by family/friends because of vision Social V11 Vision affects ability to have friendships V15 Vision affects ability to make new friends V17 Support from others affected because of vision V19 Difficulty making/avoiding social contact because of vision V23 Concern about how others treat me because of vision loss… Appendix. TABLE 8. Final VisQoL Items. Q3 Does my vision affect my ability to have friendships? My vision e makes having friendships easier. e has no effect on my friendships. e makes friendships more difficult. e makes friendships a lot more difficult. e makes friendships extremely difficult. e makes me unable to have friendships. e Not applicable; I have no friendships.* | |
| Nyman et al. (2012); | Social functioning (social interaction, social relationships, confidence, social contact)  Social isolation (participation in social activity) | Various | *Psychological well-being was reduced with the onset of depression (n=4), low self-worth (n=8), and fear of further vision loss (n=9), and social well-being was reduced by difficulties in social functioning (n = 5) and social isolation (n=5)… Social functioning. Visual impairment brought a number of challenges to social interactions and social relationships…. Some needed help with transport and were no longer confident in groups [57], and the reduction in mobility limited social contact, the ability to be spontaneous or to reciprocate help [46,53,55], and consequently led to the loss of friendships… Social isolation. Some participants were very socially active and were taking classes, going to luncheons, visiting family, serving community boards, attending church, and talking on citizen band radio* | |
| Khadka et al. (2015) | Component of glaucoma-specific QoL  Not identified | Literature review  Focus groups  Qualitative interviews | *On the basis of the comprehensive literature review, input from patient focus groups, semi-structured interviews and consensus between the researchers, ten domains important to patients with glaucoma were identified. The domains were activity limitation, mobility, visual symptoms, ocular surface symptoms, general symptoms, convenience, health concerns, emotional well-being, social well-being and economic issues… Table 6: Domain question format and response options. Social: How much of a problem do you have with…? None to Unable to do because of my vision* | |
| Sweeting et al. (2020) | Not identified | Not identified | *Outcome measures included a range of physical measurements, such as body fat percentage, blood pressure, body mass, waist circumference; physical activity/ fitness measures such as flexibility, daily step count, balance and muscle strength and endurance; and well-being measures including social and emotional well-being and depression.* | |
| Matthews et al. (2017) | Social engagement | Binary variable describing participation in organizations, clubs or societies (e.g. political parties, environmental groups, neighbourhood watch groups, religious groups, charitable associations, educational groups or classes, social clubs and exercise classes or gyms) | *Using longitudinal data, we investigate whether deterioration and improvement in self-reported vision among people aged 50 years and older in England experience subsequent changes in various aspects of economic, psychological and social well-being… Social engagement is measured using a binary variable describing whether or not the respondent belongs to any organizations, clubs or societies, including political parties, environmental groups, neighbourhood watch groups, religious groups, charitable associations, educational groups or classes, social clubs and exercise classes or gyms… The research presented in this paper demonstrates the importance of understanding the impact of changes in self-reported vision on the psychological, social and financial well-being of older people* | |
| Hernandez Trillo and Dickinson (2012) | QoL | Not identified | *QoL is used to define the physical, psychological, functional, social, and economic well-being of an individual: the impact of a disease may reduce health-related quality of life8 or in this specific case, vision-related QoL* | |
| Smedema and McKenzie (2010) | Family and social well-being:  Not identified | Sense of Well-Being Inventory | *The SWBI is a subjective well-being measure developed specifically for people with disabilities… The SWBI consists of five subscales: physical well-being and associated feelings about self, psychological well-being, family and social well-being, financial well-being… It may be that for individuals with visual impairments, in particular, the internet may represent an expansion of communication capabilities such that they are able to more easily connect with loved ones and make additional social connections with like individuals. In addition, mobility limitations which may make it difficult for individuals with visual impairments to participate in face-to-face social interactions, such as going out to social events, are not present in online communication. Participating in online support groups was negatively associated with physical and family and social well-being* | |
| Guerette and Smedema (2011) | Family and social well-being:  Component of subjective well-being  Social interaction (interactions with others) | Sense of Well-Being Inventory | *The SWBI, developed specifically for people with disabilities, asks participants to rate their level of agreement with 36 items (such as “I get frustrated about my disability”) using a 4-point Likert rating scale (from 1 = strongly disagree to 4 = strongly agree). The five subscales of the SWBI are physical well-being and associated feelings about self, psychological well-being, financial well-being, family and social well-being, and medical care… This finding suggests that older individuals, persons with higher levels of employment, and individuals who report greater perceived social support indicate higher levels of economic (for instance, financial) and family and social (such as interactions with others) well-being.* | |
| Ajuwon and Bieber (2014) | QoL | Not identified | *On the one hand, as stated by Bach and Rioux, (25) “Quality of life is the social well-being enjoyed by people, communities and their society.”* | |
| Interpersonal well-being | | | | |
| Study | **Indicator** | **Measure** | **Quote** | |
| Naylor and Labbe (2017) | Not identified | ORS | *The ORS (…) is a four-item ultra-brief measure given to participants to monitor their individual progress from session to session... Traditionally, participants are to mark on a line with one end being 0 and the other being 10, indicating how the participants’ feel that area of their life has been for them over the course of the week (i.e. individual well-being, interpersonal well-being, social well-being, and general well-being).* | |
| Socio-emotional well-being | | | | |
| Study | **Indicator** | **Measure** | **Quote** | |
| Pondorfer et al. (2021) | Socio-emotional distress | Focus groups  Qualitative interviews | *Moreover, in both the interviews and the FGDs, common themes were socio-emotional distress due to the concern of losing independence, worsening vision in the future, and the resulting impact on everyday life. Based on the results of the qualitative analysis we developed a draft questionnaire consisting of 75 items with a 5-step response scale, including the domains of “reading and accessing information,” “orientation and mobility,” “safety,” and “socio-emotional well-being.”* | |
| Fenwick et al. (2012c) | Component of QoL  Not identified | Macular disease quality of life questionnaire (MacDQoL) | *However, the MacDQoL was multidimensional, necessitating the omission of a number of items and splitting it into an activity limitation and mobility and a socioemotional well-being scale* | |
| Gan et al. (2020a) | Frustration  Anger  Sadness  Miss doing the things you used to do  Confidence  Worry (about further vision loss)  Reduction in social life  Role disruption  Ability to maintain responsibilities | Diabetic retinopathy utility index | *Socio-emotional well-being: For example, feeling frustrated, angry, sad; miss doing the things you used to do, loss of confidence; worry about vision getting worse, reduction in social life, not being able to maintain roles and responsibilities?* | |
| Physical well-being | | | | |
| Study | **Indicator** | **Measure** | **Quote** | |
| Assi et al. (2021) | Component of QoL  Not identified | Not identified | *Existing evidence suggests that vision impairment is associated with lower quality of life,2 defined as physical, emotional, and social well-being… Systematic reviews that reported on quality-of-life outcomes, such as health-related, vision-related, or disease-specific quality-of-life questionnaires, or qualitative assessments of physical, emotional, and social well-being and vision function in day-to-day life* | |
| Wittich et al. (2014) | Used synonymously with physiological well-being and physical wellness  Balance confidence  Falls | ABC  TUG | *Physical well-being. Physical wellness was measured by a combination of the Activity-Specific Balance Confidence (ABC) Scale (…) and the Timed Up and Go test (TUG). The ABC scale measures balance confidence, an important indicator of functional mobility and independence in older adults. The TUG is a sensitive and specific measure for identifying elderly individuals prone to falls… We speculate that the lack of change in our subset partially reflects the accomplishment of the Day Centre to support the physiological and psychological well-being of the participants* | |
| Castle et al. (2021) | Not identified | Not identified | *This is surprising given the cognitive, social, emotional and physical well-being benefits which have been associated with engagement with art activities in general populations* | |
| Heine and Browning (2002) | Not identified | Not identified | *Older adults with sensory loss are frequently sensitive to their disability and have numerous psychosocial behaviours that become evident as a result of their diagnosis. They are often aware of the consequences of their sensory loss and acknowledge that their sensory loss impacts on their physical and mental well-being, often resulting in decreased social performance.* | |
| Hackney et al. (2013) | Balance  Walking  Mood  Coordination  Strength  Endurance  Physical activity | Exit questionnaire | *Participants reported enjoyment and improvements in physical well-being… The questionnaire contains nine items asking if the participant enjoyed the classes, would continue, and noted improvements in aspects of physical well-being… TABLE 2. Exit Questionnaire Item and Open-Ended Responses. Aspects of well-being: Enjoyment, Balance, Walking, Mood, Coordination, Strength, Endurance, Continuing, More physically active. Note. Participant responses to items regarding aspects of physical well-being on a 5-point Likert scale with medians and first and third interquartiles: 1 = strong agreement and 5 = strong disagreement, with items stating that the participant noted improvement in various aspects of physical well-being as a result of participation in adapted tango.* | |
| Rooney et al. (2018) | Not identified | Not identified | *The quality of the home environment influences an occupant’s mental and physical wellbeing, although not always positively* | |
| Bambara et al. (2009) | Not identified | Not identified | *A longitudinal study by Strawbridge, Wallhagen, and Shema (2007) found that the spouses of individuals with vision loss had an increased risk of poorer physical and emotional well-being over five years and that the characteristics of one had an impact on the other.* | |
| Kelly et al. (2021) | Component of VR and HRQoL  Not clear | SF-36v2  NEI-VFQ  IND-VFQ | *In addition to the NEI-VFQ and SF-36v2, participants enrolled in India also completed the IND-VFQ.… The SF-36v2 evaluates HRQoL by assessing a patient’s self-perception of their physical and mental health... The questionnaire can be divided into 2 components, physical and mental health, and 8 domains: physical functioning, role limitations caused by physical health, bodily pain, general health perceptions, vitality (energy and fatigue), general mental health (psychological distress), role limitations because of emotional problems, and social functioning limitations because of emotional problems...* *National Eye Institute Visual Function Questionnaire. The 25-item NEI-VFQ is a validated VRQoL questionnaire that measures functioning and well-being in physical, mental, and social aspects of a patient’s life and is designed specifically for use in vision studies… On all 4 QoL scales, participants who did not achieve corticosteroid-sparing control of ocular inflammation showed substantially lower VRQoL and HRQoL, indicating that inflammation likely impaired visual functioning and, in turn, physical and mental well-being* | |
| Menon et al. (2020) | Not identified | Case notes | *Vision loss has a significant impact on physical, mental and social well-being… ECLOs are a core patient service within ophthalmology and have a significant impact on patients’ emotional and physical well-being and ultimately their quality of life.* | |
| McCormack (2021) | Not identified | Qualitative interviews | *Those individuals with visual impairments who had extensive training in independence skills prior to Hurricane Irma explained that they utilized those skills throughout the hurricane season, and it was an aid to their physical and mental wellbeing* | |
| Brown et al. (2009) | Not identified | Not identified | *Vision impairment associated with diabetic retinopathy (DR) impacts on an individual’s functioning, psychosocial, physical and financial wellbeing.* | |
| Marques-Brocksopp (2014) | Physical functioning  Mobility | Qualitative interviews | *Physical well-being. The fourth theme concerned the relationship between mindfulness and the participants’ perceived physical well-being, which was divided into two subthemes: (1) increased physical functioning and (2) improved mobility.* | |
| Mirandola et al. (2019) | Component of HRQoL  Functioning  Health status | SF-12 (physical component, Italian version) | *The SF-12 questionnaire results demonstrated a significantly higher physical score in visually impaired players compared with the reference population On these premises, to determine whether playing baseball can improve the physical and mental well-being of people with visual impairment, here we performed a national survey based on a structured online questionnaire assessing the PWB and QoL of visually impaired baseball players from Italian teams in comparison with visually impaired sedentary individuals… … The Italian version of the SF-12 questionnaire consists of 2 components: a physical component score and a mental component score. Higher scores on these subscales indicate greater levels of functioning and a more favorable health status, thus being indicative of a better QoL* | |
| Dev et al. (2014) | Not identified | Not identified | *Visual impairment (VI) affects physical, psychological, and emotional well-being, and social life as well… Visual function is important for an optimal orientation in functional and social life and has effects on physical, psychological, mental and emotional wellbeing* | |
| Kahaly et al. (2002) | Component of QoL  Not identified | Not identified | *The third approach mainly examines the comparability of the quality of life among various individuals. In addition to physical and mental well-being, mainly the ability to function in the workplace and in the private realm is surveyed.* | |
| Adigun et al. (2014) | Not identified | Not identified | *Visual function is important for optimal orientation in functional and social life, and has an effect on physical and emotional well-being… Visual function is important for optimal orientation in functional and social life, and has an effect on physical and emotional well-being* | |
| Dagnelie and Kiser (2008) | Not identified | Developed questionnaire on complementary and alternative medicine (CAM) | *RP patients are using CAM and are experiencing some impact on vision and physical/emotional well-being… One item inquired about the motivations or reasons for using each of the CAM areas and the results are depicted in Figure 2. The respondents were able to select multiple answers and could choose from ‘physical well-being’, ‘emotional well-being’, ‘might help, can’t hurt’, ‘desire to fight RP’ and ‘to help with a disease other than RP’. Across each of the CAM areas, over one-half of respondents using CAM did so for physical and emotional well-being, while 63 per cent who tried acupuncture felt that it ‘might help, can’t hurt’… There is ample evidence linking CAM use with reduction of negative mood states, stress, anxiety, depression and fatigue for other types of chronic, disabling diseases. Our findings indicate that only a minimal proportion of patients did not feel that these areas were impacted by CAM use. This suggests that CAM met the patients’ expectations in terms of the potential to improve physical and emotional well-being* | |
| Marques-Brocksopp (2012) | Not identified | Not identified | *A link has been established between positive mental health and physical wellbeing, however this raises the question of whether wellbeing can be ‘measured’?... Although within the realm of visual impairment we have seen an increased interest in the link between emotional and physical wellbeing, the majority of wellbeing measures have tended to focus on the physical and social elements of general quality of life. However, as underlined in the King’s College seminar and elsewhere in the literature (…), wellbeing covers not only the physical and social, but also the emotional and spiritual needs and capabilities of the individual.* | |
| Zimdars et al. (2012) | Physical function:  Mobility  Falls  ADLs  IADLs  Health status  Hearing | Binary ELSA measures | *We use the following binary ELSA measures to capture physical functions – mobility difficulties, falls, activities of daily living, instrumental activities of daily living, self-reported health and hearing... Using ELSA, we showed that self-reported vision is associated with multiple disadvantages, in particular with regards to other physical health functions, but also with regards to cognitive functions, economic well-being, social relationships and social participation, and emotional well-being... From our findings, it seems that self-reported poor vision is a proxy for a lower propensity of an individual to be able to enjoy various good fortunes ranging from emotional and physical well-being to economic circumstances and networks.* | |
| Heine and Browning (2004) | Not clear | Qualitative interviews | *The aims were to identify the communication difficulties and conversational strategies used by the subjects, and to explore their perceptions of their social adjustment, quality of life and physical and mental well-being… The problems of adjusting to sensory loss, depression, anxiety, lethargy and social dissatisfaction were cited as factors that affected their physical and mental well-being, while being optimistic, coping with their sensory loss, and maintaining social contact contributed to an improved quality of life… Both the reaction to sensory losses and the communication limitations they impose tend to damage physical and mental well-being and psychosocial functioning, and these in turn tend to reduce social interaction and may bring about social isolation* | |
| Hernandez Trillo and Dickinson (2012) | Component of QoL  Not identified | 25-item Low vision QoL (LVQOL)  12-item AVL  KAP | *QoL is used to define the physical, psychological, functional, social, and economic well-being of an individual: the impact of a disease may reduce health-related quality of life8 or in this specific case, vision-related QoL… Three questionnaires were treated as outcome measures representing the different aspects of QoL identified above: the Low Vision Quality of Life (the 25-item LVQOL), the Adaptation to Age-Related Vision Loss (the 12-item AVL), and the Keele Participation Restriction Questionnaire (KAP)* | |
| Orr (1991) | Comorbid physical disability  Comorbid health condition | Not identified | *Some of the most significant factors which have an impact on the older visually impaired person’s ability to cope may include: 1. Physical well being… Where no other physical disability or health problem is present, it may be easier to focus on adjusting to vision loss* | |
| Guerette and Smedema (2011) | Component of subjective well-being  Stamina | Sense of Well-Being Inventory (SWBI) | *The SWBI, developed specifically for people with disabilities, asks participants to rate their level of agreement with 36 items (such as “I get frustrated about my disability”) using a 4-point Likert rating scale (from 1 = strongly disagree to 4 = strongly agree). The five subscales of the SWBI are physical well-being and associated feelings about self, psychological well-being, financial well-being, family and social well-being, and medical care… This result suggests that individuals who have higher levels of employment, as well as higher levels of perceived social support, report higher levels of physical well-being (such as stamina).* | |
| Misajon et al. (2005) | Component of VRQoL  Coping with health problems  Injury risk  Awareness of surroundings  Ability to participate in activities | Does my vision make it likely I will injure myself (i.e. when moving around the house, yard, neighborhood, or workplace)?  -It is most unlikely I will injure myself because of my vision. -There is a small chance. e There is a good chance.  -It is very likely.  -Almost certainly my vision will cause me to injure myself | *Intrinsic factors were defined as issues concerning the self, such as independence, social well-being, emotional well-being, physical well-being, and self-actualization… The items were drawn from the broad areas of social, emotional, and physical well-being; independence; self-actualization; and planning and organization… TABLE 2. VisQoL Item Bank: 33 Items across Six Broad Dimensions. V1 Difficulty coping with any health problems because of vision V5 Increased chance of injury because of vision V13 Vision affects my awareness of what is happening around me V27 Vision restricts me from doing things I would like to do… Appendix. TABLE 8. Final VisQoL Items. Q1 Does my vision make it likely I will injure myself (i.e. when moving around the house, yard, neighborhood, or workplace)? e It is most unlikely I will injure myself because of my vision. e There is a small chance. e There is a good chance. e It is very likely. e Almost certainly my vision will cause me to injure myself* | |
| Physiological well-being | | | | |
| Study | **Indicator** | **Measure** | **Quote** | |
| Smith and Anstey (2003) | Biomarkers:  Vision  Hearing  Vibration sense  Grip strength  Lung function | Tumble E Test  Pure tone audiometer  Vibration sense  Grip strength (Spedly manual dynameter)  Forced expiratory volume | *Structural equation modelling showed that biomarkers were most important in predicting self-reported driving behaviour… The battery of sensorimotor and physiological variables was chosen to provide a reliable set of biomarkers as indices of functional age [14]. Corrected visual acuity (Vision) was measured using the Tumble E Test... Hearing was tested with a portable pure tone audiometer (Micro Audiometrics Corp.) using standard audiometric techniques... Grip strength (Grip) was measured in a seated position using a Spedly manual dynameter... Vibration sense (Vibsense) was measured on the tip of the right index finger with an apparatus consisting of an electronic device that drove a 12-cm loudspeaker, generating a 153-Hz vibration [18]... Forced expiratory volume at 1 s (FEV1 ) was measured by a vitalograph… These results suggest that individuals self-monitor according to their physiological well-being and adapt their driving behaviour accordingly.* | |
| Wittich et al. (2014) | Used synonymously with physical well-being and wellness  Balance confidence  Falls | ABC  TUG | *Physical well-being. Physical wellness was measured by a combination of the Activity-Specific Balance Confidence (ABC) Scale (Powell & Myers, 1995) and the Timed Up and Go test (TUG). The ABC scale measures balance confidence, an important indicator of functional mobility and independence in older adults. The TUG is a sensitive and specific measure for identifying elderly individuals prone to falls… We speculate that the lack of change in our subset partially reflects the accomplishment of the Day Centre to support the physiological and psychological well-being of the participants* | |
| Psychophysical well-being | | | | |
| Study | **Indicator** | **Measure** | **Quote** | |
| Mirandola et al. (2019) | QoL  Physical well-being (functioning, health status)  Psychological well-being (autonomy, environmental mastery, personal growth, positive relations with others, purpose in life, and self-acceptance) | SF-12 (Italian version)  18-item PWB (Italian version) | *On these premises, to determine whether playing baseball can improve the physical and mental well-being of people with visual impairment, here we performed a national survey based on a structured online questionnaire assessing the PWB and QoL of visually impaired baseball players from Italian teams in comparison with visually impaired sedentary individuals… In addition, we also compared PWB and QoL data from visually impaired baseball players with normative data from the Italian normally sighted population with the intent to further substantiate the role of baseball practice in mitigating the visual disability-related impact on psychophysical well-being… The Italian version of the SF-12 questionnaire consists of 2 components: a physical component score and a mental component score.. In particular, the PWB-18 scale, based on Ryff’s multidimensional model of PWB, evaluates the personal perception of well-being relative to 6 dimensions: autonomy, environmental mastery, personal growth, positive relations with others, purpose in life, and self-acceptance* | |
| Spiritual well-being | | | | |
| Study | **Indicator** | **Measure** | **Quote** | |
| Marques-Brocksopp (2014) | Intrapersonal connectedness (internal strength, personal growth, present/future outlook)  Interpersonal and transpersonal connectedness (connection with others, other species, and natural environment, relatedness to the unknown) | Qualitative interviews | *Spiritual well-being…Intrapersonal connectedness. Intrapersonal connectedness was identified in relation to the sense of internal strength that participants’ experienced during and following meditation:… Participants also spoke about how mindfulness provided a sense of development and growth through helping participants to re-evaluate their lives and seek meaning and a sense of moving forward:… Another participant spoke of thinking in the ‘present’ and in the process changing the way he thought about his future:… This subtheme was defined in the analysis as ‘inter- and transpersonal connectedness’ and related to a sense of being connected to an external source of energy. This energy was conceived as being interpersonal through connecting with known others, including other species and the natural physical environment. It was also defined by some participants as being transpersonal through a sense of relatedness to the unknown or a power greater than the self, or a combination of the two:…* | |
| Marques-Brocksopp (2012) | Not identified | Not identified | *A rise in concepts such as ‘mindfulness’ (…) and ‘human givens’ (…) echoes this underlying need to account for not just these two overarching dimensions of wellbeing, but also specific areas of emotional and indeed spiritual wellbeing that have to date remained under-researched, especially in the area of visual impairment… However, as underlined in the King’s College seminar and elsewhere in the literature […], wellbeing covers not only the physical and social, but also the emotional and spiritual needs and capabilities of the individual.* | |
| Yampolsky et al. (2008) | Intrapersonal meaning  Intrapersonal wellness  Relationship to a higher power | Spiritual Well-Being Scale (SWBS) | *There is a lack of consensus on the precise definition of spirituality, since it is a subjective experience that is intertwined with other aspects of existence; furthermore, spiritual well-being contains many subdivisions. However, the definitions are generally consistent in that they include intrapersonal meaning and wellness as well as a relationship to a higher power.* | |
| Existential well-being | | | | |
| Study | **Indicator** | **Measure** | **Quote** | |
| Yampolsky et al. (2008) | Intrapersonal wellness:  Meaning of life  Fulfillment  Purpose | SWBS | *Spirituality was tested using the SWBS, which was developed by Paloutzian and Ellison (1982). […] It measures two dimensions of spirituality: the individual’s relationship to a higher power (religious well-being) and the individual’s experience of intrapersonal wellness, which includes a sense of meaning, fulfillment, and purpose (existential well-being).* | |
| Religious well-being | | | | |
| Study | **Indicator** | **Measure** | **Quote** | |
| Yampolsky et al. (2008) | Relationship with higher power  Connection/closeness with higher power | SWBS | *In addition, spirituality involves one’s relationship with and connection or closeness to a higher power (religious well-being)… Spirituality was tested using the SWBS, which was developed by Paloutzian and Ellison (1982)… It measures two dimensions of spirituality: the individual’s relationship to a higher power (religious well-being) and the individual’s experience of intrapersonal wellness, which includes a sense of meaning, fulfillment, and purpose (existential well-being).* | |
| Economic well-being | | | | |
| Study | **Indicator** | **Measure** | **Quote** | |
| Zimdars et al. (2012) | Income  Wealth  Housing tenure  Housing conditions  Labour market involvement | ELSA measures | *For economic circumstances we use measures of income and wealth as well as housing tenure, housing conditions and labour market involvement… Economic well-being. The ELSA data also show significant differences in economic well-being by self-reported level of vision. Table 3 shows that reporting poorer than good vision is furthermore associated with a higher risk of renting rather than home ownership, higher chances of experiencing bad housing conditions, lower labour market participation and increased employment in routine and semi-routine jobs.* | |
| Matthews et al. (2017) | Used synonymously with financial well-being  Equivalised income | Equivalised weekly income | *Using longitudinal data, we investigate whether deterioration and improvement in self-reported vision among people aged 50 years and older in England experience subsequent changes in various aspects of economic, psychological and social well-being… Finally, equivalized weekly income is treated as a continuous variable and is comprised of an individual's total income from employment, pensions, benefits, assets and other sources, adjusted to account for household size… The research presented in this paper demonstrates the importance of understanding the impact of changes in self-reported vision on the psychological, social and financial well-being of older people* | |
| Hernandez Trillo and Dickinson (2012) | Component of QoL  Not identified | Not identified | *QoL is used to define the physical, psychological, functional, social, and economic well-being of an individual* | |
| Smedema and McKenzie (2010) | Used synonymously with financial well-being  Not identified | SWBI | *The SWBI consists of five subscales: physical well-being and associated feelings about self, psychological well-being, family and social well-being, financial well-being, and medical care… In the present analyses, the uncorrected p’s in order of significance are 0.077 (physical well-being), 0.085 (psychological well-being), 0.234 (medical well-being), and 0.409 (family and social well-being), and 0.801 (economic well-being)… None of the Internet activities appeared to be associated with economic well-being* | |
| La Grow et al. (2015) | Capacity for economic consumption  Economic-related social activity  Asset ownership | Economic Living Standard Index-Short Form (ELSISF) | *Economic well-being. Participants completed the Economic Living Standard Index-Short Form (ELSISF; …), which was developed by the New Zealand government as an indicator of well-being related to capacity for economic consumption, economic-related social activity, and asset ownership, rather than providing a simple assessment of the economic resources that enable them (income).* | |
| Guerette and Smedema (2011) | Used synonymously with financial well-being  Not identified | SWBI | *The five subscales of the SWBI are physical well-being and associated feelings about self, psychological well-being, financial well-being, family and social well-being, and medical care… This finding suggests that older individuals, persons with higher levels of employment, and individuals who report greater perceived social support indicate higher levels of economic (for instance, financial) and family and social (such as interactions with others) well-being.* | |
| Financial well-being | | | | |
| Study | **Indicator** | **Measure** | **Quote** | |
| Godier-McBard et al. (2020) | Financially security  Financial functioning  Satisfaction with financial circumstances | WBI | *Table 2. Proportional results for WBI domain status, functioning, and satisfaction. WBI variables: Financial well-being: Financially secure, Functioning well financially, Satisfied with financial circumstances.* | |
| Brown et al. (2009) | Not identified | Not identified | *Vision impairment associated with diabetic retinopathy (DR) impacts on an individual’s functioning, psychosocial, physical and financial wellbeing.* | |
| Matthews et al. (2017) | Used synonymously with economic well-being  Equivalised income | Equivalised weekly income | *Using longitudinal data, we investigate whether deterioration and improvement in self-reported vision among people aged 50 years and older in England experience subsequent changes in various aspects of economic, psychological and social well-being… The research presented in this paper demonstrates the importance of understanding the impact of changes in self-reported vision on the psychological, social and financial well-being of older people… Increasing the uptake of free eye tests among older people potentially provides an effective means of protecting against vision decline42 and the subsequent decline in mental, social and financial well-being associated with worsening sight* | |
| Smedema and McKenzie (2010) | Used synonymously with economic well-being  Not identified | SWBI | *The SWBI consists of five subscales: physical well-being and associated feelings about self, psychological well-being, family and social well-being, financial well-being, and medical care… In the present analyses, the uncorrected p’s in order of significance are 0.077 (physical well-being), 0.085 (psychological well-being), 0.234 (medical well-being), and 0.409 (family and social well-being), and 0.801 (economic well-being)* | |
| Guerette and Smedema (2011) | Used synonymously with economic well-being  Not identified | SWBI | *The five subscales of the SWBI are physical well-being and associated feelings about self, psychological well-being, financial well-being, family and social well-being, and medical care… This finding suggests that older individuals, persons with higher levels of employment, and individuals who report greater perceived social support indicate higher levels of economic (for instance, financial) and family and social (such as interactions with others) well-being.* | |
| Vocational well-being | | | | |
| Article | **Indicator** | **Measure** | **Quote** | |
| Godier-McBard et al. (2020) | Employment status  Education status  Unpaid work  Work functioning  Work satisfaction | WBI | *Table 2. Proportional results for WBI domain status, functioning, and satisfaction. WBI variables: Vocational well-being: Currently employed (full and part-time), Carrying out unpaid work (e.g., volunteering and care work), Functioning well in paid or unpaid work, Satisfied with paid or unpaid work, Pursuing part-time education.* | |
| Environmental well-being | | | | |
| Study | **Indicator** | **Measure** | **Quote** | |
| Marques-Brocksopp (2014) | Not identified | Not identified | *Similar evidence for a sense of ‘socio-ecological’ well-being can be found in Fisher’s (2011) creation of the ‘environmental domain’ of well-being or the importance of handling the environment in Ryff’s (1989) core eudemonic dimension of ‘environmental mastery’.* | |
| Siira et al. (2020) | Not clear | Not clear | *The environmental well-being of elderly home-dwelling people with VI is analyzed using health-related quality of life (HRQoL), which is understood as a multidimensional concept (…) that encompasses physical, emotional, and social factors associated with disability… The aim of this study is to describe the well-being supportive home environment of elderly people with VI and its relationship with HRQoL… The structured well-being supportive environment instrument (…) and the generic 15D health-related quality of life (HRQoL) instrument (…) were included in the interviews… The well-being supportive environment instrument used in this study is based on an instrument development and theory testing process introduced by Elo (2006) to assess environments that support the well-being of elderly people in northern regions… The physical environment is evaluated in terms of safety at home, the pleasance of the physical environment, and the safety of the living environment. The social environment is analyzed in terms of the pleasance of the social environment, interpersonal relationships, and ability to get help. The symbolic environment is analyzed in terms of the respondent’s fears, the natural environment (nature), the respondent’s mood, and feelings of restrictiveness… The instruments used in this study are valid and reliable for assessing environmental well-being and the HRQoL of elderly people in northern environments.* | |
| Socio-ecological well-being | | | | |
| Study | **Indicator** | **Measure** | **Quote** | |
| Marques-Brocksopp (2014) | Environmental well-being  Environmental mastery | Not identified | *Similar evidence for a sense of ‘socio-ecological’ well-being can be found in Fisher’s (2011) creation of the ‘environmental domain’ of well-being or the importance of handling the environment in Ryff’s (1989) core eudemonic dimension of ‘environmental mastery’.* | |
| Eudaimonic well-being | | | | |
| Study | **Indicator** | **Measure** | **Quote** | |
| Marques-Brocksopp (2014) | Meaning  Reflexivity | Not identified | *Furthermore, links may be drawn between such concepts of spirituality and that of the dimension of eudemonic well-being… Mindfulness may therefore encourage long-term changes in an individual’s sense of eudemonic well-being by encouraging the conditions within which individuals are able to find meaning and act in a reflexive way* | |
| Marques-Brocksopp (2012) | Health outcomes  Coping | Not identified | *It is suggested, therefore, that the eudemonic dimension of wellbeing warrants further attention, especially within visual impairment research. As noted by Vázquez et al. (2010), this second dimension of wellbeing is particularly related to long-term health outcomes and to the ability to cope flexibly and creatively with life’s challenges* | |
| McLean et al. (2016) | Component of QoL  Living one’s life well:  Relationships  Standing out/being different  Feelings about inner self  Daily functioning  Restriction of movement  Future | Nystagmus-specific quality-of-life questionnaire (NYS-29) | *We have developed a 29-item, nystagmus-specific QOL questionnaire (NYS-29) based on eudaimonic aspects of well-being with subscales that address not only physical functioning but also psychosocial issues… In this study, we aim to develop a questionnaire to assess QOL among patients with nystagmus with an emphasis on measuring those eudaimonic well-being themes previously identified of relationships, standing out/being different, feelings about the inner self, daily functioning, restriction of movement, and the futureThe items and scales within this study have been developed in the context of eudaimonic well-being, emphasizing the measurement of well-being in terms of overall engagement with living one’s life well* | |
| Capability well-being | | | | |
| Study | **Indicator** | **Measure** | **Quote** | |
| Breheny et al. (2020) | Attachment (love, friendship)  Security (thinking about the future without concern)  Role (doing things that make you feel valued)  Enjoyment  Control (independence) | ICECAP-O  ICECAP-A | *Developments in preference-based measures (PBM) also includes the EQ-5D-5L and the ICECAP-O capability wellbeing measure… The ICECAP-O’s five domains cover attachment (love and friendship), security (thinking about the future without concern), role (doing things that make you feel valued), enjoyment and control (independence)… Future work could explore the suitability and performance of the ICECAP-A [44] in cataract patients given the potential importance of capability wellbeing in this context. The ICECAP-A measures capability wellbeing in adults as opposed to the focus on older adults in the ICECAP-O.* | |
| Functional well-being | | | | |
| Study | **Indicator** | **Measure** | **Quote** | |
| McGwin and Owsley (2007) | Component of QoL  Not identified | SF-36 | *Although generic quality of life questionnaires like the SF-36 have the advantage of facilitating a comparison of personal burden across diseases, at times they can be insensitive to the consequences of visual impairment for health status and functional well-being.* | |
| Dubey et al. (2020) | Personal wellbeing (personal safety, personal care, leisure activities)  Social wellbeing (fulfilling responsibilities, interacting with the world, social interaction) | 6-item Social functional scale (SFS) | *Social functional scale Six-item SFS was developed based on literature review, clinical expertise, and data from an initial pilot study with 26 glaucoma patients… Individual items on the social function questionnaire were grouped under two broad categories to reflect on the primary aspects of functional performance: 1. Personal wellbeing (personal safety, personal care, leisure activities) and 2. Social wellbeing (fulfill responsibilities; interact with the world, social interaction). We investigated the impact of glaucoma on quality of life and functional wellbeing of Indians.* | |
| Hernandez Trillo and Dickinson (2012) | Component of QoL  Not identified | Not identified | *QoL is used to define the physical, psychological, functional, social, and economic well-being of an individual* | |
| Health-related well-being | | | | |
| Study | **Indicator** | **Measure** | **Quote** | |
| Godier-McBard et al. (2020) | Chronic physical health conditions  Chronic mental health conditions  Health functioning  Health satisfaction | WBI | *Table 2. Proportional results for WBI domain status, functioning, and satisfaction. WBI variables: Health-related well-being: Chronic mental health condition, Additional chronic physical health condition (other than VI), Higher health functioning, Satisfied with health.* | |
| Medical well-being | | | | |
| Study | **Indicator** | **Measure** | **Quote** | |
| Smedema and McKenzie (2010) | Medical care | SWBI | *The SWBI consists of five subscales: physical well-being and associated feelings about self, psychological well-being, family and social well-being, financial well-being, and medical care… In the present analyses, the uncorrected p’s in order of significance are 0.077 (physical well-being), 0.085 (psychological well-being), 0.234 (medical well-being),…* | |
| Guerette and Smedema (2011) | Medical care | SWBI | *Subjective well-being was measured via the outcome variables from Cimarolli and Boerner’s study (depressive symptoms and satisfaction with life), along with five additional sense of wellbeing factors: physical well-being, psychological well-being, financial well-being, family or social well-being, and medical well-being… The five subscales of the SWBI are physical well-being and associated feelings about self, psychological well-being, financial well-being, family and social well-being, and medical care* | |
| Clinical well-being | | | | |
| Study | **Indicator** | **Measure** | **Quote** | |
| Friedman et al. (2003) | Not identified | Not identified | *In the present report, erythropoietin was administered to five anemic azotemic diabetic subjects for one year to assess the effect of increasing red cell mass on clinical well-being and the course of renal functional decline* | |
| Cognitive well-being | | | | |
| Study | **Indicator** | **Measure** | **Quote** | |
| Castle et al. (2021) | Not identified | Not identifed | *This is surprising given the cognitive, social, emotional and physical well-being benefits which have been associated with engagement with art activities in general populations* | |
| Wettstein et al. (2015) | Life satisfaction | SWLS | *Cognitive well-being was assessed with the Satisfaction with Life Scale (SWLS;* | |
| Wahl et al. (2013) | Life satisfaction  Environmental mastery | SWLS (German version)  Environmental Mastery scale | *Cognitive well-being was assessed with the 5-item Satisfaction with Life scale (…; German version …). The Environmental Mastery scale (…) assesses perceived mastery of one’s environment and day-to-day life.* | |
| Wahl (2013) | Component of subjective well-being  Life satisfaction | Not identified | *Subjective well-being (SWB) is frequently defined via its cognitive component as degree of satisfaction with one’s current life… This suggests that although vision impairment is a pronounced psychological challenge in late life, many visually impaired older adults seem to adapt rather well at the level of cognitive well-being, thus also supporting what has been titled the paradox of well-being in later life* | |
| Vision-specific well-being | | | | |
| Study | **Indicator** | **Measure** | **Quote** | |
| Kutzbach et al. (2009) | Component of VRQoL  Not identified | 39-item NEI-VFQ | *The NEI-VFQ-39 is a method to evaluate self-reported effects of vision-related QOL in albinism and may be used as a baseline for evaluating outcomes in interventional studies in these patients… The NEI-VFQ-25 can be given alone or with the inclusion of 14 additional questions about vision-specific well-being (when all questions are answered and scored, it is referred to as the NEI-VFQ-39).* | |
| Visual well-being | | | | |
| Study | **Indicator** | **Measure** | **Quote** | |
| Nguyen et al. (2019) | Not identified | 39-item NEI-VFQ | *All patients signed an informed consent form approved by the institutional review board of St. Joseph Hospital, Orange, California, and completed the 39-item National Eye Institute Visual Function Questionnaire (NEI-VFQ-39) to assess visual well-being…The NEI-VFQ-39 is useful in quantifying the impact on general visual well-being, but floater-specific questionnaires may be more telling.* | |

Key: 12-item Medical Outcomes Study – Short Form (SF-12); 36-item Medical Outcomes Study – Short Form (SF-36); 12-item Well-Being Questionnaire (W-BQ12); 22-item Well-Being Questionnaire (W-BQ22); 19-item Control, Autonomy, Self-realization and Pleasure scale (CASP-19); 20-item WHO/Prevention of Blindness and Deafness Visual Functioning questionnaire (WHO/PBD-VF20); 7-item General Anxiety Disorder form (GAD-7); 9-item Patient Health Questionnaire (PHQ-9); Activity-Specific Balance Confidence (ABC) Scale; Adaptation to Vision Loss scale (AVL); Affect Balance Scale (ABS); Center for Epidemiologic Studies Depression Scale (CES-D); Clinical Outcomes Routine Evaluation – Outcome Measure (CORE-OM); Depression, anxiety and stress scale (DASS); Diagnostic and statistical manual 4th edition (DSM-IV); EuroQoL (EQ-5D); Functional Low-vision Observer Rated Assessment (FLORA); General Health Questionnaire (GHQ); 12-item General Health Questionnaire (GHQ-12); Geriatric Depression Scale (GDS); Glaucoma Outcomes Assessment Tool (GOAT); Hamilton Depression Rating Scale (HDRS); Hospital Anxiety and Depression Scale (HADS); ICEpop CAPability measure for Adults (ICECAP-A); ICEpop CAPability measure for Older people (ICECAP-O); Impact of Vision Impairment questionnaire (IVI); Impact of Vision Impairment for Residential Care (IVI-RC); Impact of Vision Impairment-Very Low Vision (IVI-VLV); Indian Visual Function Questionnaire (IND-VFQ); Life Satisfaction in the Elderly Scale (LSES); Life Satisfaction Index – A (LSI-A); Life Satisfaction Index – Well-being (LSI-W); Life Space Questionnaire (LSQ); Mood Adjective Check List (MACL); Multiple Affect Adjective Check List-Revised Sixth Grade Reading Level (MAACL-R6); National Eye Institute Visual Function Questionnaire (NEI-VFQ); Nottingham Adjustment Scale (NAS); Orientation and mobility outcomes (OMO); Outcome Rating Scale (ORS); Philadelphia Geriatric Center Morale Scale (PGCM); Positive and Negative Affect Schedule (PANAS); Profile of Mood States (POMS); Psychological and general well-being Index (PGWB); Psychological Well-Being Scale (PWB); Satisfaction with Life Scale (SWLS); Sense of Well-Being Inventory (SWBI); Short Warwick-Edinburgh Mental Wellbeing Scale (SWEMWBS); Spiritual Well-Being Scale (SWBS); Symptom Checklist-90-Revised (SCL-90-R); Timed Up and Go Test (TUG); Vision-related outcomes in orientation and mobility (VROOM); Visual Function Questionnaire-14 (VF-14); Warwick-Edinburgh Mental Wellbeing Scale (WEMWBS); Well-Being Inventory (WBI); WHO Quality of Life-BREF (WHOQOL-BREF); WHO Well-Being Index (WHO-5)

**References**

- Abas, M.A., Leese, M., Punpuing, S., Tangchonlatip, K., and Jirapramupitak, T. (2009). Psychological wellbeing, physical impairments and rural aging in a developing country setting. *Health and Quality of Life Outcomes* 7(1)**,** 66. doi: http://dx.doi.org/10.1186/1477-7525-7-66.
- Abels, A.V. (2001). Career variables and life adjustment: A comparison of employed and unemployed adults who are blind or visually impaired. *Dissertation Abstracts International Section A: Humanities and Social Sciences* 62(1-A)**,** 86.
- Acton, J.H., Molik, B., Court, H., and Margrain, T.H. (2016a). Effect of a home visit-based low vision rehabilitation intervention on visual function outcomes: An exploratory randomized controlled trial. *Investigative Ophthalmology and Visual Science* 57(15)**,** 6662-6667. doi: http://dx.doi.org/10.1167/iovs.16-19901.
- Acton, J.H., Molik, B., Margrain, T.H., Binns, A., and Court, H. (2016b). Effect of rehabilitation worker input on visual function outcomes in individuals with low vision: Study protocol for a randomised controlled trial. *Trials* 17(1)**,** 105. doi: http://dx.doi.org/10.1186/s13063-016-1235-2.
- Adigun, K., Ladipo, M.M.A., Oluleye, T.S., and Olowookere, S.A. (2014). Quality of life in patients with visual impairment in Ibadan: A clinical study in primary care. *Journal of Multidisciplinary Healthcare* 7**,** 173-178. doi: http://dx.doi.org/10.2147/JMDH.S51359.
- Ahmmed, A.A., Ting, D.S.J., and Figueiredo, F.C. (2021). Epidemiology, economic and humanistic burdens of Ocular Surface Chemical Injury: A narrative review. *Ocular Surface* 20**,** 199-211. doi: http://dx.doi.org/10.1016/j.jtos.2021.02.006.
- Ajuwon, P.M., and Bieber, R. (2014). Vision impairment and quality of life. *International Public Health Journal* 6(4)**,** 341-354.
- Allen, E.D., Wood, C.M., Currie, S., and Jayamanne, D.G.R. (1999). Correlation between early, measurable improvement in quality of life and speed of visual rehabilitation after phacoemulsification. *Journal of Cataract and Refractive Surgery* 25(8)**,** 1135-1139. doi: http://dx.doi.org/10.1016/S0886-3350%2899%2900138-8.
- Alma, M.A., Van der Mei, S.F., Melis-Dankers, B.J., Van Tilburg, T.G., Groothoff, J.W., and Suurmeijer, T.P. (2011). Participation of the elderly after vision loss. *Disability and Rehabilitation* 33(1)**,** 63-72.
- Ang, G.S., Fenwick, E.K., Constantinou, M., Gan, A.T.L., Man, R.E.K., Casson, R.J., et al. (2020). Selective laser trabeculoplasty versus topical medication as initial glaucoma treatment: the glaucoma initial treatment study randomised clinical trial. *British Journal of Ophthalmology* 104(6)**,** 813-821.
- Anil, K., and Garip, G. (2018). Coping strategies, vision-related quality of life, and emotional health in managing retinitis pigmentosa: a survey study. *BMC Ophthalmology* 18(1)**,** 21. doi: http://dx.doi.org/10.1186/s12886-018-0689-2.
- Araki, A., Nakano, T., Ito, H., Oba, K., Ito, C., Mori, S., et al. (2004). Low well-being, cognitive impairment and visual impairment associated with functional disabilities in elderly Japanese patients with diabetes mellitus. *Geriatrics and Gerontology International* 4(1)**,** 15-24. doi: http://dx.doi.org/10.1111/j.1447-0594.2003.00108.x.
- Aroney, C., Fraser-Bell, S., Gillies, M.C., Lim, L.L., Fenwick, E.K., and Lamoureux, E.L. (2016). Vision-related quality of life outcomes in the BEVORDEX study: A clinical trial comparing ozurdex sustained release dexamethasone intravitreal implant and bevacizumab treatment for diabetic macular edema. *Investigative Ophthalmology and Visual Science* 57(13)**,** 5541-5546. doi: http://dx.doi.org/10.1167/iovs.16-19729.
- Assi, L., Chamseddine, F., Ibrahim, P., Sabbagh, H., Rosman, L., Congdon, N., et al. (2021). A Global Assessment of Eye Health and Quality of Life: A Systematic Review of Systematic Reviews. *JAMA Ophthalmology* (5). doi: http://dx.doi.org/10.1001/jamaophthalmol.2021.0146.
- Baker, H., Murdoch, I., Smith, A.F., Shilio, B., and Dhalla, K. (2020). The cost and quality of life impact of glaucoma in Tanzania: An observational study. *PLoS ONE* 15(6)**,** e0232796. doi: http://dx.doi.org/10.1371/journal.pone.0232796.
- Bambara, J.K., Wadley, V., Owsley, C., Martin, R.C., Porter, C., and Dreer, L.E. (2009). Family functioning and low vision: a systematic review. *Journal of Visual Impairment and Blindness* 103(3)**,** 137-149.
- Barr, W., Hodge, S., Leeven, M., Bowen, L., and Knox, P. (2012). Emotional support and counselling for people with visual impairment: Quantitative findings from a mixed methods pilot study. *Counselling and Psychotherapy Research* 12(4)**,** 294-302. doi: http://dx.doi.org/10.1080/14733145.2012.663776.
- Bazargan, M., Baker, R.S., and Bazargan, S.H. (2001). Sensory impairments and subjective well-being among aged African American persons. *Journals of Gerontology - Series B Psychological Sciences and Social Sciences* 56(5)**,** P268-P278. doi: http://dx.doi.org/10.1093/geronb/56.5.P268.
- Béchetoille, A., Arnould, B., Bron, A., Baudouin, C., Renard, J.-P., Sellem, E., et al. (2008). Measurement of health-related quality of life with glaucoma: validation of the Glau-QoL 36-item questionnaire. *Acta Ophthalmologica* 86(1)**,** 71-80. doi: 10.1111/j.1600-0420.2007.00999.x.
- Bell, S.L., and Foley, R. (2021). A(nother) time for nature? Situating non-human nature experiences within the emotional transitions of sight loss. *Social Science and Medicine* 276**,** 113867. doi: http://dx.doi.org/10.1016/j.socscimed.2021.113867.
- Ben-Zur, H., and Debi, Z. (2005). Optimism, social comparisons, and coping with vision loss in Israel. *Journal of Visual Impairment and Blindness* 99(3)**,** 151-164. doi: http://dx.doi.org/10.1177/0145482x0509900304.
- Bergeron, C.M., and Wanet-Defalque, M.-C. (2013). Psychological adaptation to visual impairment: The traditional grief process revised. *British Journal of Visual Impairment* 31(1)**,** 20-31. doi: http://dx.doi.org/10.1177/0264619612469371.
- Boerner, K., and Cimarolli, V.R. (2005). Optimizing rehabilitation for adults with visual impairment: Attention to life goals and their links to well-being. *Clinical Rehabilitation* 19(7)**,** 790-798. doi: http://dx.doi.org/10.1191/0269215505cr893oa.
- Boerner, K., Wang, S.W., and Cimarolli, V.R. (2006). The impact of functional loss: Nature and implications of life changes. *Journal of Loss and Trauma* 11(4)**,** 265-287. doi: http://dx.doi.org/10.1080/15325020600662625.
- Bray, N., Edwards, R.T., Brand, A., Hoare, Z., Taylor, J., and Dickinson, C. (2017). Portable electronic vision enhancement systems in comparison with optical magnifiers for near vision activities: an economic evaluation alongside a randomized crossover trial. *Acta Ophthalmologica* 95(5)**,** e415-e423. doi: http://dx.doi.org/10.1111/aos.13255.
- Breheny, K., Hollingworth, W., Kandiyali, R., Dixon, P., Loose, A., Craggs, P., et al. (2020). Assessing the construct validity and responsiveness of Preference-Based Measures (PBMs) in cataract surgery patients. *Quality of Life Research* 29(7)**,** 1935-1946. doi: http://dx.doi.org/10.1007/s11136-020-02443-3.
- Brenner, M.H., Curbow, B., Javitt, J.C., Legro, M.W., and Sommer, A. (1993). Vision change and quality of life in the elderly: response to cataract surgery and treatment of other chronic ocular conditions. *Archives of Ophthalmology* 111(5)**,** 680-685.
- Brown, C.M., Wong, E.Y.H., O'Connor, P.M., and Keeffe, J.E. (2009). Measurement of quality of life for people with diabetic retinopathy impairment. *Expert Review of Ophthalmology* 4(6)**,** 587-593. doi: http://dx.doi.org/10.1586/eop.09.57.
- Brunes, A., Nielsen, M.B., and Heir, T. (2018). Bullying among people with visual impairment: Prevalence, associated factors and relationship to self-efficacy and life satisfaction. *World Journal of Psychiatry* 8(1)**,** 43-50. doi: 10.5498/wjp.v8.i1.43.
- Brunnström, G., Sörensen, S., Alsterstad, K., and Sjöstrand, J. (2004). Quality of light and quality of life–the effect of lighting adaptation among people with low vision. *Ophthalmic and Physiological Optics* 24(4)**,** 274-280.
- Burmedi, D., Becker, S., Heyl, V., Wahl, H.-W., and Himmelsbach, I. (2002). Emotional and social consequences of age-related low vision. *Visual Impairment Research* 4(1)**,** 47-71.
- Burton, A.E., Gibson, J.M., and Shaw, R.L. (2016). How do older people with sight loss manage their general health? A qualitative study. *Disability and Rehabilitation* 38(23)**,** 2277-2285. doi: http://dx.doi.org/10.3109/09638288.2015.1123310.
- Burton, A.E., Shaw, R.L., and Gibson, J.M. (2015). Living together with age-related macular degeneration: An interpretative phenomenological analysis of sense-making within a dyadic relationship. *Journal of Health Psychology* 20(10)**,** 1285-1295. doi: http://dx.doi.org/10.1177/1359105313511134.
- Castle, C.L., Engward, H., and Kersey, T. (2021). Arts activity and well-being for visually impaired military veterans: a narrative discussion of current knowledge. *Public Health* 194**,** 232-237. doi: http://dx.doi.org/10.1016/j.puhe.2021.03.010.
- Chia, E.-M., Wang, J.J., Rochtchina, E., Smith, W., Cumming, R.R., and Mitchell, P. (2004). Impact of Bilateral Visual Impairment on Health-Related Quality of Life: The Blue Mountains Eye Study. *Investigative Ophthalmology and Visual Science* 45(1)**,** 71-76. doi: http://dx.doi.org/10.1167/iovs.03-0661.
- Chou, K.-L., and Chi, I. (2004). Combined effect of vision and hearing impairment on depression in elderly Chinese. *International Journal of Geriatric Psychiatry* 19(9)**,** 825-832. doi: http://dx.doi.org/10.1002/gps.1174.
- Cimarolli, V.R., and Boerner, K. (2005). Social support and well-being in adults who are visually impaired. *Journal of Visual Impairment and Blindness* 99(9).
- Clark, A., Ng, J.Q., Spilsbury, K., Semmens, J.B., Morlet, N., Tropiano, E., et al. (2008). Quality of life after postoperative endophthalmitis. *Clinical and Experimental Ophthalmology* 36(6)**,** 526-531. doi: http://dx.doi.org/10.1111/j.1442-9071.2008.01827.x.
- Cooper, O.A.E., Taylor, D.J., Crabb, D.P., Sim, D.A., and McBain, H. (2020). Psychological, social and everyday visual impact of diabetic macular oedema and diabetic retinopathy: a systematic review. *Diabetic Medicine* 37(6)**,** 924-933. doi: 10.1111/dme.14125.
- Cruess, A., Zlateva, G., Xu, X., and Rochon, S. (2007). Burden of illness of neovascular age-related macular degeneration in Canada. *Canadian Journal of Ophthalmology* 42(6)**,** 836-843. doi: http://dx.doi.org/10.3129/I07-153.
- Cruess, A.F., Gordon, K.D., Bellan, L., Mitchell, S., and Pezzullo, M.L. (2011). The cost of vision loss in Canada. 2. Results. *Canadian Journal of Ophthalmology* 46(4)**,** 315-318. doi: http://dx.doi.org/10.1016/j.jcjo.2011.06.006.
- Cumberland, P.M., Chianca, A., and Rahi, J.S. (2015). Laser refractive surgery in the UK Biobank study: Frequency, distribution by sociodemographic factors, and general health, happiness, and social participation outcomes. *Journal of Cataract and Refractive Surgery* 41(11)**,** 2466-2475. doi: http://dx.doi.org/10.1016/j.jcrs.2015.05.040.
- Dagnelie, G., and Kiser, A.K. (2008). Reported effects of non-traditional treatments and complementary and alternative medicine by retinitis pigmentosa patients. *Clinical and Experimental Optometry* 91(2)**,** 166-176. doi: http://dx.doi.org/10.1111/j.1444-0938.2007.00224.x.
- Das, A., MacKenzie, K., Adams, G., Xing, W., Dahlmann-Noor, A., Theodorou, M., et al. (2018). Visual functioning in adults with Idiopathic Infantile Nystagmus Syndrome (IINS). *Strabismus* 26(4)**,** 203-209. doi: http://dx.doi.org/10.1080/09273972.2018.1526958.
- De Bel, V., Spiegel, T., and Steverink, N. (2016). Keeping up appearances: the role of identity concealment in the workplace among adults with degenerative eye conditions and its relationship with wellbeing and career outcomes. *Disability and Rehabilitation* 38(7)**,** 627-636. doi: http://dx.doi.org/10.3109/09638288.2015.1055378.
- De La Jara, P.L., Erickson, P., Erickson, D., and Stapleton, F. (2010). Pre-operative quality of life and psychological factors that influence patient decision making in LASIK. *Eye* 24(2)**,** 270-275. doi: http://dx.doi.org/10.1038/eye.2009.115.
- Dean, W.C. (1999). *TELECARE with blind rehabilitation students/graduates.* Fuller Theological Seminary, School of Psychology.
- Delyfer, M.-N., Korobelnik, J.-F., Gaucher, D., Barale, P.-O., Ayello-Scheer, S., Mohand-Said, S., et al. (2020). Improved performance and safety from Argus II retinal prosthesis post-approval study in France. *Acta Ophthalmologica* 99(7)**,** e1212-e1221. doi: http://dx.doi.org/10.1111/aos.14728.
- Dersh, G. (1997). Evaluation of a program designed to enhance independence and psychological well-being in a cohort of visually impaired adults. *Dissertation Abstracts International: Section B: The Sciences and Engineering* 57(8-B)**,** 4972.
- Dev, M.K., Paudel, N., Joshi, N.D., Shah, D.N., and Subba, S. (2014). Psycho-social impact of visual impairment on health-related quality of life among nursing home residents. *BMC Health Services Research* 14(1)**,** 1-7. doi: http://dx.doi.org/10.1186/1472-6963-14-345.
- Deverell, L., Bradley, J., Foote, P., Bowden, M., and Meyer, D. (2019). Measuring the benefits of guide dog mobility with the Orientation and Mobility Outcomes (OMO) Tool. *Anthrozoos* 32(6)**,** 741-755. doi: http://dx.doi.org/10.1080/08927936.2019.1673036.
- Dillon, L., Keay, L., Gandhi, S., Tang, D., Liew, G., Mitchell, P., et al. (2021). Perspectives of people with late age-related macular degeneration on mental health and mental wellbeing programmes: a qualitative study. *Ophthalmic and Physiological Optics* 41(2)**,** 255-265. doi: http://dx.doi.org/10.1111/opo.12779.
- Dillon, L., Keay, L., Tang, D., Liew, G., Gopinath, B., Hackett, M., et al. (2020). Facilitators and barriers to participation in mental well-being programs by older Australians with vision impairment: community and stakeholder perspectives. *Eye* 34(7)**,** 1287-1295. doi: http://dx.doi.org/10.1038/s41433-020-0992-z.
- Dubey, S., Matah, P., Sahu, J., Mukherjee, S., Chauhan, L., Bedi, H., et al. (2020). Impact of visual impairment on the wellbeing and functional disability of patients with glaucoma in India. *Journal of Current Ophthalmology* 32(1)**,** 14-18. doi: http://dx.doi.org/10.1016/j.joco.2019.09.006.
- Elliott, T.R., Fletcher, D.C., Swanson, M., and Dreer, L.E. (2005). Social problem-solving abilities and psychological adjustment of persons in low vision rehabilitation. *Rehabilitation Psychology* 50(3)**,** 232-238. doi: http://dx.doi.org/10.1037/0090-5550.50.3.232.
- Engel, R.J., Welsh, R.L., and Lewis, L.J. (2000). Improving the well-being of vision-impaired older adults through orientation and mobility training and rehabilitation: An evaluation. *RE:view: Rehabilitation and Education for Blindness and Visual Impairment* 32(2)**,** 67-76.
- Erickson, D.B., Stapleton, F., Erickson, P., Du Toit, R., Giannakopoulos, E., and Holden, B. (2004). Development and validation of a multidimensional quality-of-life scale for myopia. *Optometry and Vision Science* 81(2)**,** 70-81.
- Estcourt, S., Vaidya, B., Quinn, A., and Shepherd, M. (2008). The impact of thyroid eye disease upon patients’ wellbeing: a qualitative analysis. *Clinical Endocrinology* 68(4)**,** 635-639.
- Fenwick, E., Lamoureux, E.L., Moore, K., Klaic, M., Borschmann, K., and Hill, K. (2009). Impact of the severity of distance and near-vision impairment on depression and vision-specific quality of life in older people living in residential care. *Investigative Ophthalmology and Visual Science* 50(9)**,** 4103-4109. doi: http://dx.doi.org/10.1167/iovs.08-3294.
- Fenwick, E., Pesudovs, K., Rees, G., Dirani, M., Kawasaki, R., Wong, T., et al. (2011). The impact of diabetic retinopathy: understanding the patient's perspective. *British Journal of Ophthalmology* 95(6)**,** 774-782.
- Fenwick, E., Rees, G., Pesudovs, K., Dirani, M., Kawasaki, R., Wong, T.Y., et al. (2012a). Social and emotional impact of diabetic retinopathy: a review. *Clinical and Experimental Ophthalmology* 40(1)**,** 27-38.
- Fenwick, E.K., Pesudovs, K., Khadka, J., Dirani, M., Rees, G., Wong, T.Y., et al. (2012b). The impact of diabetic retinopathy on quality of life: Qualitative findings from an item bank development project. *Quality of Life Research* 21(10)**,** 1771-1782.
- Fenwick, E., Marella, M., Finger, R.P., Holz, F.G., Pesudovs, K., and Lamoureux, E.L. (2012c). Rasch analysis reveals problems with multiplicative scoring in the macular disease quality of life questionnaire. *Ophthalmology* 119(11)**,** 2351-2357. doi: http://dx.doi.org/10.1016/j.ophtha.2012.05.031.
- Fenwick, E.K., Ong, P.G., Cheung, C.M.G., Tan, G., Lee, S.Y., Yeo, I., et al. (2017). The impact of typical neovascular age-related macular degeneration and polypoidal choroidal vasculopathy on vision-related quality of life in Asian patients. *British Journal of Ophthalmology* 101(5)**,** 591-596. doi: http://dx.doi.org/10.1136/bjophthalmol-2016-308541.
- Fenwick, E.K., Rees, G., Xie, J., Holloway, E., Ong, P.G., Lim, B., et al. (2016). Assessment of the psychometric properties of the Chinese Impact of Vision Impairment questionnaire in a population-based study: findings from the Singapore Chinese Eye Study. *Quality of Life Research* 25(4)**,** 871-880. doi: http://dx.doi.org/10.1007/s11136-015-1141-1.
- Fenwick, E.K., Man, R.E.K., Lamoureux, E.L., Aung, T., and Ramulu, P. (2020). Beyond intraocular pressure: Optimizing patient-reported outcomes in glaucoma. *Progress in Retinal and Eye Research* 76**,** 100801. doi: http://dx.doi.org/10.1016/j.preteyeres.2019.100801.
- Finger, R.P., Fenwick, E., Marella, M., Charbel Issa, P., Scholl, H.P.N., Holz, F.G., et al. (2011). The relative impact of vision impairment and cardiovascular disease on quality of life: The example of Pseudoxanthoma elasticum. *Health and Quality of Life Outcomes***,** 113. doi: http://dx.doi.org/10.1186/1477-7525-9-113.
- Finger, R.P., Guymer, R.H., Keeffe, J.E., and Gillies, M.C. (2014a). The impact of anti-vascular endothelial growth factor treatment on quality of life in neovascular age-related macular degeneration. *Ophthalmology* 121(6)**,** 1246-1251. doi: http://dx.doi.org/10.1016/j.ophtha.2013.12.032.
- Finger, R.P., Tellis, B., Keeffe, J.E., Ayton, L.N., Guymer, R.H., and Crewe, J. (2014b). Developing the impact of vision impairment-very low vision (IVI-VLV) questionnaire as part of the LoVADA protocol. *Investigative Ophthalmology and Visual Science* 55(10)**,** 6150-6158. doi: http://dx.doi.org/10.1167/iovs.14-14731.
- Freitas, C., Oliveiros, B.M., Marques, E., and Leite, E.B. (1995). Effect of photorefractive keratectomy on visual functioning and quality of life. *Journal of Refractive Surgery* 11(3 SUPPL.)**,** S327-S334.
- Friedman, E.A., L'Esperance Jr, F.A., Brown, C.D., and Berman, D.H. (2003). Treating azotemia-induced anemia with erythropoietin improves diabetic eye disease. *Kidney International, Supplement* 64(87)**,** S57-S63.
- Gan, A.T.L., Fenwick, E.K., Lamoureux, E.L., Bansback, N., Ratcliffe, J., Burgess, L., et al. (2020a). Validation of a novel diabetic retinopathy utility index using discrete choice experiments. *British Journal of Ophthalmology* 104(2)**,** 188-193. doi: http://dx.doi.org/10.1136/bjophthalmol-2019-313899.
- Gan, A.T.L., Kumari, N., Wong, C., Aravindhan, A., Gupta, P., Wang, J.J., et al. (2019). Beyond vision loss: The independent impact of diabetic retinopathy on vision-related quality of life in a Chinese Singaporean population. *British Journal of Ophthalmology* 103(9)**,** 1314-1319. doi: http://dx.doi.org/10.1136/bjophthalmol-2018-313082.
- Gan, A.T.L., Man, R.E.K., Fenwick, E.K., Finkelstein, E.A., Constantinou, M., Coote, M., et al. (2020b). Effectiveness of an innovative and comprehensive eye care model for individuals in residential care facilities: Results of the residential ocular care (ROC) multicentred randomised controlled trial. *British Journal of Ophthalmology* 104(11)**,** 1585-1590. doi: http://dx.doi.org/10.1136/bjophthalmol-2019-315620.
- Garcia, G.A., Khoshnevis, M., Gale, J., Frousiakis, S.E., Hwang, T.J., Poincenot, L., et al. (2017). Profound vision loss impairs psychological well-being in young and middle-aged individuals. *Clinical Ophthalmology (Auckland, NZ)* 11**,** 417.
- Geruschat, D.R., Dagnelie, G., Deremeik, J., Flax, M., Tanna, N., Bianchi, M., et al. (2015). FLORATM: Phase I development of a functional vision assessment for prosthetic vision users. *Clinical and Experimental Optometry* 98(4)**,** 342-347. doi: http://dx.doi.org/10.1111/cxo.12242.
- Ghazi-Nouri, S.M.S., Adams, Z.C., Charteris, D.G., Rubin, G.S., and Tranos, P.G. (2004). Visual function and subjective perception of visual ability after macular hole surgery. *American Journal of Ophthalmology* 138(6)**,** 995-1002. doi: http://dx.doi.org/10.1016/j.ajo.2004.07.049.
- Gleeson, M., Auld, R., Keay, L., Sherrington, C., and Lo, S. (2017). Impact of the Alexander technique on well-being: a randomised controlled trial involving older adults with visual impairment. *Clinical and Experimental Optometry* 100(6)**,** 633-641. doi: http://dx.doi.org/10.1111/cxo.12517.
- Gleeson, M., Sherrington, C., Borkowski, E., and Keay, L. (2014). Improving balance and mobility in people over 50 years of age with vision impairments: can the Alexander Technique help? A study protocol for the VISIBILITY randomised controlled trial. *Injury Prevention: Journal of the International Society for Child and Adolescent Injury Prevention* 20(1)**,** e3. doi: http://dx.doi.org/10.1136/injuryprev-2012-040726.
- Glen, F.C., and Crabb, D.P. (2015). Living with glaucoma: a qualitative study of functional implications and patients’ coping behaviours. *BMC Ophthalmology* 15(1)**,** 1-15.
- Glick, P., Luoto, J., Orrs, M.S., Tasfaw, A.K., Oliva, M.S., Tabin, G.C., et al. (2019). The individual and household impacts of cataract surgery on older blind adults in Ethiopia. *Ophthalmic Epidemiology* 26(1)**,** 7-18. doi: http://dx.doi.org/10.1080/09286586.2018.1504310.
- Godier-McBard, L.R., Castle, C.L., Heinze, N., Hussain, S.F., Borowski, S., Vogt, D.S., et al. (2020). A preliminary investigation of the well-being of visually impaired ex-service personnel in the United Kingdom. *British Journal of Visual Impairment* 40(2), 274-288. doi: http://dx.doi.org/10.1177/0264619620973683.
- Gordon, K.D., Cruess, A.F., Bellan, L., Mitchell, S., and Pezzullo, M.L. (2011). The cost of vision loss in Canada. 1. Methodology. *Canadian Journal of Ophthalmology* 46(4)**,** 310-314.
- Gothwal, V.K., and Bharani, S. (2015). Outcomes of multidisciplinary low vision rehabilitation in adults. *Investigative Ophthalmology and Visual Science* 56(12)**,** 7451-7461. doi: http://dx.doi.org/10.1167/iovs.15-16892.
- Gothwal, V.K., Reddy, S.P., Fathima, A., Bharani, S., Sumalini, R., Bagga, D.K., et al. (2013). Assessment of the impact of keratoconus on vision-related quality of life. *Investigative Ophthalmology and Visual Science* 54(4)**,** 2902-2910. doi: http://dx.doi.org/10.1167/iovs.12-10783.
- Guerette, A.R., and Smedema, S.M. (2011). The relationship of perceived social support with well-being in adults with visual impairments. *Journal of Visual Impairment and Blindness* 105(7)**,** 425-439.
- Hackney, M.E., Echt, K.V., Hall, C.D., and Wolf, S.L. (2013). Dancing for balance: Feasibility and efficacy in oldest-old adults with visual impairment. *Nursing Research* 62(2)**,** 138-143. doi: http://dx.doi.org/10.1097/NNR.0b013e318283f68e.
- Haibach-Beach, P., McNamera, S., and Lieberman, L. (2020). Home-based balance pilot intervention for adults with visual impairments. *British Journal of Visual Impairment* 40(2), 145-159. doi: http://dx.doi.org/10.1177/0264619620935937.
- Harada, S., Nishiwaki, Y., Michikawa, T., Kikuchi, Y., Iwasawa, S., Nakano, M., et al. (2008). Gender difference in the relationships between vision and hearing impairments and negative well-being. *Preventive Medicine* 47(4)**,** 433-437. doi: http://dx.doi.org/10.1016/j.ypmed.2008.06.011.
- Hasan, M.K., Ashraf, M., Narasimhan, P., and Aggarwal, R. (2018). Expanding freedoms of people with visual impairment through information and communication technologies: Narratives from Bangladesh. *International Journal of Disability Management* 13. doi: http://dx.doi.org/10.1017/idm.2018.7.
- Heine, C., and Browning, C.J. (2002). Communication and psychosocial consequences of sensory loss in older adults: overview and rehabilitation directions. *Disability and Rehabilitation* 24(15)**,** 763-773. doi: 10.1080/09638280210129162.
- Heine, C., and Browning, C.J. (2004). The communication and psychosocial perceptions of older adults with sensory loss: A qualitative study. *Ageing and Society* 24(1)**,** 113-130.
- Heine, C., Gong, C.H., Feldman, S., and Browning, C. (2020). Older women in Australia: Facing the challenges of dual sensory loss. *International Journal of Environmental Research and Public Health* 17(1)**,** 263. doi: http://dx.doi.org/10.3390/ijerph17010263.
- Hernandez Trillo, A., and Dickinson, C.M. (2012). The impact of visual and nonvisual factors on quality of life and adaptation in adults with visual impairment. *Investigative Ophthalmology and Visual Science* 53(7)**,** 4234-4241. doi: http://dx.doi.org/10.1167/iovs.12-9580.
- Heyl, V., and Wahl, H.-W. (2014). Experiencing age-related vision and hearing impairment: The psychosocial dimension. *Journal of Clinical Outcomes Management* 21(7)**,** 323-335.
- Hodge, S., Barr, W., Bowen, L., Leeven, M., and Knox, P. (2013). Exploring the role of an emotional support and counselling service for people with visual impairments. *British Journal of Visual Impairment* 31(1)**,** 5-19. doi: http://dx.doi.org/10.1177/0264619612465168.
- Holmes, W., Durrant, K., Shajehan, R., Kitnasamy, S., Abeywickrama, C., Arsath, Y., et al. (2018). Impact of vision impairment and self-reported barriers to vision care: The views of elders in Nuwara Eliya district, Sri Lanka. *Global Public Health* 13(5)**,** 642-655. doi: http://dx.doi.org/10.1080/17441692.2016.1241816.
- Holz, F.G., Finger, R.P., Fenwick, E., Marella, M., Dirani, M., Chiang, P.P.-C., et al. (2011). The impact of vision impairment on vision-specific quality of life in Germany. *Investigative Ophthalmology and Visual Science* 52(6)**,** 3613-3619. doi: http://dx.doi.org/10.1167/iovs.10-7127.
- Hooper, C.Y., Lim, L., Hunt, N., Guymer, R.H., Pallant, J.F., Keeffe, J.E., et al. (2007). Impact of cataract surgery on quality of life in patients with early age-related macular degeneration. *Optometry and Vision Science* 84(8)**,** 683-688. doi: http://dx.doi.org/10.1097/OPX.0b013e31812f755f.
- Horowitz, A. (2004). The prevalence and consequences of vision impairment in later life. *Topics in Geriatric Rehabilitation* 20(3)**,** 185-195.
- Horowitz, A., and Reinhardt, J.P. (1998). Development of the adaptation to age-related vision loss scale. *Journal of Visual Impairment and Blindness* 92(1)**,** 30-41.
- Iuliano, E., Aquino, G., Fiorilli, G., Calcagno, G., Di Cagno, A., Battaglia, C., et al. (2013). Psychological well-being and social participation assessment in visually impaired subjects playing Torball: A controlled study. *Research in Developmental Disabilities* 34(4)**,** 1204-1209. doi: http://dx.doi.org/10.1016/j.ridd.2012.11.010.
- Jackson, M.L., Schoessow, K.A., Selivanova, A., and Wallis, J. (2017). Adding access to a video magnifier to standard vision rehabilitation: initial results on reading performance and well-being from a prospective, randomized study. *Digital Journal of Ophthalmology : DJO* 23(1)**,** 1-10. doi: http://dx.doi.org/10.5693/djo.01.2017.02.001.
- Jackson, S.E., Hackett, R.A., Pardhan, S., Smith, L., and Steptoe, A. (2019). Association of perceived discrimination with emotional well-being in older adults with visual impairment. *JAMA Ophthalmology* 137(7)**,** 825-832.
- Kahaly, G.J., Hardt, J., Petrak, F., and Egle, U.T. (2002). Psychosocial factors in subjects with thyroid-associated ophthalmopathy. *Thyroid* 12(3)**,** 237-239.
- Kaleemunnisha, S., Sudharshan, S., and Biswas, J. (2014). Quality of life in non-infectious uveitis patients on immunosuppressive therapy. *Middle East African Journal of Ophthalmology* 21(3)**,** 225-231. doi: http://dx.doi.org/10.4103/0974-9233.134675.
- Kaur, D., Gupta, A., and Singh, G. (2012). Perspectives on quality of life in glaucoma. *Journal of Current Glaucoma Practice* 6(1)**,** 9-12.
- Kekecs, Z., Jakubovits, E., Varga, K., and Gombos, K. (2014). Effects of patient education and therapeutic suggestions on cataract surgery patients: A randomized controlled clinical trial. *Patient Education and Counseling* 94(1)**,** 116-122. doi: http://dx.doi.org/10.1016/j.pec.2013.09.019.
- Kelly, N.K., Chattopadhyay, A., Ebert, C.D., Berlinberg, E.J., Gonzales, J.A., Murugan, S.B., et al. (2021). Health- and Vision-Related Quality of Life in a Randomized Controlled Trial Comparing Methotrexate and Mycophenolate Mofetil for Uveitis. *Ophthalmology* 128(9). doi: http://dx.doi.org/10.1016/j.ophtha.2021.02.024.
- Khadka, J., McAlinden, C., Craig, J.E., Fenwick, E.K., Lamoureux, E.L., and Pesudovs, K. (2015). Identifying content for the glaucoma-specific item bank to measure quality-of-life parameters. *Journal of Glaucoma* 24(1)**,** 12-19.
- Khoo, K., Gupta, P., Man, R.E.K., Fenwick, E.K., Rees, G., and Lamoureux, E.L. (2019). The relationship between diabetic retinopathy and psychosocial functioning: a systematic review. *Quality of Life Research* 28(8)**,** 2017-2039. doi: http://dx.doi.org/10.1007/s11136-019-02165-1.
- Kirkcaldy, A., and Barr, W. (2011). Coming to terms with sight loss: Impact of RNIB's 'Finding your Feet' programmes on participants' quality of life. *British Journal of Visual Impairment* 29(2)**,** 145-154. doi: http://dx.doi.org/10.1177/0264619611401798.
- Kowalski, J.W., Rentz, A.M., Walt, J.G., Lloyd, A., Lee, J., Young, T.A., et al. (2012). Rasch analysis in the development of a simplified version of the National Eye Institute Visual-Function Questionnaire-25 for utility estimation. *Quality of Life Research* 21(2)**,** 323-334.
- Kutzbach, B.R., MacDonald, J.T., Merrill, K.S., Hogue, K.M., Downes, S.J., Holleschau, A.M., et al. (2009). Evaluation of vision-specific quality-of-life in albinism. *Journal of AAPOS* 13(2)**,** 191-195. doi: http://dx.doi.org/10.1016/j.jaapos.2008.10.008.
- La Grow, S.J., Towers, A., Yeung, P., Alpass, F., and Stephens, C. (2015). The relationship between loneliness and perceived quality of life among older persons with visual impairments. *Journal of Visual Impairment and Blindness* 109(6)**,** 487-499.
- Lang, C., and Brooks, R. (2015). The experience of older adults with sight loss participating in audio book groups. *Journal of Occupational Science* 22(3)**,** 277-290. doi: http://dx.doi.org/10.1080/14427591.2013.851763.
- Lange, R., Kumagai, A., Weiss, S., Zaffke, K.B., Day, S., Wicker, D., et al. (2021). Vision-related quality of life in adults with severe peripheral vision loss: a qualitative interview study. *Journal of Patient-reported Outcomes* 5(1)**,** 7. doi: 10.1186/s41687-020-00281-y.
- Layat, I., Challe, G., LeHoang, P., Bodaghi, B., and Touitou, V. (2017). Neuro-ophthalmological conditions: Study of the clinical care pathway. *Journal Francais d'Ophtalmologie* 40(6)**,** e169-e175. doi: http://dx.doi.org/10.1016/j.jfo.2017.05.004.
- Lee, P.P., Whitcup, S.M., Hays, R.D., Spritzer, K., and Javitt, J. (1995). The relationship between visual acuity and functioning and well-being among diabetics. *Quality of Life Research* 4(4)**,** 319-323. doi: http://dx.doi.org/10.1007/BF01593884.
- Legro, M.W. (1991). Quality of life and cataracts: A review of patient-centered studies of cataract surgery outcomes. *Ophthalmic Surgery* 22(8)**,** 431-443.
- Lehane, C.M., Dammeyer, J., and Elsass, P. (2017a). Sensory loss and its consequences for couples’ psychosocial and relational wellbeing: an integrative review. *Aging and Mental Health* 21(4)**,** 337-347.
- Lehane, C.M., Dammeyer, J., Hovaldt, H.B., and Elsass, P. (2017b). Sexuality and Well-Being Among Couples Living with Acquired Deafblindness. *Sexuality and Disability* 35(2)**,** 135-146. doi: http://dx.doi.org/10.1007/s11195-016-9470-8.
- Li, W., Zhong, B., Liu, X., Huang, X.e., Dai, X., Hu, Q., et al. (2013). Depressive symptoms among the visually disabled in Wuhan: An epidemiological survey. *Shanghai Archives of Psychiatry* 25(5)**,** 306-313.
- Liljas, A.E.M., Jones, A., Cadar, D., Steptoe, A., and Lassale, C. (2020). Association of Multisensory Impairment with Quality of Life and Depression in English Older Adults. *JAMA Otolaryngology - Head and Neck Surgery* 146(3)**,** 278-285. doi: http://dx.doi.org/10.1001/jamaoto.2019.4470.
- Lindo, G., and Nordholm, L. (1999). Adaptation strategies, well-being, and activities of daily living among people with low vision. *Journal of Visual Impairment and Blindness* 93(7)**,** 434-446.
- Liu, Z., Huang, J., Li, S., Jin, L., Wang, X., Qian, D., et al. (2016). Visual impairment, but not hearing impairment, is independently associated with lower subjective well-being among individuals over 95 years of age: A population-based study. *Archives of Gerontology and Geriatrics* 62**,** 30-35. doi: http://dx.doi.org/10.1016/j.archger.2015.10.011.
- Lotery, A., Xu, X., Zlatava, G., and Loftus, J. (2007). Burden of illness, visual impairment and health resource utilisation of patients with neovascular age-related macular degeneration: results from the UK cohort of a five-country cross-sectional study. *British Journal of Ophthalmology* 91(10)**,** 1303-1307.
- Luu, W., Kalloniatis, M., Tu, M., Zangerl, B., Ly, A., Bartley, E., et al. (2020). A holistic model of low vision care for improving vision-related quality of life. *Clinical and Experimental Optometry* 103(6)**,** 733-741. doi: http://dx.doi.org/10.1111/cxo.13054.
- Madsen, H.S., Martiny, K., Ba-Ali, S., Lund-Andersen, H., and Hageman, I. (2021). Light therapy for seasonal affective disorder in visual impairment and blindness- A pilot study. *Acta Neuropsychiatrica* 33(4), 191-199. doi: http://dx.doi.org/10.1017/neu.2021.6.
- Man, R., Fenwick, E., Lamoureux, E., Ang, M., and Wilkins, M. (2018). Impact of type i Boston keratoprosthesis implantation on vision-related quality of life. *British Journal of Ophthalmology* 102(7)**,** 878-881. doi: http://dx.doi.org/10.1136/bjophthalmol-2017-310745.
- Marakis, T.P., Koutsandrea, C., and Poulou, M.S. (2020). The impact of vision impairment on vision-related quality of life of patients with neovascular age-related macular degeneration. *European Journal of Ophthalmology* 32(1), 481-490. doi: http://dx.doi.org/10.1177/1120672120972625.
- Marques, A.P., Zhang, J.H., Bastawrous, A., Gordon, I., Ramke, J., Cairns, J., et al. (2020). Estimating the global cost of vision impairment and its major causes: protocol for a systematic review. *BMJ Open* 10(9)**,** e036689. doi: http://dx.doi.org/10.1136/bmjopen-2019-036689.
- Marques-Brocksopp, L. (2012). The broad reach of the wellbeing debate: Emotional wellbeing and vision loss. *British Journal of Visual Impairment* 30(1)**,** 50-55. doi: http://dx.doi.org/10.1177/0264619611428244.
- Marques-Brocksopp, L. (2014). Mindfulness, spiritual well-being, and visual impairment: An exploratory study. *British Journal of Visual Impairment* 32(2)**,** 108-123. doi: http://dx.doi.org/10.1177/0264619614528343.
- Matthews, K., Nazroo, J., and Whillans, J. (2017). The consequences of self-reported vision change in later-life: evidence from the English Longitudinal Study of Ageing. *Public Health* 142**,** 7-14. doi: http://dx.doi.org/10.1016/j.puhe.2016.09.034.
- McCormack, K. (2021). Independence versus abandonment among people with visual impairments in Puerto Rico throughout the 2017 hurricane season. *Disability and Society*, 1-22. doi: http://dx.doi.org/10.1080/09687599.2021.1888282.
- McGwin, G., and Owsley, C. (2007). Measuring the personal burden of eye disease and vision impairment. *Ophthalmic Epidemiology* 14(4)**,** 188-191. doi: http://dx.doi.org/10.1080/09286580701410315.
- McIlvane, J.M., and Reinhardt, J.P. (2001). Interactive effect of support from family and friends in visually impaired elders. *Journals of Gerontology - Series B Psychological Sciences and Social Sciences* 56(6)**,** P374-P382.
- McKean-Cowdin, R., Choudhury, F., Varma, R., Azen, S.P., Wu, J., and Hays, R.D. (2010). Longitudinal changes in visual acuity and health-related quality of life: The Los Angeles latino eye study. *Ophthalmology* 117(10)**,** 1900. doi: http://dx.doi.org/10.1016/j.ophtha.2010.01.059.
- McLean, R.J., Maconachie, G.D., Gottlob, I., and Maltby, J. (2016). The development of a nystagmus-specific quality-of-life questionnaire. *Ophthalmology* 123(9)**,** 2023-2027.
- McManus, S., and Lord, C. (2012). Circumstances of People with Sight Loss: Secondary Analysis of Understanding Society and the Life Opportunities Survey. *Natcen Report and RNIB*.
- McMullan, K.S., and Butler, M. (2019). Low vision and mobility scooters: the experiences of individuals with low vision who use mobility scooters. *Disability and Rehabilitation. Assistive technology* 14(6)**,** 574-580. doi: http://dx.doi.org/10.1080/17483107.2018.1470685.
- Menon, V., Treen, T., Burdon, M.A., and Batra, R. (2020). Impact of the eye clinic liaison officer at an NHS foundation trust: a retrospective study. *BMJ Open Ophthalmology* 5(1)**,** e000587. doi: 10.1136/bmjophth-2020-000587.
- Mirandola, D., Monaci, M., Vannuzzi, A., Manetti, M., Marini, M., Miccinesi, G., et al. (2019). Psychological well-being and quality of life in visually impaired baseball players: An Italian national survey. *PLoS ONE* 14(6)**,** e0218124. doi: http://dx.doi.org/10.1371/journal.pone.0218124.
- Misajon, R., Hawthorne, G., Richardson, J., Barton, J., Peacock, S., Iezzi, A., et al. (2005). Vision and quality of life: the development of a utility measure. *Investigative Ophthalmology and Visual Science* 46(11)**,** 4007-4015.
- Mitchell, J., and Bradley, C. (2001). Psychometric evaluation of the 12-item Well-being Questionnaire for use with people with macular disease. *Quality of Life Research* 10(5)**,** 465-473. doi: http://dx.doi.org/10.1023/A:1012540100613.
- Mitchell, J., and Bradley, C. (2006). Quality of life in age-related macular degeneration: a review of the literature. *Health and Quality of Life Outcomes* 4(1)**,** 1-20.
- Mojon-Azzi, S.M., Mojon, D.S., and Sousa-Poza, A. (2010). Impact of low vision on employment. *Ophthalmologica* 224(6)**,** 381-388. doi: http://dx.doi.org/10.1159/000316688.
- Mojon-Azzi, S.M., Sousa-Poza, A., and Mojon, D.S. (2008). Impact of low vision on well-being in 10 European countries. *Ophthalmologica* 222(3)**,** 205-212. doi: http://dx.doi.org/10.1159/000126085.
- Mozaffar Jalali, M.D., Moussavi, M.S., Amin Yazdi, S.A., and Salehi Fadardi, J. (2014). Effectiveness of Rational Emotive Behavior Therapy on Psychological Well-Being of People with Late Blindness. *Journal of Rational - Emotive and Cognitive - Behavior Therapy* 32(4)**,** 233-247. doi: http://dx.doi.org/10.1007/s10942-014-0191-6.
- Nastasi, J.A. (2014). *The occupational lives of individuals with visual impairment.* Towson University.
- Naylor, P.D., and Labbe, E.E. (2017). Exploring the effects of group therapy for the visually impaired. *British Journal of Visual Impairment* 35(1)**,** 18-28. doi: http://dx.doi.org/10.1177/0264619616671976.
- Nguyen, B.J., Kim, Y., Park, K., Chen, A.J., Chen, S., Van Fossan, D., et al. (2018). Improvement in patient-reported quality of life outcomes in severely visually impaired individuals using the aira assistive technology system. *Translational Vision Science and Technology* 7(5)**,** 30. doi: http://dx.doi.org/10.1167/tvst.7.5.30.
- Nguyen, J.H., Nguyen-Cuu, J., Yee, K.M., Yu, F., Mamou, J., Ketterling, J., et al. (2019). Assessment of Vitreous Structure and Visual Function after Neodymium:Yttrium-Aluminum-Garnet Laser Vitreolysis. *Ophthalmology* 126(11)**,** 1517-1526. doi: http://dx.doi.org/10.1016/j.ophtha.2019.06.021.
- Nyman, S.R., Gosney, M.A., and Victor, C.R. (2010a). Emotional well-being in people with sight loss: Lessons from the grey literature. *British Journal of Visual Impairment* 28(3)**,** 175-203. doi: http://dx.doi.org/10.1177/0264619610374171.
- Nyman, S.R., Gosney, M.A., and Victor, C.R. (2010b). Psychosocial impact of visual impairment in working-age adults. *British Journal of Ophthalmology* 94(11)**,** 1427-1431.
- Nyman, S.R., Dibb, B., Victor, C.R., and Gosney, M.A. (2012). Emotional well-being and adjustment to vision loss in later life: a meta-synthesis of qualitative studies. *Disability and Rehabilitation* 34(12)**,** 971-981. doi: 10.3109/09638288.2011.626487.
- Ong, P.G., Man, R.E.K., Fenwick, E.K., Sabanayagam, C., Cheng, C.-Y., Wong, T.Y., et al. (2017). Vision impairment and major eye diseases reduce vision-specific emotional well-being in a Chinese population. *British Journal of Ophthalmology* 101(5)**,** 686-690. doi: http://dx.doi.org/10.1136/bjophthalmol-2016-308701.
- Onuigbo, L.N., Onyishi, C.N., and Eseadi, C. (2020). Predictive Influence of Irrational Beliefs on Self-esteem of University Students with Late Blindness. *Journal of Rational - Emotive and Cognitive - Behavior Therapy* 38(4), 472-497. doi: http://dx.doi.org/10.1007/s10942-020-00347-2.
- Orr, A.L. (1991). The psychosocial aspects of aging and vision loss. *Journal of Gerontological Social Work* 17(3-4)**,** 1-14. doi: http://dx.doi.org/10.1300/J083V17N03_01.
- Pabon, S., Kaleem, M.A., and Sunness, J.S. (2017). Low Vision Therapy for Glaucoma Patients. *Current Ophthalmology Reports* 5(1)**,** 85-92. doi: http://dx.doi.org/10.1007/s40135-017-0124-5.
- Pankow, L., Luchins, D., Studebaker, J., and Chettleburgh, D. (2004). Evaluation of a Vision Rehabilitation Program for Older Adults With Visual Impairment. *Topics in Geriatric Rehabilitation* 20(3)**,** 223-232.
- Patty, N.J.S., Koopmanschap, M., and Holtzer-Goor, K. (2018). A cost-effectiveness study of ICT training among the visually impaired in the Netherlands. *BMC Ophthalmology* 18(1)**,** 98. doi: http://dx.doi.org/10.1186/s12886-018-0761-y.
- Paudel, P., Burnett, A., Naduvilath, T., Fricke, T.R., Khadka, J., and Hani, Y. (2015). Papua New Guinea vision-specific quality of life questionnaire: A new patient-reported outcome instrument to assess the impact of impaired vision. *Clinical and Experimental Ophthalmology* 43(3)**,** 202-213. doi: http://dx.doi.org/10.1111/ceo.12413.
- Paz, S.H., Slotkin, J., McKean-Cowdin, R., Lee, P., Owsley, C., Vitale, S., et al. (2013). Development of a vision-targeted health-related quality of life item measure. *Quality of Life Research* 22(9)**,** 2477-2487.
- Pesudovs, K., Caudle, L.E., Rees, G., and Lamoureux, E.L. (2008). Validity of a visual impairment questionnaire in measuring cataract surgery outcomes. *Journal of Cataract and Refractive Surgery* 34(6)**,** 925-933. doi: http://dx.doi.org/10.1016/j.jcrs.2007.12.052.
- Peters, T., Wilhelm, B., Klingberg, S., and Zrenner, E. (2013). Emotional wellbeing of blind patients in a pilot trial with subretinal implants. *Graefe's Archive for Clinical and Experimental Ophthalmology* 251(6)**,** 1489-1493. doi: http://dx.doi.org/10.1007/s00417-012-2210-6.
- Peyron, F., Garweg, J.G., Wallon, M., Descloux, E., Rolland, M., and Barth, J. (2011). Long-term impact of treated congenital toxoplasmosis on quality of life and visual performance. *The Pediatric Infectious Disease Journal* 30(7)**,** 597-600. doi: 10.1097/INF.0b013e31820bb5f3.
- Pilling, R.F., Thompson, J.R., and Gottlob, I. (2005). Social and visual function in nystagmus. *British Journal of Ophthalmology* 89(10)**,** 1278-1281. doi: http://dx.doi.org/10.1136/bjo.2005.070045.
- Pinniger, R., Brown, R.F., Thorsteinsson, E.B., and McKinley, P. (2013). Tango programme for individuals with age-related macular degeneration. *British Journal of Visual Impairment* 31(1)**,** 47-59. doi: http://dx.doi.org/10.1177/0264619612470651http://dx.doi.org/10.1177/0264619612470651.
- Pinquart, M., and Pfeiffer, J.P. (2011). Psychological well-being in visually impaired and unimpaired individuals: A meta-analysis. *British Journal of Visual Impairment* 29(1)**,** 27-45. doi: http://dx.doi.org/10.1177/0264619610389572.
- Pondorfer, S.G., Terheyden, J.H., Holz, F.G., Finger, R.P., Overhoff, H., and Stasch-Bouws, J. (2021). Development of the vision impairment in low luminance questionnaire. *Translational Vision Science and Technology* 10(1)**,** 1-11. doi: http://dx.doi.org/10.1167/tvst.10.1.5.
- Prem Senthil, M., Khadka, J., Gilhotra, J.S., Simon, S., and Pesudovs, K. (2017). Exploring the quality of life issues in people with retinal diseases: a qualitative study. *Journal of Patient-reported Outcomes* 1(1)**,** 15. doi: 10.1186/s41687-017-0023-4.
- Rafaely, L., Carmel, S., and Bachner, Y.G. (2018). Subjective well-being of visually impaired older adults living in the community. *Aging and Mental Health* 22(9)**,** 1223-1231. doi: http://dx.doi.org/10.1080/13607863.2017.1341469.
- Rajak, S.N., Burton, M.J., Habtamu, E., Wondie, T., Aweke, S., Tadesse, Z., et al. (2016). Impact of Trichiasis Surgery on Quality of Life: A Longitudinal Study in Ethiopia. *PLoS Neglected Tropical Diseases* 10(4)**,** e0004627. doi: http://dx.doi.org/10.1371/journal.pntd.0004627.
- Ratanasukon, M., Bhurayanontachai, P., Jirarattanasopa, P., and Tongsomboon, J. (2016). The impact of vision impairment (IVI) questionnaire; validation of the Thai-version and the implementation on vision-related quality of life in Thai rural community. *PLoS ONE* 11(5)**,** e0155509. doi: http://dx.doi.org/10.1371/journal.pone.0155509.
[truncated: 20,202 more chars]
