# Supplementary material for: Conceptualizations of well-being in adults with visual impairment: A scoping review
Source: Front Psychol. 2022 Sep 26;13:964537. doi: 10.3389/fpsyg.2022.964537 (PMC9549791; doi:10.3389/fpsyg.2022.964537)
Supplement: Supplementary file 2 [file Table_2.doc]

Supplementary Table 2 - Overview of domains and indicators of general, negative and positive well-being

| General well-being (*n*=101) | | | Negative well-being (*n*=3) | | | Positive well-being (*n*=4) | | |
| --- | --- | --- | --- | --- | --- | --- | --- | --- |
|  | ***n*** | **%** |  | ***n*** | **%** |  | ***n*** | **%** |
| Hedonia | **28** | **27.7** |  | **1** | **33.3** |  | **1** | **25.0** |
| *Life satisfaction* | 17 | 16.8 | Mood | 1 | 33.3 | *Life satisfaction* | 1 | 25.0 |
| Mood | 20 | 19.8 |  |  |  | Mood | 1 | 25.0 |
| Mood | **20** | **19.8** |  | **1** | **33.3** |  | **1** | **25.0** |
| *Mood* | 4 | 4.0 | Negative affect | 1 | 33.3 | Positive affect | 1 | 25.0 |
| Positive affect | 17 | 16.8 |  |  |  |  |  |  |
| Negative affect | 7 | 6.9 |  |  |  |  |  |  |
| Positive Affect | **17** | **16.8** |  | **0** | **0** |  | **1** | **25.0** |
| *Positive affect* | 3 | 3.0 |  |  |  | *Happiness* | 1 | 25.0 |
| *Cheerfulness* | 1 | 1.0 |  |  |  |  |  |  |
| *Contentment* | 1 | 1.0 |  |  |  |  |  |  |
| *Enjoyment* | 3 | 3.0 |  |  |  |  |  |  |
| *Feel good* | 1 | 1.0 |  |  |  |  |  |  |
| *Good spirits* | 2 | 2.0 |  |  |  |  |  |  |
| *Happiness* | 5 | 5.0 |  |  |  |  |  |  |
| *Hope* | 1 | 1.0 |  |  |  |  |  |  |
| *Joy* | 2 | 2.0 |  |  |  |  |  |  |
| *Laughter* | 1 | 1.0 |  |  |  |  |  |  |
| *Pleasure* | 1 | 1.0 |  |  |  |  |  |  |
| *Relaxed/at ease* | 2 | 2.0 |  |  |  |  |  |  |
| Negative affect | **7** | **6.9** |  | **1** | **33.3** |  | **0** | **0** |
| *Negative affect* | 4 | 4.0 | *Fear* | 1 | 33.3 |  |  |  |
| *Agitation* | 1 | 1.0 | *Downhearted/blue* | 1 | 33.3 |  |  |  |
| *Downhearted/blue* | 1 | 1.0 | *Panic* | 1 | 33.3 |  |  |  |
| *Fear* | 1 | 1.0 | *Tearfulness* | 1 | 33.3 |  |  |  |
| *Panic* | 1 | 1.0 | *Upset* | 1 | 33.3 |  |  |  |
| *Pressure* | 1 | 1.0 |  |  |  |  |  |  |
| *Tearfulness* | 1 | 1.0 |  |  |  |  |  |  |
| *Tension* | 1 | 1.0 |  |  |  |  |  |  |
| *Upset* | 1 | 1.0 |  |  |  |  |  |  |
| *Worry* | 1 | 1.0 |  |  |  |  |  |  |
| Eudaimonia | **8** | **7.9** |  | **0** | **0** |  | **0** | **0** |
| *Eudaimonia* | 2 | 2.0 |  |  |  |  |  |  |
| *Autonomy* | 2 | 2.0 |  |  |  |  |  |  |
| *Flourishing* | 1 | 1.0 |  |  |  |  |  |  |
| *Goals* | 1 | 1.0 |  |  |  |  |  |  |
| *Personal development* | 1 | 1.0 |  |  |  |  |  |  |
| *Self-acceptance* | 3 | 3.0 |  |  |  |  |  |  |
| *Self-realisation* | 2 | 2.0 |  |  |  |  |  |  |
| *Social relationships* | 4 | 4.0 |  |  |  |  |  |  |
| Mental Health | **24** | **23.8** |  | **2** | **66.7** |  | **0** | **0** |
| *Mental health* | 7 | 6.9 | *Anxiety* | 1 | 33.3 |  |  |  |
| *Anxiety* | 5 | 5.0 | *Depression* | 2 | 66.7 |  |  |  |
| *Crying episodes on seeing effects of comorbid condition* | 1 | 1.0 |  |  |  |  |  |  |
| *Depression* | 18 | 17.8 |  |  |  |  |  |  |
| *Despair* | 1 | 1.0 |  |  |  |  |  |  |
| *Distress* | 3 | 3.0 |  |  |  |  |  |  |
| *Psychological state* | 1 | 1.0 |  |  |  |  |  |  |
| *Stress* | 1 | 1.0 |  |  |  |  |  |  |
| Self/identity | **18** | **17.8** |  | **0** | **0** |  | **2** | **50.0** |
| *Identity* | 1 | 1.0 |  |  |  | *Able to deal with problems/changes* | *1* | *25.0* |
| *Able to deal with problems/changes* | 1 | 1.0 |  |  |  | *Enthusiasm for life* | *1* | *25.0* |
| *Agency* | 1 | 1.0 |  |  |  | *Eager to tackle tasks* | *1* | *25.0* |
| *Attitude towards ageing* | 1 | 1.0 |  |  |  | *Vitality/energy* | *1* | *25.0* |
| *Attitude towards life* | 1 | 1.0 |  |  |  |  |  |  |
| *Behavioural-emotional control* | 1 | 1.0 |  |  |  |  |  |  |
| *Capability* | 1 | 1.0 |  |  |  |  |  |  |
| *Confidence* | 2 | 2.0 |  |  |  |  |  |  |
| *Control* | 2 | 2.0 |  |  |  |  |  |  |
| *Eager to tackle daily tasks* | 1 | 1.0 |  |  |  |  |  |  |
| *Future orientation* | 1 | 1.0 |  |  |  |  |  |  |
| *Interest in everyday matters* | 2 | 2.0 |  |  |  |  |  |  |
| *Morale* | 3 | 3.0 |  |  |  |  |  |  |
| *Optimism* | 2 | 2.0 |  |  |  |  |  |  |
| *Pessimism* | 1 | 1.0 |  |  |  |  |  |  |
| *Role* | 1 | 1.0 |  |  |  |  |  |  |
| *Role disruption* | 1 | 1.0 |  |  |  |  |  |  |
| *Security* | 2 | 2.0 |  |  |  |  |  |  |
| *Self-care* | 1 | 1.0 |  |  |  |  |  |  |
| *Self-confidence* | 1 | 1.0 |  |  |  |  |  |  |
| *Self-control* | 1 | 1.0 |  |  |  |  |  |  |
| *Self-efficacy* | 1 | 1.0 |  |  |  |  |  |  |
| *Self-esteem* | 2 | 2.0 |  |  |  |  |  |  |
| *Self-worth* | 1 | 1.0 |  |  |  |  |  |  |
| *Vitality/energy* | 5 | 5.0 |  |  |  |  |  |  |
| Psycho. reaction to disability | **6** | **5.9** |  | **0** | **0** |  | **0** | **0** |
| *Acceptance/Adaptation* | 1 | 1.0 |  |  |  |  |  |  |
| *Adjustment to vision loss* | 2 | 2.0 |  |  |  |  |  |  |
| *Attitude towards rehab* | 1 | 1.0 |  |  |  |  |  |  |
| *Coping* | 2 | 2.0 |  |  |  |  |  |  |
| *Feel accepted* | 1 | 1.0 |  |  |  |  |  |  |
| Health | **18** | **17.8** |  | **1** | **33.3** |  | **0** | **0** |
| *Contact with doctors* | 1 | 1.0 | *Disability* | 1 | 33.3 |  |  |  |
| *DALYs* | 5 | 5.0 | *Health status* | 1 | 33.3 |  |  |  |
| *Days in bed* | 1 | 1.0 |  |  |  |  |  |  |
| *Days of in-home service* | 1 | 1.0 |  |  |  |  |  |  |
| *Disability* | 1 | 1.0 |  |  |  |  |  |  |
| *Discomfort/pain* | 2 | 2.0 |  |  |  |  |  |  |
| *Health satisfaction* | 1 | 1.0 |  |  |  |  |  |  |
| *Health status* | 9 | 8.9 |  |  |  |  |  |  |
| *Morbidity burden* | 4 | 4.0 |  |  |  |  |  |  |
| *Mortality burden* | 5 | 5.0 |  |  |  |  |  |  |
| *Nights in hospital/nursing home* | 1 | 1.0 |  |  |  |  |  |  |
| *Physical health* | 3 | 3.0 |  |  |  |  |  |  |
| *Psychological health* | 1 | 1.0 |  |  |  |  |  |  |
| *QALYs* | 1 | 1.0 |  |  |  |  |  |  |
| *Satisfaction with surgery outcome* | 1 | 1.0 |  |  |  |  |  |  |
| *Satisfaction with visual health* | 1 | 1.0 |  |  |  |  |  |  |
| *Trouble with vision* | 1 | 1.0 |  |  |  |  |  |  |
| *Years of sight loss* | 1 | 1.0 |  |  |  |  |  |  |
| Functioning | **10** | **9.9** |  | **1** | **33.3** |  | **0** | **0** |
| *ADLs* | 4 | 4.0 | *Functional ability* | 1 | 33.3 |  |  |  |
| *Balance* | 1 | 1.0 | *Self-sufficiency* | 1 | 33.3 |  |  |  |
| *Capacity* | 1 | 1.0 |  |  |  |  |  |  |
| *Cognitive status* | 1 | 1.0 |  |  |  |  |  |  |
| *Falls* | 2 | 2.0 |  |  |  |  |  |  |
| *Feel safe/assured* | 2 | 2.0 |  |  |  |  |  |  |
| *Fulfilling responsibilities* | 1 | 1.0 |  |  |  |  |  |  |
| *Leisure activities* | 1 | 1.0 |  |  |  |  |  |  |
| *Managing better* | 1 | 1.0 |  |  |  |  |  |  |
| *Mobility* | 1 | 1.0 |  |  |  |  |  |  |
| *Personal care* | 1 | 1.0 |  |  |  |  |  |  |
| *Personal safety* | 1 | 1.0 |  |  |  |  |  |  |
| *Physical activity* | 2 | 2.0 |  |  |  |  |  |  |
| *Physical functioning* | 1 | 1.0 |  |  |  |  |  |  |
| *Usual activities* | 1 | 1.0 |  |  |  |  |  |  |
| Social functioning | **19** | **18.8** |  | **0** | **0** |  | **0** | **0** |
| *Social functioning* | 1 | 1.0 |  |  |  |  |  |  |
| *Attachment* | 1 | 1.0 |  |  |  |  |  |  |
| *Feeling of belonging* | 1 | 1.0 |  |  |  |  |  |  |
| *Positive outlook toward social relationships* | 1 | 1.0 |  |  |  |  |  |  |
| *Satisfaction with social activities* | 1 | 1.0 |  |  |  |  |  |  |
| *Social activity* | 2 | 2.0 |  |  |  |  |  |  |
| *Social engagement* | 2 | 2.0 |  |  |  |  |  |  |
| *Social integration* | 1 | 1.0 |  |  |  |  |  |  |
| *Social interaction* | 3 | 3.0 |  |  |  |  |  |  |
| *Loneliness* | 5 | 5.0 |  |  |  |  |  |  |
| *Social isolation* | 3 | 3.0 |  |  |  |  |  |  |
| *Social participation* | 2 | 2.0 |  |  |  |  |  |  |
| *Social relationships* | 4 | 4.0 |  |  |  |  |  |  |
| Environment | **6** | **5.9** |  | **0** | **0** |  | **0** | **0** |
| *Assets* | 1 | 1.0 |  |  |  |  |  |  |
| *Broader life circumstances* | 1 | 1.0 |  |  |  |  |  |  |
| *Economic (viability)* | 1 | 1.0 |  |  |  |  |  |  |
| *Food security* | 1 | 1.0 |  |  |  |  |  |  |
| *Income* | 3 | 3.0 |  |  |  |  |  |  |
| *Life space* | 1 | 1.0 |  |  |  |  |  |  |
| Other types of well-being | **6** | **5.9** |  | **0** | **0** |  | **0** | **0** |
| *Emotional well-being* | 2 | 2.0 |  |  |  |  |  |  |
| *Personal well-being* | 2 | 2.0 |  |  |  |  |  |  |
| *Physical well-being* | 1 | 1.0 |  |  |  |  |  |  |
| *Positive well-being* | 1 | 1.0 |  |  |  |  |  |  |
| *Psychological well-being* | 1 | 1.0 |  |  |  |  |  |  |
| *Social well-being* | 1 | 1.0 |  |  |  |  |  |  |
| QoL | **18** | **17.8** |  | **0** | **0** |  | **0** | **0** |
| *QoL* | 9 | 8.9 |  |  |  |  |  |  |
| *Component of QoL* | 9 | 8.9 |  |  |  |  |  |  |
| Other | **1** | **1.0** |  | **0** | **0** |  | **0** | **0** |
| *Dwelling (feel at home with what’s been given)* | 1 | 1.0 |  |  |  |  |  |  |
| *Mobility (feel able to explore possibilities)* | 1 | 1.0 |  |  |  |  |  |  |
| *Embodiment* | 1 | 1.0 |  |  |  |  |  |  |
| *Intersubjectivity* | 1 | 1.0 |  |  |  |  |  |  |
| *Spatiality* | 1 | 1.0 |  |  |  |  |  |  |
| *Temporality* | 1 | 1.0 |  |  |  |  |  |  |
| Not identified/clear | **46** | **45.5** |  | **0** | **0** |  | **2** | **50.0** |
