# Supplementary material for: Conceptualizations of well-being in adults with visual impairment: A scoping review
Source: Front Psychol. 2022 Sep 26;13:964537. doi: 10.3389/fpsyg.2022.964537 (PMC9549791; doi:10.3389/fpsyg.2022.964537)
Supplement: Supplementary file 3 [file Table_3.doc]

Supplementary Table 3 - Overview of domains and indicators of subjective and personal well-being

| Subjective well-being (*n*=38) | | | Personal well-being (*n*=4) | | |
| --- | --- | --- | --- | --- | --- |
|  | ***n*** | **%** |  | ***n*** | **%** |
| Hedonia | **21** | **55.3** |  | **1** | **25.0** |
| *Hedonia* | 1 | 2.6 | Mood | 1 | 25.0 |
| *Life satisfaction* | 17 | 44.7 |  |  |  |
| Mood | 12 | 31.6 |  |  |  |
| Mood | **12** | **31.6** |  | **1** | **25.0** |
| *Mood* | 2 | 5.3 | *Mood* | 1 | 25.0 |
| *Affect balance* | 2 | 5.3 | *Affect* | 1 | 25.0 |
| *Feelings* | 1 | 2.6 |  |  |  |
| Positive affect | 11 | 28.9 |  |  |  |
| Negative affect | 7 | 18.4 |  |  |  |
| Positive affect | **11** | **28.9** |  | **0** | **0** |
| *Positive affect/emotions* | 5 | 13.2 |  |  |  |
| *Cheerful* | 2 | 5.3 |  |  |  |
| *Content* | 3 | 7.9 |  |  |  |
| *Full of life* | 1 | 2.6 |  |  |  |
| *Happiness* | 5 | 13.2 |  |  |  |
| *In good spirits* | 1 | 2.6 |  |  |  |
| *Nonagitation* | 1 | 2.6 |  |  |  |
| *Relaxed* | 1 | 2.6 |  |  |  |
| Negative affect | **7** | **18.4** |  | **0** | **0** |
| *Negative affect* | 6 | 15.8 |  |  |  |
| *Boredom* | 1 | 2.6 |  |  |  |
| *Sadness* | 1 | 2.6 |  |  |  |
| *Upset* | 1 | 2.6 |  |  |  |
| Eudaimonia | **3** | **7.9** |  | **0** | **0** |
| *Eudaimonia* | 1 | 2.6 |  |  |  |
| *Achievements* | 1 | 2.6 |  |  |  |
| *Environmental mastery* | 1 | 2.6 |  |  |  |
| *Flourishing* | 1 | 2.6 |  |  |  |
| *Goals* | 1 | 2.6 |  |  |  |
| *Purpose in life* | 1 | 2.6 |  |  |  |
| *Self-acceptance* | 2 | 5.3 |  |  |  |
| *Self-realisation* | 1 | 2.6 |  |  |  |
| *Social relationships* | 1 | 2.6 |  |  |  |
| Mental Health | **9** | **23.7** |  | **0** | **0** |
| *Mental health* | 2 | 5.3 |  |  |  |
| *Depression* | 5 | 13.2 |  |  |  |
| *Distress* | 1 | 2.6 |  |  |  |
| *Stress* | 2 | 5.3 |  |  |  |
| Self/identity | **11** | **28.9** |  | **0** | **0** |
| *Able to make up own mind* | 1 | 2.6 |  |  |  |
| *Able to think clearly* | 1 | 2.6 |  |  |  |
| *Concerns* | 1 | 2.6 |  |  |  |
| *Expectations* | 1 | 2.6 |  |  |  |
| *Feelings about oneself* | 1 | 2.6 |  |  |  |
| *Feelings about the future* | 1 | 2.6 |  |  |  |
| *Feel useful* | 1 | 2.6 |  |  |  |
| *Future orientation* | 2 | 5.3 |  |  |  |
| *Interest in everyday matters* | 1 | 2.6 |  |  |  |
| *Morale* | 2 | 5.3 |  |  |  |
| *Optimism* | 3 | 7.9 |  |  |  |
| *Outlook on life* | 1 | 2.6 |  |  |  |
| *Pessimism* | 2 | 5.3 |  |  |  |
| *Priorities* | 1 | 2.6 |  |  |  |
| *Role difficulties* | 1 | 2.6 |  |  |  |
| *Self-assurance* | 1 | 2.6 |  |  |  |
| *Self-confidence* | 2 | 5.3 |  |  |  |
| *Self-esteem* | 1 | 2.6 |  |  |  |
| *Self-worth* | 1 | 2.6 |  |  |  |
| *Trait anxiety* | 1 | 2.6 |  |  |  |
| *Values* | 1 | 2.6 |  |  |  |
| *Vitality/energy* | 1 | 2.6 |  |  |  |
| Health | **6** | **15.8** |  | **0** | **0** |
| *Discomfort (pain)* | 1 | 2.6 |  |  |  |
| *Health satisfaction* | 1 | 2.6 |  |  |  |
| *General health* | 1 | 2.6 |  |  |  |
| *Medical care* | 2 | 5.3 |  |  |  |
| *Sleep* | 1 | 2.6 |  |  |  |
| Functioning | **3** | **7.9** |  | **1** | **25.0** |
| *Ability to perform expected tasks* | 1 | 2.6 | *Leisure activities* | 1 | 25.0 |
| *Dealing with problems well* | 1 | 2.6 | *Personal care* | 1 | 25.0 |
| *Dependency* | 1 | 2.6 | *Personal safety* | 1 | 25.0 |
| *Leisure time satisfaction* | 1 | 2.6 |  |  |  |
| Social functioning | **5** | **13.2** |  | **0** | **0** |
| *Social functioning* | 1 | 2.6 |  |  |  |
| *Ability to participate in society* | 1 | 2.6 |  |  |  |
| *Family and social relationships* | 1 | 2.6 |  |  |  |
| *Feeling close to others* | 1 | 2.6 |  |  |  |
| *Loneliness* | 1 | 2.6 |  |  |  |
| *Social relationships* | 1 | 2.6 |  |  |  |
| Environment | **1** | **2.6** |  | **0** | **0** |
| *Income satisfaction* | 1 | 2.6 |  |  |  |
| *Job satisfaction* | 1 | 2.6 |  |  |  |
| Other types of well-being | **4** | **10.5** |  | **0** | **0** |
| *Financial well-being* | 2 | 5.3 |  |  |  |
| *General well-being* | 1 | 2.6 |  |  |  |
| *Mental well-being* | 1 | 2.6 |  |  |  |
| *Physical well-being* | 2 | 5.3 |  |  |  |
| *Psychological well-being* | 3 | 7.9 |  |  |  |
| *Social and family well-being* | 2 | 5.3 |  |  |  |
| QoL | **7** | **18.4** |  | **1** | **25.0** |
| *QoL* | 2 | 5.3 | *Component of QoL* | 1 | 25.0 |
| *Component of QoL* | 5 | 13.2 |  |  |  |
| Other | **3** | **7.9** |  | **0** | **0** |
| *Adjustment to living in a foreign country* | 1 | 2.6 |  |  |  |
| *Satisfaction with ageing process* | 1 | 2.6 |  |  |  |
| *Spiritual life* | 1 | 2.6 |  |  |  |
| *Thoughts* | 1 | 2.6 |  |  |  |
| Not identified/clear | **10** | **26.3** |  | **2** | **50.0** |
