# Supplementary material for: Conceptualizations of well-being in adults with visual impairment: A scoping review
Source: Front Psychol. 2022 Sep 26;13:964537. doi: 10.3389/fpsyg.2022.964537 (PMC9549791; doi:10.3389/fpsyg.2022.964537)
Supplement: Supplementary file 4 [file Table_4.doc]

Supplementary Table 4 - Overview of domains and indicators of psychological, mental and psychosocial well-being

| Psychological well-being (*n*=66) | | | Mental well-being (n=28) | | | Psychosocial well-being (n=18) | | |
| --- | --- | --- | --- | --- | --- | --- | --- | --- |
|  | ***n*** | **%** |  | ***n*** | **%** |  | ***n*** | **%** |
| Hedonia | **21** | **31.8** |  | **6** | **21.4** |  | **6** | **33.3** |
| *Life satisfaction* | 7 | 10.6 | *Life satisfaction* | 1 | 3.6 | *Life satisfaction* | 1 | 5.6 |
| Mood | 17 | 25.8 | Mood | 5 | 17.9 | Mood | 5 | 27.8 |
| Mood | **17** | **25.8** |  | **5** | **17.9** |  | **5** | **27.8** |
| *Mood* | 6 | 9.1 | *Mood* | 1 | 3.6 | Positive affect | 1 | 5.6 |
| Positive affect | 7 | 10.6 | *Feelings* | 2 | 7.1 | Negative affect | 5 | 27.8 |
| Negative affect | 10 | 15.2 | Positive affect | 1 | 3.6 |  |  |  |
|  |  |  | Negative affect | 2 | 7.1 |  |  |  |
| Positive affect | **7** | **10.6** |  | **1** | **3.6** |  | **1** | **5.6** |
| *Positive Affect* | 3 | 4.5 | *Feel calm/peaceful* | 1 | 3.6 | *Happiness* | 1 | 5.6 |
| *Alertness* | 1 | 1.5 |  |  |  |  |  |  |
| *Enjoyment* | 1 | 1.5 |  |  |  |  |  |  |
| *Excitement* | 1 | 1.5 |  |  |  |  |  |  |
| *Happiness* | 1 | 1.5 |  |  |  |  |  |  |
| *Interest* | 1 | 1.5 |  |  |  |  |  |  |
| *Joy* | 1 | 1.5 |  |  |  |  |  |  |
| *Pleasantness (hedonistic tone)* | 1 | 1.5 |  |  |  |  |  |  |
| *Relaxation (calmness-tension)* | 2 | 3.0 |  |  |  |  |  |  |
| Negative affect | **10** | **15.2** |  | **2** | **7.1** |  | **5** | **27.8** |
| *Negative affect* | 3 | 4.5 | *Negative affect* | 1 | 3.6 | *Negative emotions* | 1 | 5.6 |
| *Anger* | 2 | 3.0 | *Anger* | 1 | 3.6 | *Anger/Annoyance* | 2 | 11.1 |
| *Contempt* | 1 | 1.5 | *Downhearted/blue* | 1 | 3.6 | *Fear (of further sight loss)* | 3 | 16.7 |
| *Disgust* | 1 | 1.5 | *Feel low* | 1 | 3.6 | *Feel like a burden* | 1 | 5.6 |
| *Dissatisfaction* | 1 | 1.5 | *Frustration* | 1 | 3.6 | *Frustration* | 3 | 16.7 |
| *Downhearted/blue* | 2 | 3.0 | *Irritation* | 1 | 3.6 | *Sadness* | 1 | 5.6 |
| *Fear* | 4 | 6.1 | *Shock* | 1 | 3.6 | *Upset* | 1 | 5.6 |
| *Frustration* | 2 | 3.0 |  |  |  | *Worry* | 3 | 16.7 |
| *Guilt* | 1 | 1.5 |  |  |  |  |  |  |
| *Hopelessness* | 1 | 1.5 |  |  |  |  |  |  |
| *Hostility* | 1 | 1.5 |  |  |  |  |  |  |
| *Panic* | 1 | 1.5 |  |  |  |  |  |  |
| *Sadness* | 1 | 1.5 |  |  |  |  |  |  |
| *Tearfulness* | 1 | 1.5 |  |  |  |  |  |  |
| *Upset* | 1 | 1.5 |  |  |  |  |  |  |
| *Worry* | 1 | 1.5 |  |  |  |  |  |  |
| Eudaimonia | **9** | **13.6** |  | **0** | **0** |  | **2** | **11.1** |
| *Autonomy* | 4 | 6.1 |  |  |  | *Autonomy* | 1 | 5.6 |
| *Career goals* | 1 | 1.5 |  |  |  | *Interpersonal relationships* | 1 | 5.6 |
| *Environmental mastery* | 3 | 4.5 |  |  |  |  |  |  |
| *Goal attainment* | 1 | 1.5 |  |  |  |  |  |  |
| *Harmony* | 1 | 1.5 |  |  |  |  |  |  |
| *Personal growth* | 3 | 4.5 |  |  |  |  |  |  |
| *Purpose in life* | 3 | 4.5 |  |  |  |  |  |  |
| *Self-acceptance* | 5 | 7.6 |  |  |  |  |  |  |
| *Social/close relationships* | 5 | 7.6 |  |  |  |  |  |  |
| Mental health | **35** | **53.0** |  | **13** | **46.4** |  | **9** | **50.0** |
| *Mental Health* | 8 | 12.1 | *Mental Health* | 9 | 32.1 | *Mental health* | 5 | 27.8 |
| *Anxiety* | 19 | 28.8 | *Alcohol misuse* | 2 | 7.1 | *Anxiety* | 5 | 27.8 |
| *Depression* | 28 | 42.4 | *Anxiety* | 6 | 21.4 | *Depression* | 8 | 44.4 |
| *Distress* | 7 | 10.6 | *Bereavement* | 1 | 3.6 | *Distress* | 1 | 5.6 |
| *Obsessive/compulsive* | 1 | 1.5 | *Depression* | 8 | 28.6 | *Social anxiety* | 1 | 5.6 |
| *Paranoid ideation* | 1 | 1.5 | *Distress* | 1 | 3.6 | *Stress* | 1 | 5.6 |
| *Phobic anxiety* | 1 | 1.5 | *Nervous breakdowns* | 1 | 3.6 |  |  |  |
| *Psychoticism* | 1 | 1.5 | *PTSD* | 2 | 7.1 |  |  |  |
| *Risk to self/others* | 2 | 3.0 | *Suicide attempts* | 1 | 3.6 |  |  |  |
| *Somatisation (bodily dysfunction)* | 1 | 1.5 | *Suicidal thoughts* | 1 | 3.6 |  |  |  |
| *Stress* | 2 | 3.0 |  |  |  |  |  |  |
| *Suicidal history* | 1 | 1.5 |  |  |  |  |  |  |
| *Trauma* | 1 | 1.5 |  |  |  |  |  |  |
| Self/identity | **17** | **25.8** |  | **4** | **14.3** |  | **8** | **44.4** |
| *Able to deal with problems/change* | 1 | 1.5 | *Identity* | 1 | 3.6 | *Change personal items* | 1 | 5.6 |
| *Attribution style* | 1 | 1.5 | *Able to make up own mind* | 1 | 3.6 | *Confidence* | 1 | 5.6 |
| *Confidence* | 3 | 4.5 | *Able to think clearly* | 1 | 3.6 | *Control* | 1 | 5.6 |
| *Control* | 3 | 4.5 | *Feel useful* | 1 | 3.6 | *Empowerment* | 1 | 5.6 |
| *Eager to tackle daily tasks* | 1 | 1.5 | *Optimism* | 1 | 3.6 | *Motivated to try new things* | 1 | 5.6 |
| *Enthusiasm for life* | 1 | 1.5 | *Self-esteem* | 2 | 7.1 | *Role disruption* | 2 | 11.1 |
| *Extroversion-introversion* | 1 | 1.5 | *Self-worth* | 1 | 3.6 | *Self-confidence* | 1 | 5.6 |
| *Feelings about oneself* | 1 | 1.5 | *Vitality/energy* | 1 | 3.6 | *Self-esteem* | 2 | 11.1 |
| *Feelings about the future* | 1 | 1.5 |  |  |  | *Self-worth* | 2 | 11.1 |
| *Motivated to try new things* | 1 | 1.5 |  |  |  | *Vitality/energy* | 1 | 5.6 |
| *Outlook on life* | 1 | 1.5 |  |  |  |  |  |  |
| *Role disruption* | 1 | 1.5 |  |  |  |  |  |  |
| *Self-concept* | 1 | 1.5 |  |  |  |  |  |  |
| *Self-control* | 1 | 1.5 |  |  |  |  |  |  |
| *Self-efficacy* | 3 | 4.5 |  |  |  |  |  |  |
| *Self-esteem* | 6 | 9.1 |  |  |  |  |  |  |
| *Self-worth* | 1 | 1.5 |  |  |  |  |  |  |
| *Vitality/energy* | 4 | 6.1 |  |  |  |  |  |  |
| Psych. reaction to disability | **5** | **7.6** |  | **0** | **0** |  | **2** | **11.1** |
| *Psychological reaction to VI* | 1 | 1.5 |  |  |  | *Acceptance of vision loss* | 1 | 5.6 |
| *Acceptance of disability* | 3 | 4.5 |  |  |  | *Adaptation* | 1 | 5.6 |
| *Activities, beliefs, expectations, satisfaction with visual function* | 1 | 1.5 |  |  |  | *Adjustment* | 2 | 11.1 |
| *Adaptation to disability* | 2 | 3.0 |  |  |  |  |  |  |
| *Adjustment* | 1 | 1.5 |  |  |  |  |  |  |
| *Attitudes towards vision loss* | 1 | 1.5 |  |  |  |  |  |  |
| *Coping* | 1 | 1.5 |  |  |  |  |  |  |
| Health | **4** | **6.1** |  | **0** | **0** |  | **1** | **5.6** |
| *General health* | 1 | 1.5 |  |  |  | *Complex visual hallucinations* | 1 | 5.6 |
| *Physical health* | 1 | 1.5 |  |  |  |  |  |  |
| *Problems/symptoms* | 2 | 3.0 |  |  |  |  |  |  |
| *Psychological health* | 1 | 1.5 |  |  |  |  |  |  |
| Functioning | **5** | **7.6** |  | **3** | **10.7** |  | **4** | **22.2** |
| *Functioning* | 2 | 3.0 | *Functioning* | 1 | 3.6 | *Concern about safety at home* | 1 | 5.6 |
| *Day-to-day functioning* | 1 | 1.5 | *Deal with problems well* | 1 | 3.6 | *Independence* | 2 | 11.1 |
| *Dependence* | 1 | 1.5 | *Independence* | 1 | 3.6 | *Limitations on leaving home* | 1 | 5.6 |
| *Limitations on leaving home* | 1 | 1.5 | *Reliance on others* | 1 | 3.6 | *Reliance on others* | 2 | 11.1 |
| *Perceived security in performing daily occupations* | 1 | 1.5 |  |  |  |  |  |  |
| *Reliance on others* | 1 | 1.5 |  |  |  |  |  |  |
| Social functioning | **11** | **16.7** |  | **3** | **10.7** |  | **8** | **44.4** |
| *Social functioning* | 1 | 1.5 | *Communication* | 1 | 3.6 | *Social functioning* | 3 | 16.7 |
| *Close relationships* | 1 | 1.5 | *Feel close to others* | 1 | 3.6 | *Family relations* | 1 | 5.6 |
| *Interdependence with close others* | 1 | 1.5 | *Interpersonal relationships* | 1 | 3.6 | *Interpersonal relationships* | 1 | 5.6 |
| *Interpersonal sensitivity* | 1 | 1.5 | *Social/leisure activity* | 1 | 3.6 | *Loneliness* | 3 | 16.7 |
| *Loneliness* | 1 | 1.5 | *Social withdrawal* | 1 | 3.6 | *Social isolation* | 1 | 5.6 |
| *Social isolation* | 1 | 1.5 |  |  |  | *Social interaction* | 1 | 5.6 |
| *Respect from others* | 1 | 1.5 |  |  |  | *Social reaction* | 1 | 5.6 |
| *Social orientation* | 1 | 1.5 |  |  |  | *Social support* | 3 | 16.7 |
| *Social participation* | 1 | 1.5 |  |  |  | *Visits with family and friends* | 1 | 5.6 |
| *Social relationships* | 5 | 7.6 |  |  |  |  |  |  |
| Other types of well-being | **8** | **12.1** |  | **0** | **0** |  | **2** | **11.1** |
| *Emotional well-being* | 2 | 3.0 |  |  |  | *General well-being* | 1 | 5.6 |
| *Mental well-being* | 1 | 1.5 |  |  |  | *Psychological well-being* | 1 | 5.6 |
| *Negative well-being* | 1 | 1.5 |  |  |  | *Social well-being* | 1 | 5.6 |
| *Positive well-being* | 2 | 3.0 |  |  |  |  |  |  |
| *Subjective well-being* | 3 | 4.5 |  |  |  |  |  |  |
| QoL | **15** | **22.7** |  | **6** | **21.4** |  | **7** | **38.9** |
| *QoL* | 8 | 12.1 | *QoL* | 2 | 7.1 | *QoL* | 4 | 22.2 |
| *Component of QoL* | 8 | 12.1 | *Component of QoL* | 4 | 14.3 | *Component of QoL* | 3 | 16.7 |
| Other | **2** | **3.0** |  | **1** | **3.6** |  |  |  |
| *Psychological wellness* | 1 | 1.5 | *Thoughts* | 1 | 3.6 |  |  |  |
| *Activity* | 1 | 1.5 |  |  |  |  |  |  |
| Not identified/clear | **16** | **24.2** |  | **13** | **46.4** |  | **7** | **38.9** |
