# Supplementary material for: Conceptualizations of well-being in adults with visual impairment: A scoping review
Source: Front Psychol. 2022 Sep 26;13:964537. doi: 10.3389/fpsyg.2022.964537 (PMC9549791; doi:10.3389/fpsyg.2022.964537)
Supplement: Supplementary file 5 [file Table_5.doc]

Supplementary Table 5 - Overview of domains and indicators of emotional and affective well-being

| Emotional well-being (*n*=86) | | | Affective well-being (*n*=6) | | |
| --- | --- | --- | --- | --- | --- |
|  | ***n*** | **%** |  | ***n*** | **%** |
| Hedonia | **31** | **36.0** |  | **6** | **100.0** |
| *Life satisfaction* | 2 | 2.3 | Mood | 6 | 100.0 |
| Mood | 30 | 34.9 |  |  |  |
| Mood | **30** | **34.9** |  | **6** | **100.0** |
| *Mood* | 2 | 2.3 | Positive affect | 6 | 100.0 |
| *Emotions, emotional states/reactions* | 3 | 3.5 | Negative affect | 5 | 83.3 |
| *Emotional instability* | 1 | 1.2 |  |  |  |
| Positive affect | 10 | 11.6 |  |  |  |
| Negative affect | 27 | 31.4 |  |  |  |
| Positive affect | **10** | **11.6** |  | **6** | **100.0** |
| *Positive affect* | 1 | 1.2 | *Positive affect* | 5 | 83.3 |
| *Enjoyment* | 1 | 1.2 | *Happiness* | 1 | 16.7 |
| *Happiness* | 6 | 7.0 |  |  |  |
| *Hopefulness* | 3 | 3.5 |  |  |  |
| *Peacefulness* | 1 | 1.2 |  |  |  |
| Negative affect | **27** | **31.4** |  | **5** | **83.3** |
| *Negative affect* | 1 | 1.2 | *Negative affect* | 5 | 83.3 |
| *Anger* | 6 | 7.0 |  |  |  |
| *Annoyance* | 10 | 11.6 |  |  |  |
| *Awkwardness* | 1 | 1.2 |  |  |  |
| *Concern* | 1 | 1.2 |  |  |  |
| *Despair* | 1 | 1.2 |  |  |  |
| *Embarrassment* | 12 | 14.0 |  |  |  |
| *Fear* | 6 | 7.0 |  |  |  |
| *Feel like a burden* | 12 | 14.0 |  |  |  |
| *Feel low* | 8 | 9.3 |  |  |  |
| *Feel powerless* | 1 | 1.2 |  |  |  |
| *Feel worthless* | 1 | 1.2 |  |  |  |
| *Frustration* | 21 | 24.4 |  |  |  |
| *Grief* | 1 | 1.2 |  |  |  |
| *Guilt* | 1 | 1.2 |  |  |  |
| *Helplessness* | 3 | 3.5 |  |  |  |
| *Hostility* | 2 | 2.3 |  |  |  |
| *Irritation* | 1 | 1.2 |  |  |  |
| *Miss doing things you used to do* | 1 | 1.2 |  |  |  |
| *Panic* | 1 | 1.2 |  |  |  |
| *Regrets* | 1 | 1.2 |  |  |  |
| *Sadness* | 14 | 16.3 |  |  |  |
| *Shock* | 3 | 3.5 |  |  |  |
| *Uncertainty* | 1 | 1.2 |  |  |  |
| *Upset* | 2 | 2.3 |  |  |  |
| *Vulnerability* | 3 | 3.5 |  |  |  |
| *Worry (about eyesight)* | 13 | 15.1 |  |  |  |
| Mental Health | **24** | **27.9** |  | **1** | **16.7** |
| *Mental Health* | 7 | 8.1 | *Depression* | 1 | 16.7 |
| *Anxiety* | 11 | 12.8 |  |  |  |
| *Depression* | 17 | 19.8 |  |  |  |
| *Distress* | 6 | 7.0 |  |  |  |
| *Obsessive/compulsive* | 1 | 1.2 |  |  |  |
| *Paranoid ideation (hostility, suspiciousness, fear of loss of autonomy)* | 1 | 1.2 |  |  |  |
| *Phobic anxiety* | 2 | 2.3 |  |  |  |
| *Psychiatric symptomology* | 1 | 1.2 |  |  |  |
| *Psychoticism* | 1 | 1.2 |  |  |  |
| *Self-harm* | 1 | 1.2 |  |  |  |
| *Somatisation (bodily dysfunction)* | 2 | 2.3 |  |  |  |
| *Stress* | 2 | 2.3 |  |  |  |
| *Suicidal thoughts* | 2 | 2.3 |  |  |  |
| *Trauma* | 1 | 1.2 |  |  |  |
| Self/identity | **10** | **11.6** |  | **0** | **0** |
| *Attitude to life* | 1 | 1.2 |  |  |  |
| *Confidence* | 5 | 5.8 |  |  |  |
| *Empowerment* | 2 | 2.3 |  |  |  |
| *Optimism* | 3 | 3.5 |  |  |  |
| *Resilience* | 2 | 2.3 |  |  |  |
| *Role disruption* | 1 | 1.2 |  |  |  |
| *Self-control* | 1 | 1.2 |  |  |  |
| *Self-doubt* | 1 | 1.2 |  |  |  |
| *Self-esteem* | 4 | 4.7 |  |  |  |
| *Self-perception* | 1 | 1.2 |  |  |  |
| Psychological reaction to disability | **15** | **17.4** |  | **0** | **0** |
| *Adjustment* | 1 | 1.2 |  |  |  |
| *Coping* | 12 | 14.0 |  |  |  |
| *Demoralisation* | 1 | 1.2 |  |  |  |
| *Feeling inferior* | 1 | 1.2 |  |  |  |
| *Gratitude/Recognition of remaining capabilities* | 1 | 1.2 |  |  |  |
| *Worry about safety* | 1 | 1.2 |  |  |  |
| Health | **2** | **2.3** |  | **0** | **0** |
| *Fatigue* | 1 | 1.2 |  |  |  |
| *Physical symptoms (headaches, nausea, dizziness, insomnia, and appetite loss)* | 1 | 1.2 |  |  |  |
| Functioning | **11** | **12.8** |  | **0** | **0** |
| *Ability to work* | 1 | 1.2 |  |  |  |
| *Eyesight/health/emotions interfering with life* | 8 | 9.3 |  |  |  |
| *Independence* | 1 | 1.2 |  |  |  |
| *Psychosocial functioning* | 1 | 1.2 |  |  |  |
| *Stop doing things you want to do* | 1 | 1.2 |  |  |  |
| Social functioning | **17** | **19.8** |  | **0** | **0** |
| *Social functioning* | 2 | 2.3 |  |  |  |
| *Exclusion* | 1 | 1.2 |  |  |  |
| *Interpersonal sensitivity* | 1 | 1.2 |  |  |  |
| *Loneliness* | 14 | 16.3 |  |  |  |
| *Social isolation* | 11 | 12.8 |  |  |  |
| *Social activity* | 1 | 1.2 |  |  |  |
| *Social contact* | 1 | 1.2 |  |  |  |
| *Social participation (e.g. leaving the house)* | 1 | 1.2 |  |  |  |
| *Social support* | 2 | 2.3 |  |  |  |
| QoL | **38** | **44.2** |  | **0** | **0** |
| *QoL* | 3 | 3.5 |  |  |  |
| *Component of QoL* | 35 | 40.7 |  |  |  |
| Other | **1** | **1.2** |  | **0** | **0** |
| *Thoughts* | 1 | 1.2 |  |  |  |
| Not identified/clear | **43** | **50.0** |  | **0** | **0** |
