# Supplementary material for: Conceptualizations of well-being in adults with visual impairment: A scoping review
Source: Front Psychol. 2022 Sep 26;13:964537. doi: 10.3389/fpsyg.2022.964537 (PMC9549791; doi:10.3389/fpsyg.2022.964537)
Supplement: Supplementary file 6 [file Table_6.doc]

Supplementary Table 6 - Overview of domains and indicators for social and socio-emotional well-being

| Social well-being (*n*=29) | | | Socio-emotional well-being (*n*=3) | | |
| --- | --- | --- | --- | --- | --- |
|  | ***n*** | **%** |  | ***n*** | **%** |
| Hedonia | **0** | **0** |  | **1** | **33.3** |
|  |  |  | Mood | 1 | 33.3 |
| Mood | **0** | 0 |  | **1** | **33.3** |
|  |  |  | Negative affect | 1 | 33.3 |
| Negative affect | **0** | 0 |  | **1** | **33.3** |
|  |  |  | *Anger* | 1 | 33.3 |
|  |  |  | *Frustration* | 1 | 33.3 |
|  |  |  | *Miss doing things you used to do* | 1 | 33.3 |
|  |  |  | *Sadness* | 1 | 33.3 |
|  |  |  | *Worry (about eyesight)* | 1 | 33.3 |
| Eudaimonia | **6** | **20.7** |  | **0** | **0** |
| *Career goals* | 1 | 3.4 |  |  |  |
| *Social relationships* | 5 | 17.2 |  |  |  |
| Mental Health | **0** | **0** |  | **1** | **33.3** |
|  |  |  | *Socio-emotional distress* | 1 | 33.3 |
| Self /identity | **3** | **10.3** |  | **1** | **33.3** |
| *Altruism (wanting to help others)* | 1 | 3.4 | *Confidence* | 1 | 33.3 |
| *Confidence* | 1 | 3.4 | *Role disruption* | 1 | 33.3 |
| *Connection with natural environment* | 1 | 3.4 |  |  |  |
| *Role disruption* | 1 | 3.4 |  |  |  |
| Psychological reaction to disability | **1** | **3.4** |  | **0** | **0** |
| *Concern about treatment by others* | 1 | 3.4 |  |  |  |
| Functioning | **2** | **6.9** |  | **1** | **33.3** |
| *Dependence* | 1 | 3.4 | *Ability to maintain responsibilities* | 1 | 33.3 |
| *Fulfilling responsibilities* | 1 | 3.4 |  |  |  |
| Social functioning | **13** | **44.8** |  | **1** | **33.3** |
| *Social functioning* | 2 | 6.9 | Social participation | 1 | 33.3 |
| Family functioning | 2 | 6.9 |  |  |  |
| Intimate relationships | 2 | 6.9 |  |  |  |
| Social isolation | 4 | 13.8 |  |  |  |
| Social participation | 12 | 41.4 |  |  |  |
| Social relationships | 5 | 17.2 |  |  |  |
| Family functioning | **2** | **6.9** |  | **0** | **0** |
| *Family life* | 1 | 3.4 |  |  |  |
| *Being a parent* | 1 | 3.4 |  |  |  |
| *Communication* | 1 | 3.4 |  |  |  |
| *Family functioning* | 1 | 3.4 |  |  |  |
| *Parental functioning* | 1 | 3.4 |  |  |  |
| *Problem solving* | 1 | 3.4 |  |  |  |
| *Satisfaction with relationship with children* | 1 | 3.4 |  |  |  |
| Intimate relationships | **2** | **6.9** |  | **0** | **0** |
| *Intimate relationships and roles* | 1 | 3.4 |  |  |  |
| *Relationship status* | 1 | 3.4 |  |  |  |
| *Relationship functioning* | 1 | 3.4 |  |  |  |
| *Relationship satisfaction* | 1 | 3.4 |  |  |  |
| Social relationships | **5** | **17.2** |  | **0** | **0** |
| *Social relationships* | 1 | 3.4 |  |  |  |
| *Interpersonal relationships* | 2 | 6.9 |  |  |  |
| *Ability to maintain friendships* | 1 | 3.4 |  |  |  |
| *Ability to make new friends* | 1 | 3.4 |  |  |  |
| *Interpersonal connectedness* | 1 | 3.4 |  |  |  |
| *Intimacy (physical/emotional)* | 1 | 3.4 |  |  |  |
| Social isolation | **4** | **13.8** |  | **0** | **0** |
| *Social isolation* | 4 | 13.8 |  |  |  |
| *Disengagement with community* | 1 | 3.4 |  |  |  |
| *Exclusion* | 1 | 3.4 |  |  |  |
| *Loneliness* | 2 | 6.9 |  |  |  |
| Social participation | **12** | **41.4** |  | **1** | **33.3** |
| *Social participation* | 3 | 10.3 | *Reduction in social life* | 1 | 33.3 |
| *Interacting with the world* | 1 | 3.4 |  |  |  |
| *Satisfaction with social involvement* | 1 | 3.4 |  |  |  |
| *Social activity* | 4 | 13.8 |  |  |  |
| *Social contact* | 2 | 6.9 |  |  |  |
| *Social interaction* | 9 | 31.0 |  |  |  |
| *Social support* | 2 | 6.9 |  |  |  |
| Environment | **2** | **6.9** |  | **0** | **0** |
| *Ability to maintain work* | 1 | 3.4 |  |  |  |
| *Financial strain/loss of income* | 1 | 3.4 |  |  |  |
| *Premature retirement* | 1 | 3.4 |  |  |  |
| *Unequal treatment by others* | 1 | 3.4 |  |  |  |
| *Work life* | 1 | 3.4 |  |  |  |
| QoL | **12** | **41.4** |  | **1** | **33.3** |
| *QoL* | 2 | 6.9 | *Component of QoL* | 1 | 33.3 |
| *Component of QoL* | 10 | 34.5 |  |  |  |
| Not identified/clear | **14** | **48.3** |  | **1** | **33.3** |
