# Supplementary material for: Conceptualizations of well-being in adults with visual impairment: A scoping review
Source: Front Psychol. 2022 Sep 26;13:964537. doi: 10.3389/fpsyg.2022.964537 (PMC9549791; doi:10.3389/fpsyg.2022.964537)
Supplement: Supplementary file 7 [file Table_7.doc]

**Supplementary Table 7 - Overview of domains and indicators of physical and physiological well-being**

| Physical well-being (*n*=24) | | | Physiological well-being (*n*=2) | | |
| --- | --- | --- | --- | --- | --- |
|  | ***n*** | **%** |  | ***n*** | **%** |
| Hedonia | **1** | **4.2** |  | **0** | **0** |
| Mood | 1 | 4.2 |  |  |  |
| Mood | **1** | **4.2** |  | **0** | **0** |
| *Mood* | 1 | 4.2 |  |  |  |
| Psychological reaction to disability | **1** | **4.2** |  | **0** | **0** |
| *Coping with health problems due to VI* | 1 | 4.2 |  |  |  |
| Health | **3** | **12.5** |  | **1** | **50.0** |
| *Comorbid health problem* | 1 | 4.2 | *Hearing* | 1 | 50.0 |
| *Comorbid physical disability* | 1 | 4.2 | *Lung function* | 1 | 50.0 |
| *Health status* | 2 | 8.3 | *Vision* | 1 | 50.0 |
| *Hearing* | 1 | 4.2 |  |  |  |
| Functioning | **7** | **29.2** |  | **2** | **100.0** |
| Physical functioning | 7 | 29.2 | Physical functioning | 2 | 100.0 |
| Activity functioning | 3 | 12.5 |  |  |  |
| Physical functioning | **7** | **29.2** |  | **2** | **100.0** |
| *Physical functioning* | 3 | 12.5 | *Balance* | 1 | 50.0 |
| *Awareness of surroundings* | 1 | 4.2 | *Falls* | 1 | 50.0 |
| *Balance* | 2 | 8.3 | *Grip strength* | 1 | 50.0 |
| *Coordination* | 1 | 4.2 | *Vibration sense* | 1 | 50.0 |
| *Endurance* | 1 | 4.2 |  |  |  |
| *Falls* | 2 | 8.3 |  |  |  |
| *Injury risk* | 1 | 4.2 |  |  |  |
| *Mobility* | 2 | 8.3 |  |  |  |
| *Stamina* | 1 | 4.2 |  |  |  |
| *Strength* | 1 | 4.2 |  |  |  |
| Activity functioning | **3** | **12.5** |  | **0** | **0** |
| *Ability to participate in activities* | 1 | 4.2 |  |  |  |
| *ADLs* | 1 | 4.2 |  |  |  |
| *Physical activity* | 1 | 4.2 |  |  |  |
| *Walking* | 1 | 4.2 |  |  |  |
| QoL | **6** | **25.0** |  | **0** | **0** |
| *Component of QoL* | 6 | 25.0 |  |  |  |
| Not identified/clear | **16** | **66.7** |  | **0** | **0** |
