# Supplementary material for: Conceptualizations of well-being in adults with visual impairment: A scoping review
Source: Front Psychol. 2022 Sep 26;13:964537. doi: 10.3389/fpsyg.2022.964537 (PMC9549791; doi:10.3389/fpsyg.2022.964537)
Supplement: Supplementary file 8 [file Table_8.doc]

**Supplementary Table 8 - Overview of indicators of other types of well-being**

|  | *n* | % |  | *n* | % |  | *n* | % |
| --- | --- | --- | --- | --- | --- | --- | --- | --- |
| Spiritual well-being | **3** |  | **Religious well-being** | **1** |  | **Existential well-being** | **1** |  |
| *Interpersonal connection* | 1 | 33.3 | *Relationship with a higher power* | 1 | 100.0 | *Intrapersonal wellness* | 1 | 100.0 |
| *Intrapersonal connection* | 1 | 33.3 | *Closeness with a higher power* | 1 | 100.0 | *Purpose* | 1 | 100.0 |
| *Intrapersonal meaning* | 1 | 33.3 |  |  |  | *Sense of meaning of life* | 1 | 100.0 |
| *Intrapersonal wellness* | 1 | 33.3 |  |  |  | *Fulfilment* | 1 | 100.0 |
| *Relationship to a higher power/transpersonal connection* | 2 | 66.7 |  |  |  |  |  |  |
| Not identified/clear | 1 | 33.3 |  |  |  |  |  |  |
| Economic well-being | **6** |  | **Financial well-being** | **5** |  | **Vocational well-being** | **1** |  |
| *Income* | 2 | 33.3 | *Income* | 1 | 20.0 | *Being in education* | 1 | 100.0 |
| *Asset ownership* | 1 | 16.7 | *Financial functioning* | 1 | 20.0 | *Employment status* | 1 | 100.0 |
| *Capacity for economic consumption* | 1 | 16.7 | *Financial security* | 1 | 20.0 | *Volunteering/unpaid work* | 1 | 100.0 |
| *Economic-related social activity* | 1 | 16.7 | *Satisfaction with finances* | 1 | 20.0 | *Work functioning* | 1 | 100.0 |
| *Housing tenure* | 1 | 16.7 | Not identified/clear | 3 | 60.0 | *Work satisfaction* | 1 | 100.0 |
| *Housing conditions* | 1 | 16.7 |  |  |  |  |  |  |
| *Labour market involvement* | 1 | 16.7 |  |  |  |  |  |  |
| *Wealth* | 1 | 16.7 |  |  |  |  |  |  |
| *Component of QoL* | 1 | 16.7 |  |  |  |  |  |  |
| Not identified/clear | 3 | 50.0 |  |  |  |  |  |  |
| Environmental well-being | **2** |  | **Socio-ecological well-being** | **1** |  | **Eudaimonic well-being** | **3** |  |
| *Environmental mastery* | 1 | 50.0 | *Environmental mastery* | 1 | 100.0 | *Coping* | 1 | 33.3 |
| Not identified/clear | 1 | 50.0 | *Environmental well-being* | 1 | 100.0 | *Daily functioning* | 1 | 33.3 |
|  |  |  |  |  |  | *Feelings about inner self* | 1 | 33.3 |
|  |  |  |  |  |  | *Future* | 1 | 33.3 |
|  |  |  |  |  |  | *Health outcomes* | 1 | 33.3 |
|  |  |  |  |  |  | *Living one’s life well* | 1 | 33.3 |
|  |  |  |  |  |  | *Meaning* | 1 | 33.3 |
|  |  |  |  |  |  | *Reflexivity* | 1 | 33.3 |
|  |  |  |  |  |  | *Relationships* | 1 | 33.3 |
|  |  |  |  |  |  | *Restriction of movement* | 1 | 33.3 |
|  |  |  |  |  |  | *Stand out/be different* | 1 | 33.3 |
|  |  |  |  |  |  | *Component of QoL* | 1 | 33.3 |
| Functional well-being | **3** |  | **Capability well-being** | **1** |  | **Cognitive well-being** | **4** |  |
| *Personal well-being (personal safety, personal care, leisure activities)* | 1 | 33.3 | *Attachment* | 1 | 100.0 | *Life satisfaction* | 3 | 75.0 |
| *Social well-being (fulfilling responsibilities, interacting with the world, social interaction)* | 1 | 33.3 | *Control* | 1 | 100.0 | *Environmental mastery* | 1 | 25.0 |
| *Component of QoL* | 2 | 66.7 | *Enjoyment* | 1 | 100.0 | Not identified/clear | 1 | 25.0 |
| Not identified/clear | 2 | 66.7 | *Role* | 1 | 100.0 |  |  |  |
|  |  |  | *Security* | 1 | 100.0 |  |  |  |
| Health-related well-being | **1** |  | **Medical well-being** | **2** |  | **Clinical well-being** | **1** |  |
| *Chronic mental health conditions* | 1 | 100.0 | *Medical care* | 2 | 100.0 | Not identified/clear | 1 | 100.0 |
| *Chronic physical health conditions* | 1 | 100.0 |  |  |  |  |  |  |
| *Health functioning* | 1 | 100.0 |  |  |  |  |  |  |
| *Satisfaction with health* | 1 | 100.0 |  |  |  |  |  |  |
| Vision-specific well-being | **1** |  | **Visual well-being** | **1** |  |  |  |  |
| *Component of QoL* | 1 | 100.0 | Not identified/clear | 1 | 100.0 |  |  |  |
| Not identified/clear | 1 | 100.0 |  |  |  |  |  |  |
